# Triterpenoids and Their Glycosides from *Glinus Oppositifolius* with Antifungal Activities against *Microsporum Gypseum* and *Trichophyton Rubrum*

**DOI:** 10.3390/molecules24122206

**Published:** 2019-06-12

**Authors:** Dongdong Zhang, Yao Fu, Jun Yang, Xiao-Nian Li, Myint Myint San, Thaung Naing Oo, Yuehu Wang, Xuefei Yang

**Affiliations:** 1 Southeast Asia Biodiversity Research Institute, Chinese Academy of Sciences, Yezin, Nay Pyi Taw 05282, Myanmar; zhangdongdong@mail.kib.ac.cn (D.Z.); fuyao@mail.kib.ac.cn (Y.F.); yangjuna@mail.kib.ac.cn (J.Y.); 2 Key Laboratory of Economic Plants and Biotechnology and Yunnan Key Laboratory for Wild Plant Resources, State Key Laboratory of Phytochemistry and Plant Resources in West China, Kunming Institute of Botany, Chinese Academy of Sciences, Kunming 650201, China; lixiaonian@mail.kib.ac.cn; 3 Forest Research Institute, Yezin, Nay Pyi Taw 05282, Myanmar; dawmyintmyintsanyezin@gmail.com (M.M.S.); tnoo71@gmail.com (T.N.O.)

**Keywords:** *Glinus oppositifolius*, triterpenoids and triterpenoid saponins, antifungal activity

## Abstract

Four new triterpenoids, 3β,12β,16β,21β,22-pentahydroxyhopane (**1**), 12β,16β,21β,22-tetrahydroxyhopan-3-one (**2**), 3-oxo-olean-12-ene-28,30-dioic acid (**3**), and 3β-hydroxyoleana-11,13(18)-diene-28,30-dioic acid 30-methyl ester (**4**); 21 new triterpenoid saponins, glinusopposides A–U (**5**–**25**); and 12 known compounds (**26**–**37**) were isolated from the whole plants of *Glinus oppositifolius*. The structures of the new compounds were elucidated based on the analysis of one-dimensional (1D) and two-dimensional (2D) nuclear magnetic resonance (NMR) and mass spectrometry (MS) data. All compounds from the plants were measured for antifungal activities against *Microsporum gypseum* and *Trichophyton rubrum*. Glinusopposide B (**6**), glinusopposide Q (**21**), glinusopposide T (**24**), and glinusopposide U (**25**) showed strong inhibitory activities against *M. gypseum* (MIC_50_ 7.1, 6.7, 6.8, and 11.1 μM, respectively) and *T. rubrum* (MIC_50_ 14.3, 13.4, 11.9, and 13.0 μM, respectively). For those active compounds with an oleanane skeleton, glycosylation (**21**–**26**) or oxidation (**3**) of 3-OH was helpful in increasing the activity; replacement of the 30-methyl group (**29**) by a carboxymethyl group (**26**) enhanced the activity; the presence of 11,13(18) double bonds (**20**) decreased the activity.

## 1. Introduction

Dermatophytosis is one of the most common skin diseases in animals and humans, which is mainly caused by *Epidermophyton*, *Microsporum* and *Trichophyton* [1,2]. As a chronic disease, dermatophytosis is difficult to treat due to the drug resistance developed by the related fungus [2]. Therefore, it is important to search for novel agents to treat dermatophytosis.

**Figure 1:**
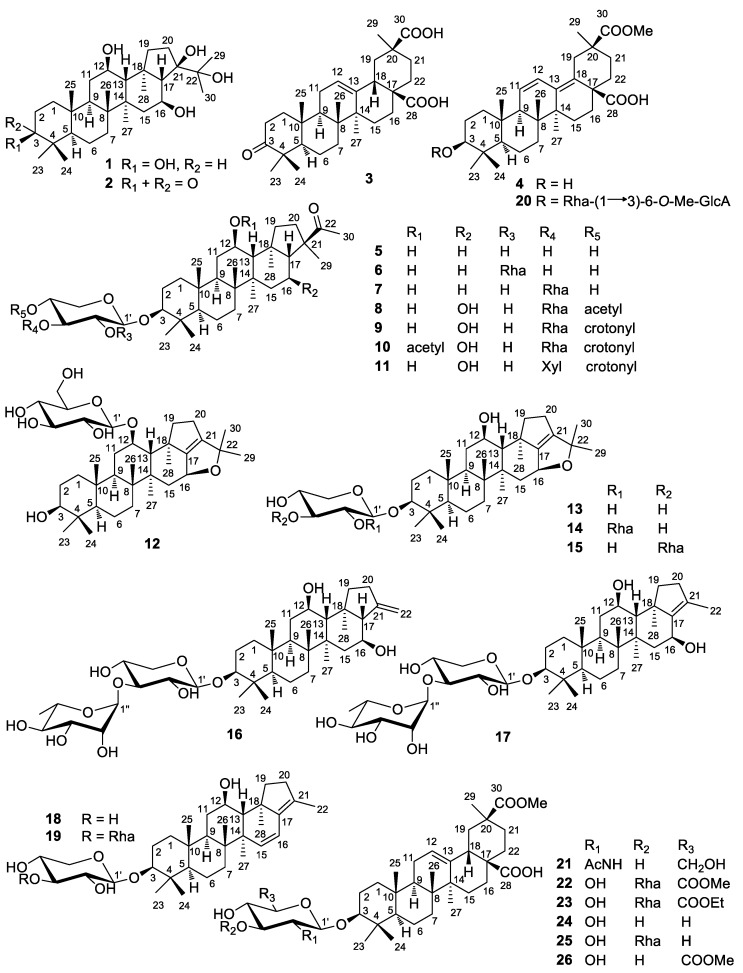
Chemical structures of compounds **1**–**26** from *Glinus oppositifolius.*

*Glinus oppositifolius* (L.) Aug. DC. (Syn: *Mollugo spergula* L. and *Mollugo oppositifolia* L; family: Molluginaceae) is a small herb widely distributed in tropical Asia, tropical Africa, and Australia [3]. Traditionally it has been used for treating skin and various infectious diseases in Bangladesh, China, India, Mali and Myanmar [4,5,6]. As a Chinese folk medicine, the whole plants of *G. oppositifolius* are used to treat diarrhea, coughs, hyperthermia, heat rashes, pinkeye, furuncles, snakebites, and burns [6]. The plant is reputed in Indian medicine due to its antiseptic and antidermatitic properties [7]. It is used to treat leprosy, leukoderma, heart, and skin diseases in the traditional medicine of Myanmar [5]. The major secondary metabolites from *G. oppositifolius* are triterpenoids and their glycosides, which exhibit α-glucosidase inhibitory [8], cytotoxic [9], and antiprotozoal activities [10]. There is little research reported the anti-fungal activities of *G. oppositifolius*. In this study, we isolated 25 new triterpenoids and triterpenoid saponins (Figure 1), along with 12 known compounds in the whole plants of *G. oppositifolius*. Their antifungal properties against *Microsporum gypseum* and *Trichophyton rubrum* were analyzed.

## 2. Results and Discussion

### 2.1. Structure Elucidation of the Compounds

Compound **1** had the molecular formula C_30_H_52_O_5_ based on ^13^C-NMR data (Table 1) and the positive ion at *m*/*z* 515.3718 [M + Na]^+^ (calcd. for C_30_H_52_NaO_5_, 515.3712) in the high resolution electrospray ionization mass spectroscopy (HRESIMS). The ^1^H-NMR spectrum showed resonances for eight methyl groups at *δ*_H_ 1.61 (s), 1.56 (s), 1.21 (s), 1.19 (s), 1.13 (s), 1.01 (s), 0.97 (s), and 0.82 (s), as well as three oxymethines at *δ*_H_ 4.48 (m), 4.23 (m), and 3.44 (br t, *J* = 8.3 Hz) (Table 1). The ^13^C-NMR spectrum showed resonances for thirty carbon atoms as expected from high resolution mass spectrum, which were sorted by DEPT into eight methyls, eight methylenes, seven methines (three oxymethines, *δ*_C_ 78.0_,_ 69.1, and 66.8), and seven quaternary carbons group, including two oxygenated quaternary carbons. These NMR data were very similar to those of a known hopane triterpenoid saponin from this plant, glinoside C, except for the lack of signals for glucopyranose [8]. The full NMR assignments and connections were determined by ^1^H-detected heteronuclear single quantum coherence (HSQC), ^1^H-detected heteronuclear multiple bond correlation (HMBC), and ^1^H-^1^H correlation spectroscopy (COSY) analyses.

**Figure 2:**
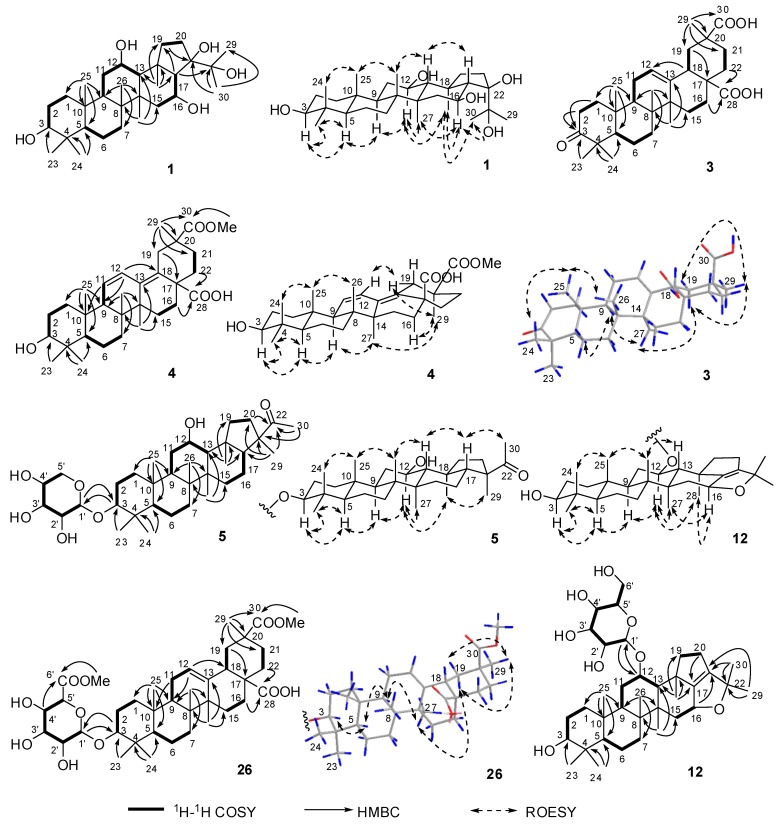
Key 2D-NMR correlations of **1**, **3**–**5**, **12**, and **26**.

**Figure 3:**
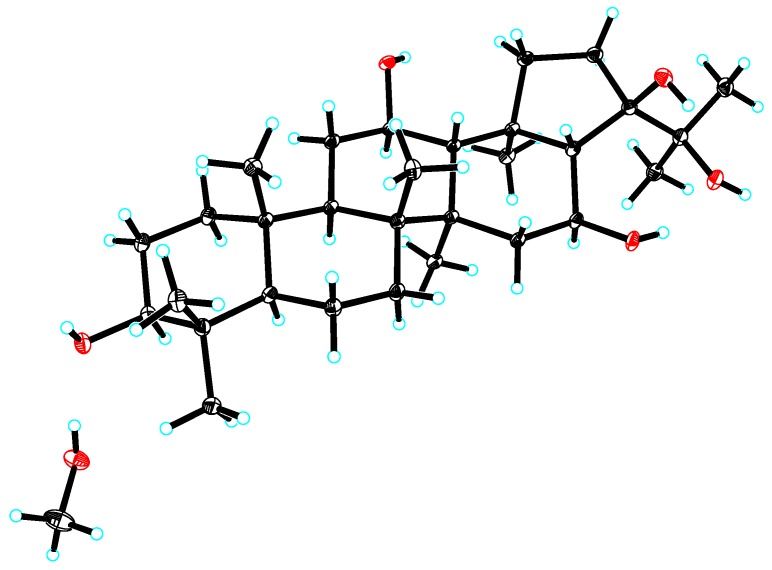
X-ray crystallographic structure of **1**.

According to the ^1^H–^1^H COSY correlations in the 2D spectra of **1** (Figure 2), five connections, C-1-C-2-C-3, C-5-C-6-C-7, C-9-C-11-C-12-C-13, C-15-C-16-C-17, and C-19-C-20, were confirmed. The planar structure of **1** was further deduced as 3,12,16,21,22-pentahydroxyhopane by the HMBC correlations from H_3_-23 and H_3_-24 to C-3, C-4, and C-5; from H_3_-25 to C-1, C-5, C-9, and C-10; from H_3_-26 to C-7, C-9, and C-14; from H_3_-27 to C-8, C-13, and C-15; from H_3_-28 to C-13, C-17, C-18, and C-19; from H_3_-29 and H_3_-30 to C-21 and C-22; and from H-17 to C-19 and C-22. The configurations of 3-OH, 12-OH, 16-OH, and 21-OH were deduced as 3β,12β,16β,21β by the key nuclear overhauser effect spectroscopy (ROESY) correlations of H-3/H-5, H-5/H-9, H-9/H-12, H-16/H_3_-27, H-16/H_3_-28, H-16/H_3_-30, and H_3_-30/H_3_-28. Thus, the structure of **1** was determined to be 3β,12β,16β,21β,22-pentahydroxyhopane. The absolute configuration was assigned by Cu Kα X-ray crystallographic analysis (Figure 3).

Compound **2** showed a molecular formula of C_30_H_50_O_5_ based on ^13^C-NMR data (Table 1) and the [M + Na]^+^ ion at *m*/*z* 513.3551 (calcd. for C_30_H_50_NaO_5_, 513.3556) in the HRESIMS. The NMR data (Table 1) of **2** were analogous to those of **1** except that the signal (*δ*_C_ 78.0) for an oxygenated methine in the ^13^C-NMR spectrum of **1** was replaced by the signal (*δ*_C_ 216.4) for a carbonyl group in the ^13^C-NMR spectrum of **2**. The structure of **2** was easily established as 12β, 16β, 21β, 22-tetrahydroxyhopan-3-one by the COSY, HMBC, and ROESY spectra of **2** (Supplementary Materials).

Compound **3** was assigned the molecular formula C_30_H_44_O_5_ based on ^13^C-NMR data (Table 2) and positive ion mode HRESIMS, which showed a pseudomolecular ion peak at *m/z* 507.3084 [M + Na]^+^ (calcd. for C_30_H_44_NaO_5_, 507.3086). The ^1^H-NMR data of **3** (Table 2) indicated the presence of six methyl groups at *δ*_H_ 1.43 (s), 1.30 (s), 1.14 (s), 1.00 (s), 0.99 (s), and 0.86 (s) and one olefinic group at *δ*_H_ 5.72. The ^13^C-NMR data of **3** (Table 2) indicated the presence of six methyl groups, two carboxylic carbons at *δ*_C_ 180.1 and 179.5, one carbonyl carbon at *δ*_C_ 216.3, and two olefinic carbons (one quaternary at *δ*_C_ 144.8 and one methane at *δ*_C_ 122.8, suggesting the presence of a double bond), 10 sp^3^ methylenes, three sp^3^ methines, and seven sp^3^ quaternary carbon atoms. The NMR data of **3** were very similar to those of 3-oxo-olean-12-en-28,29-dioic acid [11], implying that **3** was also an oleanane triterpenoid.

Six fragments, C-1-C-2, C-5-C-6-C-7, C-9-C-11-C-12, C-15-C-16, C-18-C-19, and C-21-C-22, were deduced from the ^1^H–^1^H COSY correlations in the 2D-NMR spectra of **3** (Figure 2). The structure of **3** was deduced as 3-oxo-olean-12-ene-28,30-dioic acid by the HMBC correlations from H_3_-23 and H_3_-24 to C-3, C-4, and C-5; from H_2_-1 and H_2_-2 to C-3; from H_3_-25 to C-1, C-5, C-9, and C-10; from H_3_-26 to C-7, C-9, and C-14; from H_3_-27 to C-8, C-13, and C-15; from H-18 to C-12, from H_2_-19 to C-17; from H_2_-16 and H_2_-22 to C-28; and from H_3_-29 to C-19, C-20, C-21 and C-30; as well as the key ROESY correlations of H-19α/H_3_-27 and H-19α/H_3_-29 (Figure 2).

The molecular formula of compound **4**, C_31_H_46_O_5_, with nine degrees of unsaturation, was determined by the ^13^C-NMR data in methanol-*d*_4_ (Table 2) and positive ion mode HRESIMS, which showed a pseudomolecular ion peak at *m/z* 521.3234 [M + Na]^+^ (calcd. for C_31_H_46_NaO_5_, 521.3237). The ^1^H-NMR data in methanol-*d*_4_ (Table 2) showed signals for six methyl groups at *δ*_H_ 1.11 (s), 1.00 (s), 0.98 (s), 0.94 (s), 0.81 (s), and 0.78 (s); a methoxy group at *δ*_H_ 3.67 (s); and a disubstituted double bond at *δ*_H_ 6.33 (dd, *J* = 11.2, 2.8 Hz) and 5.72 (br d, *J* = 11.2 Hz). The NMR data (Table 2) were very similar to those of 30-*O*-methyl spergulagenate (**27**) [12]. However, compound **4** had one more degree of unsaturation than 30-*O*-methyl spergulagenate, which was supported by four olefinic carbons at *δ*_C_ 139.8, 130.8, 129.0, and 126.2 for two double bonds in the ^13^C-NMR spectrum of **4** measured in methanol-*d*_4_. Finally, the structure of **4** was elucidated to be 3β-hydroxyoleana-11,13(18)-diene-28,30-dioic acid 30-methyl ester by the key HMBC correlations from H-11 to C-10, from H-12 to C-8, and from H_3_-27 to C-13, as well as the key ROESY correlations of H-3/H-5, H-5/H-9, H-9/H_3_-27, H_3_-29/H-19α, H_3_-29/H-16α, and H_3_-29/H_3_-27 (Figure 2).

The HRESIMS of glinusopposide A (**5**) indicated a molecular formula of C_35_H_58_O_7_, with the positive ion at *m*/*z* 613.4068 [M + Na]^+^ (calcd. for C_35_H_58_NaO_7_, 613.4080). By comparing its NMR data (Table 3) with those of spergulagenin A 3-*O*-β-d-xylopyranoside (**31**) [13], compound **5** might be combined by a modified hopane and a β-xylopyranose [*δ*_H_ 4.87 d (*J* = 7.6 Hz)]. The configuration of xylopyranose in the plant was determined as the d-configuration by acidic hydrolysis of **31** followed by acetylation to yield 1,2,3,4-tetra-*O*-acetyl-d-xylopyranose. The genin was deduced as 16-deoxyspergulagenin A by ^1^H–^1^H COSY, HMBC, and ROESY experiments. The ROESY correlations (Figure 2) of H-3/H-5, H-5/H-9, H-9/H-12, H-12/H_3_-28, and H_3_-28/H_3_-29 indicated that 3-OH, 12-OH, and Me-29 were β-, β-, and α-oriented, respectively. The xylose was located at 3-OH based on the HMBC correlations from H-3 to C-1′ and from H-1′ to C-3 (Figure 2). Finally, the structure of **5** was elucidated to be 16-deoxyspergulagenin A 3-*O*-β-d-xylopyranoside (glinusopposide A).

Both glinusopposides B (**6**) and C (**7**) have the same molecular formula, C_41_H_68_O_11_, based on ^13^C-NMR data (Table 3) and HRESIMS. The NMR data of **6** and **7** (Table 3) indicated the presence of the same genin in the two saponins as in compound **5**, with differences in the sugars. There are two sugars, β-d-xylopyranose and α-l-rhamnopyranose, in the structures of **6** and **7**. Base on the key HMBC correlations from H-1′′ to C-2′ and from H-1′ to C-3 in **6**, along with the correlations from H-1′′ to C-3′ and from H-1′ to C-3 in **7** (Supplementary Materials), the linkages between the two sugars were easily established to be Rha-(1→2)-Xyl-O-C-3 and Rha-(1→3)-Xyl-O-C-3 for saponins **6** and **7**, respectively. Therefore, the structures of **6** and **7** were determined to be 16-deoxyspergulagenin A 3-*O*-[α-l-rhamnopyranosyl-(1→2)]-β-d-xylopyranoside (glinusopposide B) and 16-deoxyspergulagenin A 3-*O*-[α-l-rhamnopyranosyl-(1→3)]-β-d-xylopyranoside (glinusopposide C), respectively.

Based on ^13^C-NMR data (Table 4) and HRESIMS, the molecular formulae of glinusopposides D–G (**8**–**11**) were deduced to be C_43_H_70_O_13_, C_45_H_72_O_13_, C_47_H_74_O_14_, and C_44_H_70_O_13_, respectively. By comparing their NMR data (Table 4) with those of spergulagenin A 3-*O*-β-d-xylopyranoside (**31**) [13], saponins **8–11** were deduced to be disaccharide glycosides of spergulagenin A. The presence of *trans*-2-butenoyl (crotonyl) group in **9** was confirmed by the ^1^H-NMR signals at *δ*_H_ 7.06 (m), 6.02 (dq, *J* = 15.6, 1.6 Hz), and 1.66 (3H, d, *J* = 6.8 Hz), along with COSY correlations of H-2′′′/H-3′′′ and H-3′′′/H-4′′′ (Supplementary Materials). The closely similar data and correlations can also be found in **10** and **11**, herein the *trans*-2-butenoyl group was assigned in **10** and **11** as same way. The *trans*-2-butenoyl moiety of **9**–**11** was located at C-4′ by the key HMBC correlation from H-4’ to C-1"’. According to the correlations in the ^1^H–^1^H COSY, HMBC, and ROESY spectra (Supplementary Materials), the structures of **8–11** were easily elucidated to be 3-*O*-[α-l-rhamnopyranosyl-(1→3)-4-*O*-acetyl-β-d-xylopyranosyl] spergulagenin A (glinusopposide D), 3-*O*-[α-l-rhamnopyranosyl-(1→3)-4-*O*-*trans*-2-butenoyl-β-d-xylopyranosyl] spergulagenin A (glinusopposide E), 3-*O*-[α-l-rhamnopyranosyl-(1→3)-4-*O*-*trans*-2-butenoyl-β-d-xylopyranosyl] 12-*O*-acetylspergulagenin A (glinusopposide F), and 3-*O*-[β-d-xylopyranosyl-(1→3)-4-*O*-*trans*-2-butenoyl-β-d-xylopyranosyl] spergulagenin A (glinusopposide G), respectively.

Glinusopposide H (**12**) was assigned the molecular formula C_36_H_58_O_8_, with eight degrees of unsaturation as determined by ^13^C-NMR data (Table 5) and the positive ion at *m/z* 641.4021 [M + Na]^+^ (calcd. for C_36_H_58_NaO_8_, 641.4024) in the HRESIMS. The ^1^H and ^13^C-NMR data indicated the compound might be a hopane triterpenoid saponin with eight methyl groups [*δ*_H_ 1.67 (s), 1.20 (s), 1.44 (s), 1.44 (s), 0.99 (s), 0.94 (s), 0.88 (s), and 0.79 (s)], one tetrasubstituted double bond (*δ*_C_ 152.5 and 146.8), and one β-glucopyranose [*δ*_H_ 5.19 (d, *J* = 7.6 Hz); *δ*_C_ 100.1, 78.8, 78.4, 75.9, 72.4, and 63.4]. In addition to the signals for the sugar, signals (*δ*_C_ 84.2, 78.1, 76.0, and 74.5) for four oxygenated carbon atoms were observed. The sugar was attached to C-12 based on the HMBC correlations from H-12 to C-1′ and from H-1′ to C-12, and the 17(21)-double bond was confirmed by the correlations from H_3_-28 to C-17 and from H_3_-29 and H_3_-30 to C-21 (Figure 2). The other three oxygenated carbon atoms were C-3, C-16, and C-22 based on the correlations from H_3_-23 and H_3_-24 to C-3, from H-16 to C-21, and from H_3_-29 and H_3_-30 to C-22. According to the deduced molecular formula and the degrees of unsaturation, a dihydrofuran ring containing the C-16-C-17-C-21-C-22-O fragment must be formed in the structure of **12**, which was further confirmed because of the shift in the ^13^C-NMR signals for C-16 (*δ*_C_ 76.0) and C-22 (*δ*_C_ 84.2) to downfield compared with the analogues **1**, **2**, and 22,24,28-trihydroxy-hop-17(21)-ene [14]. The 3β,12β,16β configurations were determined by the key ROESY correlations of H-3/H_3_-23, H-3/H-5, H-5/H-9, H-9/H-12, H-12/H_3_-27, H-12/H_3_-28, H-16/H_3_-27, and H-16/H_3_-28 (Figure 2). Thus, the structure of **12** was elucidated to be 3β,12β-dihydroxy-16β,22-epoxyhop-17(21)-ene 12-*O*-β-d-glucopyranoside (glinusopposide H).

Based on ^13^C-NMR data (Table 5 and Table 6) and HRESIMS, the molecular formulae of glinusopposides I–K (**13**–**15**) were deduced to be C_35_H_56_O_7_, C_41_H_66_O_11_, and C_41_H_66_O_11_, respectively. Comparison of the NMR data of **13**–**15** (Table 5 and Table 6) with those of **12** (Table 5) which are closely similar that were suggested these compounds with the same genin, 3β,12β-dihydroxy-16β,22-epoxyhop-17(21)-ene. The position of connectivity of sugars to sapogenin were established according to the correlations in the 2D-NMR spectra (Supplementary Materials). Therefore, the structures of saponins **13**–**15** were determined to be 3β,12β-dihydroxy-16β,22-epoxyhop-17(21)-ene 3-*O*-β-d-xylopyranoside (glinusopposide I), 3β,12β-dihydroxy-16β,22-epoxyhop-17(21)-ene 3-*O*-[α-l-rhamnopyranosyl-(1→2)]-β-d-xylopyranoside (glinusopposide J), and 3β,12β-dihydroxy-16β,22-epoxyhop-17(21)-ene 3-*O*-[α-l-rhamnopyranosyl-(1→3)]-β-d-xylopyranoside (glinusopposide K), respectively.

The HRESIMS of glinusopposide L (**16**) exhibited an ion peak at *m*/*z* 731.4353 [M + Na]^+^ (calcd. for C_39_H_64_NaO_11_, 731.4346), implying a molecular formula of C_39_H_64_O_11_. The NMR data of **16** (Table 6) were highly similar to those of spergulin B (**35**) [13], indicating that the compound might also be a bisnor hopane saponin with the same genin, spergulatriol, and the same sugars, xylose and rhamnose. The difference between the two saponins was the linkage mode of the two sugars. The rhamnose was linked to 3-OH of the inner sugar, xylose, based on the HMBC correlations from H-1″ to C-3′ and from H-3′ to C-1″ (Supplementary Materials). Finally, the structure of **16** was elucidated to be spergulatriol 3-*O*-[α-l-rhamnopyranosyl-(1→3)]-β-d-xylopyranoside (glinusopposide L).

According to ^13^C-NMR data (Table 6) and the positive ion HRESIMS at *m/z* 731.4347 [M + Na]^+^ (calcd. for C_39_H_64_NaO_11_, 731.4346), glinusopposide M (**17**) had the same molecular formula, C_39_H_64_O_11_, as saponin **16**. The 1D and 2D-NMR spectra (Supplementary Materials) indicated that **17** had a tetrasubstituted double bond rather than the terminal double bond of **16**. The 17(21) double bond was identified based on the HMBC correlations from H_3_-28 to C-17 and from H_3_-22 to C-17 and C-21. Therefore, the structure of **17** was determined to be 29,30-bisnor-3β,12β,16β-trihydroxyhop-17(21)-ene 3-*O*-[α-l-rhamnopyranosyl-(1 → 3)]-β-d-xylopyranoside (glinusopposide M).

According to ^13^C-NMR data (Table 7) and HRESIMS, the molecular formulae of glinusopposides N (**18**) and O (**19**) were deduced to be C_33_H_52_O_6_ and C_39_H_62_O_10_, respectively. Comparison of their NMR data (Table 7) with those of **17** indicated that saponins **18** and **19** were 29,30-bisnor hopane saponins with two double bonds and two hydroxy substitutions in the structure of the genin. 3β-OH and 12β-OH were determined based on the key HMBC correlations from H_3_-23 and H_3_-24 to C-3 and from H-9 to C-12, as well as the key ROESY correlations of H-3/H-5, H-5/H-9, H-9/H-12, H-12/H_3_-27, and H-12/H_3_-28 (Supplementary Materials). The 15,17(21) double bonds were identified by the HMBC correlations from H_3_-22 to C-17 and C-21, from H_3_-27 to C-15, from H_3_-28 to C-17, and from H-16 to C-14 and C-18. Finally, based on other correlations in the 2D-NMR spectra (Supplementary Materials), **18** and **19** were elucidated to be 29,30-bisnor-3β,12β-dihydroxyhopa-15,17(21)-diene 3-*O*-β-d-xylopyranoside (glinusopposide N) and 29,30-bisnor-3β,12β-dihydroxyhopa-15,17(21)-diene 3-*O*-[α-l-rhamnopyranosyl-(1→3)]-β-d-xylopyranoside (glinusopposide O), respectively.

The molecular formula of glinusopposide P (**20**) was determined to be C_44_H_66_O_15_ based on ^13^C-NMR data (Table 7) and the positive ion at *m/z* 857.4300 [M + Na]^+^ (calcd. for C_44_H_66_NaO_15_, 857.4299) in the HRESIMS. The NMR data (Table 7) indicated a moiety of 3β-hydroxyoleana-11,13(18)-diene-28,30-dioic acid 30-methyl ester (**4**), an α-rhamnopyranosyl group [*δ*_H_ 6.33 (br s), 5.08 (m), 4.76 (dd, *J* = 3.3, 1.4 Hz), 4.57 (dd, *J* = 9.3, 3.3 Hz), 4.35 (dd, *J* = 9.3, 9.3 Hz), and 1.71 (d, *J* = 6.1 Hz); *δ*_C_ 103.4, 74.6, 73.2, 73.0, 70.3, and 19.1], and a 6-*O*-methyl-β-glucuronopyranosyl group [*δ*_H_ 4.92 (d, *J* = 7.9 Hz), 4.58 (d, *J* = 9.3 Hz), 4.45 (dd, *J* = 9.3, 8.7 Hz), 4.41 (dd *J* = 9.3, 9.3 Hz), 4.07 (dd, *J* = 8.7, 7.9 Hz), and 3.79 (s); *δ*_C_ 171.3, 107.6, 82.3, 77.6, 76.2, 71.9, and 52.7]. The linkage of the sugar chain was determined to be Rha-(1→3)-[6-*O*-methyl-GlcA]-*O*-C-3 based on the key HMBC correlations from H-1″ to C-3′, from H-3′ to C-1″, from H-1′ to C-3, and from H-3 to C-1′ (Supplementary Materials). Thus, the structure of **20** was elucidated to be 3-*O*-[α-l-rhamnopyranosyl-(1→3)-6-*O*-methyl-β-d-glucuronopyranosyl]-3β-hydroxyoleana-11,13(18)-diene-28,30-dioic acid 30-methyl ester (glinusopposide P).

Based on ^13^C-NMR data (Table 8 and Table 9) and HRESIMS, the molecular formulae of glinusopposides Q–U (**21**–**25**) were deduced to be C_39_H_61_NO_10_, C_44_H_68_O_15_, C_45_H_70_O_15_, C_36_H_56_O_9_, and C_42_H_66_O_13_, respectively. By comparing their NMR data with those of 30-*O*-methyl spergulagenate (**27**) [12], these saponins were determined to have the same genin, 30-*O*-methyl spergulagenate. The NMR signals of **21** at *δ*_H_ 8.94 (d, *J* = 9.0 Hz) and 2.15 (s), along with *δ*_C_ 170.3 and 23.8 manifested the presence of an acetylamino unit which was further confirmed by the HMBC correlations from *δ*_H_ 2.15 (H-2″) to *δ*_C_ 170.3 (C-1″) and from *δ*_H_ 8.94 (NH) to *δ*_C_ 170.3 (C-1″). The position of the acetylamino moiety of **21** was determined by the HMBC correlation from H-2’ to C-1″. The location of the sugar in **21** was also confirmed by the HMBC correlations from H-3 to C-1’ and H-1’ to C-3. Two anomeric carbons at *δ*_C_ 107.1 and 102.9 of **22** suggested that the presence of two sugars, of which the positions were assigned by the key HMBC from H-3’ to C-1″, from H-1″ to C-3’, from H-1′ to C-3, and from H-3 to C-1’. The NMR date of **23** were almost identical to those of **22** except for the replacement of the methoxy group in **22** by ethoxy group (*δ*_C_ 61.4 and 14.3). Comparison of NMR data of 30-*O*-methyl spergulagenate (**27**), signals for an additional sugar in compound **24** and for two additional sugars in compound **25** were observed. According to these correlations in the 2D-NMR spectra (Supplementary Materials), saponins **21**–**25** were determined as 3-*O*-(2-acetylamino-2-deoxy-β-d-glucopyranosyl)-30-*O*-methyl spergulagenate (glinusopposide Q), 3-*O*-[α-l-rhamnopyranosyl-(1→3)-6-*O*-methyl-β-d-glucuronopyranosyl]-30-*O*-methyl spergulagenate (glinusopposide R), 3-*O*-[α-l-rhamnopyranosyl-(1→3)-6-*O*-ethyl-β-d-glucuronopyranosyl]-30-*O*-methyl spergulagenate (glinusopposide S), 30-*O*-methyl spergulagenate 3-*O*-β-d-xylopyranoside (glinusopposide T), and 30-*O*-methyl spergulagenate 3-*O*-[α-l-rhamnopyranosyl-(1→3)]-β-d-xylopyranoside (glinusopposide U), respectively.

The NMR data of compound **26** in methanol-*d*_4_ (Supplementary Materials) were the same as those of coryternic acid 3-*O*-β-d-glucuronopyranoside-6′-*O*-methyl ester [3β-*O*-(6-*O*-methyl-β-d-glucuronopyranosyl)-olean-12-ene-28,29-dioic acid 29-methyl ester] [15]. Based on the 1D and 2D-NMR spectra of **26** both in methanol-*d*_4_ and pyridine-*d*_5_ (Table 9, Figure 2, and Supplementary Materials), especially on the ROSEY correlations of H_3_-29/H-19α and H-19α/H_3_-27, the structure of **26** was determined to be 3β-*O*-(6-*O*-methyl-β-d-glucuronopyranosyl)-olean-12-ene-28,30-dioic acid 30-methyl ester. Therefore, the structure of coryternic acid 3-*O*-β-d-glucuronopyranoside-6′-*O*-methyl ester reported in the literature is suggested to be revised to 3β-*O*-(6-*O*-methyl-β-d-glucuronopyranosyl)-olean-12-ene-28,30-dioic acid 30-methyl ester.

Other known compounds, 30-*O*-methyl spergulagenate (**27**) [12], 28-β-d-glucopyranosyl-30-methyl 3β-hydroxyolean-12-en-28,30-dioate (**28**) [16], oleanolic acid 3-*O*-6′-*O*-methyl-β-d-glucuronopyranoside (**29**) [17], oppositifolone (**30**) [18], spergulagenin A 3-*O*-β-d-xylopyranoside (**31**) [13], spergulin A (**32**) [13], spergulacin A (**33**) [13], spergulacin (**34**) [13], spergulin B (**35**) [13], grasshopper ketone (**36**) [19], and β-carboline (**37**) [20], were determined by comparing their NMR data (for all compounds) and optical rotation values (for all compounds except **37**) with those reported in the literature.

### 2.2. Biological Evaluation

We proposed antifungal activities of *G. oppositifolius* according to traditional healthcare use. The 70% ethanol extract of the whole plants of *G. oppositifolius* showed inhibitory activity against *M. gypseum* with an inhibition of 23.0 ± 1.9% at a concentration of 128 μg/mL. All the isolated compounds (**1**–**37**) were measured for antifungal activities against *M. gypseum* and *T. rubrum*, and the results are presented in Table 10. Glinusopposide B (**6**), glinusopposide Q (**21**), glinusopposide T (**24**), and glinusopposide U (**25**) showed the most notable inhibitory activities against *M. gypseum* (MIC_50_ 7.1, 6.7, 6.8, and 11.1 μM, respectively) and *T. rubrum* (MIC_50_ 14.3, 13.4, 11.9, and 13.0 μM, respectively) compared with the positive control terbinafine hydrochloride (MIC_50_ 0.008 μM against *M. gypseum* and 1.647 μM against *T. rubrum*). Glinusopposide K (**15**), glinusopposide N (**18**), glinusopposide O (**19**), glinusopposide R (**22**), glinusopposide S (**23**), glinusopposide U (**25**), and 3β-*O*-(6-*O*-methyl-β-d-glucuronopyranosyl)-olean-12-ene-28,30-dioic acid 30-methyl ester (**26**) showed moderate inhibitory activities against *M. gypseum*, with MIC_50_ values ranging from 22.0 to 46.8 μM. Additionally, 3β,12β,16β,21β,22-pentahydroxyhopane (**1**), 3-oxo-olean-12-ene-28,30-dioic acid (**3**), glinusopposide H (**12**), and glinusopposide L (**16**) showed weak activities against *M. gypseum*, with MIC_50_ values ranging from 105.0 to 260.1 μM. Other compounds did not display activity against *M. gypseum* or *T. rubrum* (MIC_50_ > 300 μM).

The active compounds of *G. oppositifolius* against *M. gypseum* and *T. rubrum* have two types of carbon skeletons, hopane and oleanane. For those oleanane-type compounds, glycosylation (**21**–**26**) or oxidation (**3**) of 3-OH was helpful in increasing the activity based on a comparison of the MIC_50_ values of **3** and **21**–**26** with those of **27** and **28**. Replacement of the 30-methyl group (**29**) with a carboxymethyl group (**26**) enhanced the activity. The presence of 11,13(18) double bonds (**20**) decreased the activity. The structure-activity relationships (SARs) of the hopane-type compounds against the two fungi were not clear.

## 3. Experimental Section

### 3.1. General Experimental Procedures

This part can be found in the Supplementary Materials.

### 3.2. Plant Material

Whole plants of *G. oppositifolius* were bought from Zay cho market of Mandalay in Myanmar, in December 2015. The plants were identified by author, Jun Yang. A voucher specimen (No. MD1612078) was deposited at the Yunnan Key Laboratory for Wild Plant Resources, Kunming Institute of Botany, Chinese Academy of Sciences.

### 3.3. Extraction and Isolation

Powdered whole plants of *G. oppositifolius* (3.0 kg) were extracted with 70% EtOH at 60 °C for six times (each for 4 h) to obtain a crude extract (650.1 g), which was suspended in H_2_O and then extracted with petroleum ether. The water-soluble phase was adjusted to pH 1−2 with 1% HCl and then partitioned with EtOAc to afford the EtOAc-soluble extract (B, 130.0 g). The aqueous phase was basified with 5% NaOH solution to pH 9−10 and then extracted with CHCl_3_ to yield the CHCl_3_-soluble extract (A, 25.2 g). The aqueous phase was extracted with *n*-butanol to yield the *n*-butanol-soluble extract (C, 142.0 g).

The CHCl_3_ extract (A, 25.2 g) was subjected to silica gel column chromatography (CC, CH_2_Cl_2_-MeOH, 50:1→0:1, *v*/*v*) to yield four main fractions A1–A4. Fraction A1 (931.1 mg) was subjected to reversed phase (RP-C_18_) silica gel CC eluted with MeOH-H_2_O (30%→100%). The 30% MeOH-eluted part (86.3 mg) was separated on a Sephadex LH-20 CC (MeOH) and purified by semipreparative HPLC (Welch Ultimate AQ-C_18_, MeOH-H_2_O, 18:82, 0.8 mL/min) to yield **36** (6.0 mg, *t*_R_ = 56.431 min). The 60% MeOH-eluted part (399.8 mg) was separated on a Sephadex LH-20 CC (MeOH) to yield **1** (4.9 mg), **2** (4.7 mg), and **31** (24.6 mg) recrystallized from MeOH, as well as **37** (0.4 mg) recrystallized from CH_2_Cl_2_. The 80% MeOH-eluted part (127.3 mg) was purified by Sephadex LH-20 CC (CH_2_Cl_2_-MeOH, 1:1) and semipreparative HPLC (Agilent Zorbax SB-C_18_, MeOH-H_2_O (containing 0.05% TFA), 67:33, 2 mL/min) to obtain **11** (1.1 mg, *t*_R_ = 36.803 min) and **9** (5.7 mg, *t*_R_ = 43.850 min). Fraction A2 (2.1 g) was separated on an RP-18 silica gel CC eluted with MeOH-H_2_O (30%→100%). The 70% MeOH-eluted part (96.8 mg) was purified by silica gel CC (CH_2_Cl_2_-MeOH-H_2_O, 300:10:1) to yield **8** (24.3 mg) recrystallized from MeOH. Fraction A3 (2.5 g) was separated on an RP-18 silica gel CC eluted with MeOH-H_2_O (20%→100%). The 60% MeOH-eluted part (965.5 mg) was purified by silica gel CC (EtOAc-MeOH, 20:1) to yield two main subfractions (A3-1 and A3-2). The subfraction A3-1 (91.8 mg) was purified by silica gel CC (CH_2_Cl_2_-MeOH, 10:1) and semipreparative HPLC (Agilent Zorbax SB-C_18_, CH_3_CN-H_2_O, 35:65, 2 mL/min) to yield **17** (1.0 mg, *t*_R_ = 28.240 min). The subfraction A3-2 (192.0 mg) was purified by silica gel CC (CH_2_Cl_2_-MeOH-H_2_O, 80:10:1) to yield two further subfractions (A3-2-1 and A3-2-2). The subfraction A3-2-1 (33.1 mg) was purified by semipreparative HPLC (Agilent Zorbax SB-C_18_, MeCN-H_2_O, 30:70, 2 mL/min) to yield **34** (16.6 mg, *t*_R_ = 31.175 min) and **16** (7.6 mg, *t*_R_ = 45.162 min). The subfraction A3-2-2 (7.0 mg) was purified by semipreparative HPLC (Welch Ultimate AQ-C_18_, MeCN-H_2_O, 30:70, 1 mL/min) to yield **35** (1.4 mg, *t*_R_ = 7.176 min). Fraction A4 (3.0 g) was separated on an RP-C_18_ silica gel CC eluted with MeOH-H_2_O (20%→100%) to yield two further subfractions. The 40% MeOH-eluted part (217.9 mg) was purified by silica gel CC (CH_2_Cl_2_-MeOH-H_2_O, 70:10:1) to yield **32** (38.4 mg). The 50% MeOH-eluted part (494.4 mg) was recrystallized from MeOH to yield **33** (290.3 mg).

The part of EtOAc extract (B, 27.0 g) was separated on an RP-18 silica gel CC eluted with MeOH-H_2_O (5%→100%) to yield five main fractions (B1–B5). The 50% MeOH-eluted part (B1, 2.7 g) was purified by silica gel CC (CH_2_Cl_2_-MeOH, 50:1→30:1) to afford **30** (26.8 mg). The 60% MeOH-eluted part (B2, 545.7 mg) was purified by silica gel CC (CH_2_Cl_2_-MeOH, 30:1) to yield **12** (5.5 mg). The 70% MeOH-eluted part (B3, 2.5 g) was purified by silica gel CC (CH_2_Cl_2_-MeOH, 30:1→20:1) to afford **28** (11.1 mg) and three main subfractions (B3-1–B3-3). Subfraction B3-1 (29.0 mg) was purified by semipreparative HPLC (Agilent Zorbax SB-C_18_, MeCN-H_2_O (containing 0.05% TFA), 47:53, 2 mL/min) and further by semipreparative HPLC (Agilent Zorbax SB-C_18_, MeOH-H_2_O (containing 0.05% TFA), 75:25, 2 mL/min) to yield **13** (3.9 mg, *t*_R_ = 30.891 min) and **5** (2.1 mg, *t*_R_ = 41.804 min). Subfraction B3-2 (91.7 mg) was purified by silica gel CC (CH_2_Cl_2_-MeOH, 30:1) and semipreparative HPLC (Agilent Zobrax SB-C_18_, MeCN-H_2_O (containing 0.05% TFA), 50:50, 2 mL/min) to yield **7** (5.3 mg, *t*_R_ = 14.414 min) and **15** (5.3 mg, *t*_R_ = 16.844 min). Subfraction B3-3 (459.4 mg) was purified by silica gel CC (CH_2_Cl_2_-MeOH, 30:1) and semipreparative HPLC (Agilent Zorbax SB-C_18_, MeCN-H_2_O, 45:55, 2 mL/min) to yield **21** (5.7 mg, *t*_R_ = 12.996 min), **6** (5.9 mg, *t*_R_ = 16.490 min), and **14** (3.6 mg, *t*_R_ = 17.736 min). The 80% MeOH-eluted part (B4, 1.1 g) was purified by silica gel CC (CH_2_Cl_2_-MeOH, 30:1) to yield two main subfractions (B4-1 and B4-2). Subfraction B4-1 (97.3 mg) was purified by semipreparative HPLC (Agilent Zorbax SB-C_18_, MeCN-H_2_O (containing 0.05% TFA), 45:55, 2 mL/min) to afford **24** (18.3 mg, *t*_R_ = 42.714 min), **26** (29.0 mg, *t*_R_ = 51.174 min), and **10** (1.6 mg, *t*_R_ = 62.511 min). Subfraction B4-2 (153.1 mg) was purified by semipreparative HPLC (Agilent Zorbax SB-C_18_, MeCN-H_2_O (containing 0.05% TFA), 43:57, 2 mL/min) to yield **25** (12.4 mg, *t*_R_ = 37.244 min), **22** (42.6 mg, *t*_R_ = 45.406 min), **23** (16.2 mg, *t*_R_ = 66.231 min), and a mixture. The mixture was purified by semipreparative HPLC (Agilent Eclipse XDB-C_18_, MeCN-H_2_O (containing 0.05% TFA), 40:60, 1 mL/min) to yield **20** (3.5 mg, *t*_R_ = 18.741 min). The 90% MeOH-eluted part (B5, 1.1 g) was separated on a Sephadex LH-20 CC (MeOH) to yield two main subfractions (B5-1 and B5-2). Subfraction B5-1 (535.3 mg) was purified by silica gel CC (CH_2_Cl_2_-MeOH, 30:1) and semipreparative HPLC (Agilent Zorbax SB-C_18_, MeCN/H_2_O (containing 0.05% TFA), 49:51, 2 mL/min) to yield **19** (3.9 mg, *t*_R_ = 43.926 min). Subfraction B5-2 (244.5 mg) was purified by silica gel CC (petroleum ether-EtOAc, 2:1→1:1) to yield two further subfractions (B5-2-1 and B5-2-2). Subfraction B5-2-1 (52.9 mg) was purified by semipreparative HPLC (Agilent Zorbax SB-C_18_, MeCN-H_2_O (containing 0.05% TFA), 60:40, 2 mL/min) to yield **3** (7.6 mg, *t*_R_ = 35.867 min), **4** (3.5 mg, *t*_R_ = 47.842 min), and **27** (21.0 mg, *t*_R_ = 53.439 min). Subfraction B5-2-2 (20.2 mg) was purified by semipreparative HPLC (Agilent Zorbax SB-C_18_, MeCN-H_2_O (containing 0.05% TFA), 70:30, 2 mL/min) to yield **29** (0.7 mg, *t*_R_ = 15.194 min) and **18** (3.1 mg, *t*_R_ =16.328 min).

### 3.4. Spectroscopic and Physical Data

3β,12β,16β,21β,22-Pentahydroxyhopane (**1**). Colorless blocks (Me-H_2_O, 10:1); ${\left[\alpha \right]}_{D}^{25}$ −18 (*c* 0.05, MeOH); UV (MeOH) *λ*_max_ (log*ε*) 203 (3.34) nm; ECD (*c* 0.05, MeOH) *λ*_max_ (Δ*ε*) 240 (+0.35), 226 (−0.36), 198 (+1.15) nm; ^1^H and ^13^C-NMR data, see Table 1; ESIMS *m/z* 515 [M + Na]^+^; HRESIMS *m/z* 515.3718 [M + Na]^+^ (calcd for C_30_H_52_NaO_5_, 515.3712).

Crystal data for **1**: C_30_H_52_O_5_, *M* = 524.75, *a* = 7.8358(3) Å, *b* = 17.7389(6) Å, *c* = 20.4841(7) Å, *α* = 90°, *β* = 90°, *γ* = 90°, *V* = 2847.26(17) Å^3^, *T* = 100.(2) K, space group *P*212121, *Z* = 4, *μ*(Cu Kα) = 0.653 mm^−1^, 51,068 reflections measured, 5642 independent reflections (*R_int_* = 0.0514). The final *R_1_* values were 0.0363 (*I* > 2*σ*(*I*)). The final *wR*(*F*^2^) values were 0.1081 (*I* > 2*σ*(*I*)). The final *R_1_* values were 0.0371 (all data). The final *wR*(*F*^2^) values were 0.1102 (all data). The goodness of fit on *F*^2^ was 0.998. Flack parameter = 0.02(5). The supplementary crystallographic data can be obtained free of charge from the Cambridge Crystallographic Data Centre (CCDC) (deposition number CCDC 1917520) via http://www.ccdc.cam.ac.uk.

12β,16β,21β,22-Tetrahydroxyhopan-3-one (**2**). White powder; ${\left[\alpha \right]}_{D}^{25}$ +2 (*c* 0.12, MeOH); UV (MeOH) *λ*_max_ (log*ε*) 280 (1.98), 219 (2.73), 203 (2.91) nm; ECD (*c* 0.12, MeOH) *λ*_max_ (Δ*ε*) 289 nm (+0.17), 232 (+0.28), 208 (–0.29) nm; IR *ν*_max_ (KBr) 3443, 3427, 1690, 1452, 1385, 1084, 1047, 879 cm^−1^; ^1^H and ^13^C-NMR data, see Table 1; ESIMS *m/z* 513 [M + Na]^+^; HRESIMS *m/z* 513.3551 [M + Na]^+^ (calcd for C_30_H_50_NaO_5_, 513.3556).

The 3-Oxo-olean-12-ene-28,30-dioic acid (**3**). White powder; ${\left[\alpha \right]}_{D}^{25}$ +59 (*c* 0.26, MeOH); UV (MeOH) *λ*_max_ (log*ε*) 371 (1.73), 252 (2.75), 239 (2.72) nm; ^1^H and ^13^C-NMR data, see Table 2; ESIMS *m/z* 507 [M + Na]^+^; HRESIMS *m/z* 507.3084 [M + Na]^+^ (calcd for C_30_H_44_NaO_5_, 507.3086).

The 3β-Hydroxyoleana-11,13(18)-diene-28,29-dioic acid 29-methyl ester (**4**). White powder; ${\left[\alpha \right]}_{D}^{26}$–7 (*c* 0.15, MeOH); UV (MeOH) *λ*_max_ (log*ε*) 250 (2.65), 242 (2.59) nm; ECD (*c* 0.09, MeOH) *λ*_max_ (Δ*ε*) 250 (–3.92) nm; ^1^H and ^13^C-NMR data, see Table 2; ESIMS *m/z* 521 [M + Na]^+^; HRESIMS *m/z* 521.3234 [M + Na]^+^ (calcd for C_31_H_46_NaO_5_, 521.3237).

Glinusopposide A (**5**). White powder; ${\left[\alpha \right]}_{D}^{26}$ –8 (*c* 0.05, MeOH); ^1^H and ^13^C-NMR data, see Table 3; ESIMS *m/z* 613 [M + Na]^+^; HRESIMS: *m/z* 613.4068 [M + Na]^+^ (calcd for C_35_H_58_NaO_7_, 613.4080).

Glinusopposide B (**6**). White powder; ${\left[\alpha \right]}_{D}^{25}$ –20 (*c* 0.1, MeOH); ^1^H and ^13^C-NMR data, see Table 3; ESIMS *m/z* 759 [M + Na]^+^; HRESIMS *m/z* 759.4650 [M + Na]^+^ (calcd for C_41_H_68_NaO_11_, 759.4659).

Glinusopposide C (**7**). White powder; ${\left[\alpha \right]}_{D}^{26}$ –6 (*c* 0.35, MeOH); ^1^H and ^13^C-NMR data, see Table 3; ESIMS *m/z* 759 [M + Na]^+^; HRESIMS *m/z* 759.4656 [M + Na]^+^ (calcd for C_41_H_68_NaO_11_, 759.4659).

Glinusopposide D (**8**). White powder; ${\left[\alpha \right]}_{D}^{21}$ –36 (*c* 0.16, pyridine); UV (MeOH) *λ*_max_ (log*ε*) 275 (2.77), 245 (3.02), 204 (3.59) nm; ECD (*c* 0.099, MeOH) *λ*_max_ (Δ*ε*) 284 (+1.00), 249 (–0.17), 218 (+1.03), 197 (–1.11) nm; ^1^H and ^13^C-NMR data, see Table 4; ESIMS *m/z* 833 [M + K]^+^, 817 [M + Na]^+^; HRESIMS *m/z* 817.4709 [M + Na]^+^ (calcd for C_43_H_70_NaO_13_, 817.4714).

Glinusopposide E (**9**). White powder; ${\left[\alpha \right]}_{D}^{20}$ –32 (*c* 0.09, MeOH); UV (MeOH) *λ*_max_ (log*ε*) 415 (2.39), 206 (4.09) nm; ECD (*c* 0.065, MeOH) *λ*_max_ (Δ*ε*) 284 (+0.48), 206 (+0.73), 200 (–1.80) nm; IR *ν*_max_ (KBr) 3442, 3428, 1677, 1644, 1449, 1431, 1385, 1202, 1144, 1086, 1047, 879 cm^−1^; ^1^H and ^13^C-NMR data, see Table 4; ESIMS *m/z* 843 [M + Na]^+^; HRESIMS *m/z* 843.4867 [M + Na]^+^ (calcd for C_45_H_72_NaO_13_, 843.4871).

Glinusopposide F (**10**). White solid; ${\left[\alpha \right]}_{D}^{25}$ –31 (*c* 0.04, MeOH); ^1^H and ^13^C-NMR data, see Table 4; ESIMS *m/z* 885 [M + Na]^+^; HRESIMS *m/z* 885.4930 [M + Na]^+^ (calcd for C_47_H_74_NaO_14_, 885.4976).

Glinusopposide G (**11**). White powder; ${\left[\alpha \right]}_{D}^{20}$ –7 (*c* 0.15, MeOH); UV (MeOH) *λ*_max_ (log*ε*) 252 (3.21), 206 (3.81) nm; ECD (*c* 0.078, MeOH) *λ*_max_ (Δ*ε*) 201 (–1.59), 197 (+1.29) nm; IR *ν*_max_ (KBr) 3448, 3427, 1639, 1447, 1383, 1084, 1046, 879 cm^−1^; ^1^H and ^13^C-NMR data, see Table 4; ESIMS *m/z* 829 [M + Na]^+^; HRESIMS *m/z* 829.4711 [M + Na]^+^ (calcd for C_44_H_70_NaO_13_, 829.4714).

Glinusopposide H (**12**). White powder; ${\left[\alpha \right]}_{D}^{25}$ +7 (*c* 0.28, MeOH); ^1^H and ^13^C-NMR data, see Table 5; ESIMS *m/z* 641 [M + Na]^+^; HRESIMS *m/z* 641.4021 [M + Na]^+^ (calcd for C_36_H_58_NaO_8_, 641.4024).

Glinusopposide I (**13**). White powder; ${\left[\alpha \right]}_{D}^{25}$ –10 (*c* 0.07, MeOH); ^1^H and ^13^C-NMR data, see Table 5; ESIMS *m/z* 611 [M + Na]^+^; HRESIMS *m/z* 611.3898 [M + Na]^+^ (calcd for C_35_H_56_NaO_7_, 611.3924).

Glinusopposide J (**14**). White powder; ${\left[\alpha \right]}_{D}^{25}$ –22 (*c* 0.11, MeOH); ^1^H and ^13^C-NMR data, see Table 5; ESIMS: *m/z* 757 [M + Na]^+^; HRESIMS *m/z* 757.4486 [M + Na]^+^ (calcd for C_41_H_66_NaO_11_, 757.4503).

Glinusopposide K (**15**). White powder; ${\left[\alpha \right]}_{D}^{25}$ –16 (*c* 0.13, MeOH); ^1^H and ^13^C-NMR data, see Table 6; ESIMS *m/z* 757 [M + Na]^+^; HRESIMS *m/z* 757.4510 [M+ Na]^+^ (calcd for C_41_H_66_NaO_11_, 757.4503).

Glinusopposide L (**16**). White powder; ${\left[\alpha \right]}_{D}^{25}$ –25 (*c* 0.1, MeOH); UV (MeOH) *λ*_max_ (log*ε*) 203 (3.82) nm; ECD (*c* 0.076, MeOH) *λ*_max_ (Δ*ε*) 196 (–6.89) nm; IR *ν*_max_ (KBr) 3425, 1632, 1454, 1385, 1129, 1094, 1047, 974 cm^−1^; ^1^H and ^13^C-NMR data, see Table 6; ESIMS *m/z* 731 [M + Na]^+^; HRESIMS *m/z* 731.4353 [M + Na]^+^ (calcd for C_39_H_64_NaO_11_, 731.4346).

Glinusopposide M (**17**). White powder; ${\left[\alpha \right]}_{D}^{20}$ –25 (*c* 0.05, MeOH); UV (MeOH) *λ*_max_ (log*ε*) 203 (3.54) nm; ECD (*c* 0.05, MeOH) *λ*_max_ (Δ*ε*) 206 (–4.38), 196 (+4.32) nm; IR *ν*_max_ (KBr) 3443, 3426, 1639, 1453, 1421, 1384, 1084, 1047, 879 cm^−1^; ^1^H and ^13^C-NMR data, see Table 6; ESIMS *m/z* 731 [M + Na]^+^; HRESIMS *m/z* 731.4347 [M + Na]^+^ (calcd for C_39_H_64_NaO_11_, 731.4346).

Glinusopposide N (**18**). White powder; ${\left[\alpha \right]}_{D}^{26}$ –7 (*c* 0.18, MeOH); UV (MeOH) *λ*_max_ (log*ε*) 250 (3.76), 213 (3.38) nm; ^1^H and ^13^C-NMR data, see Table 7; ESIMS *m/z* 567 [M + Na]^+^; HRESIMS *m/z* 567.3655 [M + Na]^+^ (calcd for C_33_H_52_NaO_6_, 567.3656).

Glinusopposide O (**19**). White powder; ${\left[\alpha \right]}_{D}^{25}$ –14 (*c* 0.13, MeOH); UV (MeOH) *λ*_max_ (log*ε*) 250 (3.76), 218 (3.43) nm; ^1^H and ^13^C-NMR data, see Table 7; ESIMS *m/z* 729 [M + K]^+^, 713 [M + Na]^+^; HRESIMS *m/z* 713.4200 [M + Na]^+^ (calcd for C_39_H_62_NaO_10_, 713.4241).

Glinusopposide P (**20**). White powder; ${\left[\alpha \right]}_{D}^{25}$ –39 (*c* 0.1, MeOH); UV (MeOH) *λ*_max_ (log*ε*) 250 (4.07), 217 (3.68) nm; ^1^H and ^13^C-NMR data, see Table 7; ESIMS *m/z* 857 [M + Na]^+^; HRESIMS *m/z* 857.4300 [M + Na]^+^ (calcd for C_44_H_66_NaO_15_, 857.4299).

Glinusopposide Q (**21**). White powder; ${\left[\alpha \right]}_{D}^{25}$ +40 (*c* 0.13, MeOH); ^1^H and ^13^C-NMR data, see Table 8; ESIMS *m/z* 726 [M + Na]^+^; HRESIMS *m/z* 726.4201 [M + Na]^+^ (calcd for C_39_H_61_NNaO_10_, 726.4193).

Glinusopposide R (**22**). White powder; ${\left[\alpha \right]}_{D}^{25}$ +10 (*c* 0.15, MeOH); ECD (*c* 0.078, MeOH) *λ*_max_ (Δ*ε*) 221 (–1.44) nm; ^1^H and ^13^C-NMR data, see Table 8; ESIMS *m/z* 859 [M + Na]^+^; HRESIMS *m/z* 859.4452 [M + Na]^+^ (calcd for C_44_H_68_NaO_15_, 859.4450).

Glinusopposide S (**23**). White powder; ${\left[\alpha \right]}_{D}^{25}$ +8 (*c* 0.1, MeOH); ^1^H and ^13^C-NMR data, see Table 8; ESIMS *m/z* 873 [M + Na]^+^; HRESIMS *m/z* 873.4606 [M + Na]^+^ (calcd for C_45_H_70_NaO_15_, 873.4607).

Glinusopposide T (**24**). White powder; ${\left[\alpha \right]}_{D}^{24}$ +71 (*c* 0.22, MeOH); ^1^H and ^13^C-NMR data, see Table 9; ESIMS *m/z* 655 [M + Na]^+^; HRESIMS *m/z* 655.3820 [M + Na]^+^ (calcd for C_36_H_56_NaO_9_, 655.3822).

Glinusopposide U (**25**). White powder; ${\left[\alpha \right]}_{D}^{25}$ +11 (*c* 0.1, MeOH); ^1^H and ^13^C-NMR data, see Table 9; ESIMS *m/z* 801 [M + Na]^+^; HRESIMS *m/z* 801.4393 [M + Na]^+^ (calcd for C_42_H_66_NaO_13_, 801.4396).

The 3β-*O*-(6-*O*-Methyl-β-d-glucuronopyranosyl)-olean-12-ene-28,30-dioic acid 30-methyl ester (**26**). White powder; ${\left[\alpha \right]}_{D}^{25}$ +66 (*c* 0.12, MeOH); ^1^H and ^13^C-NMR data, see Table 9; ESIMS *m/z* 713 [M + Na]^+^; HRESIMS *m/z* 713.3871 [M + Na]^+^ (calcd for C_38_H_58_NaO_11_, 713.3877).

### 3.5. Acid Hydrolysis and Sugar Analysis

#### 3.5.1. Acid Hydrolysis of **31** and Acetylation of Xylose

Compound **31** (15.5 mg) was dissolved in 2 M HCl (1 mL) and stirred at 90 °C for 4 h. After cooling, the solution was evaporated to dryness under reduced pressure. The reaction mixture was purified by silica gel column chromatography (CH_2_Cl_2_-MeOH-H_2_O, 500:10:1, 300:10:1, 200:10:1) to afford xylose (1.9 mg). The sugar was dissolved in pyridine (0.1 mL) and acetic anhydride (0.1 mL) and stirred for 21 h at room temperature. Then, water (5 mL) was added to the reaction mixture, followed by extraction with EtOAc (5 mL). The organic layer was dried under reduced pressure to yield 1,2,3,4-tetra-*O*-acetyl-d-xylopyranose (0.9 mg), which was identified based on its ^1^H-NMR spectrum and optical rotation value: ${\left[\alpha \right]}_{D}^{21}$ −31 (*c* 0.08, CHCl_3_) [21].

#### 3.5.2. Acid Hydrolysis of the Saponin Mixture and Acetylation of Rhamnose

The *n*-butanol-soluble part (20.0 g) was subjected to D101 resin column chromatography, eluted using water (discarded) and 60% EtOH to yield the saponin mixture (4.0 g). The latter (1.0 g) was dissolved in 2 M HCl (3 mL) and stirred at 90 °C for 5 h. The reaction mixture was dried and purified by silica gel column chromatography (CH_2_Cl_2_-MeOH-H_2_O, 500:10:1, 200:10:1, 100:10:1) to yield rhamnose (97.9 mg) and glucose (9.4 mg). The glucose was identified as d-glucose based on its ^1^H-NMR spectrum and optical rotation value: ${\left[\alpha \right]}_{D}^{19}$ +40 (*c* 0.22, H_2_O) [22]. The rhamnose (97.9 mg) was dissolved in pyridine (0.1 mL) and acetic anhydride (0.1 mL) and stirred for 21 h at room temperature. Then, water (5 mL) was added to the reaction mixture, followed by extraction with EtOAc (5 mL). The organic layer was dried under reduced pressure and purified by silica gel column chromatography (petroleum ether-EtOAc, 50:1) to yield 1,2,3,4-tetra-*O*-acetyl-α-l-rhamnopyranose (1.4 mg), which was identified based on its ^1^H-NMR spectrum and optical rotation value: ${\left[\alpha \right]}_{D}^{21}$ −27 (*c* 0.14, CHCl_3_) [23].

### 3.6. Antimicrobial Assays

The fungi strains *T. rubrum* ATCC 4438 and *M. gypseum* CBS118893) were purchased from the Institute of Dermatology and Hospital for Skin Diseases, Chinese Academy of Medical Sciences. An antifungal assay was performed according to modified versions of the clinical and laboratory standards institute (CLSI), formerly national committee for clinical laboratory standards (NCCLS) methods, as described previously [24,25]. Terbinafine hydrochloride was used as a positive control. The 50% minimum inhibitory concentration (MIC_50_) was calculated by the Reed-Muench method [26].

## 4. Conclusions

In this study, four new triterpenoids (**1**−**4**), 21 new triterpenoids glycosides (**5**−**25**), and 12 known compounds were isolated from *G. oppositifolius*. However, we cannot exclude the possibility that some of the isolated compounds might be artifacts resulted from the extraction treatment, for example compound **23** might be artifacts of ethanol extraction. The triterpenoids and their glycosides were hopane-type and oleanane-type which have been proofed to be existing in this plant [9,13]. Four compounds including glinusopposide B (**6**), glinusopposide Q (**21**), glinusopposide T (**24**), and glinusopposide U (**25**) showed considerable inhibitory activities against *M. gypseum* and *T. rubrum*. According to the study of SARs, sugars at 3-hydroxy, 30-carboxymethyl group, and the double bond at C-12 play a key role in oleanane type compounds for antifungal activities. The SARs of hopane type compounds for antifungal activities remain for further research. This study provides a scientific evidence of traditional practice on applying *G. oppositifolius* to treat dermatophytosis.

## Figures and Tables

**Table 1:** ^1^H (500 MHz) and ^13^C (126 MHz) NMR data of **1** and **2** in Pyridine-*d*_5_ (*δ* in ppm, *J* in Hz).

	1		2	
No.	*δ*_H_	*δ*_C_	*δ*_H_	*δ*_C_
1	1.68 m 0.97 m	39.1	1.79 m 1.31 m	39.4
2	1.81 m	28.3	2.48 m 2.42 m	34.4
3	3.44 br t (8.3)	78.0		216.4
4		39.5		47.4
5	0.78 dd (12.0, 1.7)	55.7	1.28 m	54.8
6	1.51 m 1.33 m	18.9	1.37 m 1.31 m	20.0
7	1.43 m 1.21 m	33.6	1.38 m 1.19 m	32.7
8		45.1		45.2
9	1.39 m	49.3	1.39 m	48.4
10		37.3		36.8
11	2.11 m 1.64 m	33.2	2.04 m 1.65 m	33.4
12	4.23 m	69.1	4.20 m	69.0
13	1.84 d (10.7)	56.4	1.84 d (10.8)	56.5
14		41.8		41.7
15	1.93 dd (12.7, 4.2) 1.71 m	46.0	1.92 dd (12.6, 4.3) 1.70 m	45.9
16	4.48 m	66.8	4.48 m	66.7
17	2.41 d (11.7)	73.8	2.41 d (11.6)	73.8
18		47.5		47.5
19	2.60 m 2.14 m	43.1	2.60 m 2.13 m	43.1
20	2.05 m 1.95 m	37.8	2.07 m 1.96 m	37.8
21		85.7		85.7
22		75.5		75.5
23	1.21 s	28.7	1.11 s	26.6
24	1.01 s	16.3	1.00 s	21.3
25	0.82 s	16.1	0.81 s	15.6
26	0.97 s	17.0	0.95 s	16.6
27	1.13 s	19.5	1.10 s	19.4
28	1.19 s	17.3	1.19 s	17.3
29	1.56 s	26.6	1.56 s	26.7
30	1.61 s	27.3	1.61 s	27.4
3-OH	5.78 br s			
12-OH	5.32 d (6.0)		5.38 d (6.9)	

**Table 2:** ^1^H and ^13^C-NMR data of **3** and **4** (*δ* in ppm, *J* in Hz).

	3 (Pyridine-*d*_5_)		4 (Methanol-*d*_4_)		4 (Pyridine-*d*_5_)	
No.	*δ*_H_ (500 MHz)	*δ*_C_ (126 MHz)	*δ*_H_ (600 MHz)	*δ*_C_ (151 MHz)	*δ*_H_ (500 MHz)	*δ*_C_ (126 MHz)
1	1.65 m 1.30 m	39.1	1.92 m 1.05 m	39.4	1.88 m 1.07 m	38.5
2	2.51 m 2.37 m	34.4	1.69 m 1.64 m	27.9	1.94 m 1.89 m	28.1
3		216.3	3.18 dd (11.7, 4.9)	79.8	3.49 dd (10.6, 5.0)	78.1
4		47.5		40.1		39.6
5	1.32 m	55.4	0.84 br d (12.1)	56.4	0.91 dd (12.2, 1.8)	55.3
6	1.35 m	19.8	1.63 m 1.46 m	19.6	1.61 m 1.44 m	18.8
7	1.46 m 1.31 m	32.7	1.35 m	33.7	1.31 m	32.9
8		39.7		42.2		41.2
9	1.70 m	47.2	1.99 br s	56.0	2.06 br s	54.9
10		36.9		38.0		37.1
11	1.88 m	23.8	5.72 br d (11.2)	129.0	5.78 br d (10.5)	127.8
12	5.72 br t (3.3)	122.8	6.33 dd (11.2, 2.8)	126.2	6.63 dd (10.5, 2.6)	125.7
13		144.8		139.8		138.3 ^a^
14		42.2		43.6		42.7
15	2.18 m	28.5	1.72 m 1.08 m	26.2	1.97 m 1.08 m	25.6
16α 16β	2.19 m 2.08 m	24.0	1.71 m 1.95 m	33.8	1.78 m 2.25 m	33.1
17		46.4		49.3 ^a^		48.8
18	3.63 dd (13.7, 4.0)	43.5		130.8		131.2 ^a^
19α 19β	1.92 dd (13.7, 13.7) 2.50 m	43.1	2.85 dd (14.5, 1.7) 2.17 d (14.5)	35.9	3.18 d (15.2) 2.78 d (15.2)	35.6
20		44.1		44.8		43.9
21	2.41 m 1.47 m	31.2	1.83 m 1.57 m	33.0	2.28 m 1.78 m	32.5
22	2.41 m 2.08 m	34.8	2.29 m 1.41 m	35.4	2.67 ddd (13.8, 3.5, 3.5) 1.52 m	35.0
23	1.14 s	26.6	0.98 s	28.6	1.24 s	28.6
24	0.99 s	21.6	0.78 s	15.9	1.03 s	16.1
25	0.86 s	14.9	0.94 s	18.8	0.97 s	18.4
26	1.00 s	17.3	0.81 s	17.3	1.07 s	17.0
27	1.30 s	26.1	1.00 s	20.3	1.09 s	20.1
28		180.1		179.9		DAS ^b^
29	1.43 s	29.1	1.11 s	20.4	1.28 s	20.2
30		179.5		180.4		178.5
30-OMe			3.67 s	52.6	3.60 s	51.8

^a^ Detected by HMBC. ^b^ Disappeared signal.

**Table 3:** ^1^H and ^13^C-NMR data of **5–7** in Pyridine-*d*_5_ (*δ* in ppm, *J* in Hz).

	5		6		7	
No.	*δ*_H_ (600 MHz)	*δ*_C_ (151 MHz)	*δ*_H_ (500 MHz)	*δ*_C_ (126 MHz)	*δ*_H_ (600 MHz)	*δ*_C_ (151 MHz)
1	1.70 m 0.97 m	39.3	1.63 m 0.90 m	39.1	1.67 m 0.94 m	39.3
2	2.21 m 1.91 m	27.4	2.13 m 1.88 m	27.0	2.14 m 1.87 m	27.3
3	3.38 dd (11.7, 4.4)	89.1	3.29 dd (11.8, 4.2)	88.6	3.31 dd (11.9, 4.4)	89.3
4		40.1		39.7		40.1
5	0.79 br d (11.9)	56.2	0.71 br d (9.3)	56.0	0.76 br d (12.0)	56.2
6	1.51 m 1.33 m	19.1	1.46 m 1.33 m	18.6	1.50 m 1.32 m	19.0
7	1.39 m 1.19 m	34.0	1.33 m 1.15 m	33.5	1.40 m 1.19 m	34.0
8		43.8		43.4		43.8
9	1.40 m	50.0	1.35 m	49.6	1.40 m	50.0
10		37.4		37.0		37.4
11	2.12 m 1.64 m	33.5	2.07 m 1.60 m	33.0	2.10 m 1.63 m	33.5
12	4.20 m	69.2	4.17 m	68.7	4.20 m	69.1
13	1.72 d (10.7)	56.9	1.68 d (11.1)	56.5	1.72 overlapped	57.0
14		42.1		41.6		42.1
15	1.51 m 1.20 m	34.8	1.48 m 1.17 m	34.4	1.52 m 1.21 m	34.8
16	1.44 m	20.2	1.42 m	19.8	1.46 m	20.2
17	1.82 dd (12.1, 2.9)	56.3	1.78 dd (11.9, 2.9)	55.9	1.82 dd (12.0, 2.8)	56.3
18		45.8		45.4		45.8
19	2.58 m 1.68 m	45.2	2.55 m 1.65 m	44.8	2.58 m 1.68 m	45.2
20	2.17 m 1.68 m	36.2	2.14 m 1.65 m	35.7	2.17 m 1.68 m	36.2
21		54.5		54.0		54.5
22		213.0		212.6		213.0
23	1.33 s	28.6	1.25 s	28.0	1.27 s	28.5
24	1.01 s	17.2	1.17 s	16.9	0.97 s	17.2
25	0.82 s	16.5	0.79 s	16.2	0.81 s	16.5
26	0.99 s	17.5	0.95 s	17.0	0.99 s	17.5
27	1.05 s	18.4	1.01 s	18.0	1.05 s	18.4
28	1.12 s	17.2	1.09 s	16.8	1.12 s	17.2
29	1.22 s	21.5	1.19 s	21.0	1.22 s	21.5
30	2.19 s	25.9	2.16 s	25.4	2.19 s	25.9
1′	4.87 d (7.6)	108.2	4.83 d (7.3)	106.2	4.77 d (7.5)	107.8
2′	4.05 dd (8.6, 7.6)	76.0	4.24 dd (8.2, 7.3)	78.0	4.04 dd (8.6, 7.5)	75.9
3′	4.20 m	79.1	4.18 m	79.7	4.32 dd (8.8, 8.8)	83.5
4′	4.26 m	71.7	4.15 m	71.6	4.17 m	70.2
5′	4.41 dd (11.3, 5.2) 3.80 dd (11.3, 10.5)	67.6	4.33 m 3.71 dd (10.5, 9.9)	67.0	4.36 m 3.74 dd (11.2, 10.3)	67.4
1″			6.54 d (0.9)	102.0	6.30 br s	103.2
2″			4.88 m	72.5	4.82 dd (3.4, 1.3)	73.1
3″			4.69 dd (9.5, 3.1)	72.6	4.62 dd (9.3, 3.4)	73.2
4″			4.35 m	74.2	4.37 dd (9.3, 9.3)	74.6
5″			4.77 m	69.8	5.01 m	70.4
6″			1.70 d (6.2)	18.8	1.71 d (6.2)	19.1
12-OH			5.23 d (6.2)			
2″-OH			6.69 br s			
4″-OH			6.72 br s			

**Table 4:** ^1^H and ^13^C-NMR data of **8**–**11** in Pyridine-*d*_5_ (*δ* in ppm, *J* in Hz).

	8		9		10		11	
No.	*δ*_H_ (500 MHz)	*δ*_C_ (126 MHz)	*δ*_H_ (500 MHz)	*δ*_C_ (126 MHz)	*δ*_H_ 800 MHz	*δ*_C_ (201 MHz)	*δ*_H_ (800 MHz)	*δ*_C_ (201 MHz)
1	1.62 m 0.85 m	38.8	1.63 m 0.86 m	38.8	1.41 m 0.66 m	38.6	1.64 m 0.87 m	38.8
2	2.03 m 1.80 m	26.8	2.04 m 1.81 m	26.8	1.98 m 1.76 m	26.6	2.05 m 1.82 m	26.8
3	3.25 dd (11.8, 4.4)	88.8	3.26 dd (11.8, 4.4)	88.8	3.22 dd (11.9, 4.6)	88.8	3.29 dd (11.9, 4.0)	88.8
4		39.6		39.6		39.5		39.6
5	0.69 d (11.7)	55.7	0.69 br d (11.8)	55.7	0.64 m	55.5	0.70 br d (12.5)	55.7
6	1.43 m 1.27 m	18.6	1.44 m 1.27 m	18.6	1.41 m 1.23 m	18.4	1.41 m 1.31 m	18.6
7	1.40 m 1.21 m	33.6	1.41 m 1.22 m	33.6	1.35 m 1.17 m	33.3	1.42 m 1.23 m	33.6
8		45.7		45.7		45.8		45.7
9	1.33 m	49.1	1.34 m	49.1	1.28 m	48.5	1.35 m	49.1
10		36.9		36.9		36.8		36.9
11	2.07 m 1.62 m	33.0	2.09 m 1.63 m	33.0	1.98 m 1.37 m	28.1	2.09 m 1.63 m	33.0
12	4.19 m	68.6	4.21 m	68.6	5.47 m	72.2	4.20 m	68.6
13	1.79 d (10.7)	55.8	1.79 d (10.5)	55.8	1.90 d (11.5)	52.4	1.79 d (10.6)	55.8
14		41.8		41.8		41.6		41.7
15	1.87 m 1.69 m	45.7	1.88 m 1.70 m	45.7	1.83 m 1.64 m	45.2	1.88 m 1.70 m	45.6
16	4.12 m	65.5	4.11 m	65.5	4.06 m	65.0	4.12 m	65.5
17	2.28 d (11.3)	63.7	2.28 d (11.4)	63.7	2.23 d (11.5)	63.3	2.28 d (11.7)	63.7
18		47.1		47.1		46.4		47.1
19	2.61 m 1.87 m	45.8	2.63 m 1.90 m	45.8	1.83 m 1.68 m	44.8	2.62 m 1.89 m	45.7
20	2.05 m 1.74 m	37.6	2.06 m 1.74 m	37.6	2.04 m 1.66 m	37.5	2.06 m 1.73 m	37.6
21		53.6		53.6		53.4		53.6
22		214.9		215.0		214.6		214.9
23	1.22 s	28.0	1.22 s	28.0	1.21 s	27.9	1.25 s	28.0
24	0.89 s	16.6	0.90 s	16.6	0.88 s	16.6	0.94 s	16.7
25	0.76 s	16.0	0.76 s	16.0	0.72 s	15.9	0.77 s	16.0
26	0.98 s	17.0	0.99 s	17.0	0.93 s	16.8	0.99 s	17.0
27	1.11 s	19.2	1.11 s	19.2	1.08 s	18.9	1.12 s	19.1
28	1.21 s	17.8	1.21 s	17.8	1.03 s	17.7	1.21 s	17.8
29	1.66 s	21.0	1.66 s	21.0	1.61 s	20.9	1.66 s	21.0
30	2.34 s	26.3	2.34 s	26.3	2.37 s	26.3	2.35 s	26.3
1′	4.74 d (7.7)	107.2	4.76 d (7.5)	107.2	4.75 d (7.5)	107.2	4.83 d (7.3)	106.9
2′	4.00 m	75.9	4.00 dd (8.3, 7.5)	75.8	4.01 dd (8.4, 7.5)	75.8	4.08 m	75.0
3′	4.40 m	78.3	4.43 m	79.0	4.43 m	79.0	4.35 m	83.9
4′	5.28 ddd (9.7, 9.7, 5.5)	71.3	5.38 overlapped	71.0	5.39 m	70.9	5.44 m	70.7
5′	4.30 m 3.58 dd (11.3, 11.0)	63.1	4.35 m 3.63 dd (11.3 10.0)	63.2	4.38 m 3.65 m	63.2	4.35 m 3.68 m	63.3
1″	6.26 d (1.2)	102.6	6.24 br s	102.8	6.25 br s	102.9	5.25 d (7.8)	106.9
2″	4.69 br s	72.4	4.74 dd (3.2, 1.4)	72.5	4.72 m	72.5	3.99 t (7.8)	75.8
3″	4.45 m	72.7	4.45 m	72.6	4.44 m	72.6	4.14 m	78.4
4″	4.30 t (9.3)	73.9	4.30 t (9.4)	73.9	4.29 dd (9.5, 9.5)	74.0	4.15 m	71.0
5″	4.43 m	70.0	4.40 m	70.0	4.40 m	70.1	4.31 m 3.70 m	67.6
6″	1.70 d (6.2)	18.8	1.68 d (6.3)	18.9	1.69 d (6.2)	18.9		
1′′′		170.4		165.9		165.9		165.9
2′′′	2.15 s	21.1	6.02 dq (15.6, 1.6)	122.7	6.04 br d (14.6)	122.8	5.99 br d (15.5)	123.1
3′′′			7.06 m	146.0	7.07 m	145.9	7.09 m	145.4
4′′′			1.66 d (6.8)	17.8	1.61 dd (7.0, 1.5)	17.8	1.61 br d (6.9)	17.8
1′′′′						170.4		
2′′′′					2.15 s	21.9		
12-OH	5.34 d (6.1)							
16-OH	5.49 d (5.0)							
2′-OH	7.68 d (6.1)							
2″-OH	6.80 br s							
4″-OH	6.80 br s							

**Table 5:** ^1^H and ^13^C-NMR data of **12**–**14** in Pyridine-*d*_5_ (*δ* in ppm, *J* in Hz).

	12		13		14	
No.	*δ*_H_ (500 MHz)	*δ*_C_ (126 MHz)	*δ*_H_ (600 MHz)	*δ*_C_ (151 MHz)	*δ*_H_ (600 MHz)	*δ*_C_ (151 MHz)
1	1.61 m 0.84 m	39.0	1.72 m 0.98 m	39.5	1.68 m 0.95 m	39.7
2	1.78 m	28.3	2.21 m 1.90 m	27.4	2.17 m 1.92 m	27.5
3	3.41 m	78.1	3.37 dd (11.8, 4.5)	89.0	3.31 dd (11.9, 4.2)	88.9
4		39.5		40.1		40.1
5	0.69 m	56.0	0.75 br d (10.8)	56.3	0.71 br d (11.5)	56.6
6	1.49 m 1.28 m	18.8	1.51 m 1.33 m	19.0	1.47 m 1.31 m	19.0
7	1.27 m	34.2	1.32 m	34.6	1.29 m	34.6
8		46.8		47.2		47.2
9	1.22 m	48.4	1.40 m	49.5	1.37 m	49.5
10		37.6		37.6		37.5
11	2.37 m 1.48 m	27.2	2.09 m 1.66 m	33.5	2.07 m 1.65 m	33.5
12	4.37 m	74.5	4.04 m	69.7	4.04 m	69.7
13	1.87 d (11.2)	54.0	1.92 d (11.1)	56.2	1.91 d (11.1)	56.2
14		41.5		41.9		41.9
15	1.92 m 1.29 m	43.0	1.99 dd (11.6. 6.2) 1.38 m	43.7	1.97 dd (11.7, 6.1) 1.36 m	43.7
16	4.84 m	76.0	4.93 m	76.6	4.92 m	76.6
17		152.5		153.0		153.0
18		46.0		46.4		46.4
19	2.88 m 2.54 m	53.7	3.15 m 2.70 m	54.6	3.15 m 2.70 ddd (13.6, 8.2, 2.6)	54.5
20	2.37 m 2.04 m	25.6	2.50 m 2.20 m	26.1	2.50 m 2.21 m	26.1
21		146.8		147.5		147.5
22		84.2		84.5		84.5
23	1.20 s	28.7	1.31 s	28.5	1.25 s	28.3
24	0.99 s	16.4	0.99 s	17.2	1.19 s	17.4
25	0.79 s	16.6	0.85 s	17.0	0.85 s	17.1
26	0.88 s	16.5	1.02 s	16.9	1.01 s	16.9
27	0.94 s	15.9	1.07 s	16.2	1.06 s	16.2
28	1.67 s	22.2	1.59 s	23.0	1.59 s	23.0
29	1.44 s	29.2	1.49 s	29.2	1.49 s	29.2
30	1.44 s	28.6	1.47 s	29.5	1.47 s	29.5
1′	5.19 d (7.5)	100.1	4.86 d (7.5)	108.2	4.85 d (7.4)	106.6
2′	4.07 m	75.9	4.05 m	76.0	4.27 dd (8.3, 7.4)	78.4
3′	4.37 m	78.8	4.20 dd (8.7, 8.7)	79.1	4.20 dd (8.7, 8.3)	80.1
4′	4.26 dd (9.5, 9.5)	72.4	4.26 m	71.7	4.17 m	72.0
5′	4.05 m	78.4	4.39 dd (11.3, 5.2) 3.79 dd (11.3, 10.7)	67.6	4.34 dd (11.4, 4.8) 3.73 dd (11.4, 9.9)	67.4
6′	4.57 dd (11.7, 2.2) 4.36 m	63.4				
1″					6.58 br s	102.4
2″					4.90 dd (3.4, 1.4)	72.9
3″					4.71 dd (9.4, 3.4)	73.0
4″					4.38 dd (9.3, 9.3)	74.6
5″					4.80 m	70.2
6″					1.73 d (6.2)	19.2
12-OH					5.46 br s	

**Table 6:** ^1^H and ^13^C-NMR data of **15**–**17** in Pyridine-*d*_5_ (*δ* in ppm, *J* in Hz).

	15		16		17	
No.	*δ*_H_ (600 MHz)	*δ*_C_ (151 MHz)	*δ*_H_ (500 MHz)	*δ*_C_ (126 MHz)	*δ*_H_ (800 MHz)	*δ*_C_ (201 MHz)
1	1.69 m 0.95 m	39.5	1.64 m 0.90 m	38.8	1.63 m 0.91 m	39.0
2	2.14 m 1.87 m	27.3	2.11 m 1.83 m	26.8	2.10 m 1.83 m	26.9
3	3.30 dd (11.9, 4.3)	89.2	3.27 dd (11.8, 4.4)	88.8	3.26 dd (12.0, 4.3)	88.8
4		40.1		39.6		39.6
5	0.73 br d (11.3)	56.3	0.72 br d (11.9)	55.7	0.73 br d (11.7)	55.9
6	1.48 m 1.31 m	19.0	1.47 m 1.30 m	18.6	1.44 m 1.30 m	18.6
7	1.31 m	34.6	1.45 m 1.27 m	33.5	1.40 m 1.31 m	33.6
8		47.2		45.4		44.8
9	1.39 m	49.5	1.38 m	49.2	1.47 m	49.6
10		37.5		36.9		37.0
11	2.09 m 1.65 m	33.5	2.07 m 1.61 m	33.1	2.08 m 1.62 m	33.4
12	4.04 m	69.7	4.19 m	69.3	4.08 m	69.6
13	1.92 d (11.1)	56.2	1.77 d (10.9)	53.9	1.91 m	55.1
14		47.2		41.8		41.7
15	2.00 m 1.38 m	43.7	1.87 dd (12.5, 4.2) 1.73 m	45.1	1.92 m 1.87 m	44.1
16	4.93 m	76.6	4.32 m	67.2	4.99 overlapped	68.1
17		153.0	2.14 d (10.8)	62.2		143.9
18		46.4		45.4		52.6
19	3.15 m 2.70 m	54.6	2.53 m 1.78 m	43.2	2.58 m 2.20 m	46.5
20	2.50 m 2.21 m	26.1	2.42 m	29.9	2.54 m 2.18 m	38.1
21		147.5		152.2		128.5
22		84.5	6.06 t (2.0) 5.14 t (2.0)	106.1	2.33 s	16.0
23	1.25 s	28.4	1.23 s	28.0	1.21 s	28.0
24	0.95 s	17.2	0.93 s	16.7	0.92 s	16.7
25	0.83 s	17.0	0.78 s	16.0	0.79 s	16.4
26	1.02 s	16.9	1.01 s	17.0	0.98 s	16.5
27	1.07 s	16.2	1.16 s	19.1	1.25 s	17.5
28	1.59 s	23.0	1.06 s	16.0	1.39 s	20.6
29	1.49 s	29.2				
30	1.47 s	29.5				
1′	4.76 d (7.5)	107.8	4.74 d (7.4)	107.4	4.73 d (7.5)	107.4
2′	4.03 m	75.9	4.01 m	75.4	4.00 m	75.4
3′	4.32 dd (8.9, 8.9)	83.5	4.30 t (8.9)	83.0	4.29 t (8.8)	83.0
4′	4.16 m	70.2	4.14 m	69.7	4.13 m	69.7
5′	4.35 m 3.73 dd (11.2, 10.4)	67.4	4.32 m 3.72 dd (11.3, 10.2)	67.0	4.33 m 3.71 dd (11.2, 10.3)	66.9
1″	6.30 br s	103.3	6.26 d (1.2)	102.9	6.26 br s	102.8
2″	4.82 dd (3.3, 1.5)	73.1	4.78 br s	72.6	4.78 br s	72.6
3″	4.62 dd (9.4, 3.3)	73.2	4.60 br d (9.1)	72.8	4.59 br d (8.7)	72.8
4″	4.37 dd (9.4, 9.4)	74.6	4.34 m	74.2	4.33 m	74.2
5″	5.00 m	70.4	4.98 overlapped	70.0	4.97 overlapped	70.0
6″	1.71 d (6.2)	19.2	1.68 d (6.2)	18.7	1.68 d (6.1)	18.7
12-OH			5.27 d (6.5)		5.20 d (6.2)	
16-OH			5.72 d (5.9)		6.03 d (5.5)	
2′-OH			7.29 d (6.0)		7.26 d (6.0)	
4′-OH			6.79 d (5.9)		6.76 d (5.6)	
2″-OH			6.74 br s		6.71 br s	
3″-OH			6.47 br s		6.43 br s	
4″-OH			6.74 br s		6.71 br s	

**Table 7:** ^1^H and ^13^C-NMR data of **18**–**20** in Pyridine-*d*_5_ (*δ* in ppm, *J* in Hz).

	18		19		20	
No.	*δ*_H_ (600 MHz)	*δ*_C_ (151 MHz)	*δ*_H_ (500 MHz)	*δ*_C_ (126 MHz)	*δ*_H_ (*J* in Hz) (600 MHz)	*δ*_C_ (151 MHz)
1	1.69 m 0.98 m	39.2	1.61 m 0.90 m	38.8	1.70 m 0.89 m	38.5
2	2.21 m 1.91 m	27.4	2.10 m 1.82 m	26.9	2.14 m 1.87 m	27.0
3	3.39 dd (11.9, 4.3)	89.1	3.27 dd (11.9, 4.4)	88.8	3.32 dd (11.9, 4.5)	89.8
4		40.2		39.7		40.1
5	0.84 m	56.5	0.77 overlapped	56.0	0.79 br d (12.1)	55.6
6	1.57 m 1.42 m	19.1	1.54 m 1.37 m	18.6	1.52 m 1.33 m	18.8
7	1.52 m	33.9	1.48 m	33.5	1.28 m	33.2
8		41.8		41.3		41.6
9	1.50 m	50.2	1.45 m	49.7	1.98 br s	55.2
10		37.7		37.3		37.1
11	2.18 m 1.66 m	34.3	2.14 m 1.61 m	33.9	5.69 br d (10.1)	128.2
12	4.33 m	69.4	4.28 m	69.0	6.60 dd (10.1, 2.9)	126.2
13	2.17 d (11.1)	53.3	2.13 d (11.1)	52.8		138.7
14		47.5		47.0		43.1
15	5.76 d (10.3)	135.5	5.72 d (10.3)	135.1	1.96 m 1.09 m	26.0
16	6.42 d (10.3)	120.4	6.39 d (10.3)	120.0	2.26 m 1.78 m	33.5
17		141.9		141.5		49.2
18		48.6		48.2		131.4
19	2.59 m 2.24 m	45.3	2.55 m 2.21 m	44.9	3.17 d (14.5) 2.77 d (14.5)	36.0
20	2.60 m 2.14 m	36.8	2.55 m 2.09 m	36.3		44.3
21		131.8		131.4	2.31 m 1.79 m	32.9
22	1.73 s	14.4	1.70 s	14.0	2.66 m 1.52 m	35.3
23	1.35 s	28.6	1.25 s	28.0	1.25 s	28.2
24	1.02 s	17.2	0.94 s	16.7	0.89 s	16.8
25	0.81 s	16.3	0.76 s	15.9	0.84 s	18.7
26	1.03 s	19.7	0.99 s	19.3	1.02 s	17.4
27	1.34 s	19.5	1.30 s	19.0	1.11 s	20.5
28	1.32 s	20.4	1.29 s	20.0		179.1
29					1.27 s	20.7
30						178.9
30-Me					3.60 s	52.2
1′	4.88 d (7.7)	108.2	4.74 d (7.5)	107.4	4.92 d (7.9)	107.6
2′	4.05 dd (8.4, 7.7)	76.0	4.00 dd (8.4, 7.5)	75.4	4.07 dd (8.7, 7.9)	76.2
3′	4.20 dd (8.8, 8.4)	79.1	4.28 m	83.0	4.45 dd (9.3, 8.7)	82.3
4′	4.26 m	71.7	4.12 m	69.7	4.41 dd (9.3, 9.3)	71.9
5′	4.40 dd (11.0, 5.1) 3.80 dd (11.0, 10.4)	67.6	4.32 m 3.70 dd (11.0, 10.5)	67.0	4.58 d (9.3)	77.6
6′						171.3
6′-OMe					3.79 s	52.7
1″			6.27 br s	102.8	6.33 br s	103.4
2″			4.79 br s	72.6	4.76 dd (3.3, 1.4)	73.0
3″			4.59 dd (9.3, 3.0)	72.8	4.57 dd (9.3, 3.3)	73.2
4″			4.34 dd (9.3, 9.3)	74.2	4.35 dd (9.3, 9.3)	74.6
5″			4.97 m	69.9	5.08 m	70.3
6″			1.67 d (6.1)	18.7	1.71 d (6.1)	19.1

**Table 8:** ^1^H and ^13^C-NMR data of **21**–**23** in Pyridine-*d*_5_ (*δ* in ppm, *J* in Hz).

	21		22		23	
No.	*δ*_H_ (500 MHz)	*δ*_C_ (126 MHz)	*δ*_H_ (500 MHz)	*δ*_C_ (126 MHz)	*δ*_H_ (500 MHz)	*δ*_C_ (126 MHz)
1	1.35 m 0.82 m	38.6	1.36 m 0.82 m	38.5	1.37 m 0.83 m	38.5
2	2.19 m 1.76 m	26.4	2.04 m 1.77 m	26.6	2.07 m 1.78 m	26.6
3	3.25 dd (11.9, 4.4)	89.2	3.28 overlapped	89.4	3.27 overlapped	89.3
4		39.3		39.5		39.5
5	0.75 overlapped	55.8	0.75 overlapped	55.7	0.74 overlapped	55.7
6	1.49 m 1.28 m	18.6	1.46 m 1.25 m	18.4	1.46 m 1.26 m	18.4
7	1.46 m 1.27 m	33.3	1.44 m 1.26 m	33.2	1.45 m 1.26 m	33.2
8		39.7		42.1		42.1
9	1.60 m	48.1	1.61 m	48.0	1.62 dd (8.6, 8.6)	48.0
10		37.0		36.9		36.9
11	1.85 m	23.8	1.85 m	23.7	1.85 m	23.7
12	5.58 br s	122.5	5.59 br s	123.1	5.59 dd (3.4, 3.4)	123.1
13		145.5		144.5		144.5
14		42.2		39.7		39.7
15	2.21 m	28.6	2.12 m 1.18 m	28.4	2.13 m 1.27 m	28.4
16	2.08 m	24.2	2.12 m 2.02 m	23.9	2.13 m 2.03 m	23.9
17		46.4		46.2		46.2
18	3.36 dd (13.5, 3.6)	43.6	3.29 overlapped	43.4	3.29 overlapped	43.4
19	2.26 m 1.82 m	43.2	2.25 br d (13.2) 1.81 m	42.7	2.25 br d (12.0) 1.82 m	42.7
20		44.3		44.2		44.2
21	2.16 m 1.45 m	31.2	2.19 m 1.46 m	30.9	2.19 br d (13.5) 1.47 m	30.9
22	2.06 m 1.96 m	34.9	2.08 m 1.97 m	34.6	2.09 m 1.98, m	34.6
23	1.19 s	28.2	1.24 s	28.1	1.23 s	28.1
24	0.98 s	17.0	0.90 s	16.9	0.90 s	16.9
25	0.75 s	15.4	0.75 s	15.4	0.76 s	15.4
26	0.89 br s	17.6	0.95 s	17.3	0.95 s	17.3
27	1.31 s	26.2	1.31 s	26.2	1.31 s	26.2
28		DAP *^a^*		179.9		179.9
29	1.22 s	28.7	1.23 s	28.5	1.23 s	28.5
30		177.4		177.2		177.2
30-OMe	3.63 s	51.7	3.65 s	51.8	3.65 s	51.7
1′	5.05 d (8.2)	105.0	4.88 d (7.8)	107.1	4.88 d (7.9)	107.2
2′	4.58 m	58.1	4.34 m	74.1	4.05 dd (8.4, 7.9)	75.8
3′	4.41 dd (9.3, 8.7)	76.3	4.42 dd (9.0, 9.0)	81.9	4.44 dd (8.9, 8.4)	81.9
4′	4.18 dd (9.3, 9.3)	72.7	4.37 m	71.4	4.41 dd (9.4, 8.9)	71.4
5′	3.97 m	78.4	4.56 d (9.4)	77.2	4.55 d (9.4)	77.2
6′	4.57 m 4.37 m	63.0		170.8		170.3
6′-OMe,			3.77 s	52.2	4.29 m	61.4
OEt					1.19 t (7.1)	14.3
1″		170.3	6.30 br s	102.9	6.33 d (1.2)	102.8
2″	2.15 s	23.8	4.75 m	72.5	4.77 dd (3.4, 1.2)	72.6
3″			4.55 m	72.7	4.56 dd (9.2, 3.4)	72.7
4″			4.34 m	74.1	4.36 dd (9.2, 9.2)	74.1
5″			5.05 m	69.9	5.08 m	69.8
6″			1.69 d (6.1)	18.6	1.69 d (6.2)	18.7
NH	8.94 d (9.0)					

*^a^* Disappeared.

**Table 9:** ^1^H- and ^13^C-NMR data of **24**–**26** in Pyridine-*d*_5_ (*δ* in ppm, *J* in Hz).

	24		25		26	
No.	*δ*_H_ (500 MHz)	*δ*_C_ (126 MHz)	*δ*_H_ (500 MHz)	*δ*_C_ (126 MHz)	*δ*_H_ (500 MHz)	*δ*_C_ (126 MHz)
1	1.51 m 0.97 m	38.8	1.47 m 0.93 m	38.7	1.39 m 0.85 m	38.6
2	2.16 m 1.87 m	26.8	2.09 m 1.81 m	26.7	2.13 m 1.83 m	26.6
3	3.35 dd (11.7, 4.4)	88.7	3.28 overlapped	88.8	3.36 dd (11.6, 4.3)	89.2
4		39.6		39.5		39.6
5	0.82 overlapped	55.9	0.79 overlapped	55.8	0.79 overlapped	55.8
6	1.49 m 1.28 m	18.5	1.47 m 1.27 m	18.5	1.47 m 1.26 m	18.5
7	1.47 m 1.28 m	33.2	1.46 m 1.27 m	33.2	1.45 m 1.27 m	33.2
8		39.7		42.0		39.7
9	1.67 dd (9.0, 8.7)	48.1	1.65 m	48.0	1.63 dd (9.0, 8.6)	48.0
10		37.1		37.0		37.0
11	1.89 m	23.8	1.88 m	23.7	1.87 m	23.7
12	5.60 br t (3.1)	123.2	5.60 dd (3.4, 3.4)	123.1	5.60 br t (3.3)	123.1
13		144.5		144.4		144.5
14		42.1		39.7		42.1
15	2.13 m 1.19 m	28.4	2.13 m 1.27 m	28.4	2.13 m 1.20 m	28.4
16	2.13 m 2.03 m	23.9	2.12 m 2.02 m	23.8	2.12 m 2.03 m	23.9
17		46.2		46.2		46.2
18	3.30 dd (13.6, 3.6)	43.4	3.29 overlapped	43.4	3.29 dd (13.6, 3.9)	43.4
19	2.26 m 1.82 dd (13.6, 13.6)	42.7	2.26 br d (13.5) 1.82 dd (13.5, 13.5)	42.7	2.26 m 1.82 m	42.7
20		44.2		44.2		44.2
21	2.19 m 1.46 m	30.9	2.19 br d (13.1) 1.46 m	30.8	2.20 m 1.46 m	30.9
22	2.10 m 1.98 m	34.6	2.09 m 1.98 m	34.5	2.09 m 1.98 m	34.6
23	1.30 s	28.2	1.24 s	28.1	1.30 s	28.2
24	0.97 s	17.0	0.92 s	16.9	0.95 s	17.0
25	0.82 s	15.5	0.80 s	15.5	0.78 s	15.5
26	0.97 s	17.4	0.96 s	17.3	0.95 s	17.4
27	1.30 s	26.2	1.30 s	26.1	1.30 s	26.2
28		179.9		179.9		179.9
29	1.23 s	28.5	1.23 s	28.5	1.23 s	28.5
30		177.2		177.2		177.2
30-OMe	3.65 s	51.8	3.64 s	51.7	3.65 s	51.8
1′	4.83 d (7.6)	107.8	4.73 d (7.5)	107.4	5.00 d (7.8)	107.3
2′	4.01 dd (8.7, 7.6)	75.6	4.00 dd (8.8, 7.5)	75.4	4.09 dd (9.0, 7.8)	75.4
3′	4.17 dd (8.7, 8.7)	78.7	4.29 dd (8.8, 8.8)	82.9	4.28 dd (9.0, 9.0)	77.9
4′	4.23 m	71.3	4.13 m	69.7	4.48 dd (9.0, 9.7)	73.3
5′	4.38 dd (11.2, 5.1) 3.78 dd (11.2, 10.2)	67.2	4.34 m 3.73 dd (10.6, 10.2)	66.9	4.61 d (9.7)	77.3
6′						170.9
6′-OMe					3.73 s	52.1
1″			6.27 d (1.2)	102.7		
2″			4.79 dd (3.3, 1.2)	72.6		
3″			4.60 dd (9.3, 3.3)	72.7		
4″			4.34 dd (9.3, 9.3)	74.1		
5″			4.98 m	69.9		
6″			1.67 d (6.2)	18.7		

**Table 10:** Antifungal Effects of Compounds from *G. oppositifolius* against *Microsporum gypseum* and *Trichophyton rubrum* ^a^.

Compound	MIC_50_ (μM) ± SD	
	*M. gypseum*	*T. rubrum*
**1**	105.0 ± 0.6	>300
**3**	128.1 ± 1.4	>300
**6**	7.1 ± 1.2	14.3 ± 2.1
**12**	260.1 ± 2.3	>300
**15**	46.8 ± 0.1	>300
**16**	120.7 ± 1.4	>300
**18**	29.3 ± 3.4	>300
**19**	34.9 ± 1.2	>300
**21**	6.7 ± 2.1	13.4 ± 1.1
**22**	40.3 ± 0.5	>300
**23**	39.9 ± 1.2	>300
**24**	6.8 ± 3.2	11.9 ± 0.3
**25**	11.1 ± 2.4	13.0 ± 1.3
**26**	22.0 ± 0.9	>300
terbinafine hydrochloride (positive control)	0.008 ± 0.373	1.647 ± 0.101

^a^ Compounds **2**, **4**–**7**, **9**–**11**, **13**, **14**, **17**, **20**, and **27**–**37** were inactive (MIC_50_ > 300 μM).

## Supplementary Materials

The following are available online. General experimental procedures; Figures S1–S198: 1D and 2D-NMR and HRESIMS spectra of compounds **1**–**26**, structures of known compounds (**26**–**37**), and key 2D-NMR correlations of **2**, **6**–**11**, and **13**–**25**.
