# Supplementary material for: Triterpenoids and Their Glycosides from Glinus Oppositifolius with Antifungal Activities against Microsporum Gypseum and Trichophyton Rubrum
Source: Molecules. 2019 Jun 12;24(12):2206. doi: 10.3390/molecules24122206 (PMC6631934; doi:10.3390/molecules24122206)
Supplement: Supplementary file 1 [file molecules-24-02206-s001.pdf]

Supporting Information for

Triterpenoids and their glycosides from *Glinus oppositifolius* with antifungal activities against *Microsporum gypseum* and *Trichophyton rubrum*

Dong-Dong Zhang,[†,‡] Yao Fu,[†,‡] Jun Yang,[†,‡] Xiao-Nian Li,[‡] Myint Myint San,[§] Thaung Naing Oo,[§] Yue-Hu Wang*,[†,‡] and Xue-Fei Yang*,[†,‡]

[†] Southeast Asia Biodiversity Research Institute, Chinese Academy of Sciences, Yezin, Nay Pyi Taw 05282, Myanmar

[‡] Key Laboratory of Economic Plants and Biotechnology and Yunnan Key Laboratory for Wild Plant Resources, State Key Laboratory of Phytochemistry and Plant Resources in West China, Kunming Institute of Botany, Chinese Academy of Sciences, Kunming 650201, People's Republic of China

[§] Forest Research Institute, Yezin, Nay Pyi Taw 05282, Myanmar

* Correspondence: Corresponding Authors: wangyuehu@mail.kib.ac.cn (Y.-H.W.); xuefei@mail.kib.ac.cn (X.-F.Y.).

Corresponding Authors

*E-mail: wangyuehu@mail.kib.ac.cn (Y.-H.W.), xuefei@mail.kib.ac.cn (X.-F.Y.).

Contents

3.1. General Experimental Procedures
**Table S1.** ^{1}H and ^{13}C NMR Data of **26** in Methanol-*d*$_4$ (*δ* in ppm, *J* in Hz).
**Figure S1**. Structures of known compounds (**26–37**) from *Glinus oppositifolius*.
**Figure S2.** Key 2D NMR correlations of **2** and **6–10**.
**Figure S3.** Key 2D NMR correlations of **11** and **13–16**.
**Figure S4.** Key 2D NMR correlations of **17–25**.
**Figure S5.** ^{1}H NMR spectrum of **1** (pyridine-*d*$_5$, 500 MHz).
**Figure S6.** ^{13}C NMR spectrum of **1** (pyridine-*d*$_5$, 126 MHz).
**Figure S7.** HSQC spectrum of **1**.
**Figure S8.** ^{1}H–^{1}H COSY spectrum of **1**.
**Figure S9.** HMBC spectrum of **1**.
**Figure S10.** ROESY spectrum of **1**.
**Figure S11.** HRESIMS spectrum of **1**.
**Figure S12.** ^{1}H NMR spectrum of **2** (pyridine-*d*$_5$, 500 MHz).
**Figure S13.** ^{13}C NMR spectrum of **2** (pyridine-*d*$_5$, 126 MHz).
**Figure S14.** HSQC spectrum of **2**.
**Figure S15.** ^{1}H–^{1}H COSY spectrum of **2**.
**Figure S16.** HMBC spectrum of **2**.
**Figure S17.** ROESY spectrum of **2**.
**Figure S18.** HRESIMS spectrum of **2**.
**Figure S19.** ^{1}H NMR spectrum of **3** (pyridine-*d*$_5$, 500 MHz).
**Figure S20.** ^{13}C NMR spectrum of **3** (pyridine-*d*$_5$, 126 MHz).
**Figure S21.** HSQC spectrum of **3**.
**Figure S22.** ^{1}H–^{1}H COSY spectrum of **3**.
**Figure S23.** HMBC spectrum of **3.**
**Figure S24.** ROESY spectrum of **3**.
**Figure S25.** HRESIMS spectrum of **3**.
**Figure S26.** ^{1}H NMR spectrum of **4** (methanol-*d*$_4$, 600 MHz).
**Figure S27.** ^{13}C NMR spectrum of **4** (methanol-*d*$_4$, 151 MHz).
**Figure S28.** HSQC spectrum of **4** (methanol-*d*$_4$).
**Figure S29.** ^{1}H–^{1}H COSY spectrum of **4** (methanol-*d*$_4$).
**Figure S30.** HMBC spectrum of **4** (methanol-*d*$_4$).
**Figure S31.** ROESY spectrum of **4** (methanol-*d*$_4$).
**Figure S32.** HRESIMS spectrum of **4**.
**Figure S33.** ^{1}H NMR spectrum of **4** (pyridine-*d*$_5$, 500 MHz).
**Figure S34.** ^{13}C NMR spectrum of **4** (pyridine-*d*$_5$, 126 MHz).
**Figure S35.** HSQC spectrum of **4** (pyridine-*d*$_5$).
**Figure S36.** ^{1}H–^{1}H COSY spectrum of **4** (pyridine-*d*$_5$).
**Figure S37.** HMBC spectrum of **4** (pyridine-*d*$_5$).
**Figure S38.** ROESY spectrum of **4** (pyridine-*d*$_5$).
**Figure S39.** ^{1}H NMR spectrum of **5** (pyridine-*d*$_5$, 600 MHz).
**Figure S40.** ^{13}C NMR spectrum of **5** (pyridine-*d*$_5$, 151 MHz).
**Figure S41.** HSQC spectrum of **5**.
**Figure S42.** ^{1}H–^{1}H COSY spectrum of **5**.
**Figure S43.** HMBC spectrum of **5**.
**Figure S44.** ROESY spectrum of **5**.
**Figure S45.** HRESIMS spectrum of **5**.

**Figure S46.** ^{1}H NMR spectrum of **6** (pyridine-d_5, 500 MHz).
**Figure S47.** ^{13}C NMR spectrum of **6** (pyridine-d_5, 126 MHz).
**Figure S48.** HSQC spectrum of **6**.
**Figure S49.** ^{1}H–^{1}H COSY spectrum of **6**.
**Figure S50.** HMBC spectrum of **6**.
**Figure S51.** ROESY spectrum of **6**.
**Figure S52.** HRESIMS spectrum of **6**.
**Figure S53.** ^{1}H NMR spectrum of **7** (pyridine-d_5, 600 MHz).
**Figure S54.** ^{13}C NMR spectrum of **7** (pyridine-d_5, 151 MHz).
**Figure S55.** HSQC spectrum of **7**.
**Figure S56.** ^{1}H–^{1}H COSY spectrum of **7**.
**Figure S57.** HMBC spectrum of **7**.
**Figure S58.** ROESY spectrum of **7**.
**Figure S59.** HRESIMS spectrum of **7**.
**Figure S60.** ^{1}H NMR spectrum of **8** (pyridine-d_5, 500 MHz).
**Figure S61.** ^{13}C NMR spectrum of **8** (pyridine-d_5, 126 MHz).
**Figure S62.** HSQC spectrum of **8**.
**Figure S63.** ^{1}H–^{1}H COSY spectrum of **8**.
**Figure S64.** HMBC spectrum of **8**.
**Figure S65.** ROESY spectrum of **8**.
**Figure S66.** HRESIMS spectrum of **8**.
**Figure S67.** ^{1}H NMR spectrum of **9** (pyridine-d_5, 500 MHz).
**Figure S68.** ^{13}C NMR spectrum of **9** (pyridine-d_5, 126 MHz).
**Figure S69.** HSQC spectrum of **9**.
**Figure S70.** ^{1}H–^{1}H COSY spectrum of **9**.
**Figure S71.** HMBC spectrum of **9.**
**Figure S72.** ROESY spectrum of **9**.
**Figure S73.** HRESIMS spectrum of **9**.
**Figure S74.** ^{1}H NMR spectrum of **10** (pyridine-d_5, 800 MHz).
**Figure S75.** ^{13}C NMR spectrum of **10** (pyridine-d_5, 201 MHz).
**Figure S76.** HSQC spectrum of **10**.
**Figure S77.** ^{1}H–^{1}H COSY spectrum of **10**.
**Figure S78.** HMBC spectrum of **10**.
**Figure S79.** ROESY spectrum of **10**.
**Figure S80.** HRESIMS spectrum of **10**.
**Figure S81.** ^{1}H NMR spectrum of **11** (pyridine-d_5, 800 MHz).
**Figure S82.** ^{13}C NMR spectrum of **11** (pyridine-d_5, 201 MHz).
**Figure S83.** HSQC spectrum of **11**.
**Figure S84.** ^{1}H–^{1}H COSY spectrum of **11**.
**Figure S85.** HMBC spectrum of **11**.
**Figure S86.** ROESY spectrum of **11**.
**Figure S87.** HRESIMS spectrum of **11**.
**Figure S88.** ^{1}H NMR spectrum of **12** (pyridine-d_5, 500 MHz).
**Figure S89.** ^{13}C NMR spectrum of **12** (pyridine-d_5, 126 MHz).
**Figure S90.** HSQC spectrum of **12**.
**Figure S91.** ^{1}H–^{1}H COSY spectrum of **12**.
**Figure S92.** HMBC spectrum of **12**.
**Figure S93.** ROESY spectrum of **12**.
**Figure S94.** HRESIMS spectrum of **12**.

**Figure S95.** ^{1}H NMR spectrum of **13** (pyridine-d_5, 600 MHz).
**Figure S96.** ^{13}C NMR spectrum of **13** (pyridine-d_5, 151 MHz).
**Figure S97.** HSQC spectrum of **13**.
**Figure S98.** ^{1}H–^{1}H COSY spectrum of **13**.
**Figure S99.** HMBC spectrum of **13**.
**Figure S100.** ROESY spectrum of **13**.
**Figure S101.** HRESIMS spectrum of **13**.
**Figure S102.** ^{1}H NMR spectrum of **14** (pyridine-d_5, 600 MHz).
**Figure S103.** ^{13}C NMR spectrum of **14** (pyridine-d_5, 151 MHz).
**Figure S104.** HSQC spectrum of **14**.
**Figure S105.** ^{1}H–^{1}H COSY spectrum of **14**.
**Figure S106.** HMBC spectrum of **14**.
**Figure S107.** ROESY spectrum of **14**.
**Figure S108.** HRESIMS spectrum of **14**.
**Figure S109.** ^{1}H NMR spectrum of **15** (pyridine-d_5, 600 MHz).
**Figure S110.** ^{13}C NMR spectrum of **15** (pyridine-d_5, 151 MHz).
**Figure S111.** HSQC spectrum of **15**.
**Figure S112.** ^{1}H–^{1}H COSY spectrum of **15**.
**Figure S113.** HMBC spectrum of **15**.
**Figure S114.** ROESY spectrum of **15**.
**Figure S115.** HRESIMS spectrum of **15**.
**Figure S116.** ^{1}H NMR spectrum of **16** (pyridine-d_5, 500 MHz).
**Figure S117.** ^{13}C NMR spectrum of **16** (pyridine-d_5, 126 MHz).
**Figure S118.** HSQC spectrum of **16**.
**Figure S119.** ^{1}H–^{1}H COSY spectrum of **16**.
**Figure S120.** HMBC spectrum of **16**.
**Figure S121.** ROESY spectrum of **16**.
**Figure S122.** HRESIMS spectrum of **16**.
**Figure S123.** ^{1}H NMR spectrum of **17** (pyridine-d_5, 800 MHz).
**Figure S124.** ^{13}C NMR spectrum of **17** (pyridine-d_5, 201 MHz).
**Figure S125.** HSQC spectrum of **17**.
**Figure S126.** ^{1}H–^{1}H COSY spectrum of **17**.
**Figure S127.** HMBC spectrum of **17**.
**Figure S128.** ROESY spectrum of **17**.
**Figure S129.** HRESIMS spectrum of **17**.
**Figure S130.** ^{1}H NMR spectrum of **18** (pyridine-d_5, 600 MHz).
**Figure S131.** ^{13}C NMR spectrum of **18** (pyridine-d_5, 151 MHz).
**Figure S132.** HSQC spectrum of **18**.
**Figure S133.** ^{1}H–^{1}H COSY spectrum of **18**.
**Figure S134.** HMBC spectrum of **18**.
**Figure S135.** ROESY spectrum of **18**.
**Figure S136.** HRESIMS spectrum of **18**.
**Figure S137.** ^{1}H NMR spectrum of **19** (pyridine-d_5, 500 MHz).
**Figure S138.** ^{13}C NMR spectrum of **19** (pyridine-d_5, 126 MHz).
**Figure S139.** HSQC spectrum of **19**.
**Figure S140.** ^{1}H–^{1}H COSY spectrum of **19**.
**Figure S141.** HMBC spectrum of **19**.
**Figure S142.** ROESY spectrum of **19**.
**Figure S143.** HRESIMS spectrum of **19**.

**Figure S144.** ^{1}H NMR spectrum of **20** (pyridine-d_5, 600 MHz).
**Figure S145.** ^{13}C NMR spectrum of **20** (pyridine-d_5, 151 MHz).
**Figure S146.** HSQC spectrum of **20**.
**Figure S147.** ^{1}H–^{1}H COSY spectrum of **20**.
**Figure S148.** HMBC spectrum of **20**.
**Figure S149.** ROESY spectrum of **20**.
**Figure S150.** HRESIMS spectrum of **20**.
**Figure S151.** ^{1}H NMR spectrum of **21** (pyridine-d_5, 500 MHz).
**Figure S152.** ^{13}C NMR spectrum of **21** (pyridine-d_5, 126 MHz).
**Figure S153.** HSQC spectrum of **21**.
**Figure S154.** ^{1}H–^{1}H COSY spectrum of **21**.
**Figure S155.** HMBC spectrum of **21**.
**Figure S156.** ROESY spectrum of **21**.
**Figure S157.** HRESIMS spectrum of **21**.
**Figure S158.** ^{1}H NMR spectrum of **22** (pyridine-d_5, 500 MHz).
**Figure S159.** ^{13}C NMR spectrum of **22** (pyridine-d_5, 126 MHz).
**Figure S160.** HSQC spectrum of **22**.
**Figure S161.** ^{1}H–^{1}H COSY spectrum of **22**.
**Figure S162.** HMBC spectrum of **22**.
**Figure S163.** ROESY spectrum of **22**.
**Figure S164.** HRESIMS spectrum of **22**.
**Figure S165.** ^{1}H NMR spectrum of **23** (pyridine-d_5, 500 MHz).
**Figure S166.** ^{13}C NMR spectrum of **23** (pyridine-d_5, 126 MHz).
**Figure S167.** HSQC spectrum of **23**.
**Figure S168.** ^{1}H–^{1}H COSY spectrum of **23**.
**Figure S169.** HMBC spectrum of **23**.
**Figure S170.** ROESY spectrum of **23**.
**Figure S171.** HRESIMS spectrum of **23**.
**Figure S172.** ^{1}H NMR spectrum of **24** (pyridine-d_5, 500 MHz).
**Figure S173.** ^{13}C NMR spectrum of **24** (pyridine-d_5, 126 MHz).
**Figure S174.** HSQC spectrum of **24**.
**Figure S175.** ^{1}H–^{1}H COSY spectrum of **24**.
**Figure S176.** HMBC spectrum of **24**.
**Figure S177.** ROESY spectrum of **24**.
**Figure S178.** HRESIMS spectrum of **24**.
**Figure S179.** ^{1}H NMR spectrum of **25** (pyridine-d_5, 500 MHz).
**Figure S180.** ^{13}C NMR spectrum of **25** (pyridine-d_5, 126 MHz).
**Figure S181.** HSQC spectrum of **25**.
**Figure S182.** ^{1}H–^{1}H COSY spectrum of **25**.
**Figure S183.** HMBC spectrum of **25**.
**Figure S184.** ROESY spectrum of **25**.
**Figure S185.** HRESIMS spectrum of **25**.
**Figure S186.** ^{1}H NMR spectrum of **26** (pyridine-d_5, 500 MHz).
**Figure S187.** ^{13}C NMR spectrum of **26** (pyridine-d_5, 126 MHz).
**Figure S188.** HSQC spectrum of **26** (pyridine-d_5).
**Figure S189.** ^{1}H–^{1}H COSY spectrum of **26** (pyridine-d_5).
**Figure S190.** HMBC spectrum of **26** (pyridine-d_5).
**Figure S191.** ROESY spectrum of **26** (pyridine-d_5).
**Figure S192.** HRESIMS spectrum of **26**.

**Figure S193.** ^{1}H NMR spectrum of **26** (methanol-d_4, 500 MHz).
**Figure S194.** ^{13}C NMR spectrum of **26** (methanol-d_4, 126 MHz).
**Figure S195.** HSQC spectrum of **26** (methanol-d_4).
**Figure S196.** ^{1}H–^{1}H COSY spectrum of **26** (methanol-d_4).
**Figure S197.** HMBC spectrum of **26** (methanol-d_4).
**Figure S198.** ROESY spectrum of **26** (methanol-d_4).

3.1. General Experimental Procedures

Optical rotations were recorded using a JASCO P-1020 Polarimeter (Jasco Corp., Tokyo, Japan). UV spectra were recorded on a Shimadzu UV-2401 PC spectrophotometer (Shimadzu, Kyoto, Japan). Electronic circular dichroism (ECD) spectra were recorded on a Chirascan CD spectrometer (Applied Photophysics Ltd., Leatherhead, UK). IR spectra were measured on a Bruker Tensor 27 FTIR Spectrometer (Bruker Corp., Ettlingen, Germany) with KBr disks. ^{1}H and ^{13}C NMR spectra were collected on Bruker DRX-500, Avance III-600, and Ascend™ 800 MHz NMR spectrometers (Bruker Corporation, Karlsruhe, Germany), with TMS as an internal standard. ESIMS and HRESIMS analyses were performed on an API QSTAR Pulsar 1 spectrometer (Applied Biosystems/MDS Sciex, Foster City, CA, USA). Silica gel G (80–100 and 300–400 mesh, Qingdao Meigao Chemical Co., Ltd., Qingdao, China), C_{18} silica gel (40–75 μm, Fuji Silysia Chemical Ltd., Aichi, Japan), and Sephadex LH-20 (GE Healthcare Bio-Sciences AB, Uppsala, Sweden) were used for column chromatography. Thin-layer chromatography (TLC) spots were visualized under UV light at 254 nm and by dipping in 5% H_2SO_4 in alcohol followed by heating. Semipreparative high-performance liquid chromatography (HPLC) was performed on an Agilent 1200 series pump (Agilent Technologies, Santa Clara, USA) equipped with a diode array detector and a Welch Ultimate AQ-C_{18} column (5 μm, ϕ 4.6 × 300 mm, Welch Materials Inc., Shanghai, China), an Agilent Eclipse XDB-C_{18} column (5.0 μm, ϕ 4.6 × 150 mm), and an Agilent Zorbax SB-C_{18} column (5.0 μm, ϕ 9.4 × 250 mm).

Table S1. ^{1}H and ^{13}C NMR Data of 26 in Methanol-d_4 (δ in ppm, J in Hz)

no.	δ_H (500 MHz)	δ_C (126 MHz)
1	1.60, m	39.7
	0.96, m	
2	1.78, m	27.0
	1.66, m	
3	3.14, dd (11.6, 4.5)	91.1
4		40.2
5	0.78, overlapped	57.0
6	1.54, m	19.3
	1.39, m	
7	1.49, m	34.0
	1.30, m	
8		40.5
9	1.58, m	49.0
10		37.9
11	1.89, m	24.5
12	5.29, br t (3.4)	124.2
13		144.7
14		42.8
15	1.74, m	28.9
	1.07, m	
16	1.99, m	24.2
	1.64, m	
17		47.0
18	2.69, dd (13.7, 3.5)	44.0
19	1.92, m	43.3
	1.65, dd (13.7, 13.7)	
20		45.0
21	1.98, m	31.3
	1.35, m	
22	1.60, m	35.0
	1.56, m	
23	1.04, s	28.5
24	0.84, s	16.9
25	0.94, s	15.9
26	0.79, s	17.7
27	1.16, s	26.3
28		181.2
29	1.13, s	28.7
30		178.8
30-OMe	3.69, s	52.3
1′	4.38, d (7.8)	107.0
2′	3.22, dd (9.1, 7.8)	75.3

3′	3.35, dd (9.1, 9.1)	77.5
4′	3.50, dd (9.8, 9.1)	73.2
5′	3.82, d (9.8)	76.6
6′		171.4
6′-OMe	3.76, s	52.8

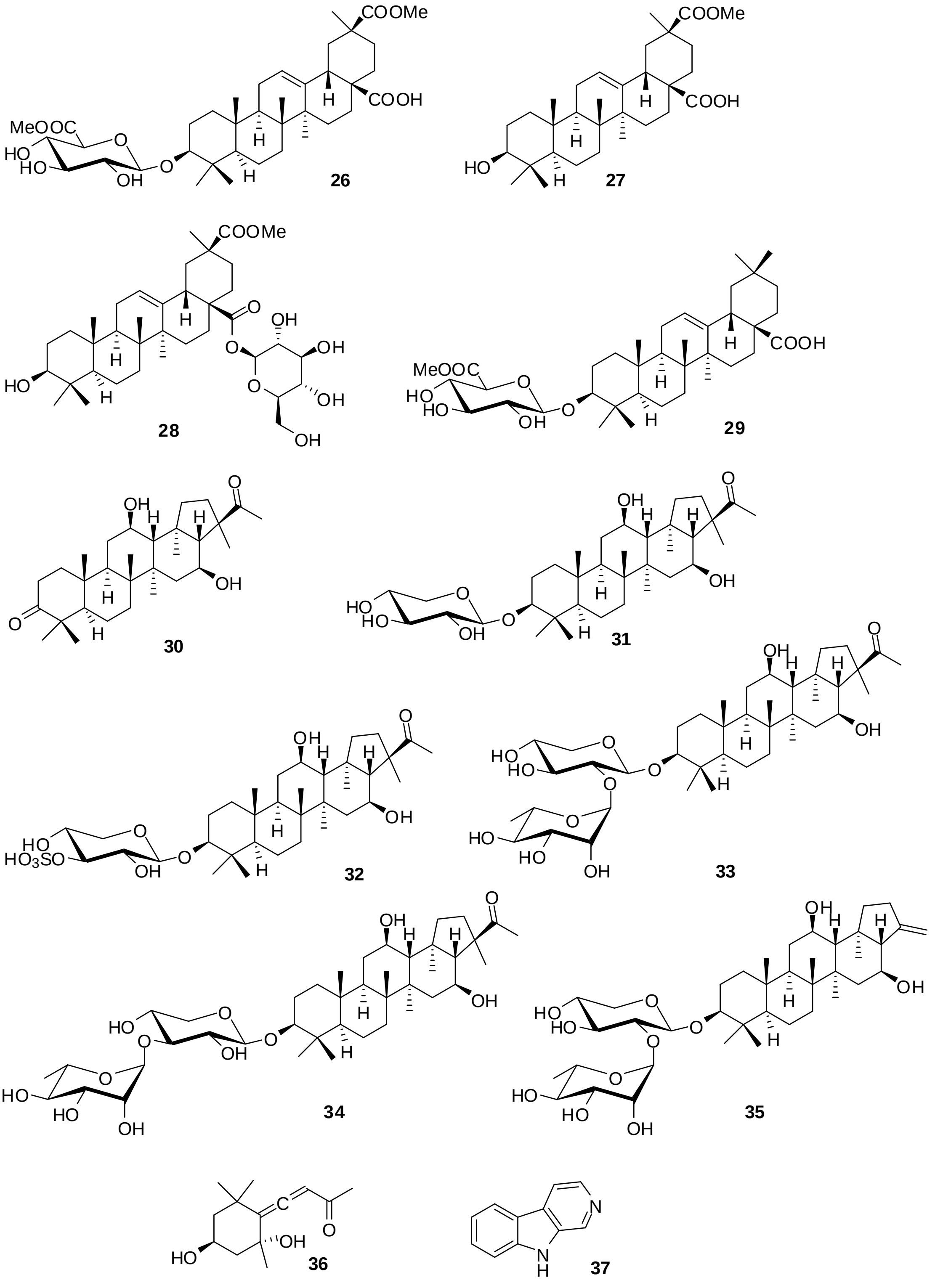

Figure S1. Structures of known compounds (**26**–**37**) from *Glinus oppositifolius*.

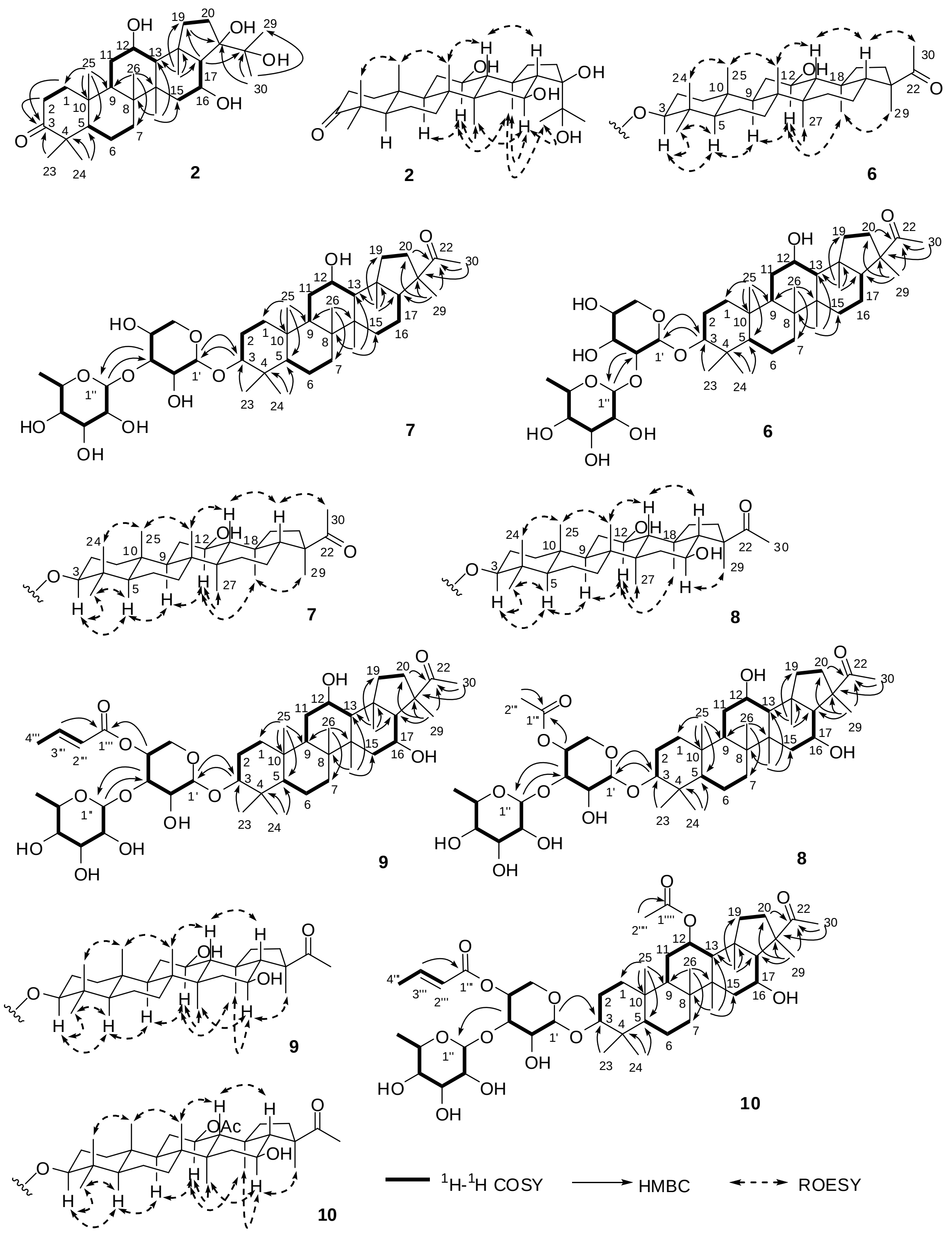

Figure S2. Key 2D NMR correlations of **2** and **6–10**.

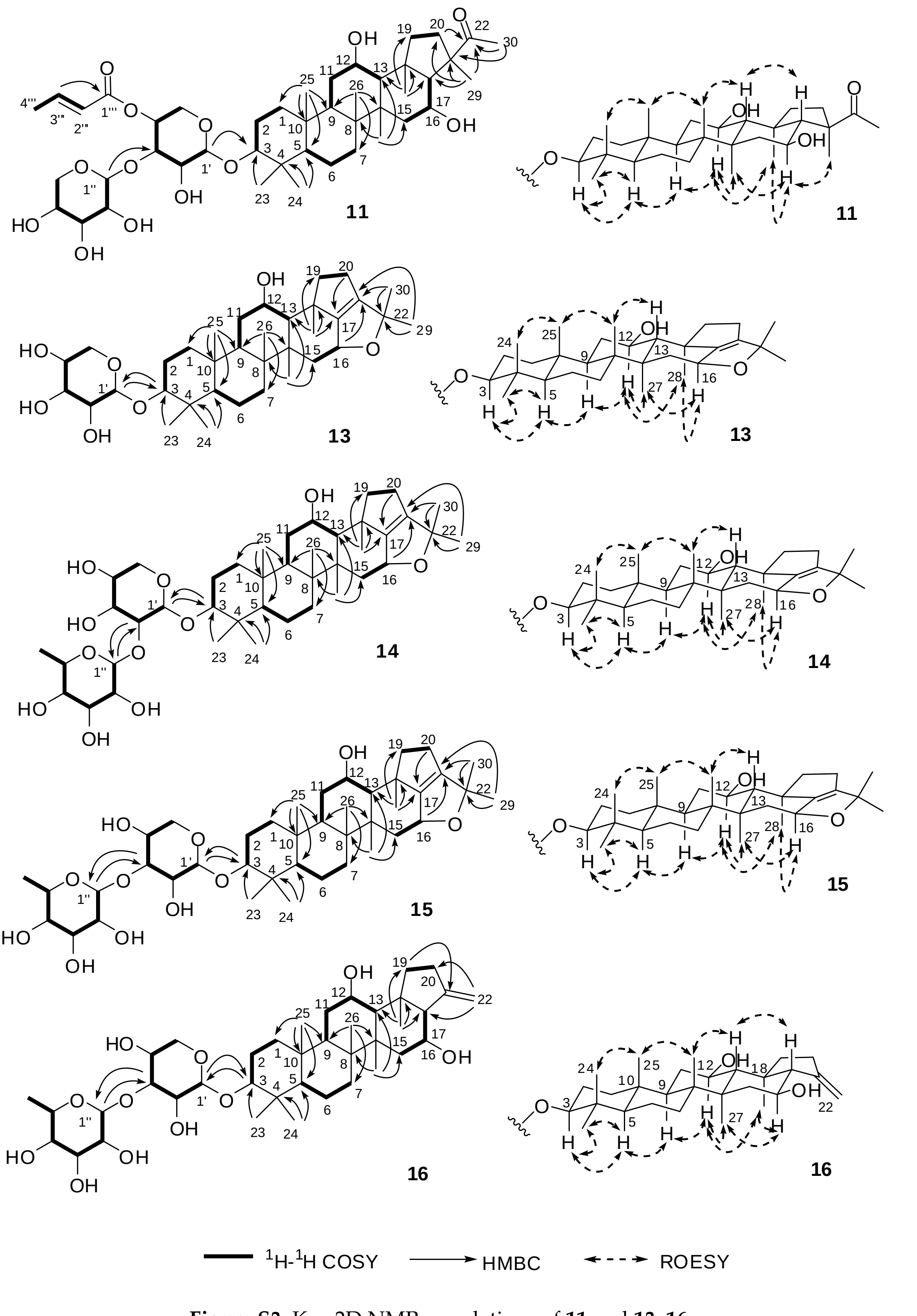

Figure S3. Key 2D NMR correlations of **11** and **13–16**.

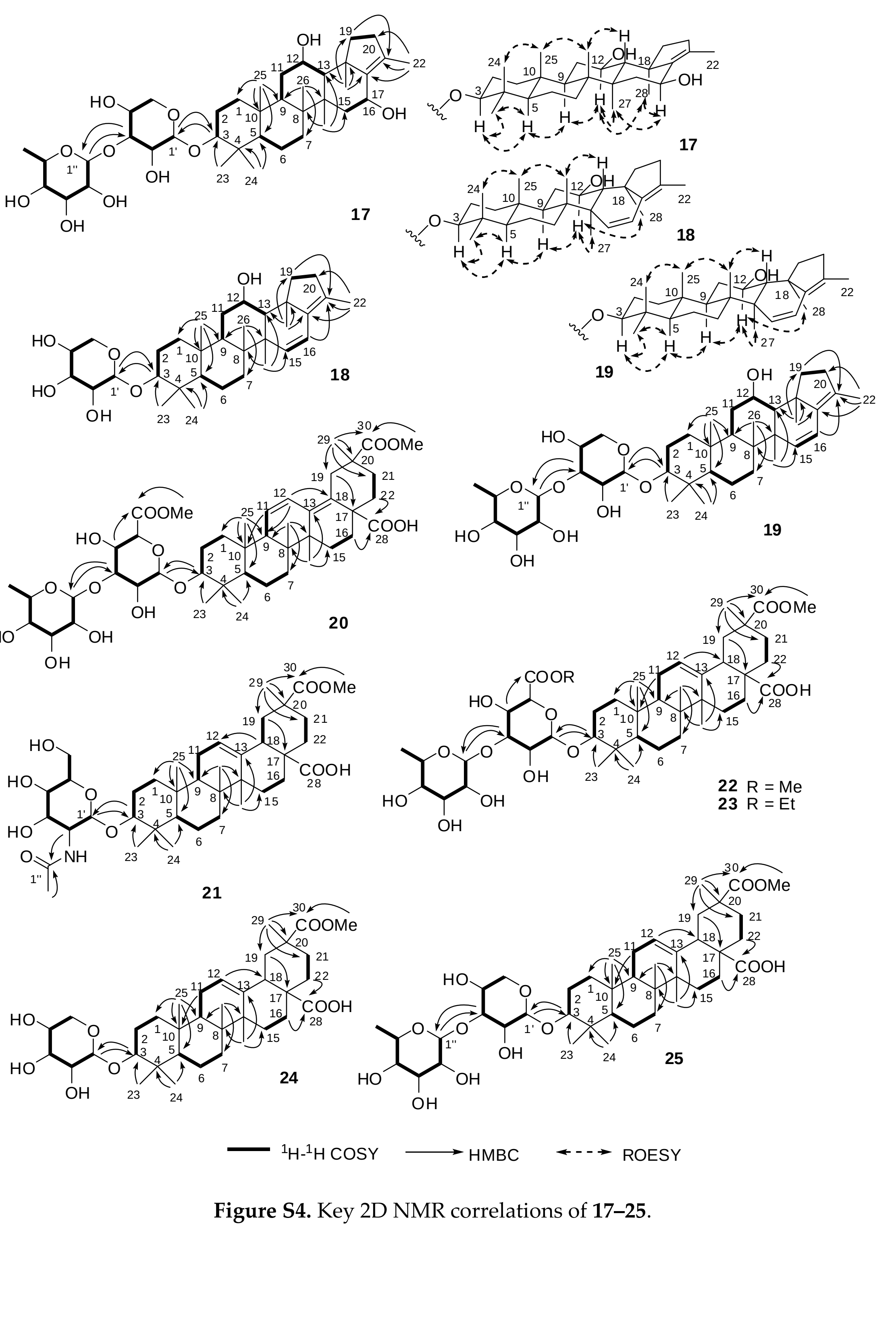

Figure S4. Key 2D NMR correlations of **17–25**.

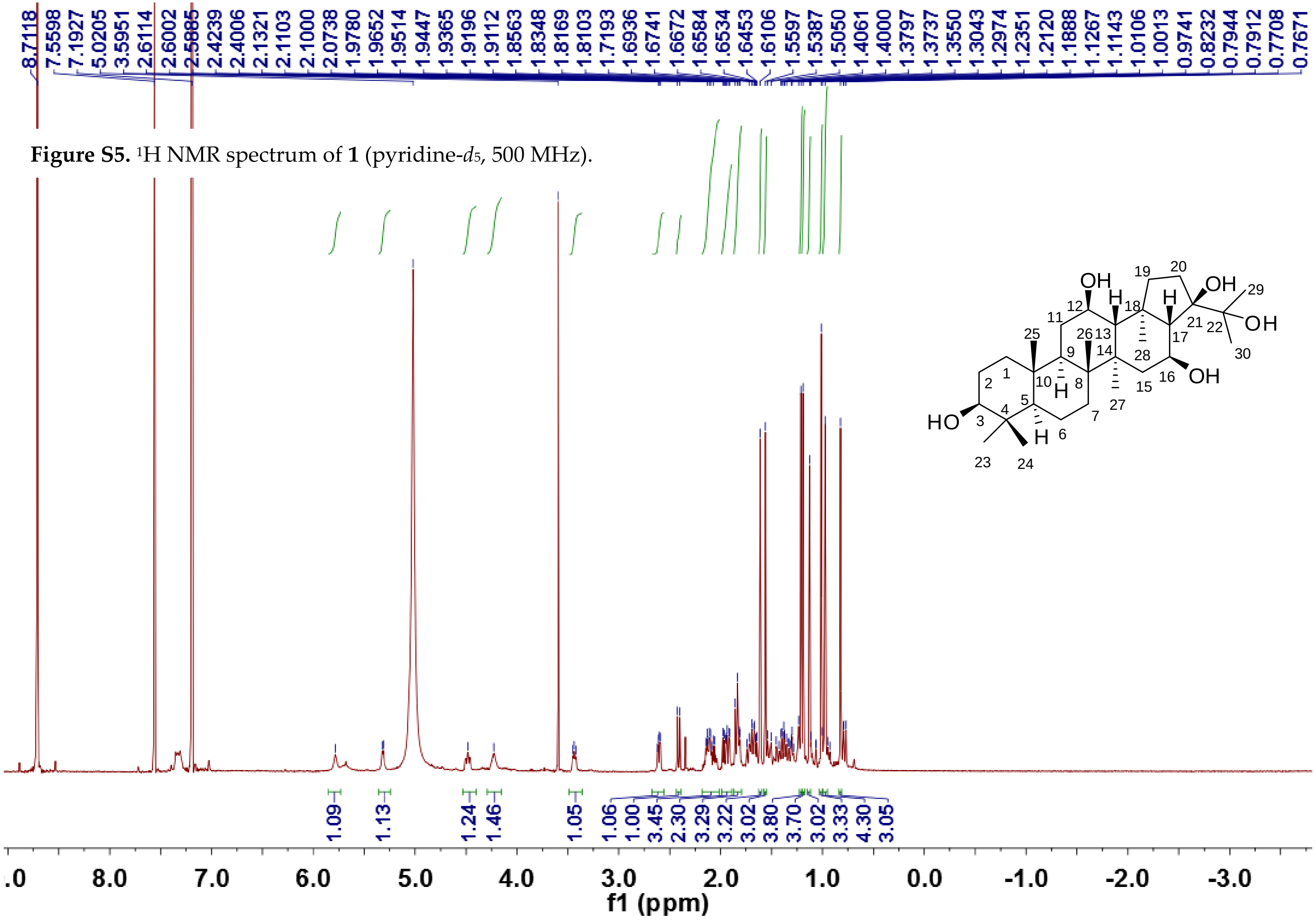

Figure S5. ^{1}H NMR spectrum of **1** (pyridine-d_5, 500 MHz).

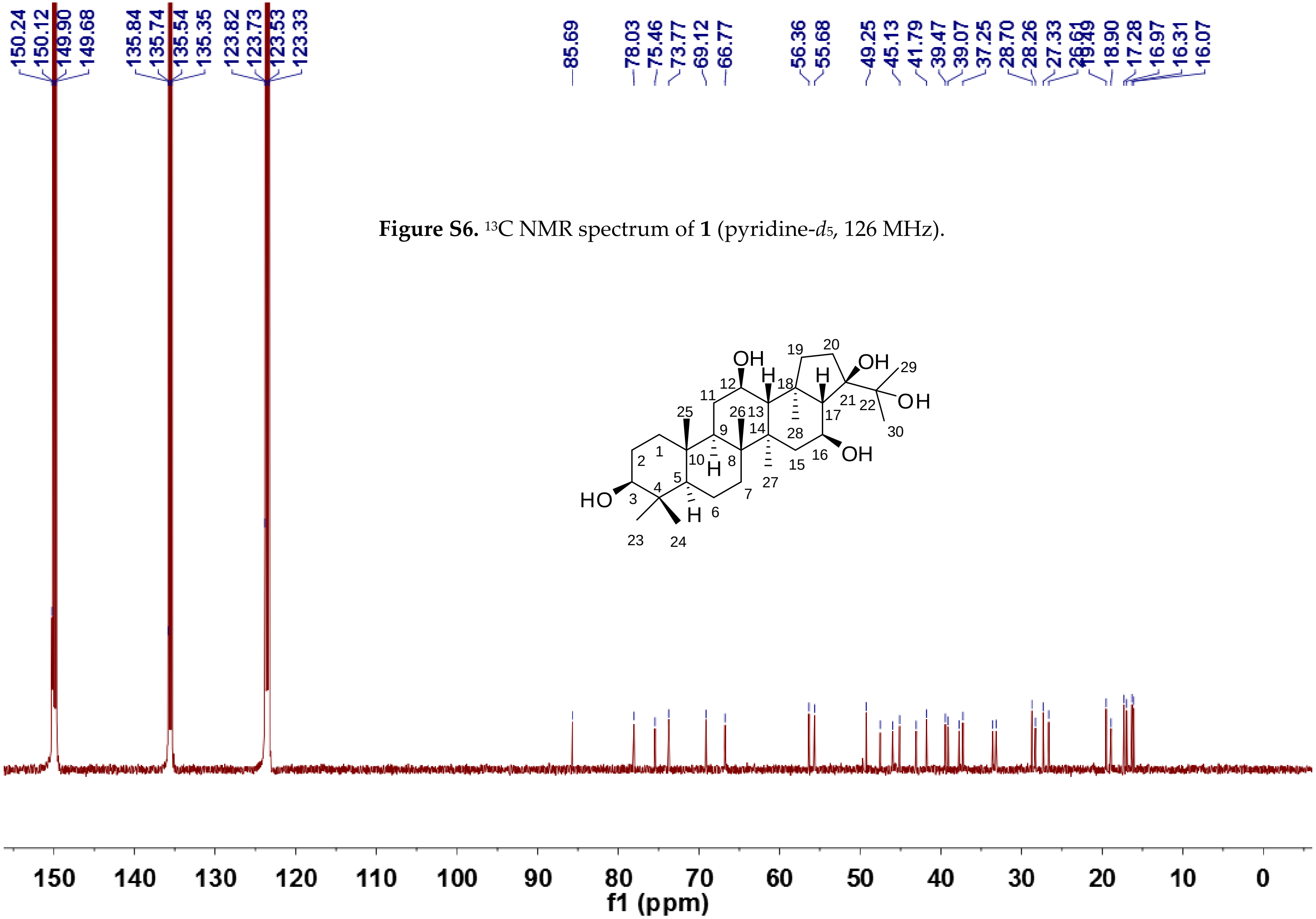

Figure S6. ^{13}C NMR spectrum of **1** (pyridine-d_5, 126 MHz).

Figure S7. HSQC spectrum of **1**.

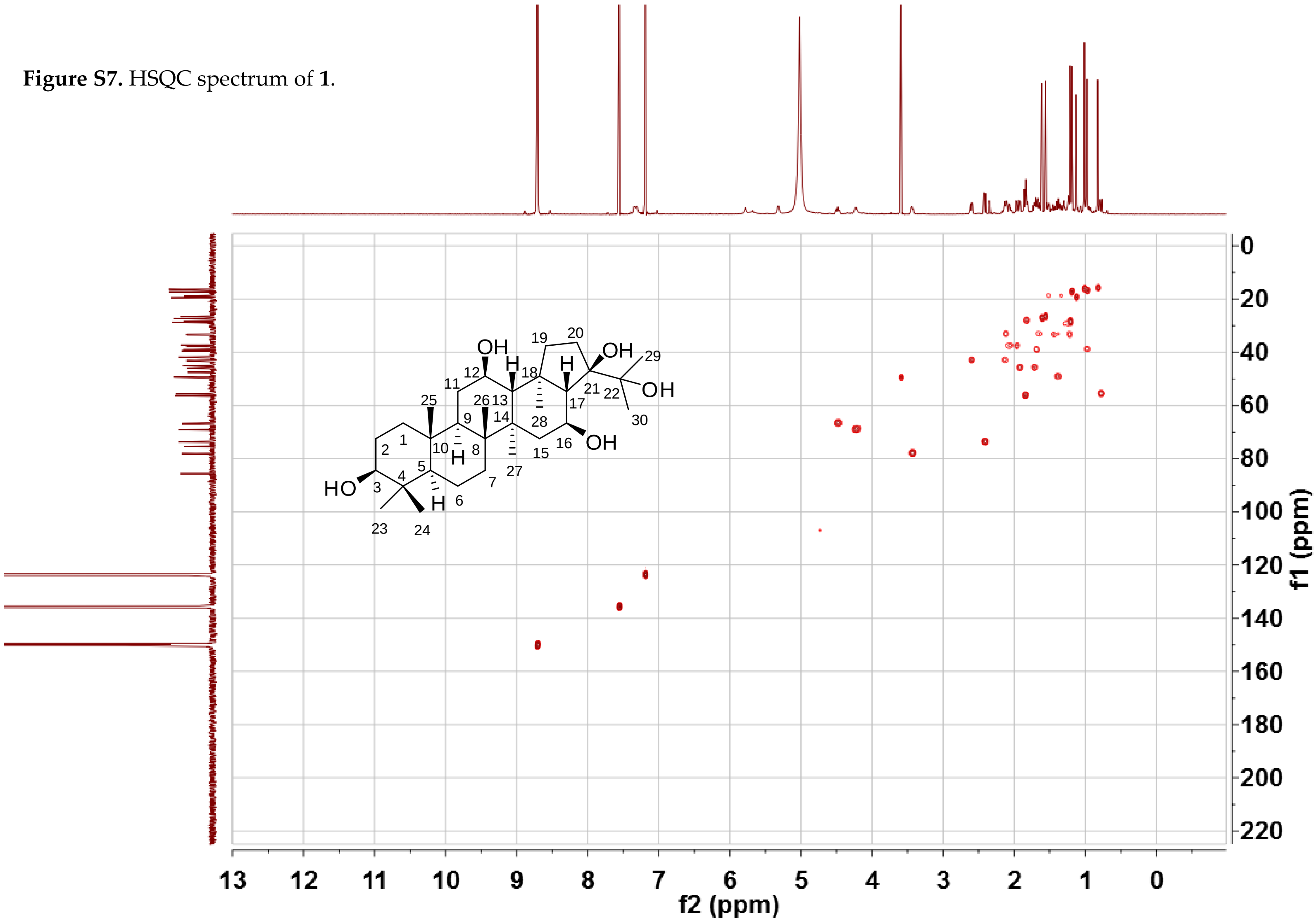

Figure S8. ^{1}H–^{1}H COSY spectrum of **1**.

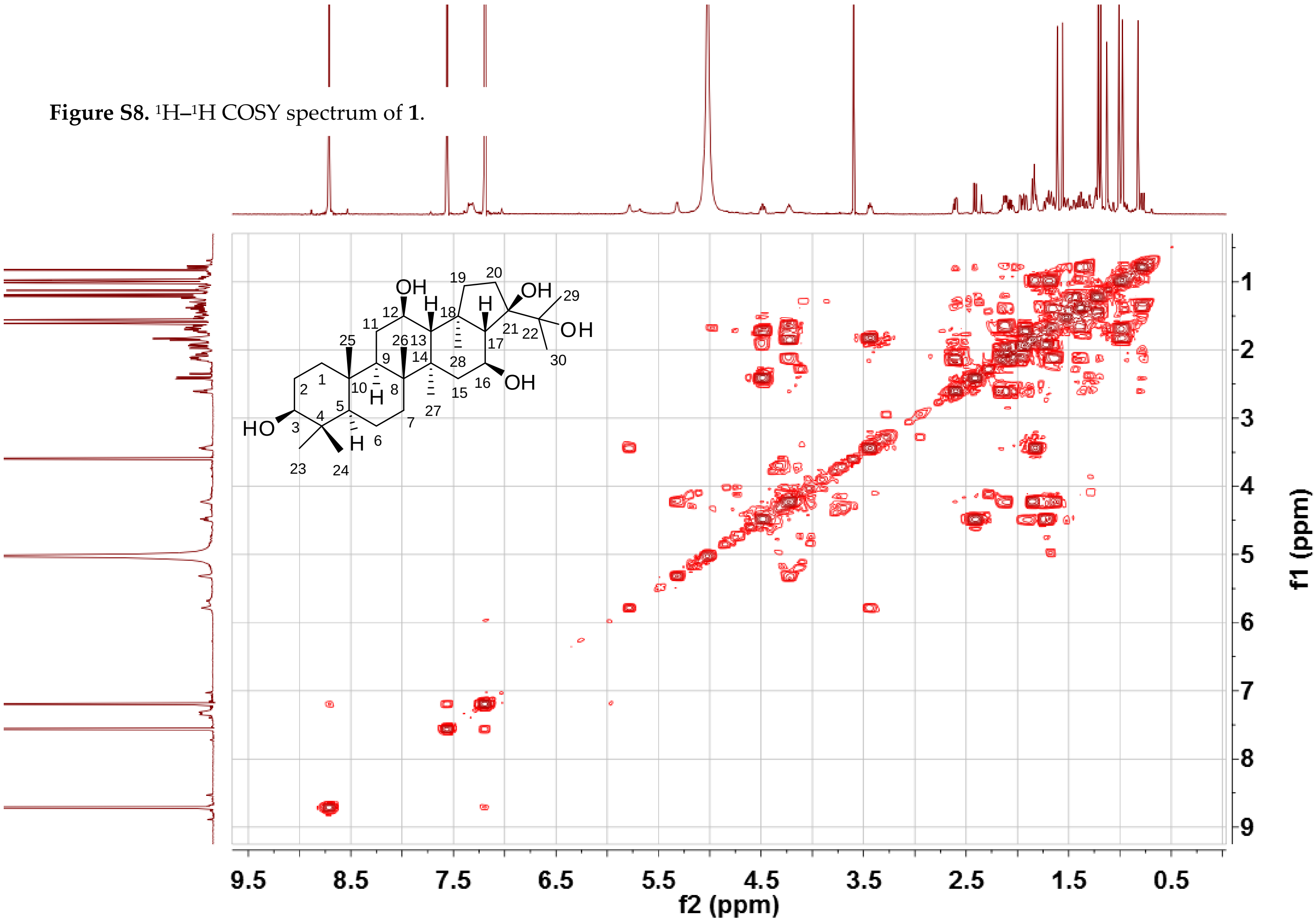

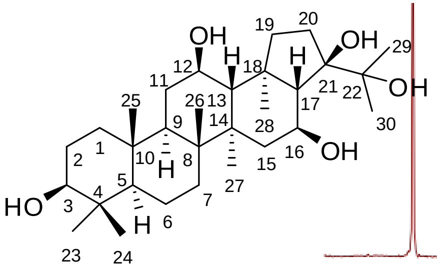

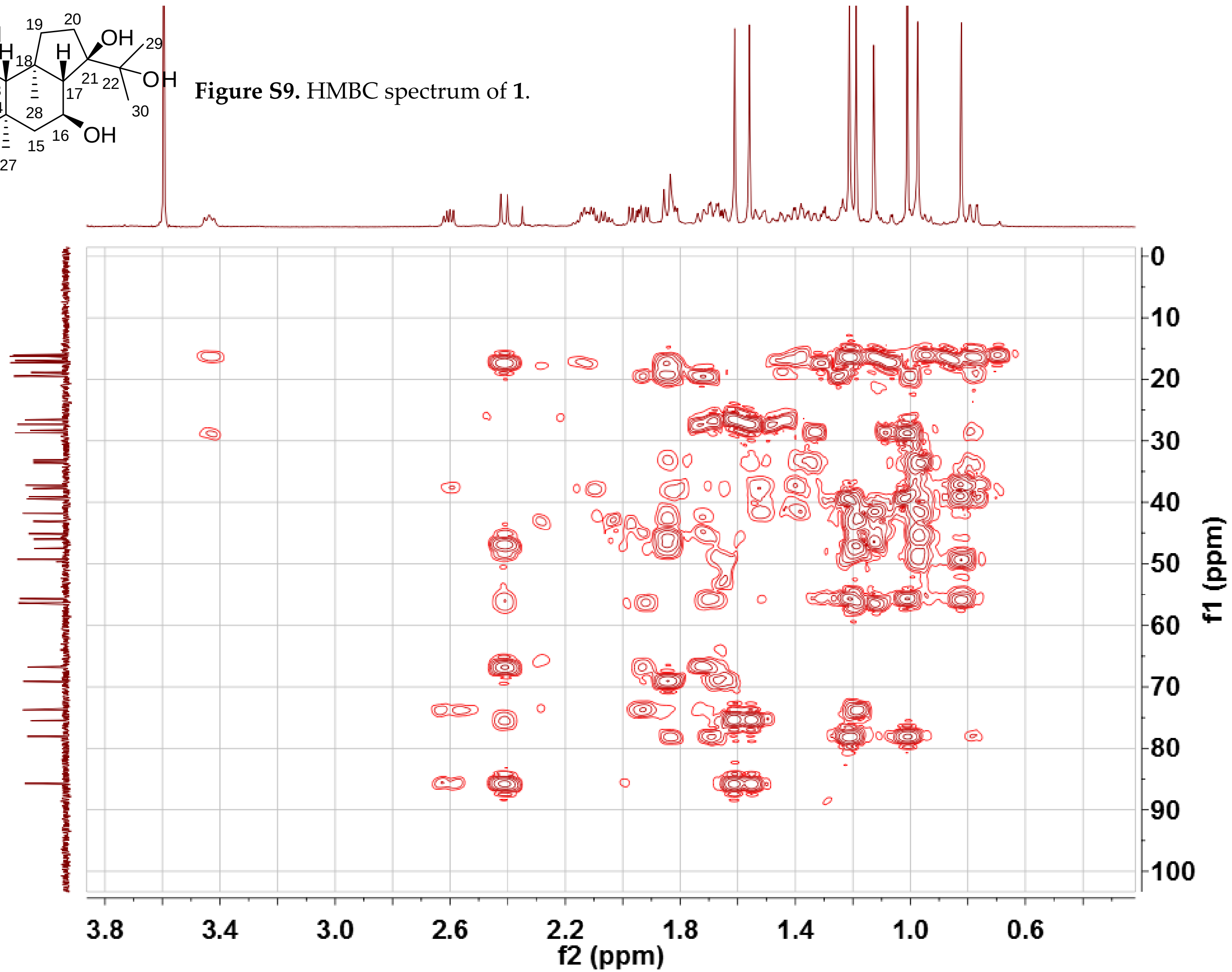

Figure S9. HMBC spectrum of **1**.

Figure S10. ROESY spectrum of **1**.

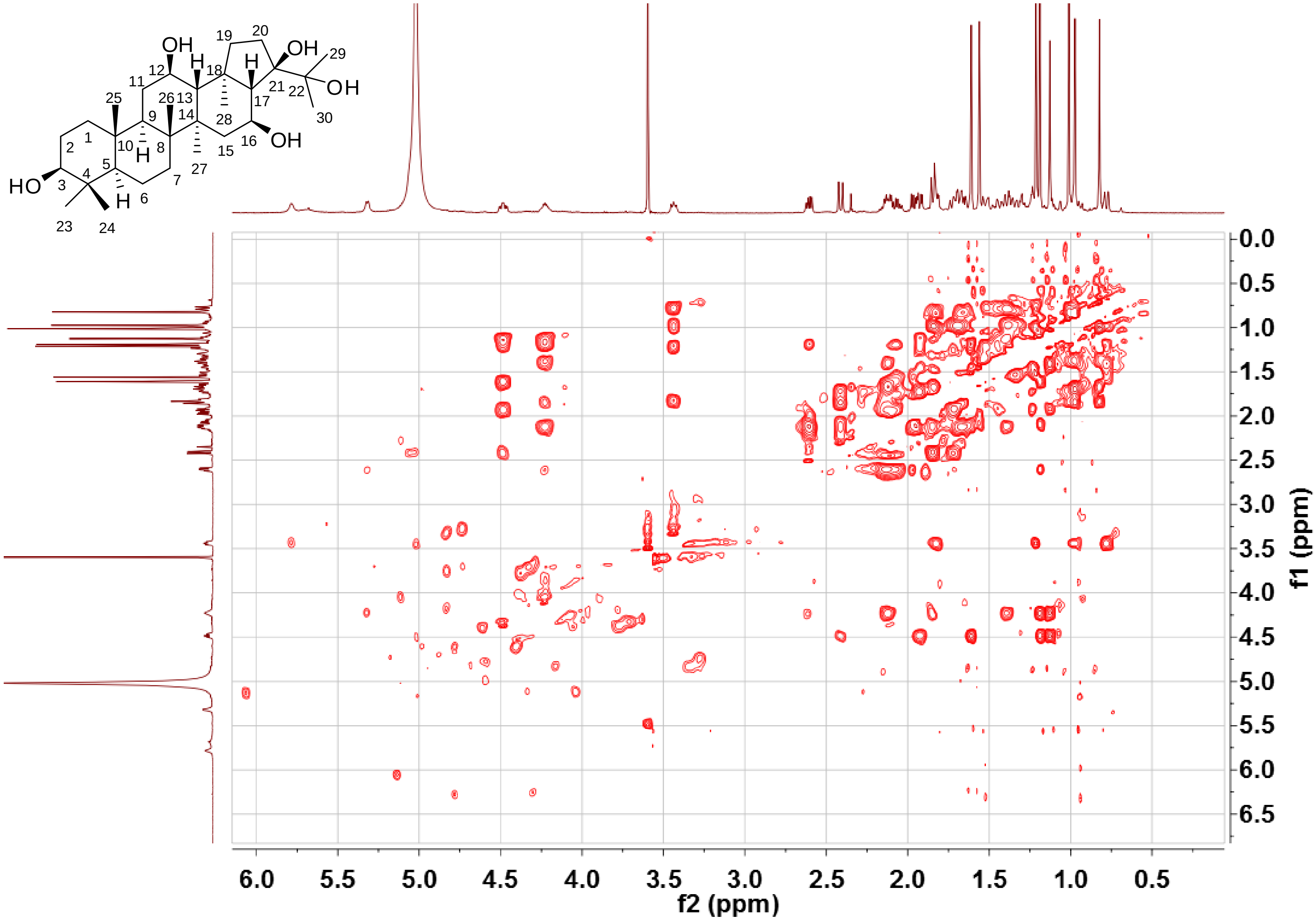

Figure S11. HRESIMS spectrum of **1**.

**Data Filename**	171020ESIA5.d	**Sample Name**	pdt11
**Sample Type**	Sample	**Position**	
**Instrument Name**	Agilent G6230 TOF MS	**User Name**	KIB
**Acq Method**	ESI.m	**Acquired Time**	10/23/2017 10:45:31 AM
**IRM Calibration Status**	Success	**DA Method**	ESI.m
**Comment**			

Sample Group **Info.**
Acquisition SW Version 6200 series TOF/6500 series Q-TOF B.05.01 (B5125.2)

User Spectra

Fragmentor Voltage	Collision Energy	Ionization Mode
230	0	ESI

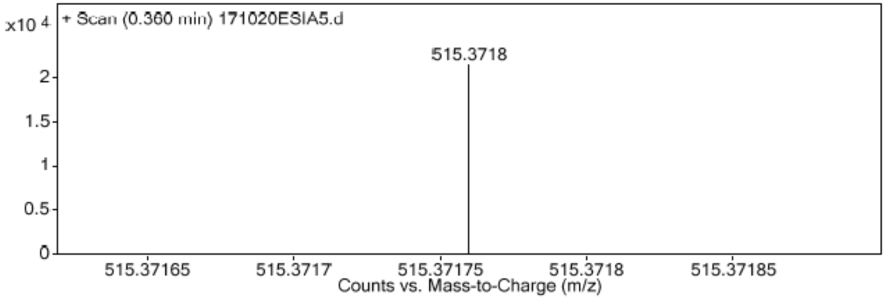

Peak List

m/z	z	Abund	Formula	Ion
100.1114		7040.62		
121.0509		7712.63		
125.1122	1	12984.43		
142.1592	1	30774.7		
186.2219	1	23846.48		
242.2848	1	104219.18		
243.2875	1	13899.02		
274.2739	1	6901.11		
515.3718	1	21392.95	C30 H52 Na O5	M+
922.0098	1	8991.66		

Formula Calculator Element Limits

Element	Min	Max
C	0	200
H	0	400
O	0	10
Na	1	1

Formula Calculator Results

Formula	CalculatedMass	Mz	Diff.(mDa)	Diff. (ppm)	DBE
C30 H52 Na O5	515.3712	515.3718	-0.6	1.1	4.5

--- End Of Report ---

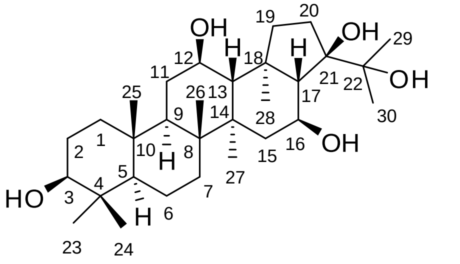

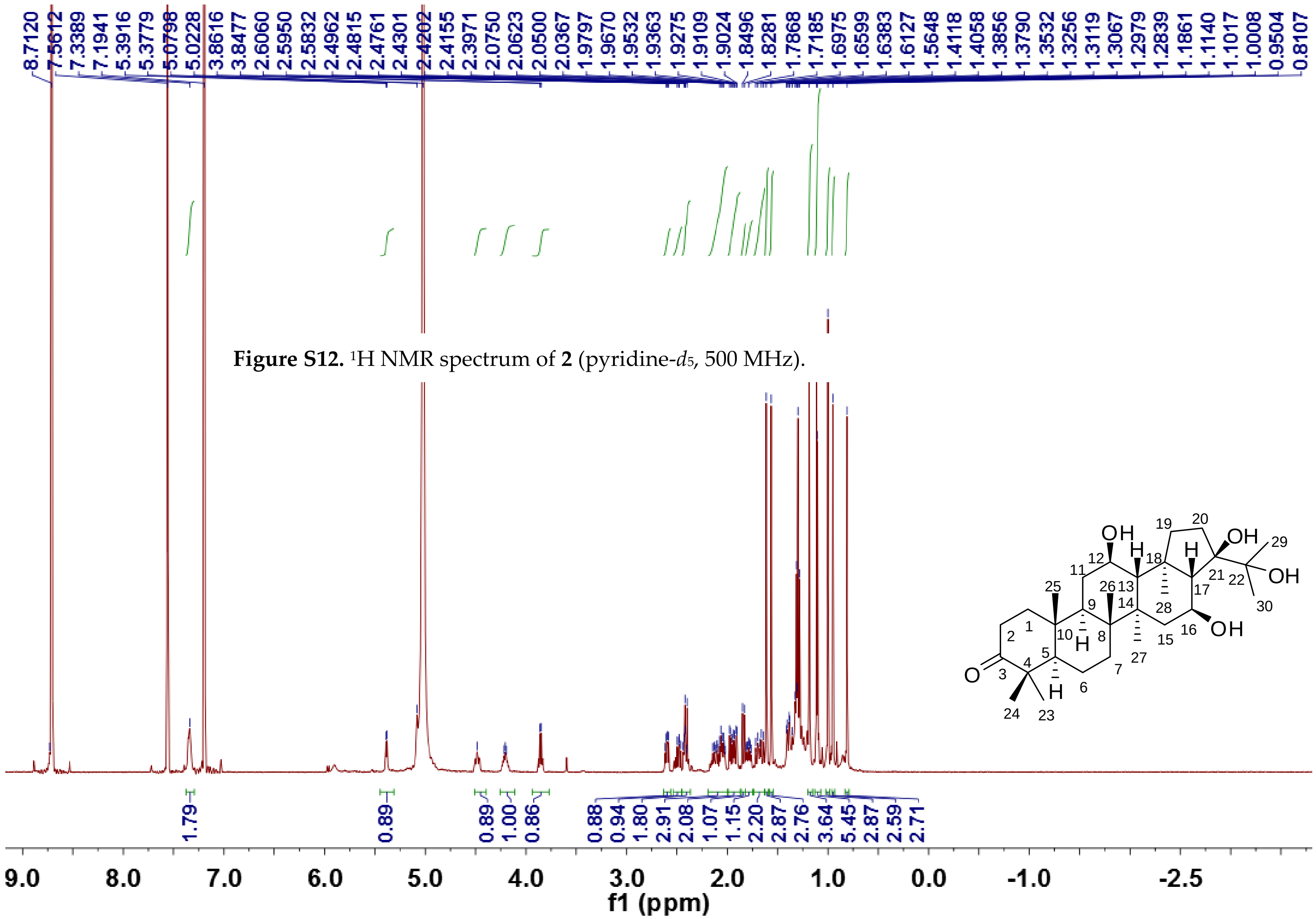

Figure S12. ^{1}H NMR spectrum of **2** (pyridine-d_5, 500 MHz).

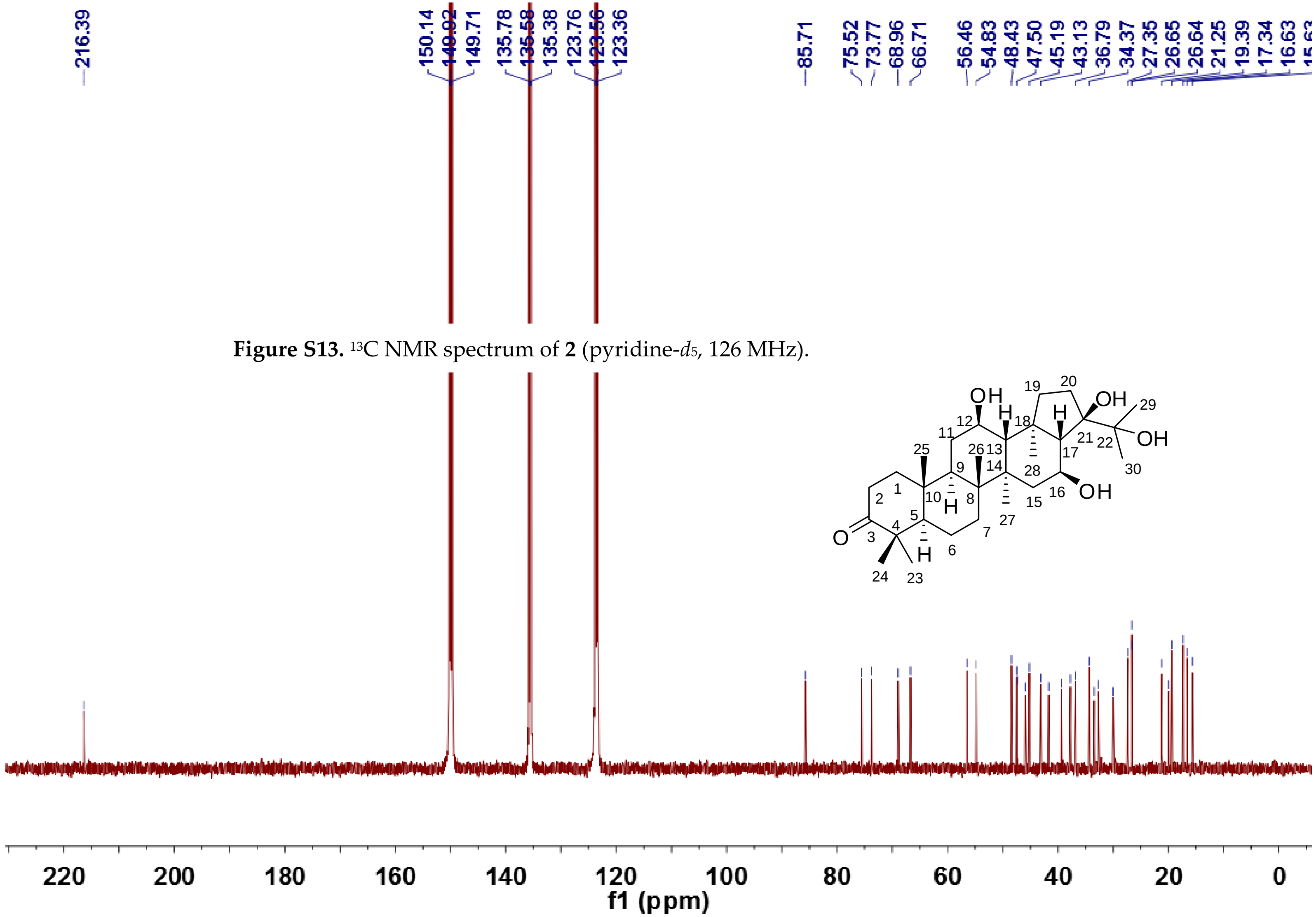

Figure S13. ^{13}C NMR spectrum of **2** (pyridine-*d*5, 126 MHz).

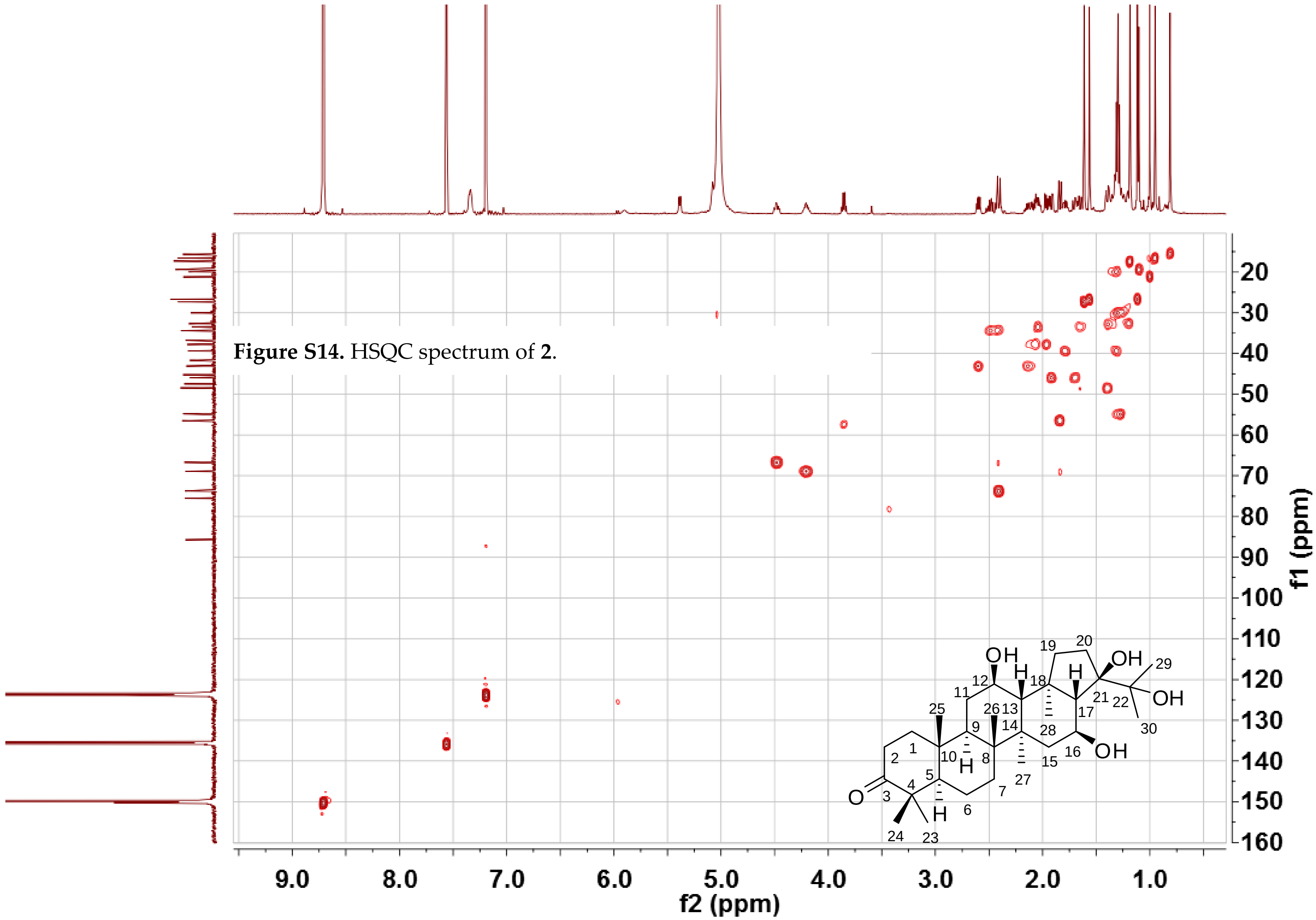

Figure S14. HSQC spectrum of **2**.

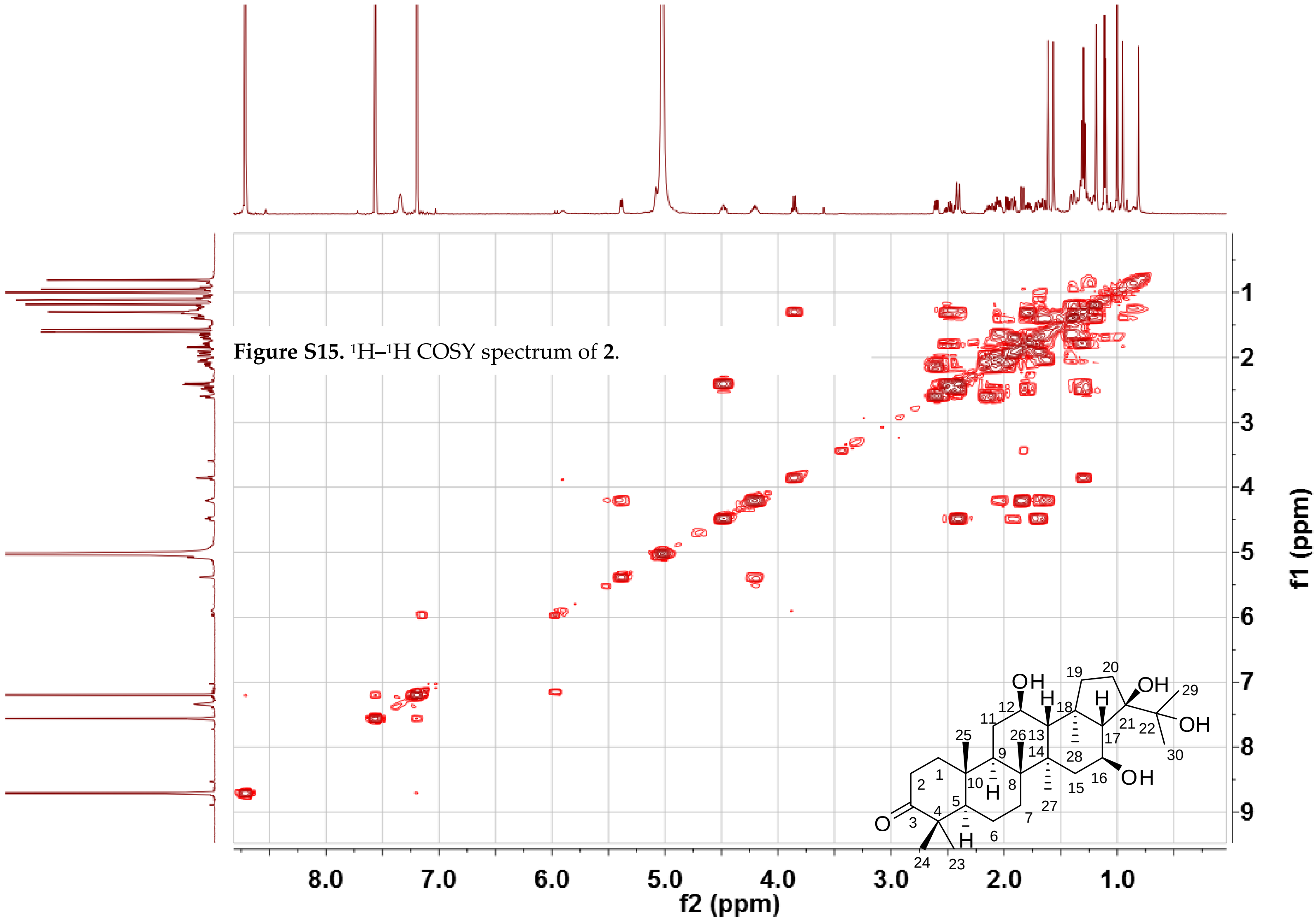

Figure S15. ^{1}H–^{1}H COSY spectrum of **2**.

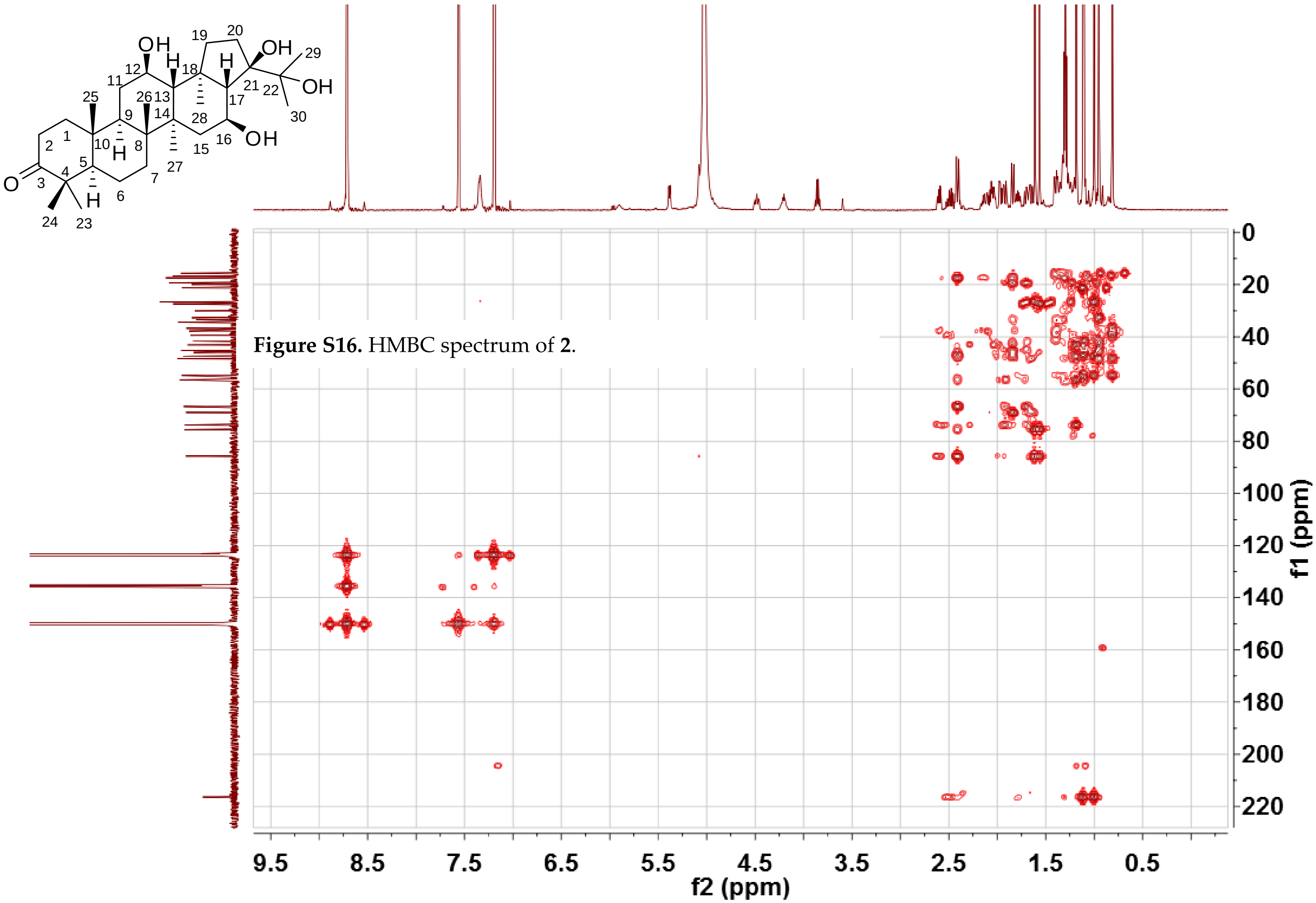

Figure S16. HMBC spectrum of **2**.

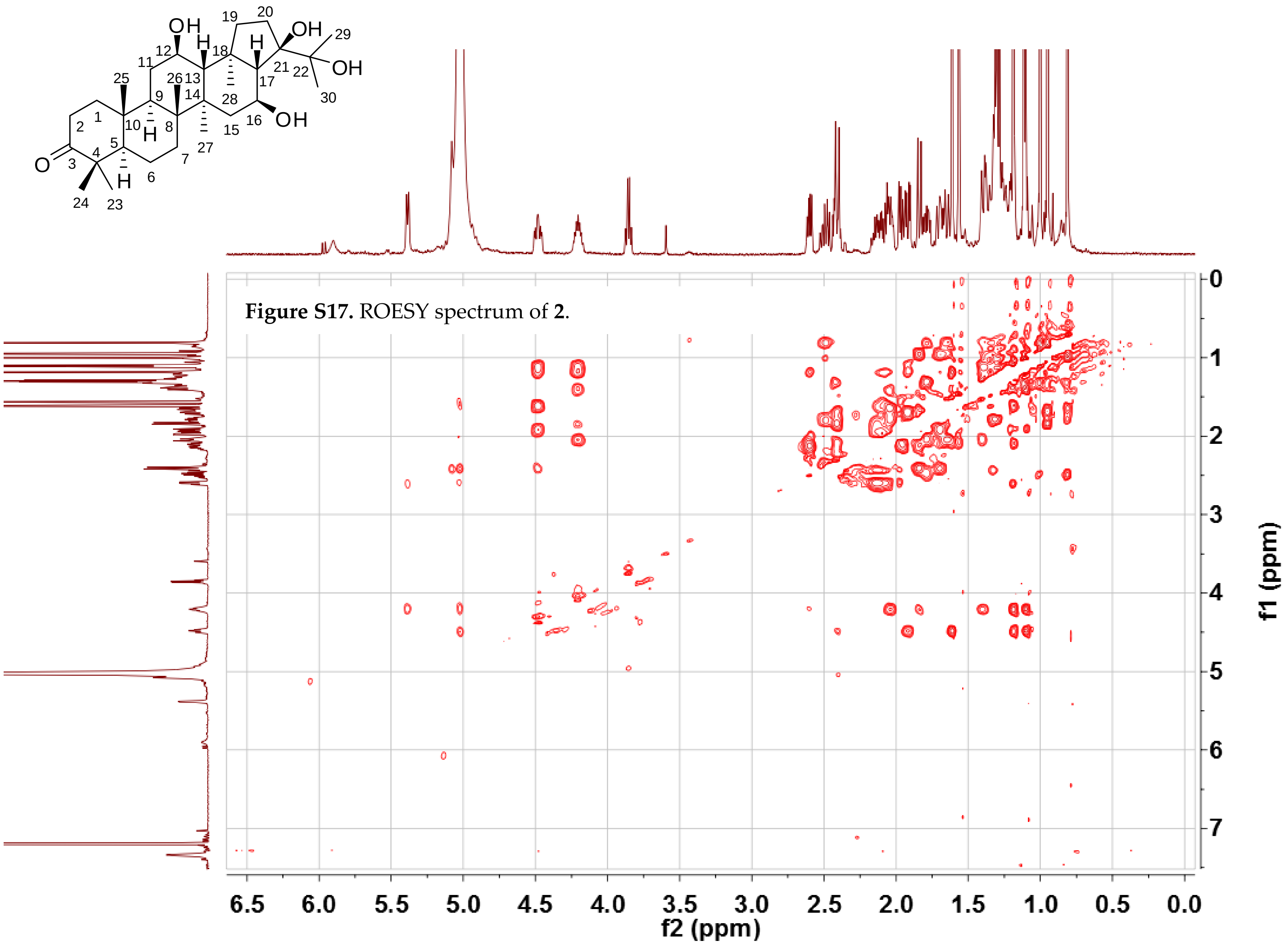

Figure S17. ROESY spectrum of **2**.

Figure S18. HRESIMS spectrum of **2**.

**Data Filename**	171020ESIA6.d	**Sample Name**	pdt16
**Sample Type**	Sample	**Position**	
**Instrument Name**	Agilent G6230 TOF MS	**User Name**	KIB
**Acq Method**	ESI.m	**Acquired Time**	10/23/2017 10:47:04 AM
**IRM Calibration Status**	Success	**DA Method**	ESI.m
**Comment**			

**Sample Group**		**Info.**
**Acquisition SW Version**	6200 series TOF/6500 series Q-TOF B.05.01 (B5125.2)	

User Spectra

Fragmentor Voltage	Collision Energy	Ionization Mode
230	0	ESI

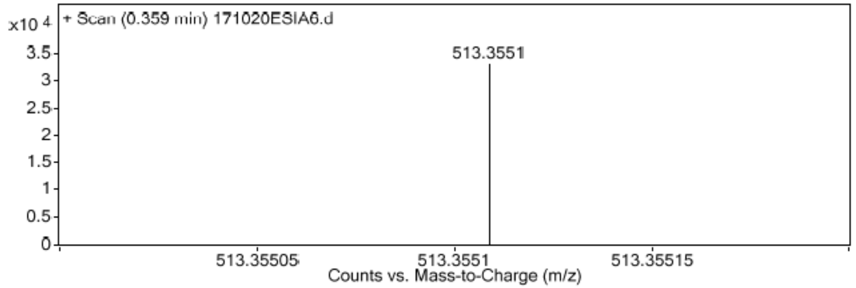

Peak List

m/z	z	Abund	Formula	Ion
121.0509	1	12467.49		
142.1588	1	9737.21		
242.284	1	25951.61		
513.3551	1	33099.68	C30 H50 Na O5	M+
514.3579	1	8436.8	C30 H50 Na O5	M+
701.4931	1	15047.89		
814.5779	1	7262.12		
922.0098	1	23822.68		
1003.7204	1	17353.7		
1004.7223	1	10234.4		

Formula Calculator Element Limits

Element	Min	Max
C	0	200
H	0	400
O	0	10
Na	1	1

截图(Alt + A)

Formula Calculator Results

Formula	CalculatedMass	Mz	Diff.(mDa)	Diff. (ppm)	DBE
C30 H50 Na O5	513.3556	513.3551	0.5	1.0	5.5

--- End Of Report ---

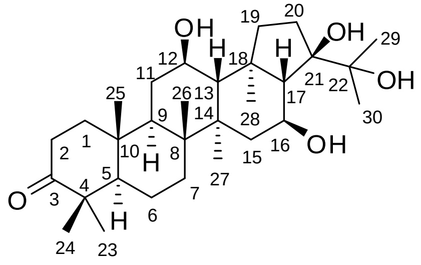

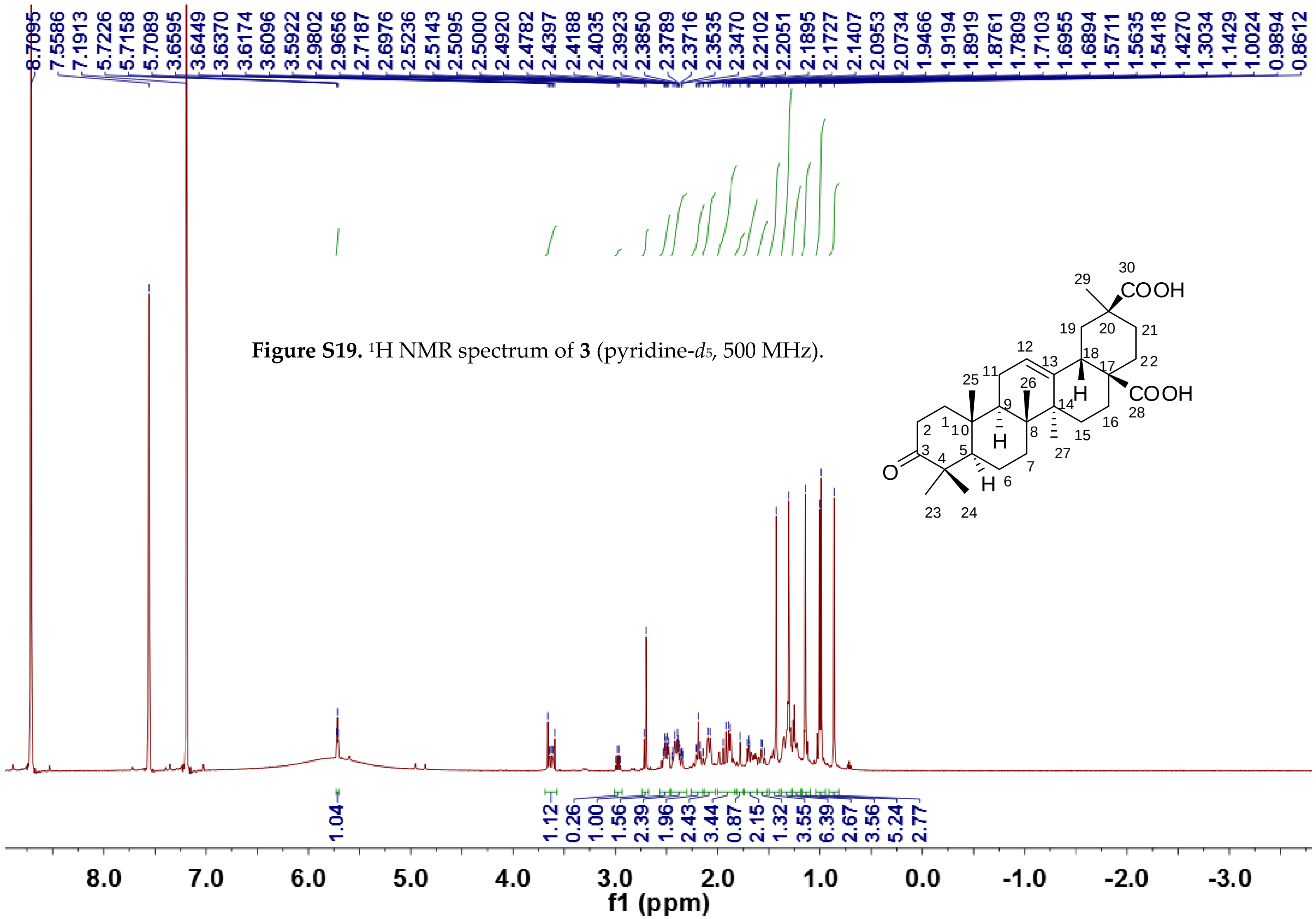

Figure S19. ^{1}H NMR spectrum of **3** (pyridine-*d*5, 500 MHz).

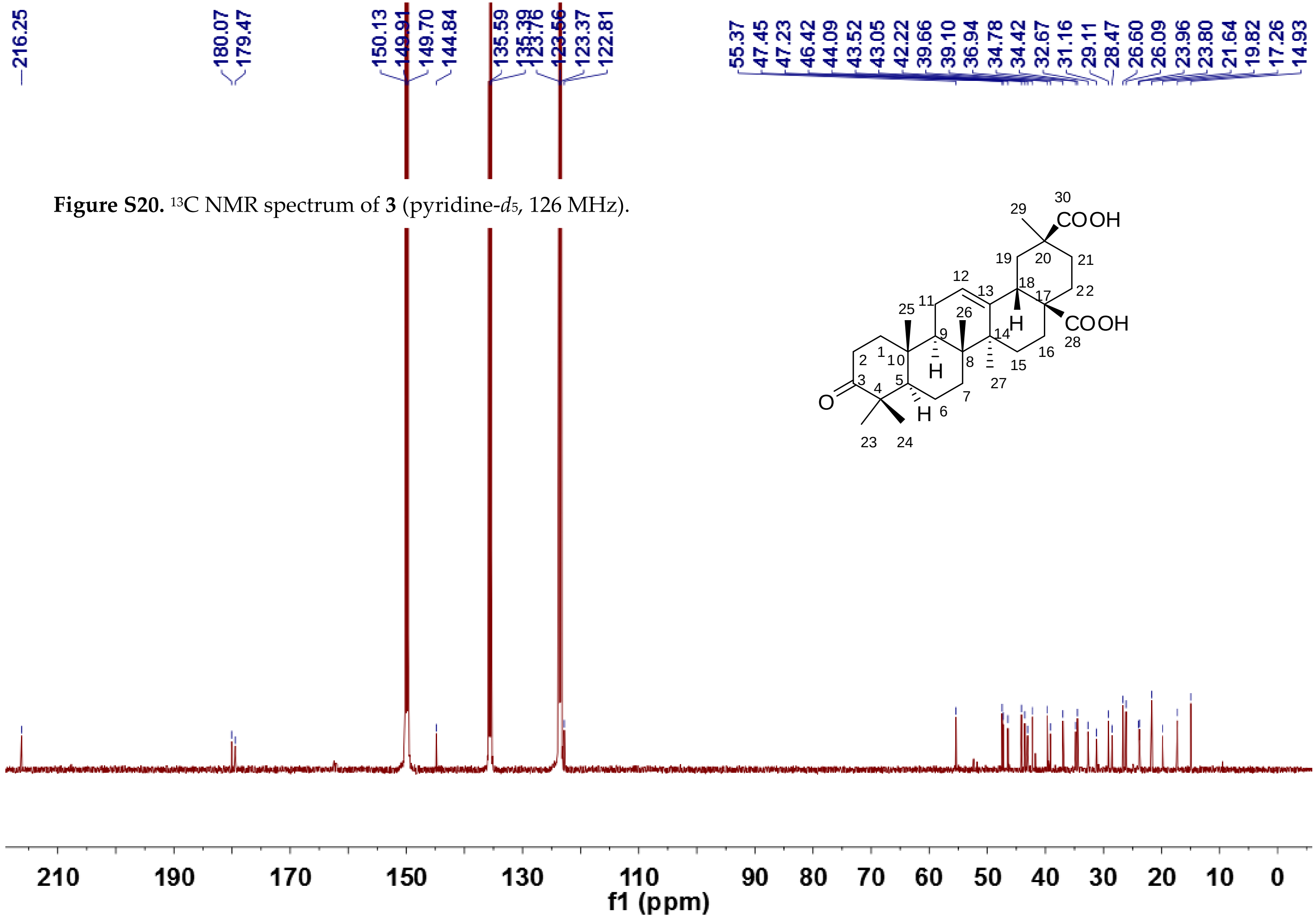

Figure S20. ^{13}C NMR spectrum of **3** (pyridine-*d*5, 126 MHz).

Figure S21. HSQC spectrum of **3**.

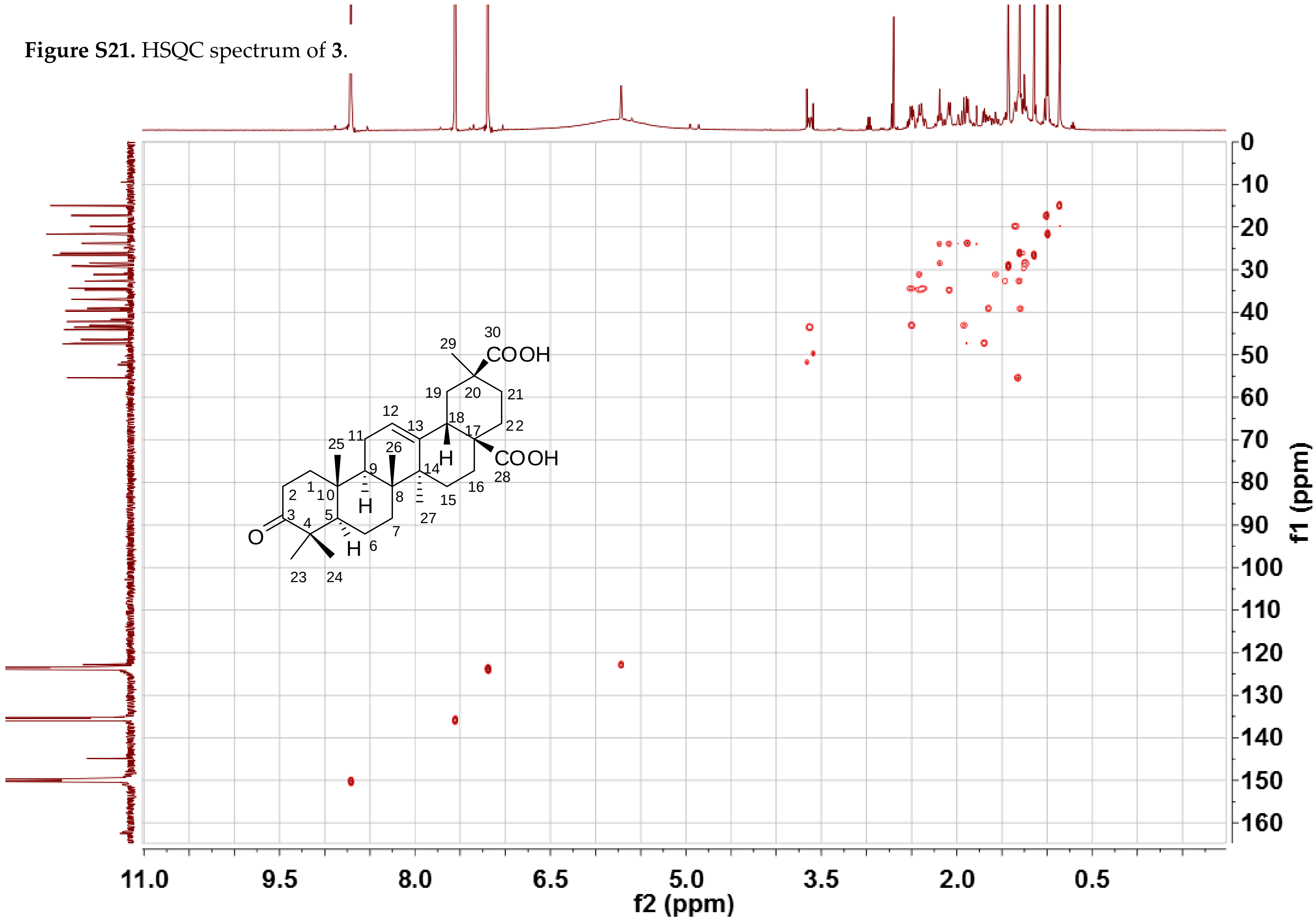

Figure S22. ¹H–¹H COSY spectrum of **3**.

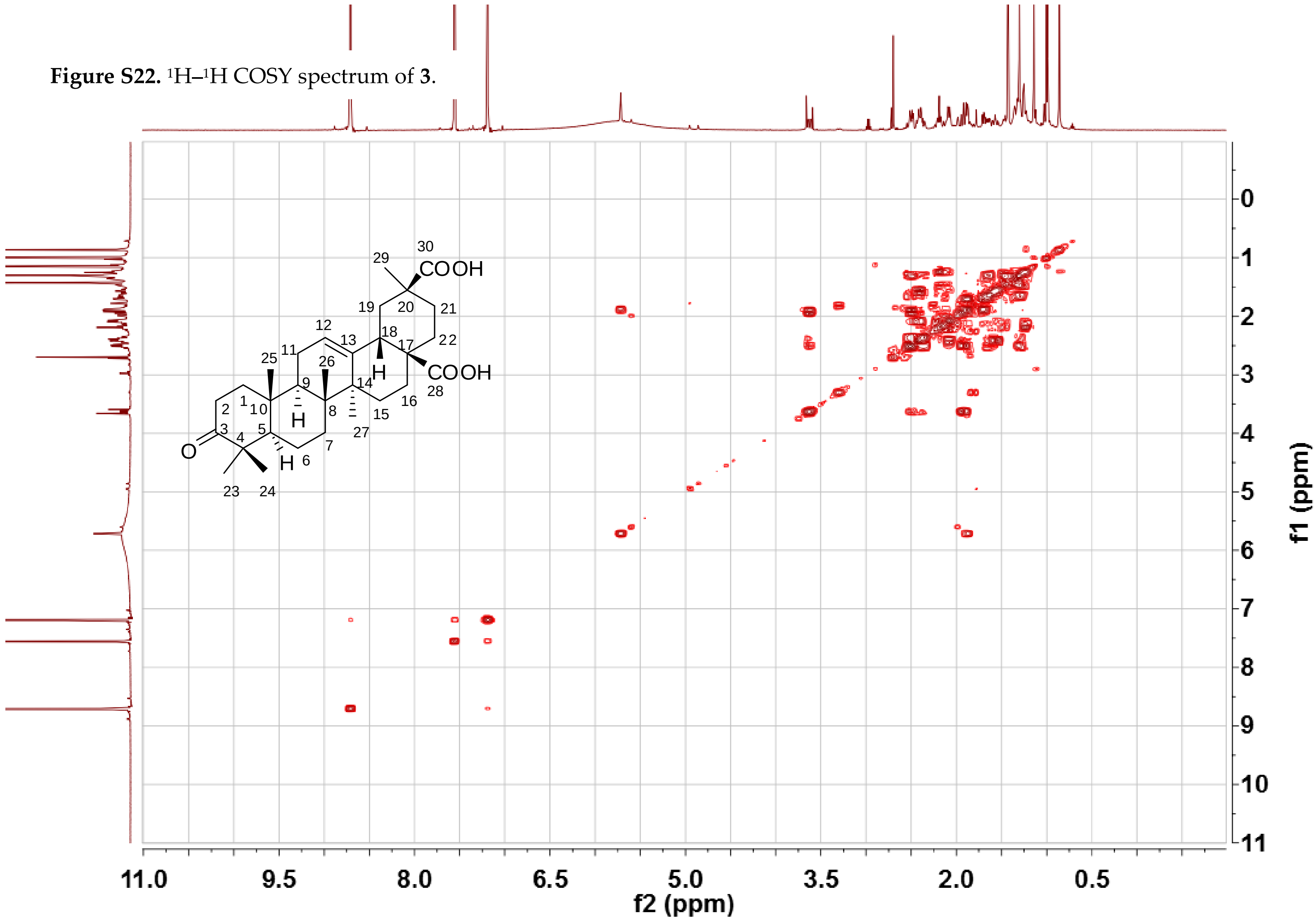

Figure S23. HMBC spectrum of **3**.

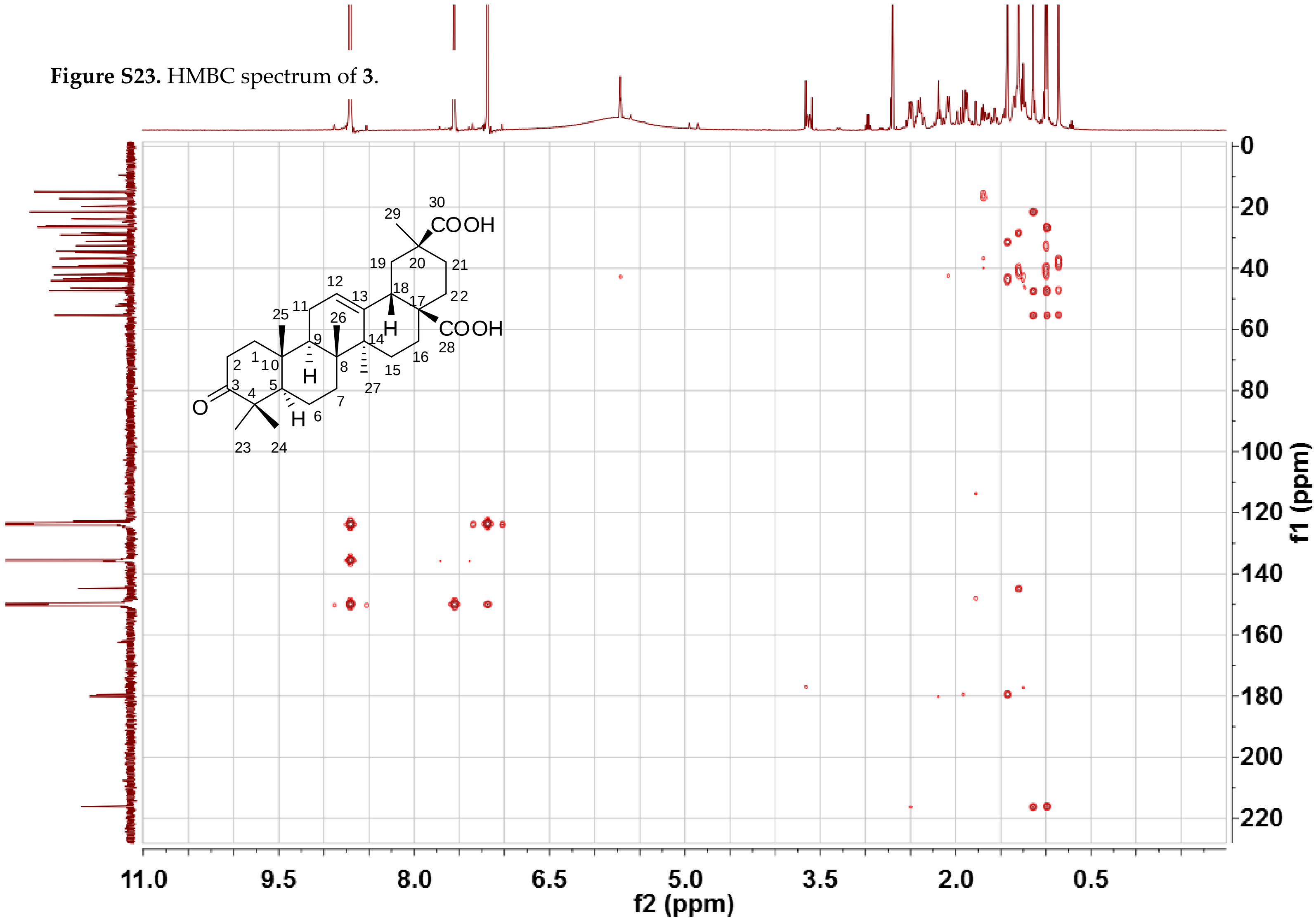

Figure S24. ROESY spectrum of **3**.

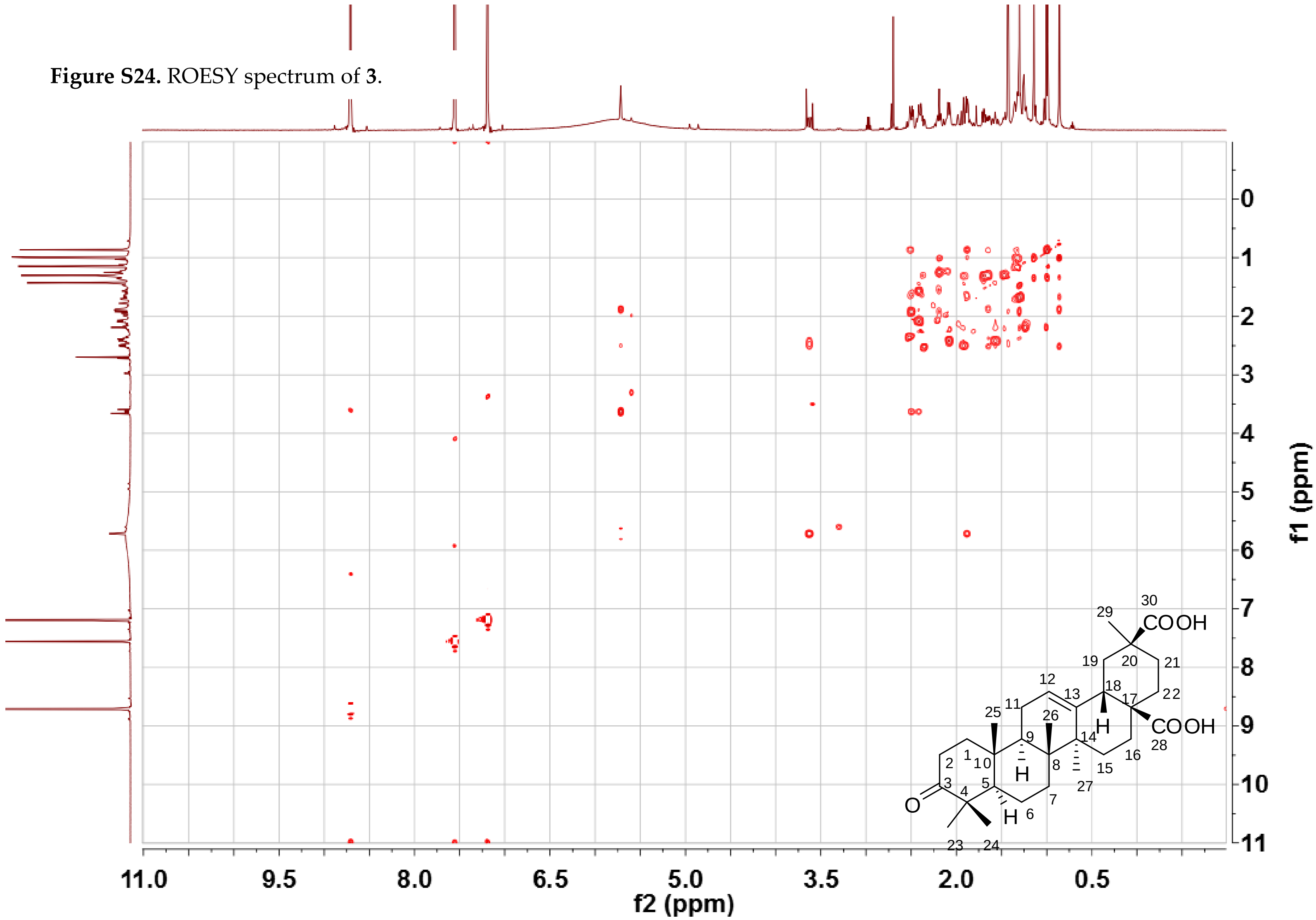

**Data Filename**	190107ESIA5.d	**Sample Name**	pdt31
**Sample Type**	Sample	**Position**	
**Instrument Name**	Agilent G6230 TOF MS	**User Name**	KIB
**Acq Method**	ESI.m	**Acquired Time**	1/7/2019 3:09:04 PM
**IRM Calibration Status**	Success	**DA Method**	ESI.m
**Comment**			

**Sample Group**		**Info.**
**Acquisition SW Version**	6200 series TOF/6500 series Q-TOF B.05.01 (B5125.2)	

User Spectra

Fragmentor Voltage	Collision Energy	Ionization Mode
320	0	ESI

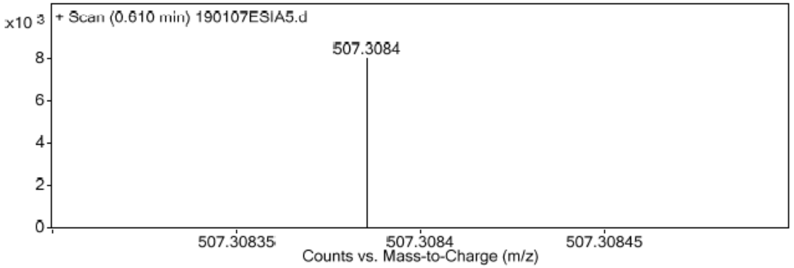

Peak List

m/z	z	Abund	Formula	Ion
105.0435		6260.53		
112.1882		7240.67		
166.063	1	12333.17		
182.0406	1	60230.32		
218.9738		9341.78		
507.3084	1	7971.67	C30 H44 Na O5	M+
523.2797	1	10704.83		
537.3184	1	7292.68		
548.3337	1	6272.26		
553.2923	1	5397.99		

Formula Calculator Element Limits

Element	Min	Max
C	0	200
H	0	400
O	0	10
Na	1	1

Formula Calculator Results

Formula	CalculatedMass	Mz	Diff.(mDa)	Diff. (ppm)	DBE
C30 H44 Na O5	507.3086	507.3084	0.2	0.5	8.5

--- End Of Report ---

Figure S25. HRESIMS spectrum of **3**.

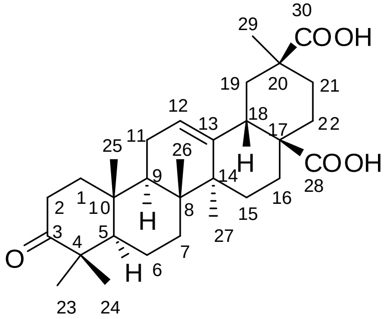

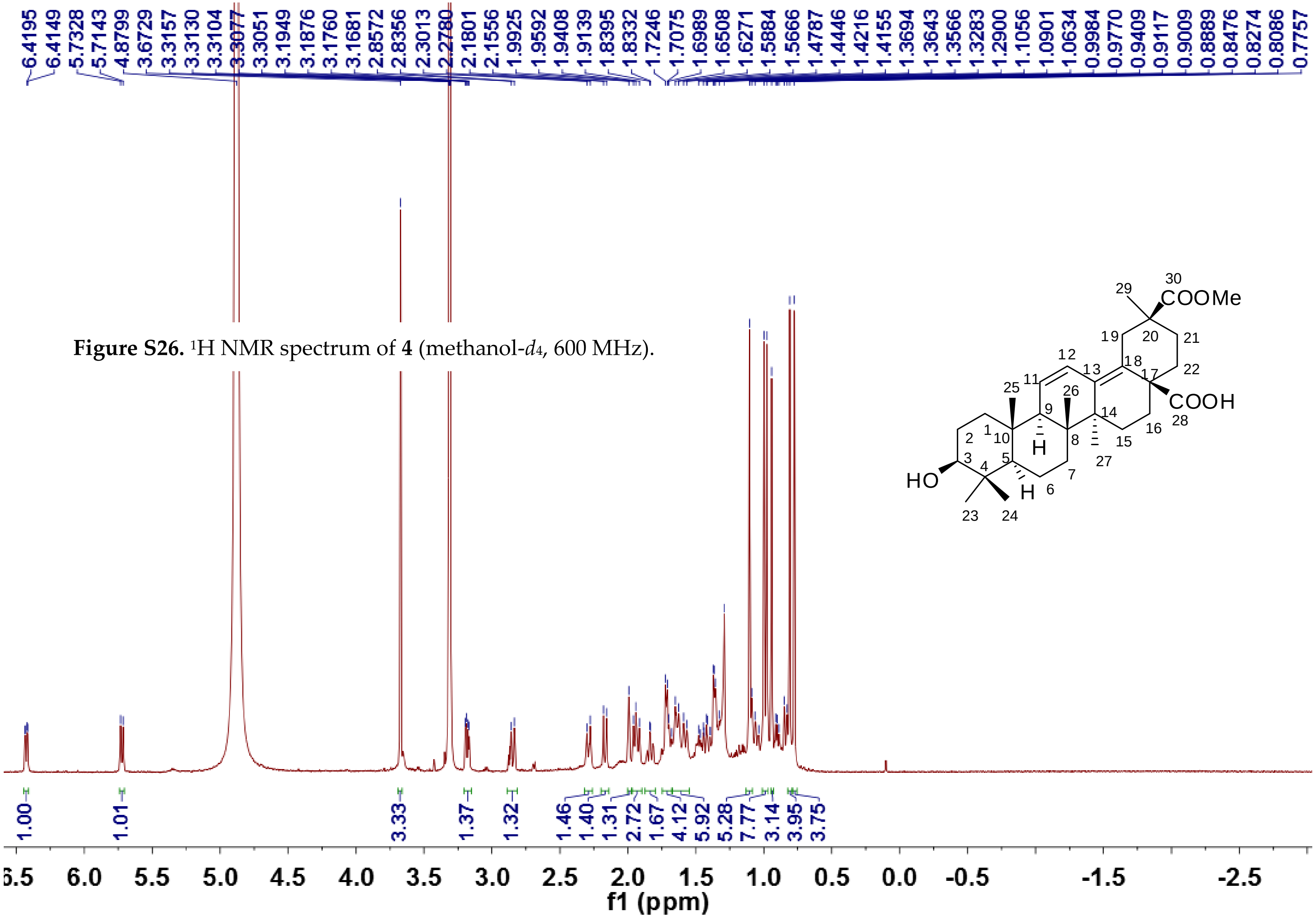

Figure S26. ^{1}H NMR spectrum of **4** (methanol-d_4, 600 MHz).

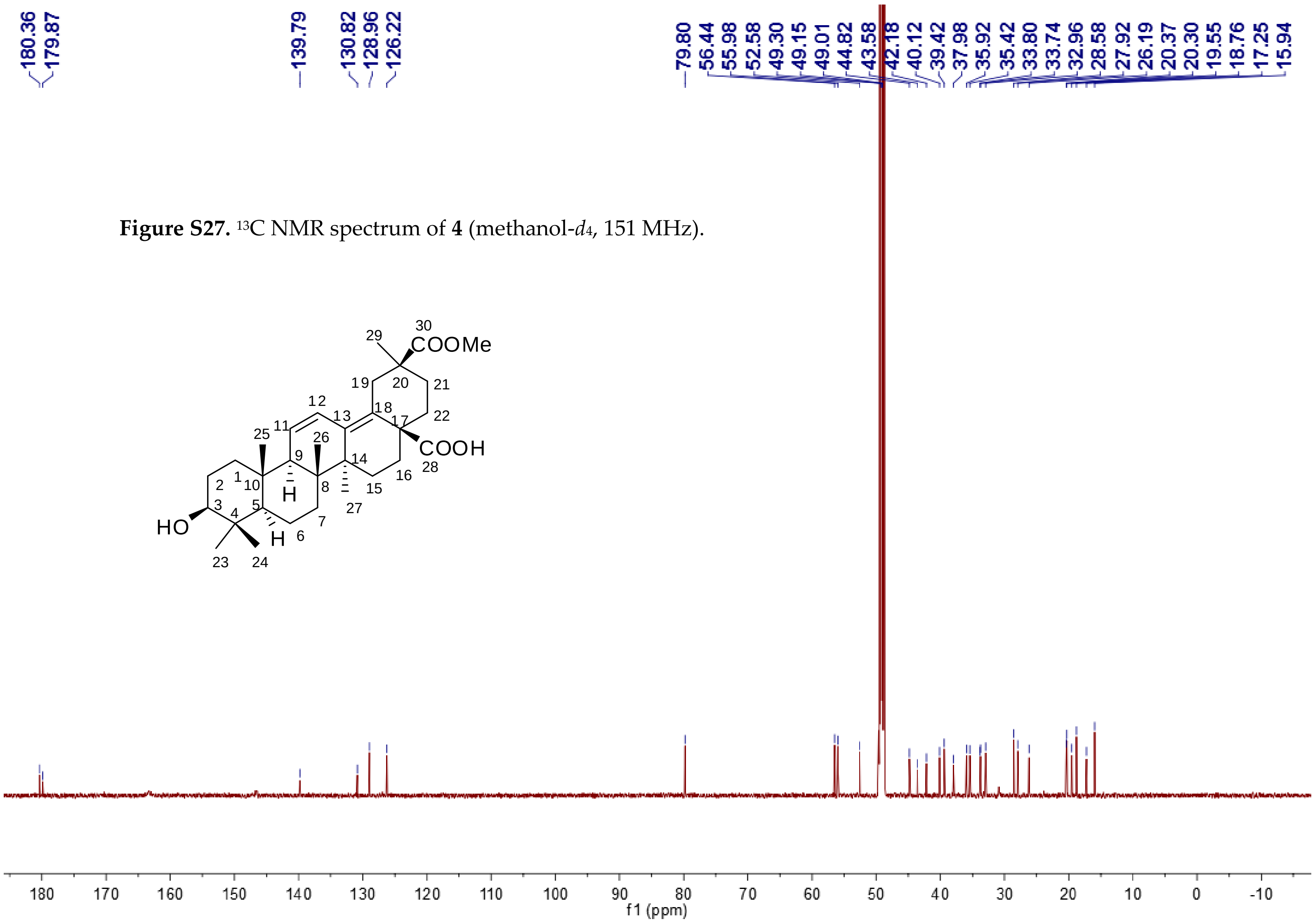

Figure S27. ^{13}C NMR spectrum of **4** (methanol-*d*4, 151 MHz).

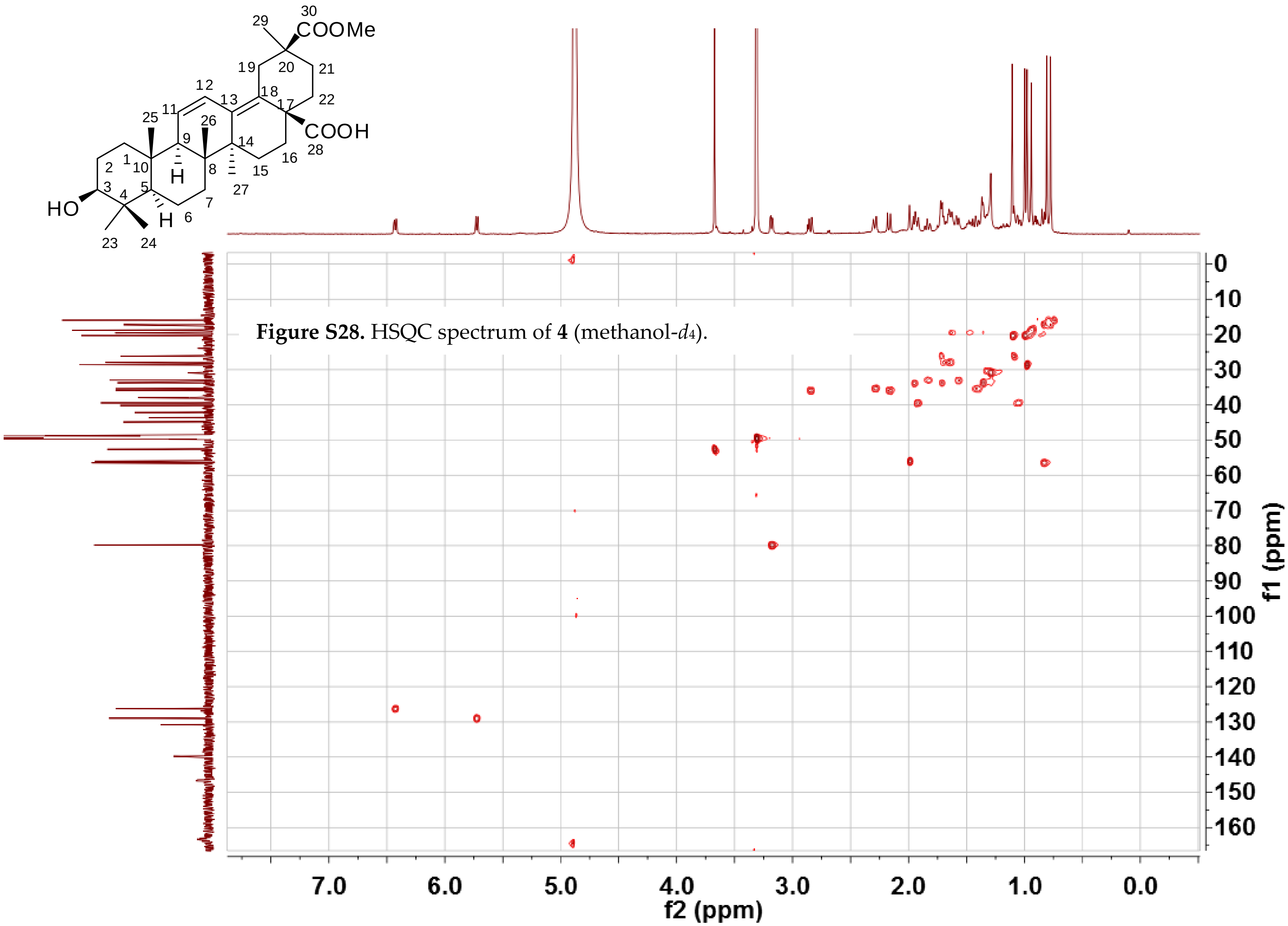

Figure S28. HSQC spectrum of **4** (methanol-*d*4).

Figure S29. ${}^1H$–${}^1H$ COSY spectrum of **4** (methanol-*d*$_4$).

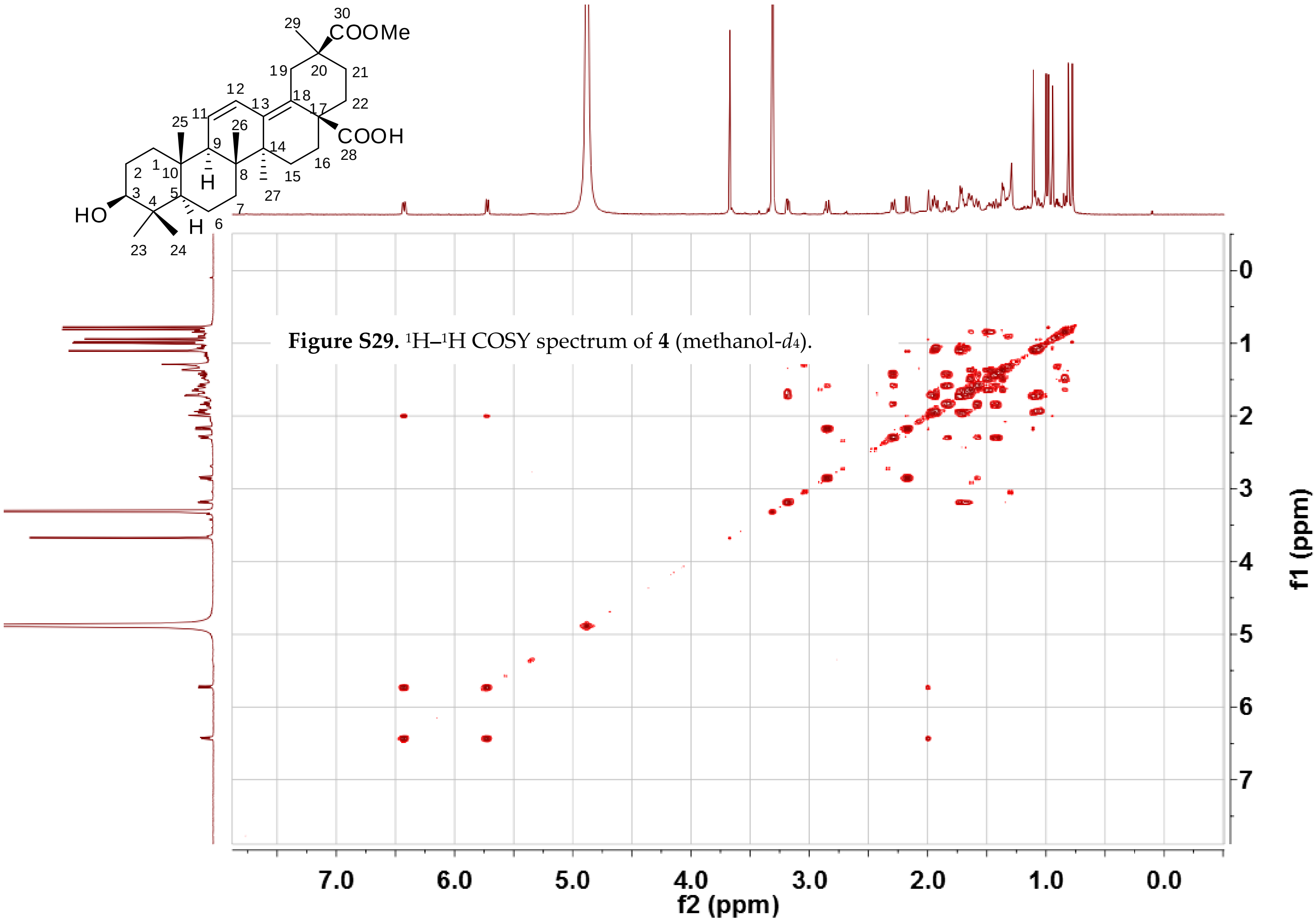

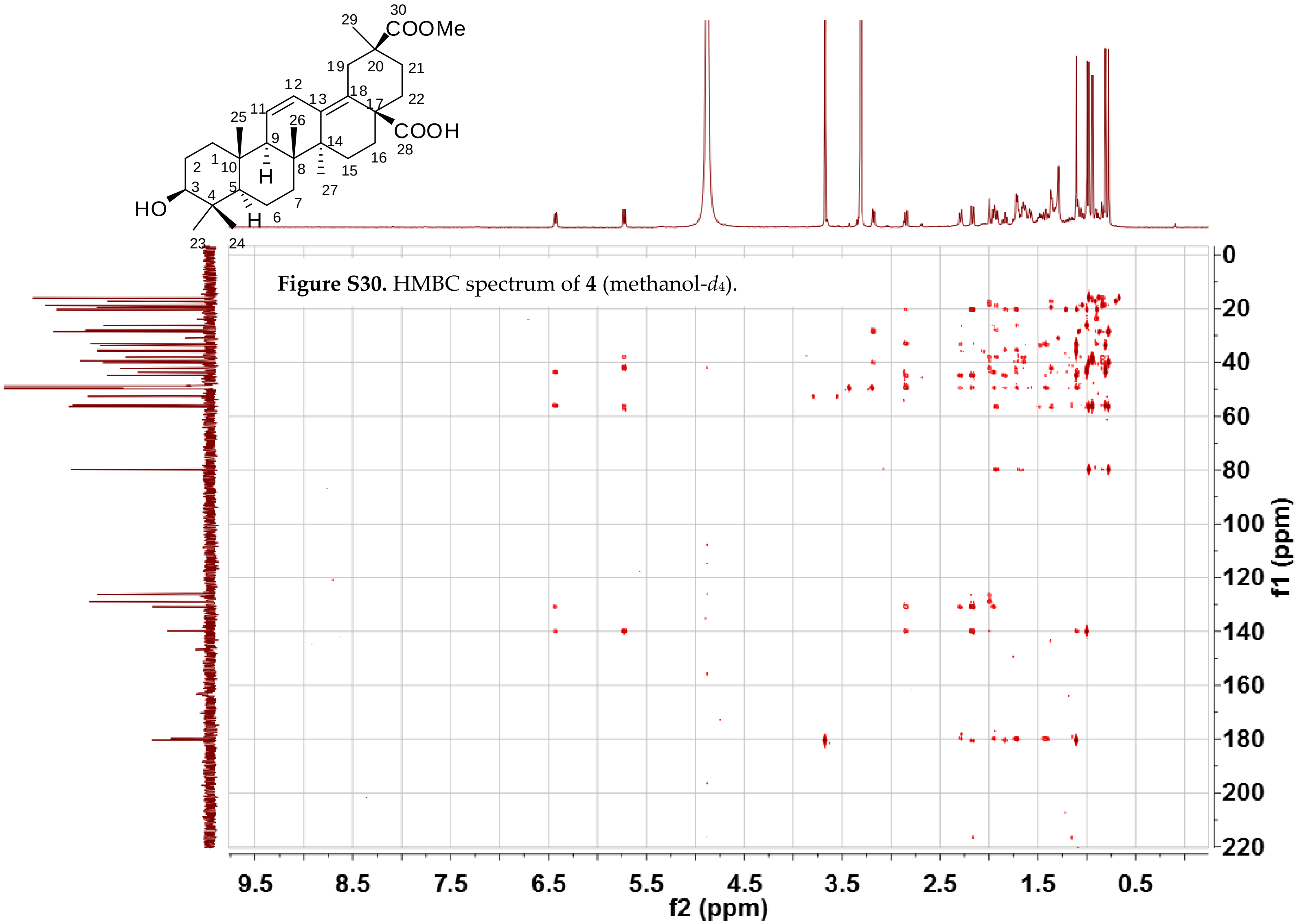

Figure S30. HMBC spectrum of **4** (methanol-d_4).

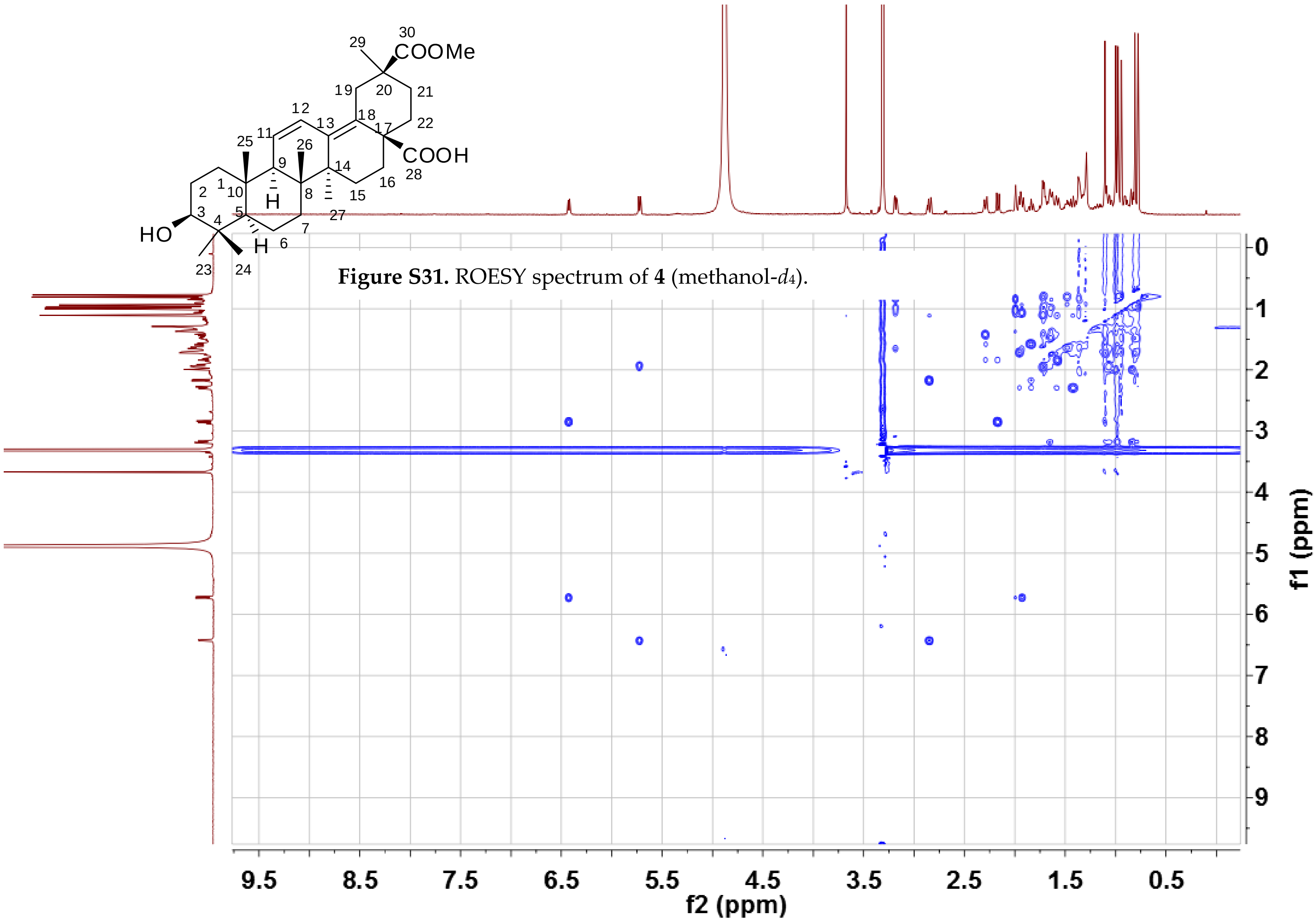

Figure S31. ROESY spectrum of **4** (methanol-d_4).

Figure S32. HRESIMS spectrum of **4**.

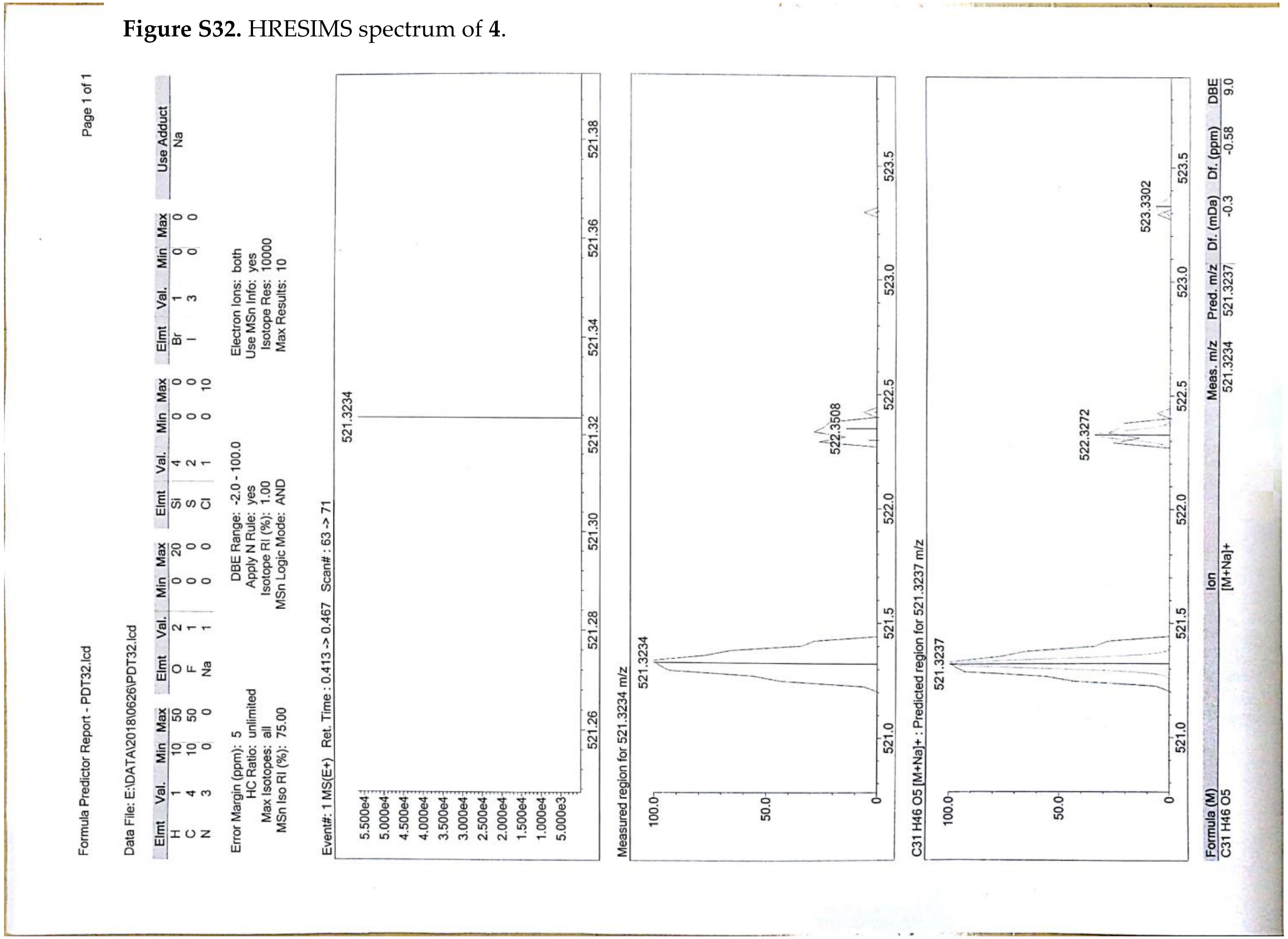

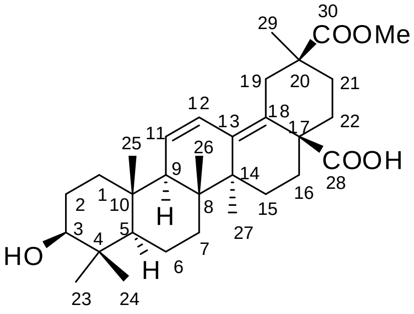

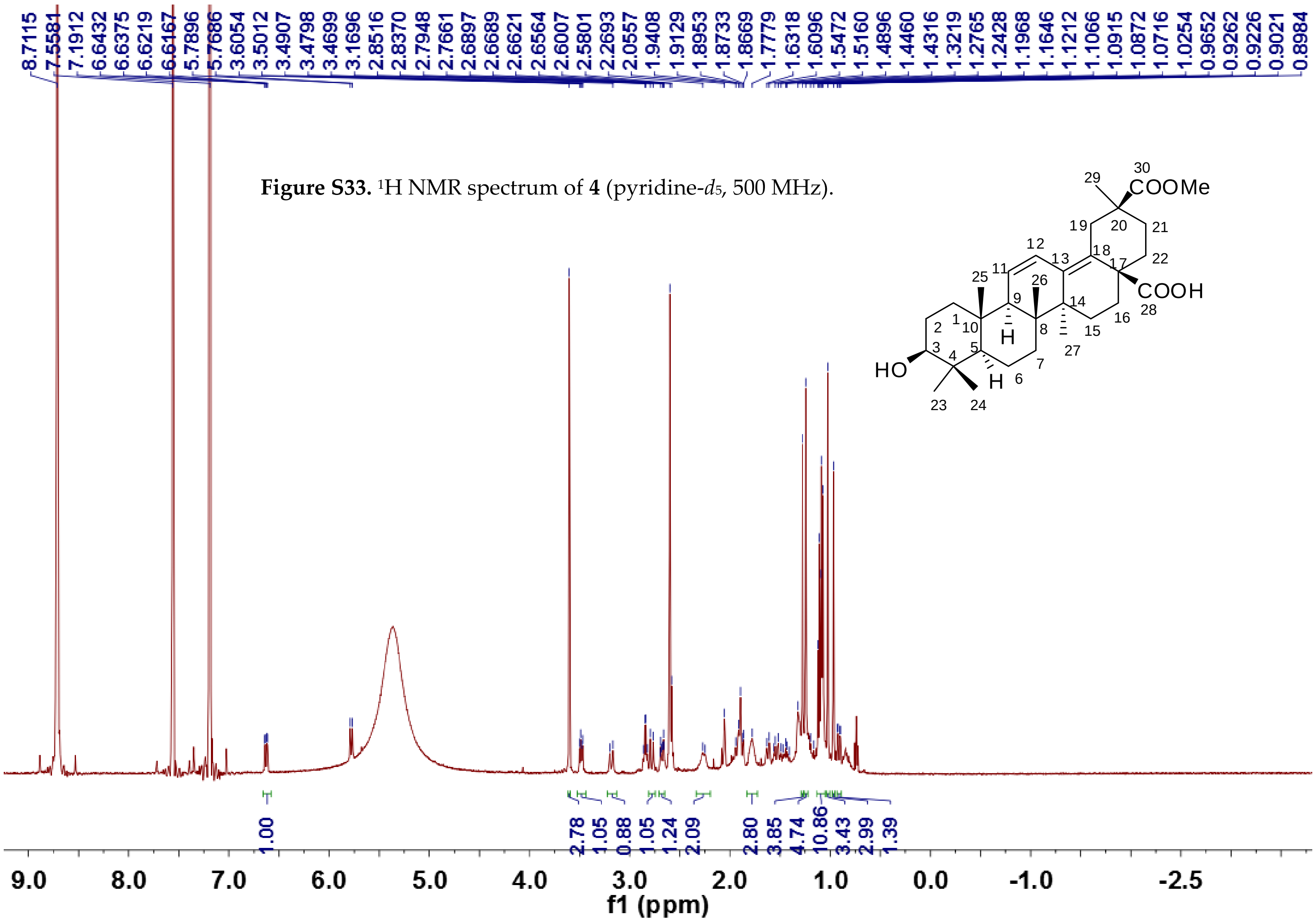

Figure S33. ^{1}H NMR spectrum of **4** (pyridine-d_5, 500 MHz).

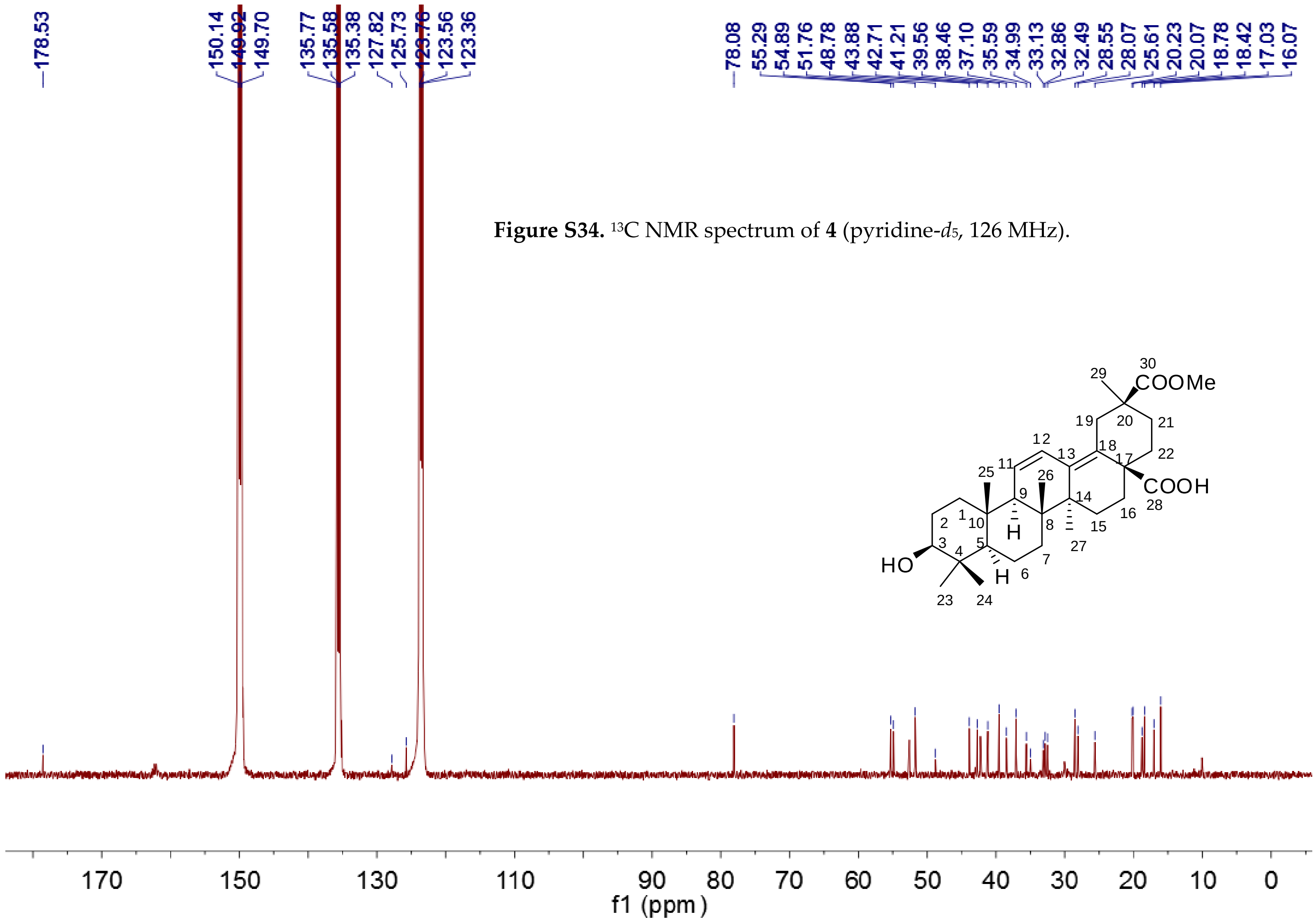

Figure S34. ^{13}C NMR spectrum of **4** (pyridine-d_5, 126 MHz).

Figure S35. HSQC spectrum of **4** (pyridine-*d*5).

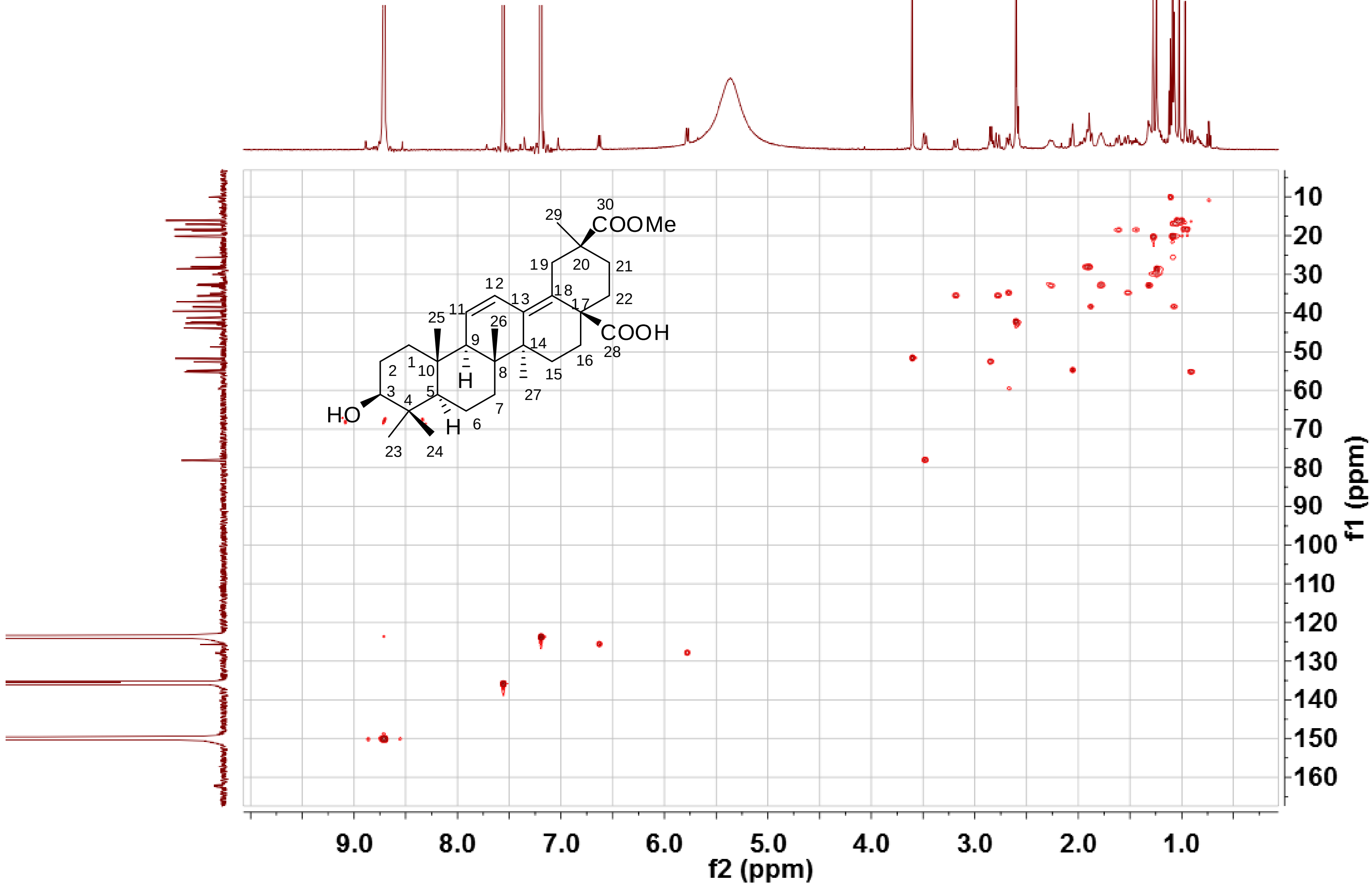

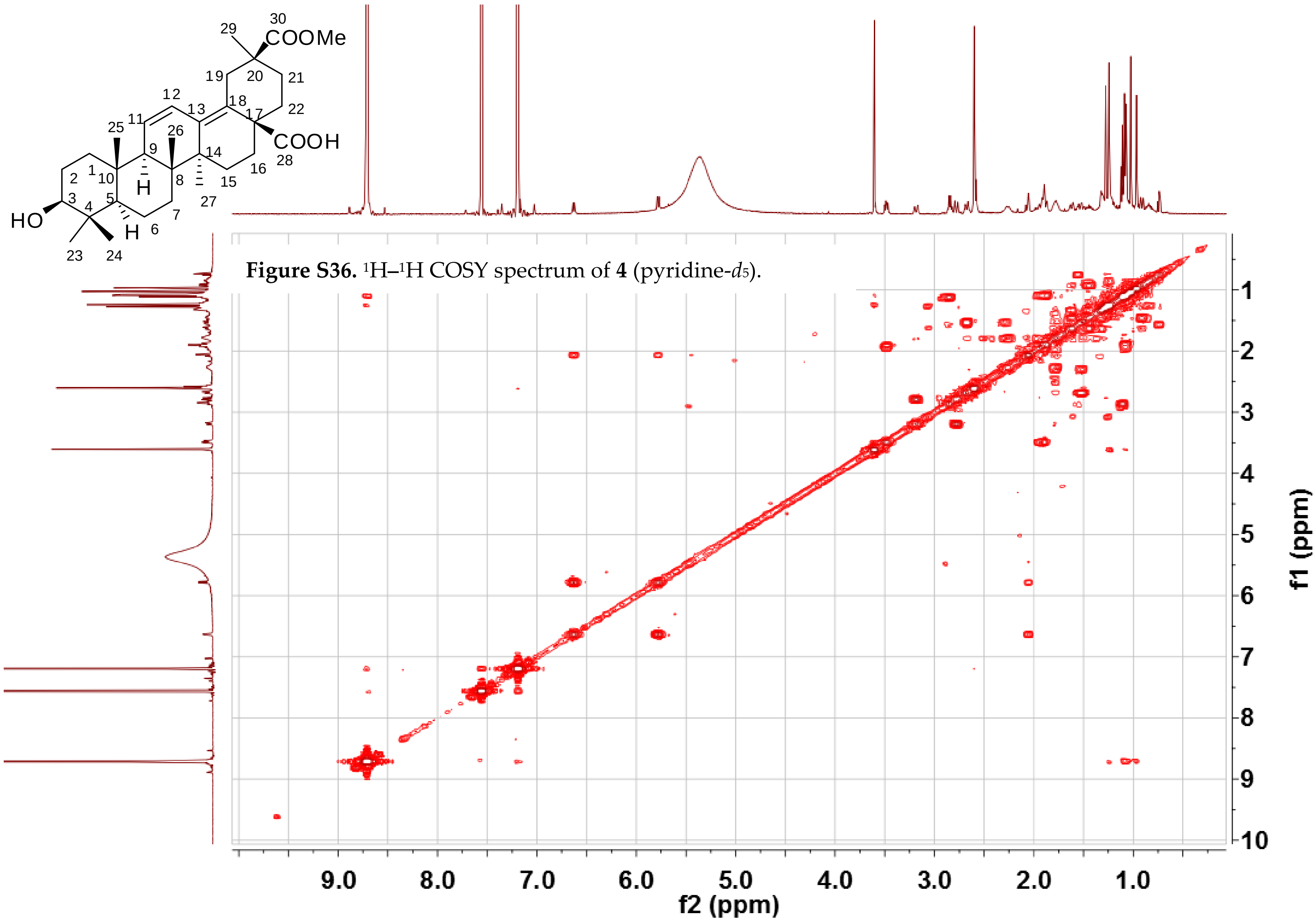

Figure S36. ^{1}H–^{1}H COSY spectrum of **4** (pyridine-d_5).

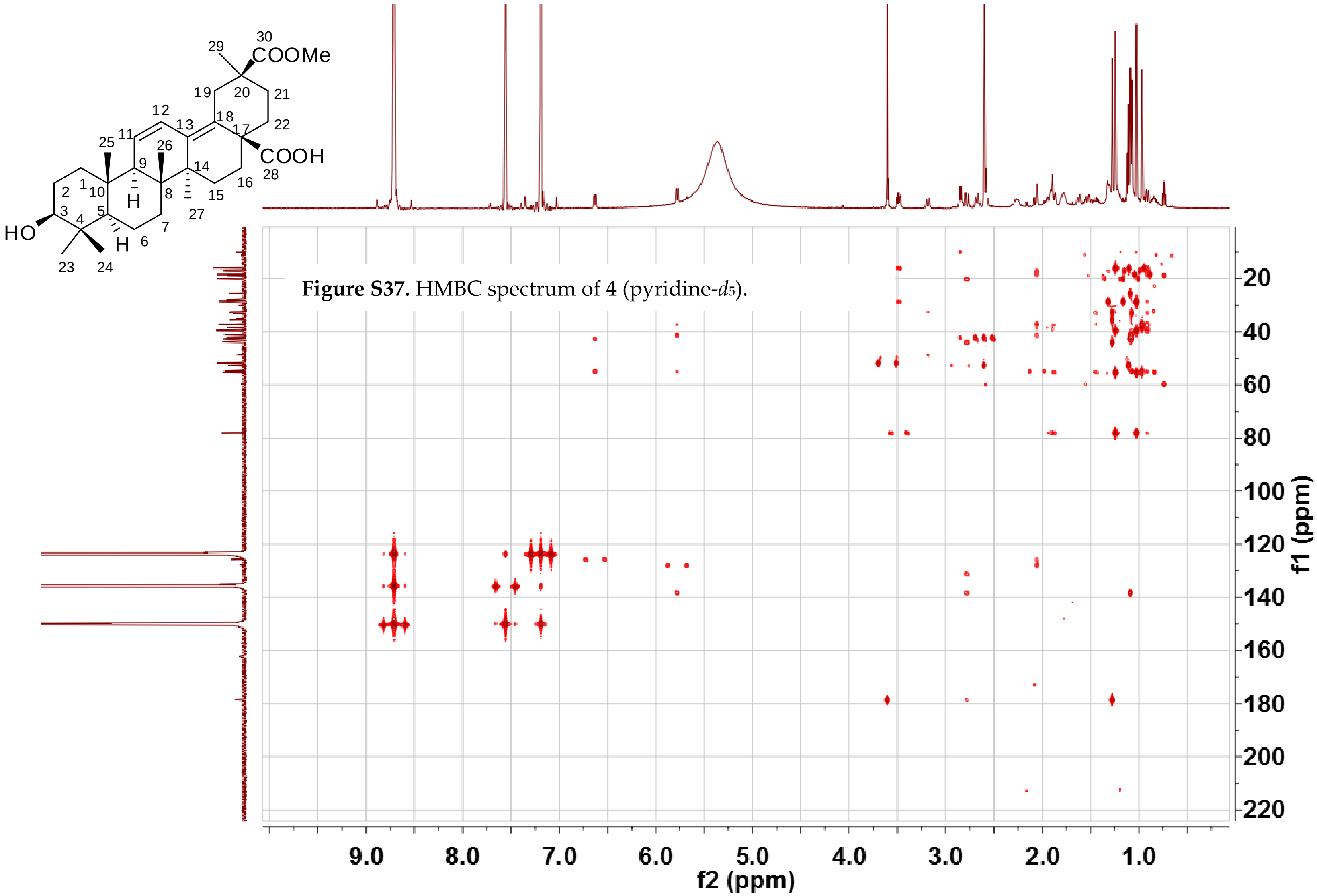

Figure S37. HMBC spectrum of **4** (pyridine-d_5).

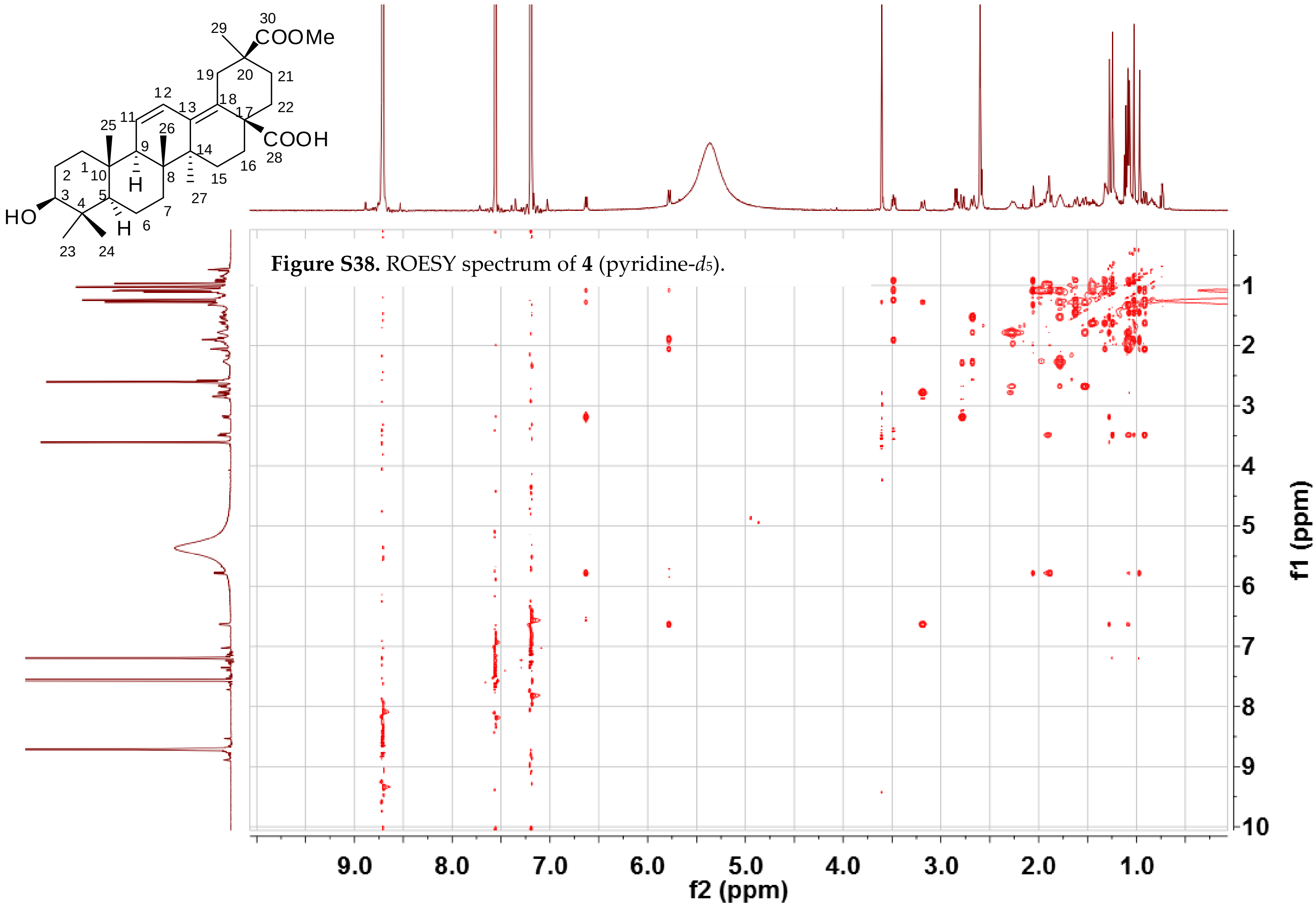

Figure S38. ROESY spectrum of **4** (pyridine-*d*5).

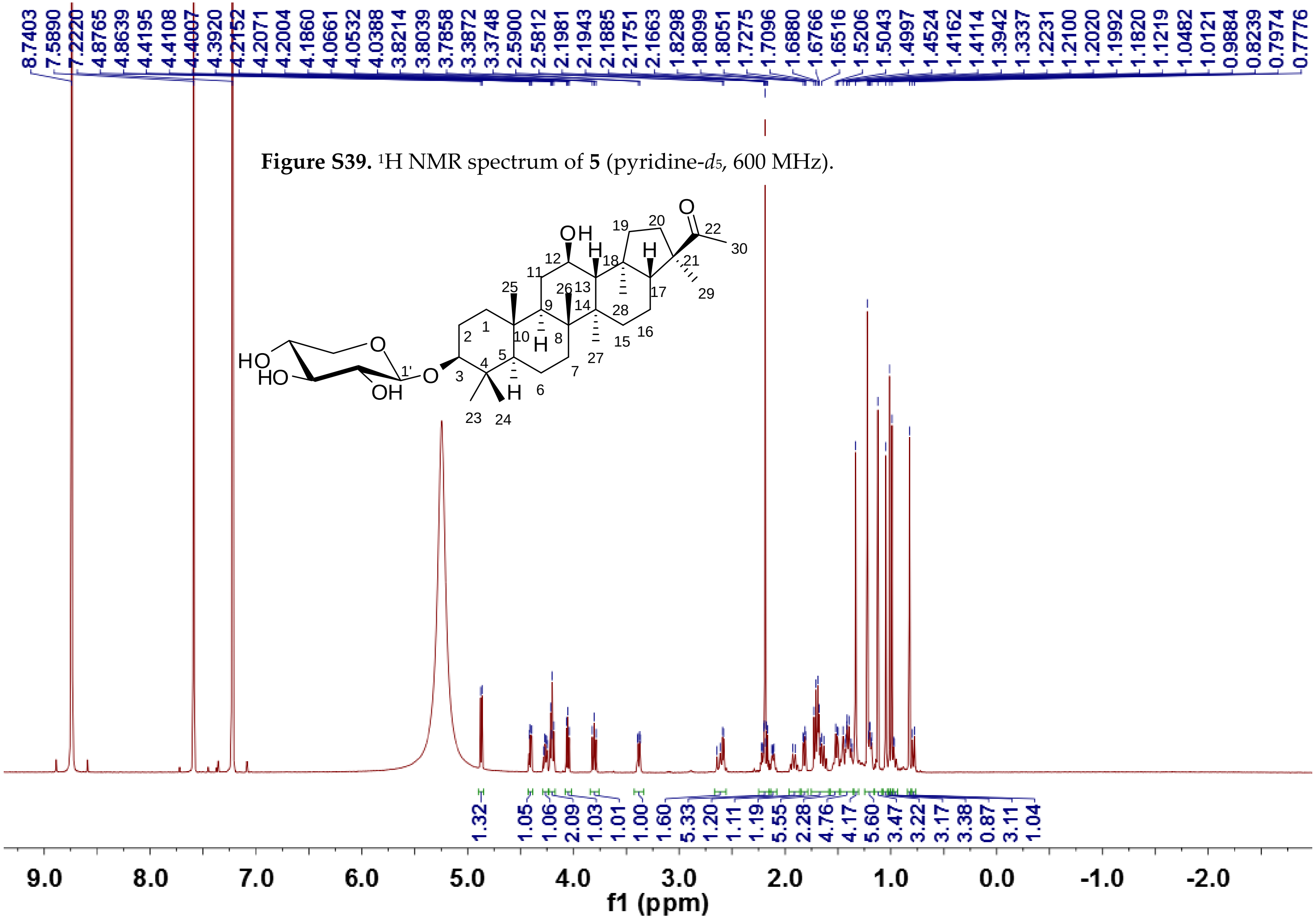

Figure S39. ^{1}H NMR spectrum of **5** (pyridine-d_5, 600 MHz).

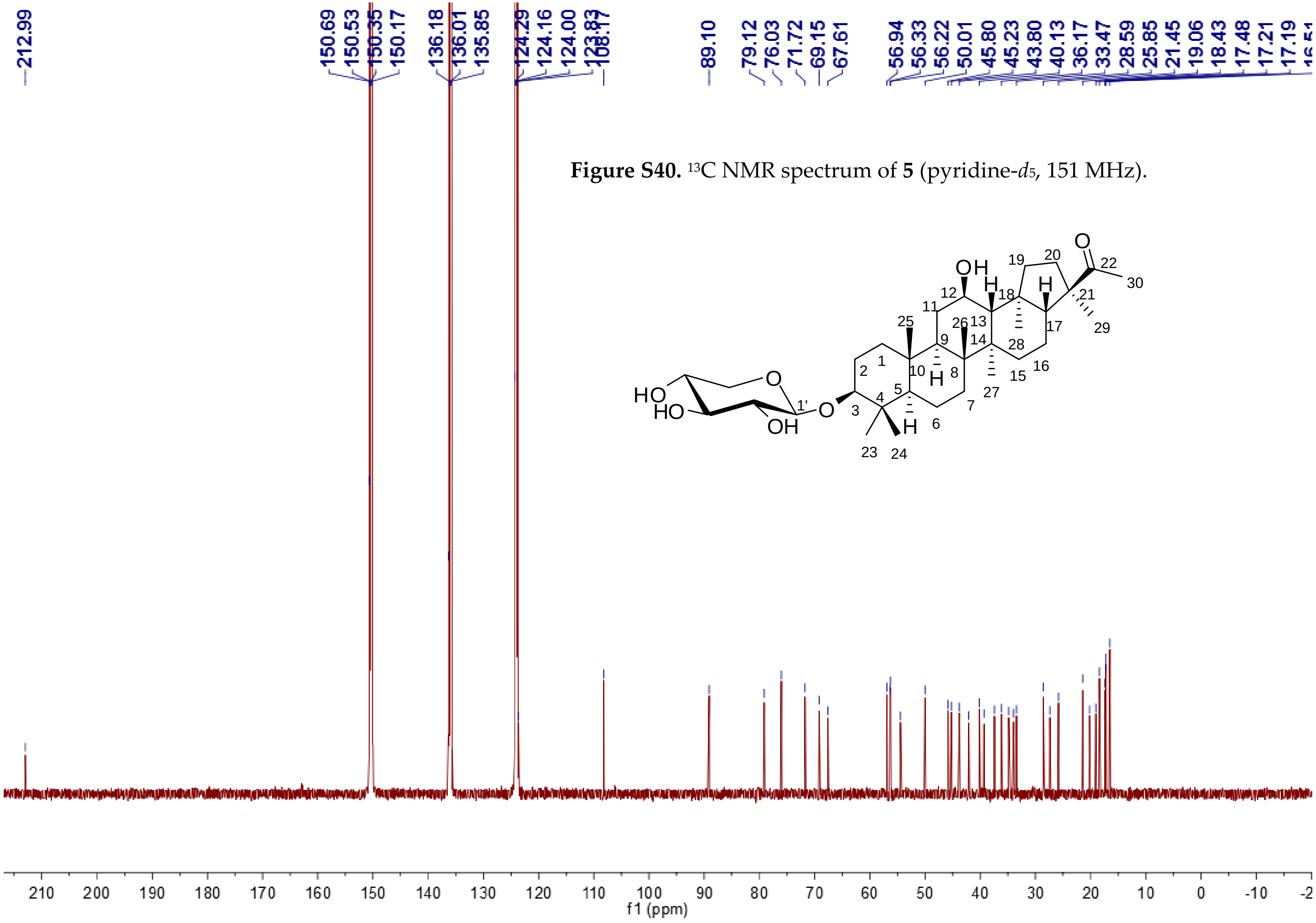

Figure S40. ^{13}C NMR spectrum of **5** (pyridine-d_5, 151 MHz).

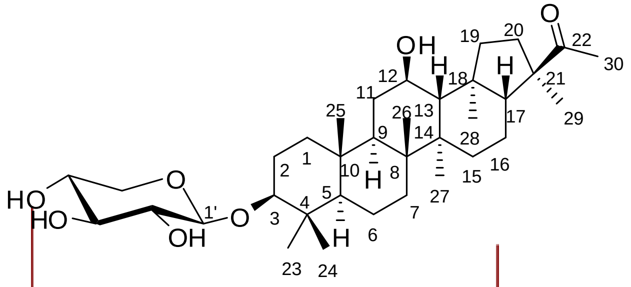

Figure S41. HSQC spectrum of **5**.

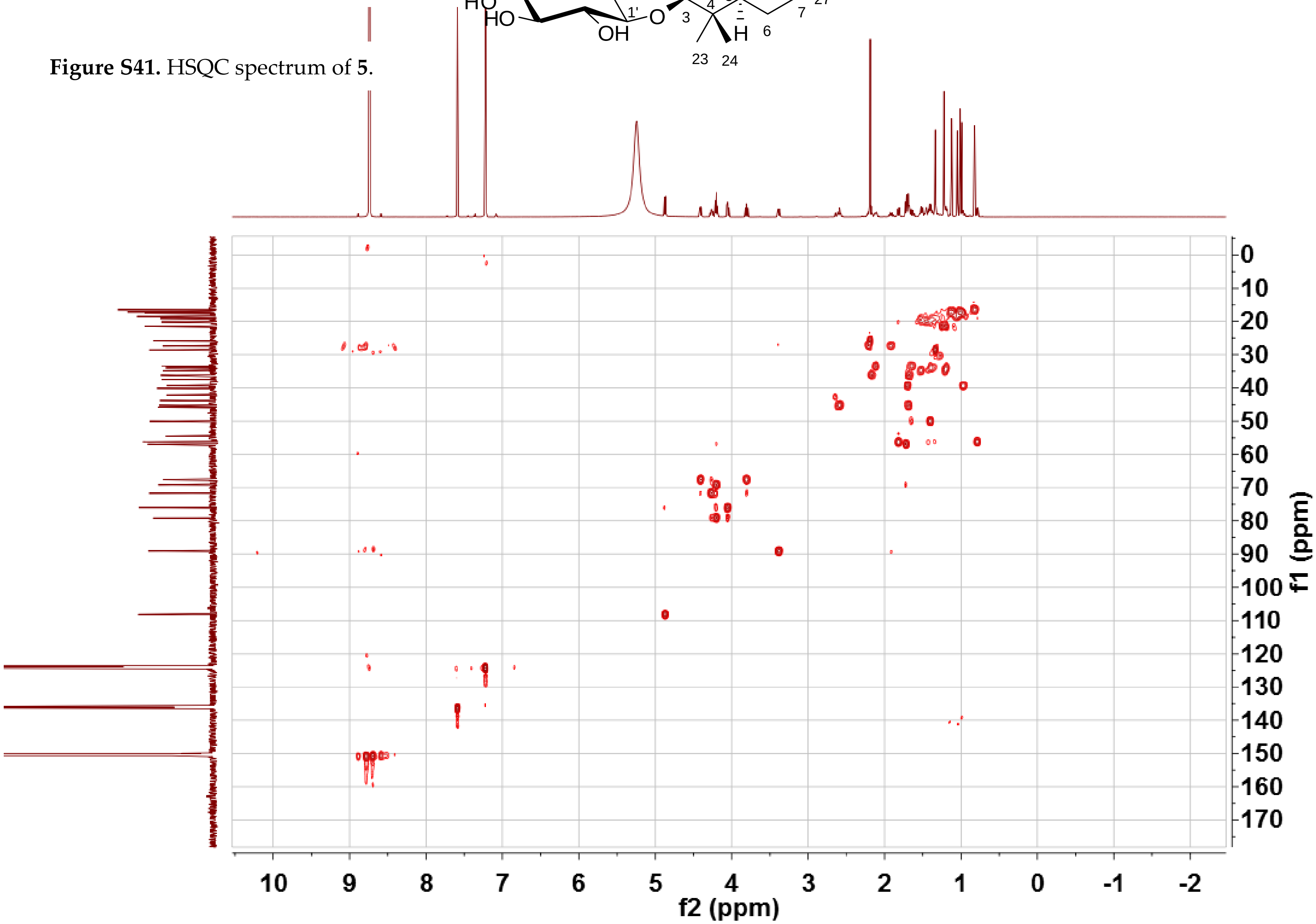

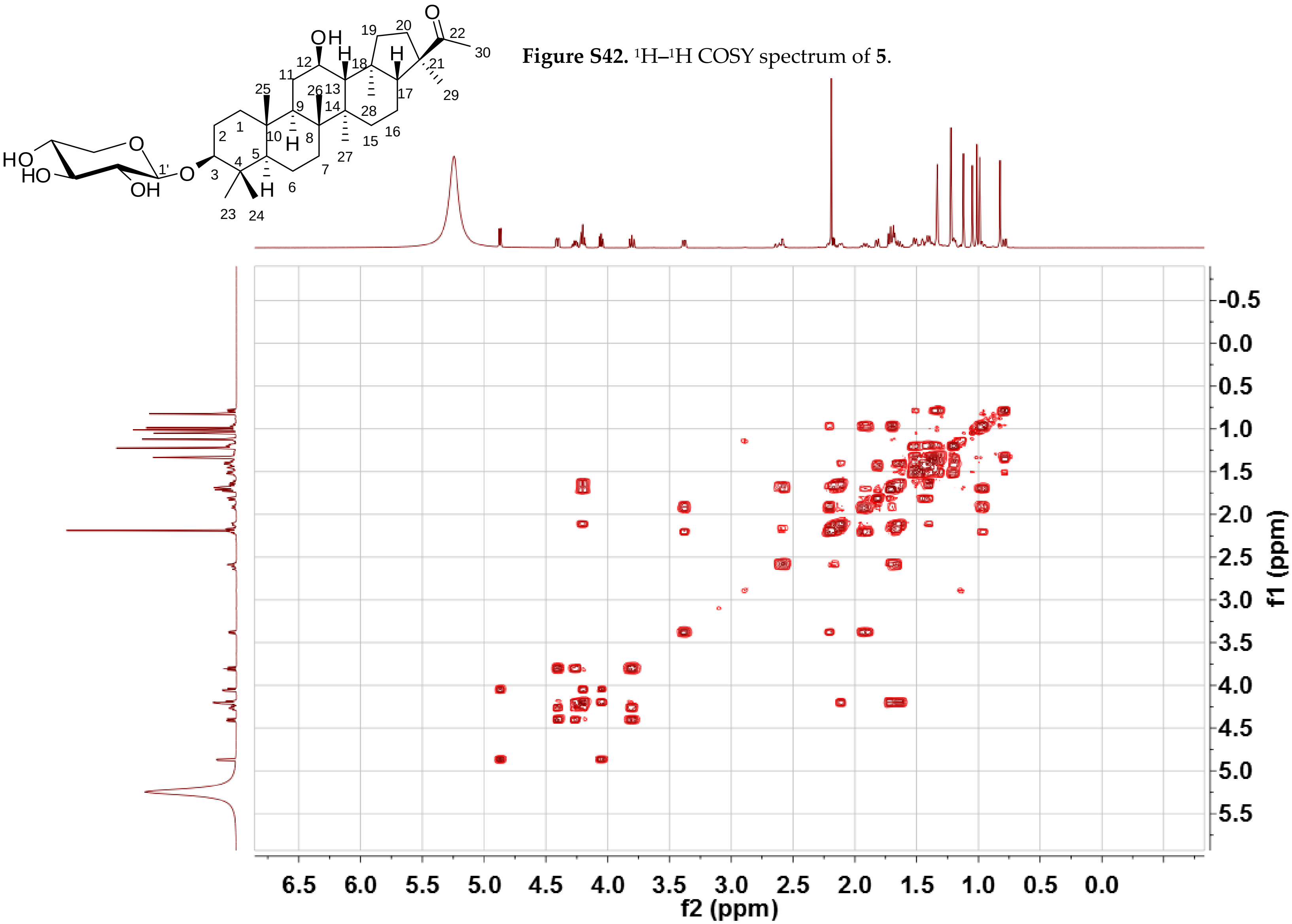

Figure S42. ^{1}H–^{1}H COSY spectrum of **5**.

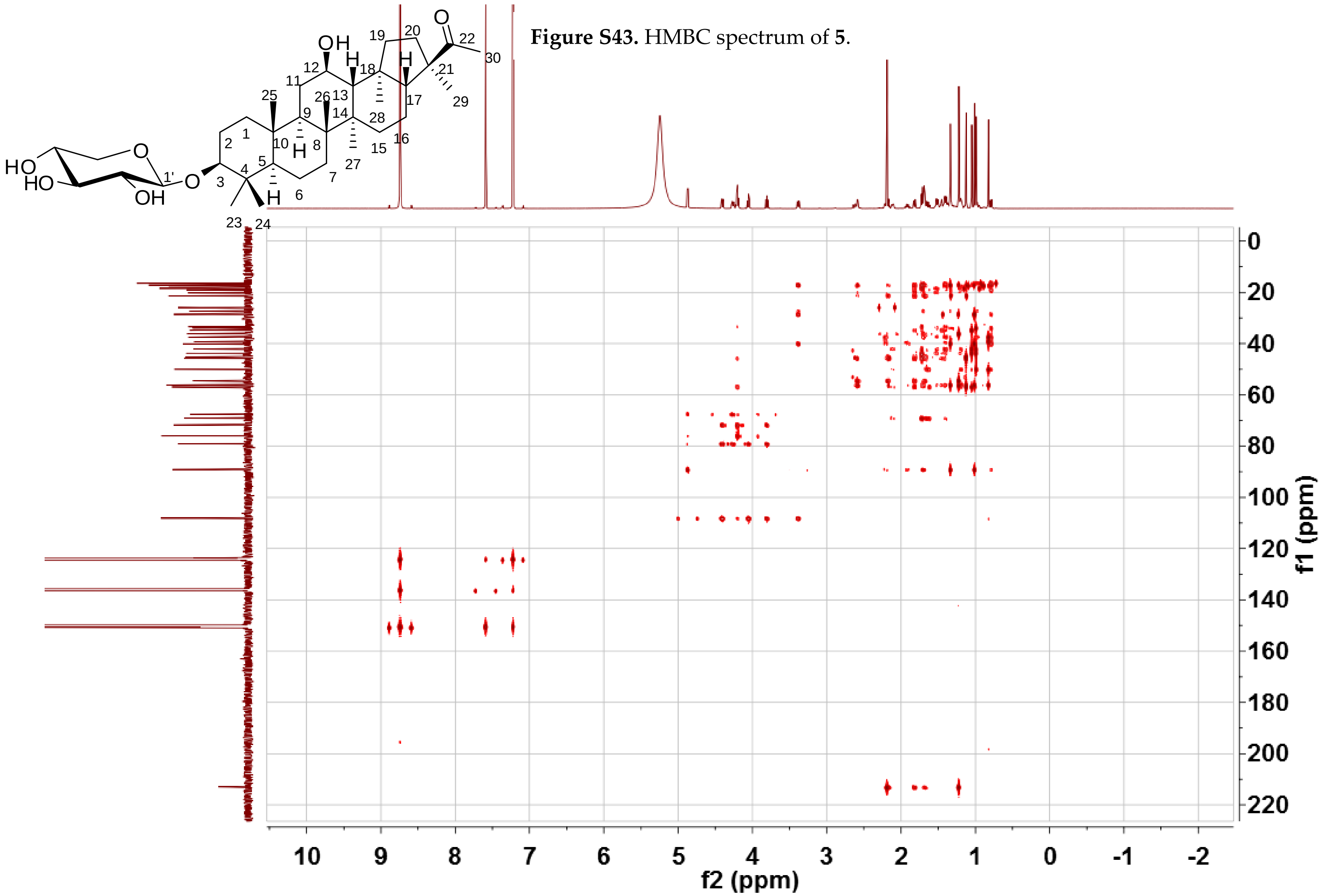

Figure S43. HMBC spectrum of **5**.

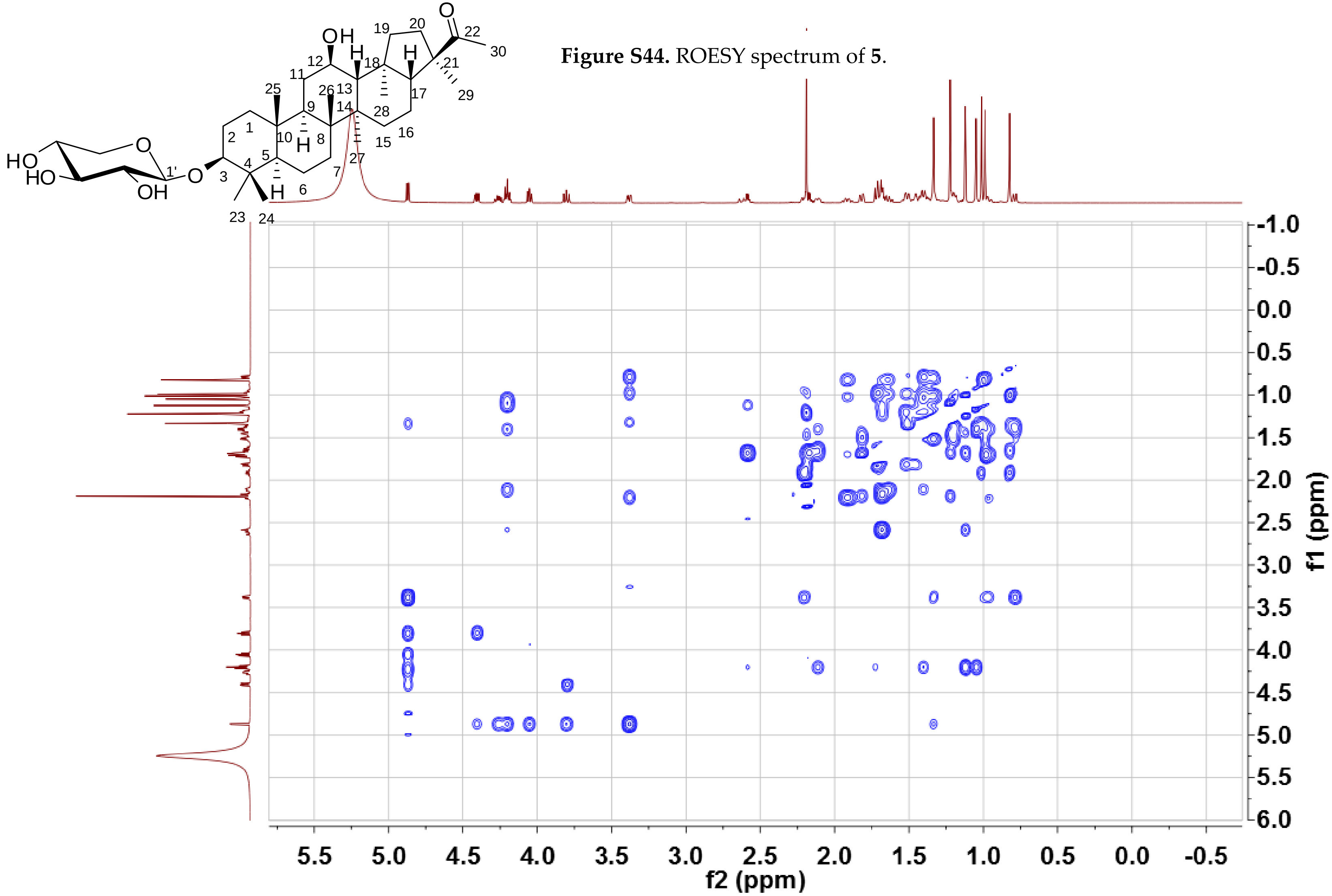

Figure S44. ROESY spectrum of **5**.

**Data Filename**	180827ESIA1.d	**Sample Name**	pdt54b
**Sample Type**	Sample	**Position**	
**Instrument Name**	Agilent G6230 TOF MS	**User Name**	KIB
**Acq Method**	ESI.m	**Acquired Time**	8/28/2018 9:54:22 AM
**IRM Calibration Status**	Success	**DA Method**	ESI.m
**Comment**			

Sample Group **Info.**

Acquisition SW Version 6200 series TOF/6500 series Q-TOF B.05.01 (B5125.2)

User Spectra

Fragmentor Voltage	Collision Energy	Ionization Mode
200	0	ESI

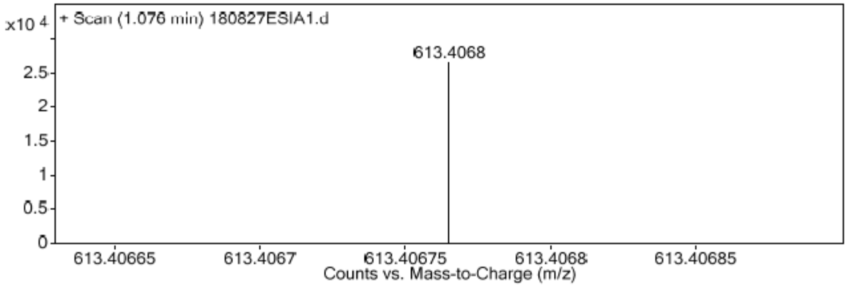

Peak List

m/z	z	Abund	Formula	Ion
102.1278	1	67271.03		
111.1809		41545.32		
112.1872	1	204281.06		
144.2428		42565.64		
145.2492	1	218638.56		
146.2522	1	19284.63		
152.2175	1	23178.26		
368.4239	1	23795.48		
458.4712	1	25047.8		
613.4068	1	26485.34	C35 H58 Na O7	M+

Formula Calculator Element Limits

Element	Min	Max
C	0	200
H	0	400
O	3	10
Na	1	1

Formula Calculator Results

Formula	CalculatedMass	Mz	Diff.(mDa)	Diff. (ppm)	DBE
C35 H58 Na O7	613.4080	613.4068	1.2	2.0	6.5

--- End Of Report ---

Figure S45. HRESIMS spectrum of **5**.

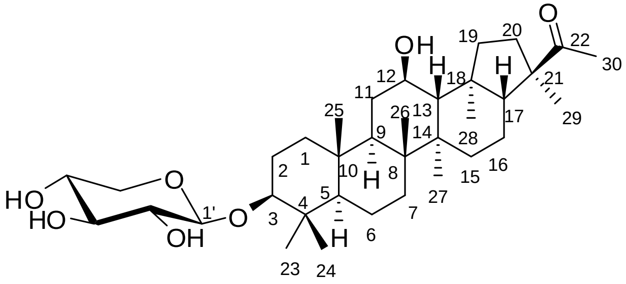

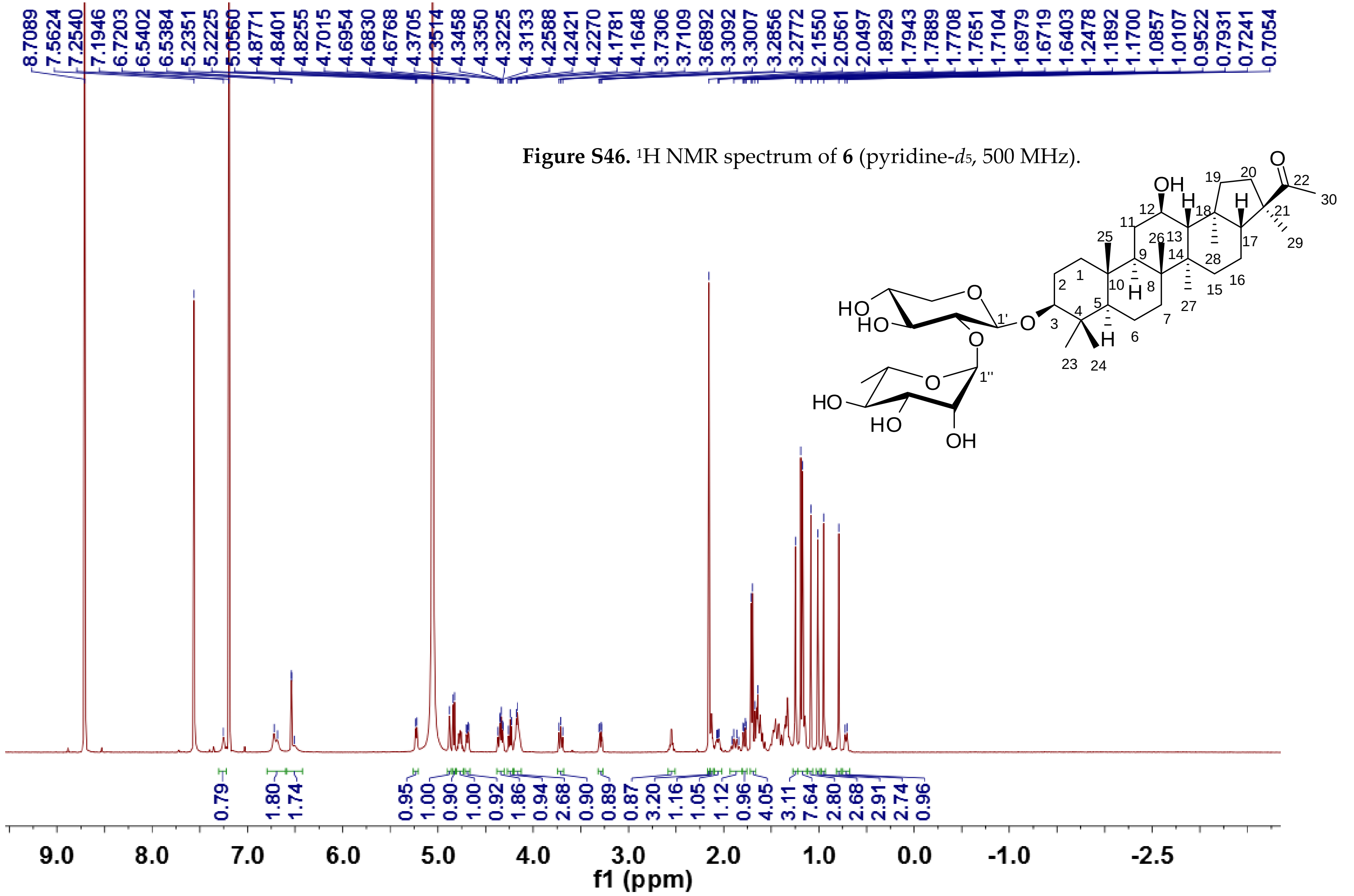

Figure S46. ^{1}H NMR spectrum of **6** (pyridine-d_5, 500 MHz).

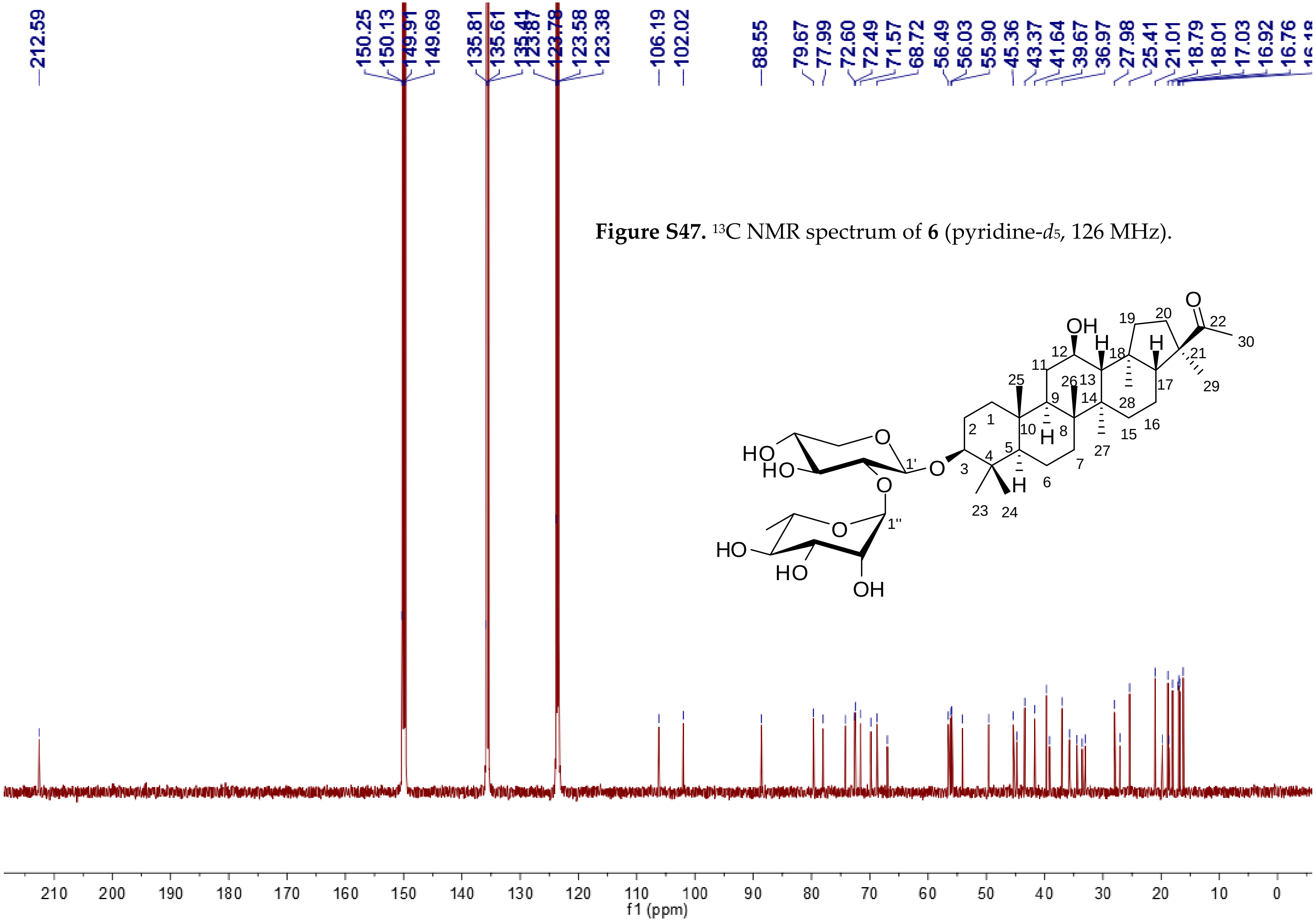

Figure S47. ^{13}C NMR spectrum of **6** (pyridine-*d*$_5$, 126 MHz).

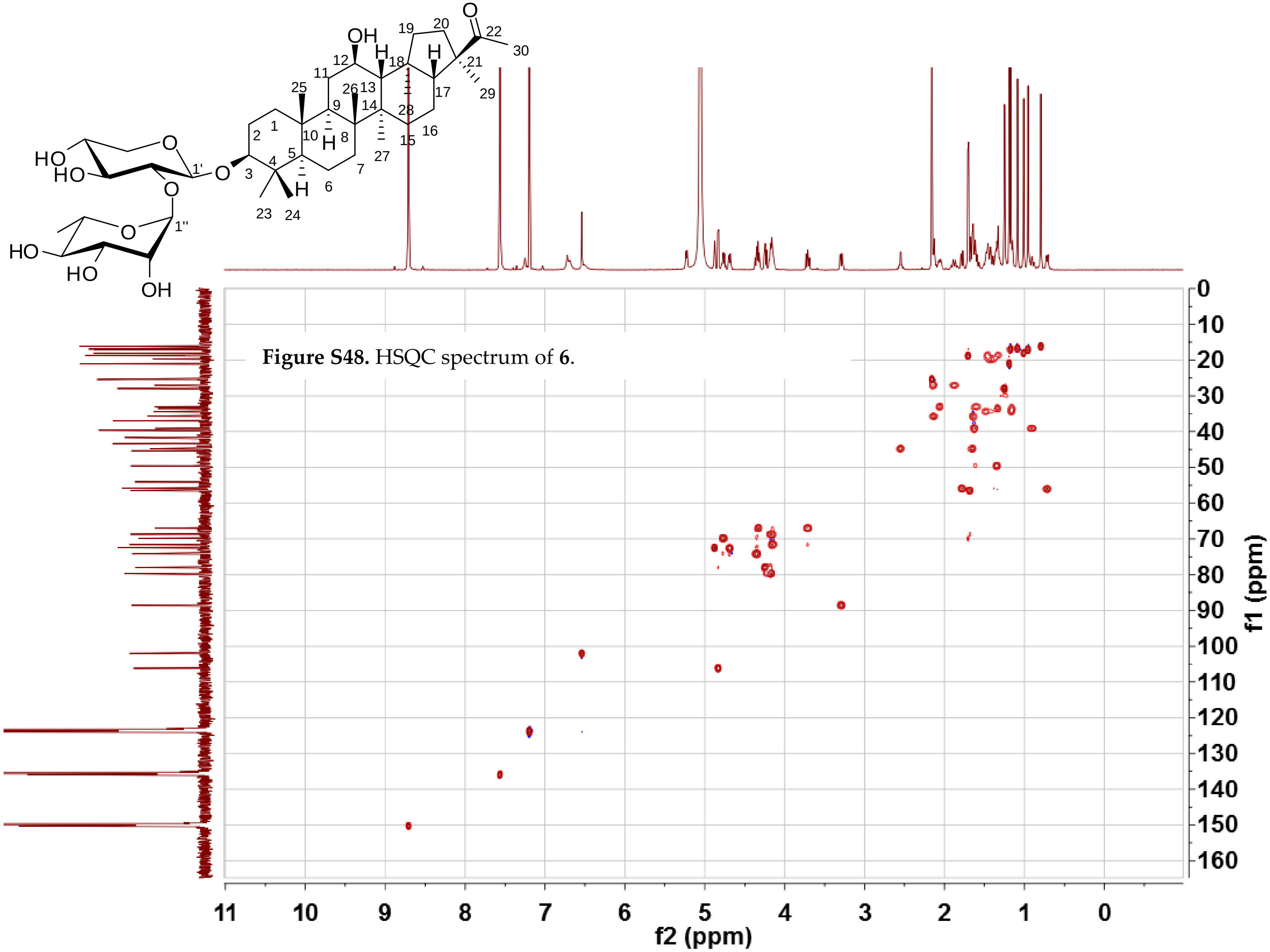

Figure S48. HSQC spectrum of **6**.

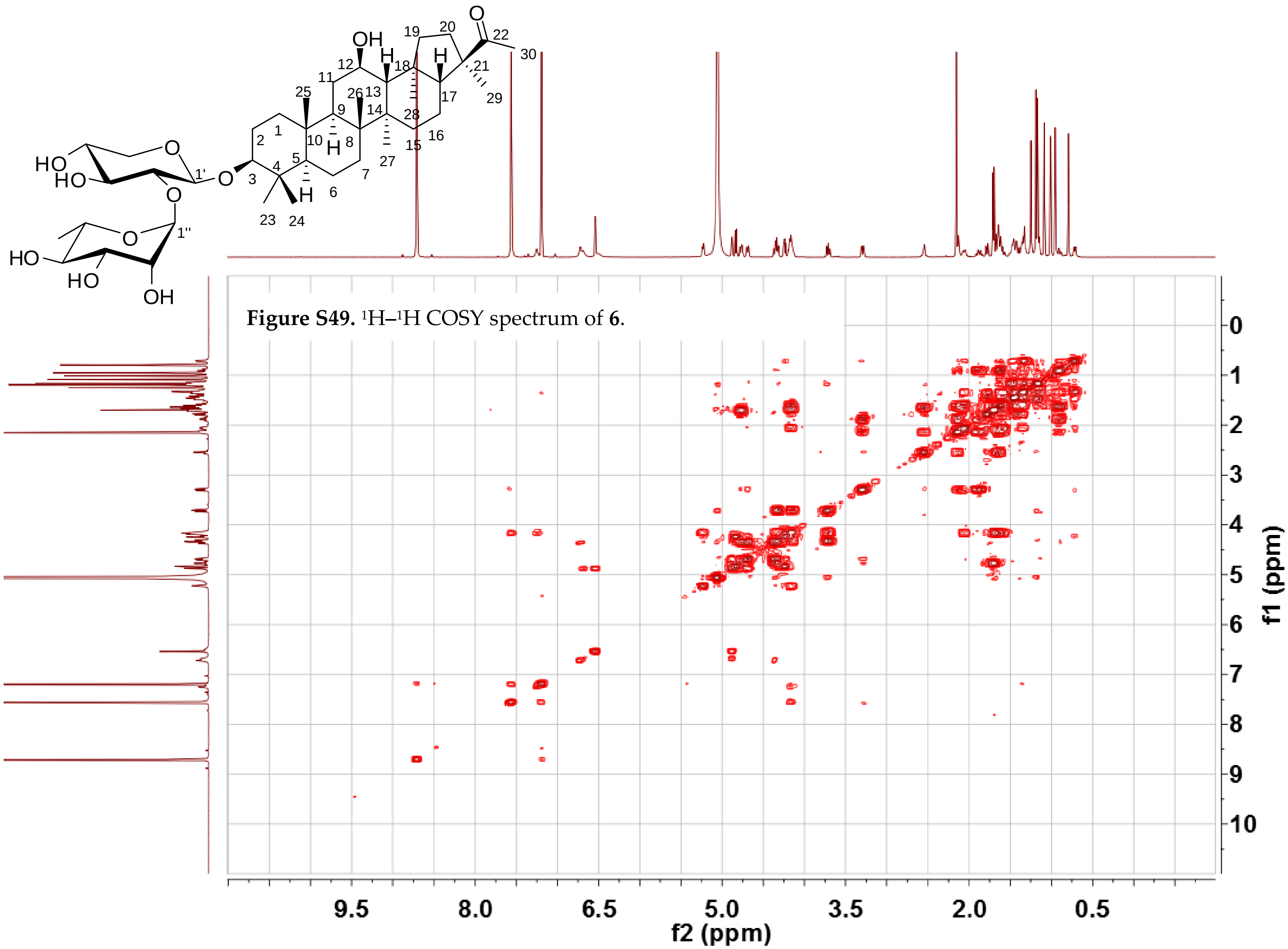

Figure S49. ¹H–¹H COSY spectrum of **6**.

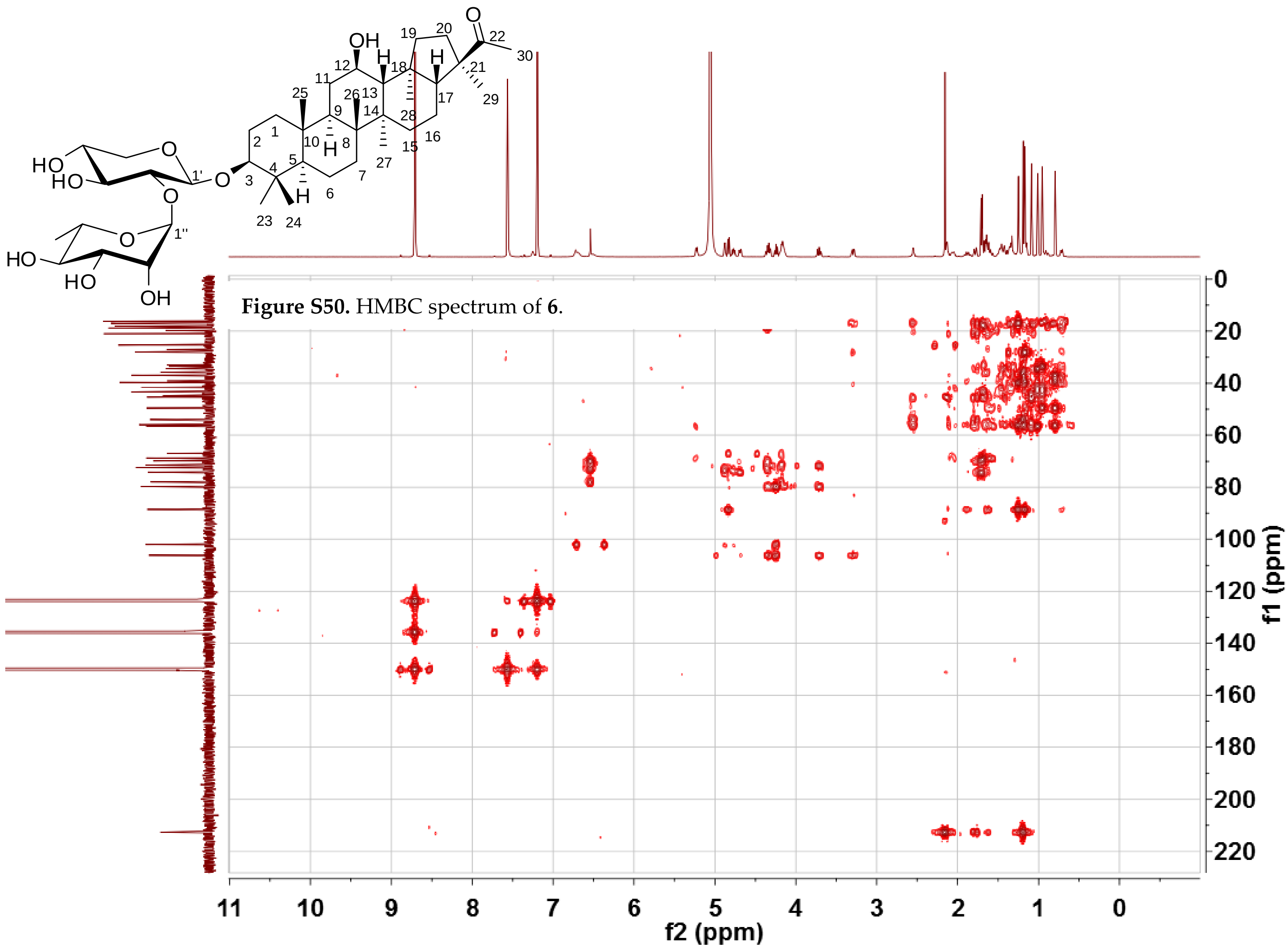

Figure S50. HMBC spectrum of **6**.

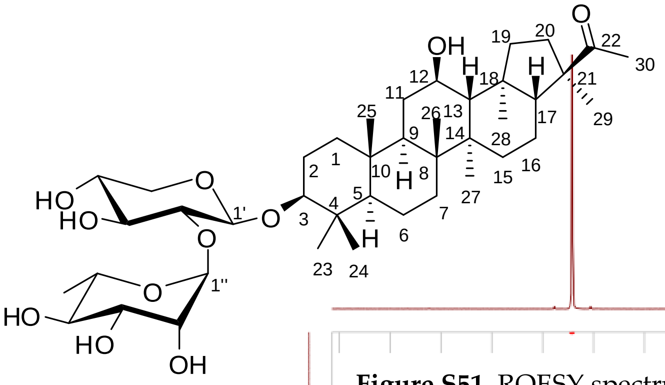

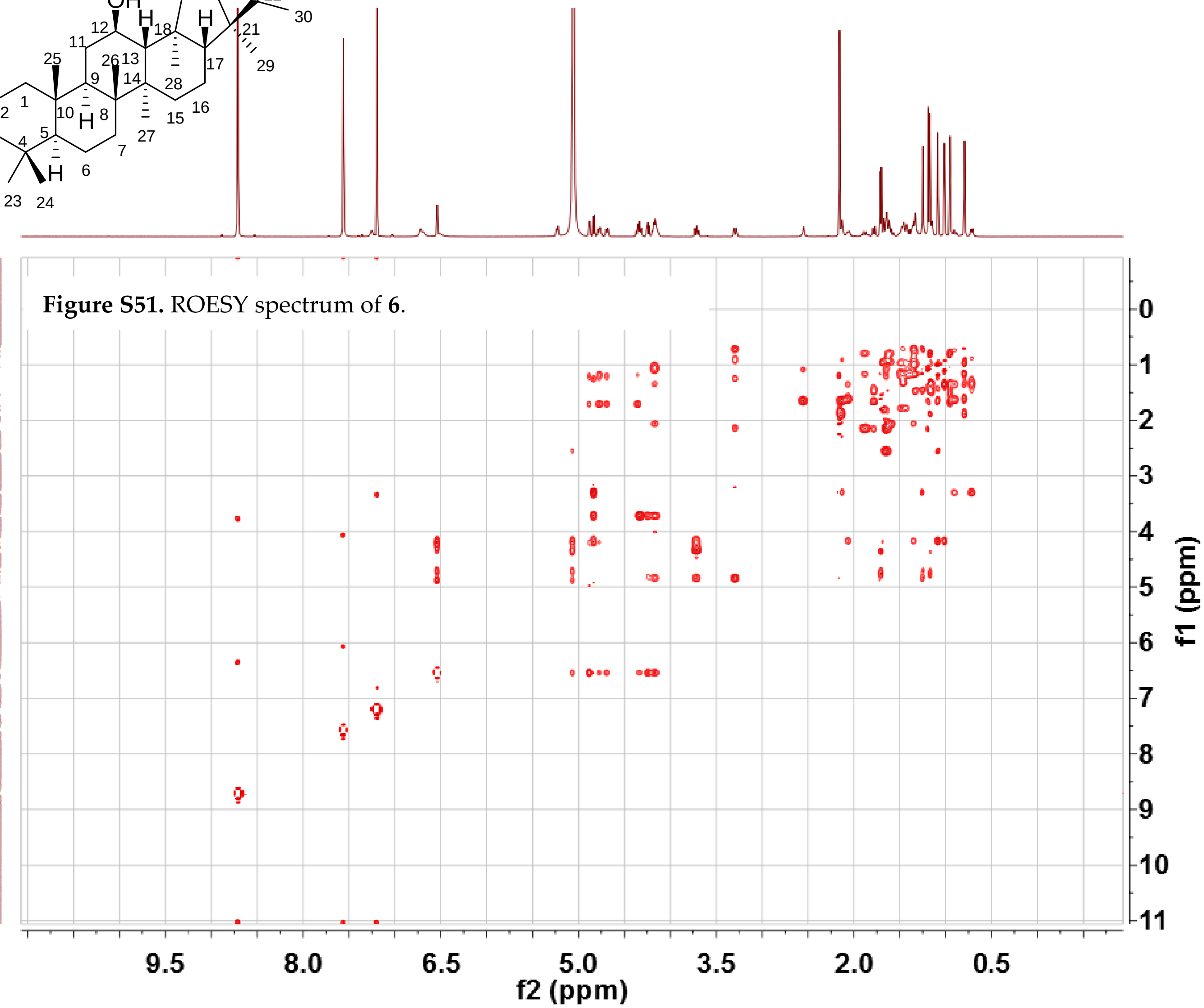

Figure S51. ROESY spectrum of **6**.

**Data Filename**	180828ESIA4.d	**Sample Name**	pdt52
**Sample Type**	Sample	**Position**	
**Instrument Name**	Agilent G6230 TOF MS	**User Name**	KIB
**Acq Method**	ESI.m	**Acquired Time**	8/28/2018 9:57:33 AM
**IRM Calibration Status**	Success	**DA Method**	ESI.m
**Comment**			

**Sample Group**		**Info.**
**Acquisition SW Version**	6200 series TOF/6500 series Q-TOF B.05.01 (B5125.2)	

User Spectra

Fragmentor Voltage	Collision Energy	Ionization Mode
200	0	ESI

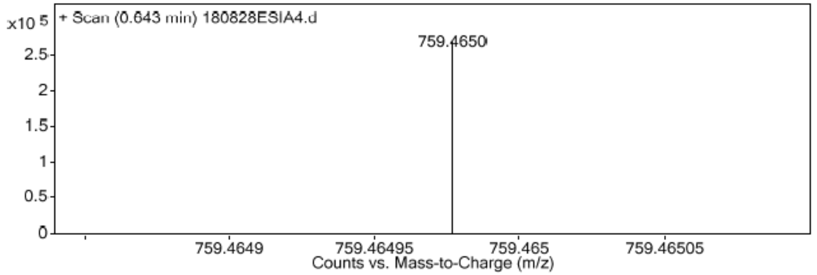

Peak List

m/z	z	Abund	Formula	Ion
274.2733	1	108594.22		
318.2996	1	104633.04		
340.2813	1	47140.76		
362.3255	1	30291.13		
384.3068	1	25858.5		
759.465	1	267465.66	C41 H68 Na O11	M+
760.4678	1	114278.83	C41 H68 Na O11	M+
761.4691	1	27402.06	C41 H68 Na O11	M+
775.438	1	42564.01		
1496.9424	2	24183.79		

Formula Calculator Element Limits

Element	Min	Max
C	0	200
H	0	400
O	7	15
Na	1	1

Formula Calculator Results

Formula	CalculatedMass	Mz	Diff.(mDa)	Diff. (ppm)	DBE
C41 H68 Na O11	759.4659	759.4650	0.9	1.2	7.5

--- End Of Report ---

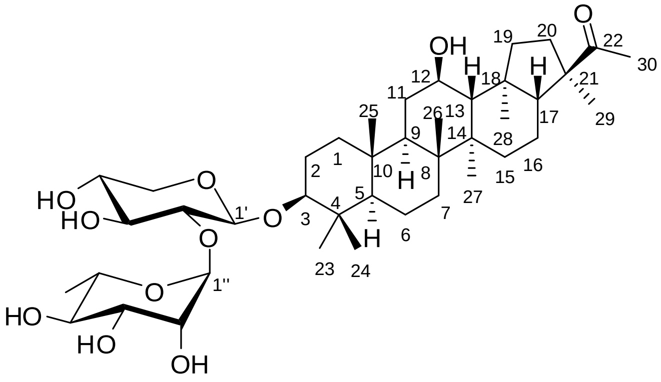

Figure S52. HRESIMS spectrum of **6**.

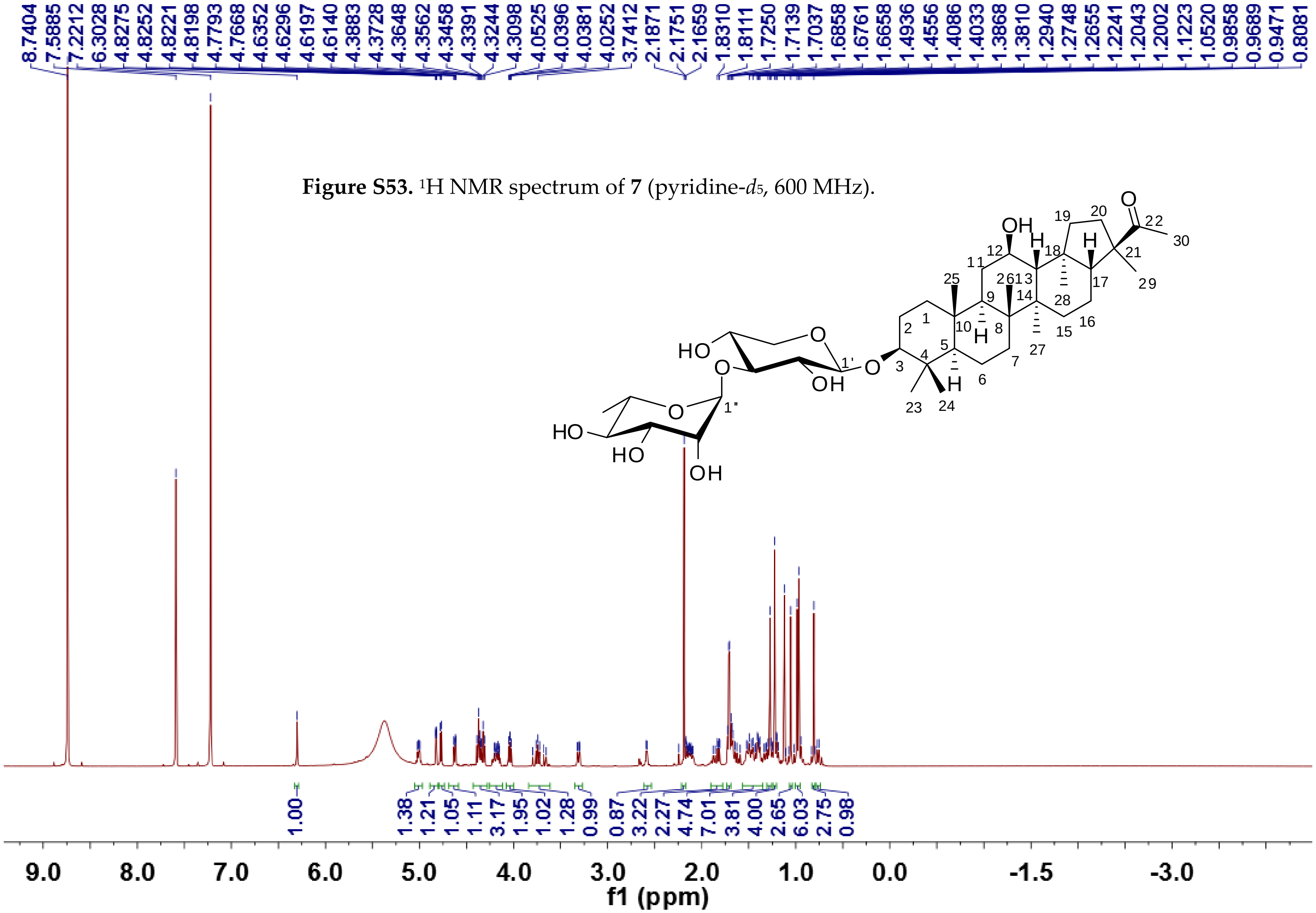

Figure S53. ^{1}H NMR spectrum of **7** (pyridine-*d*5, 600 MHz).

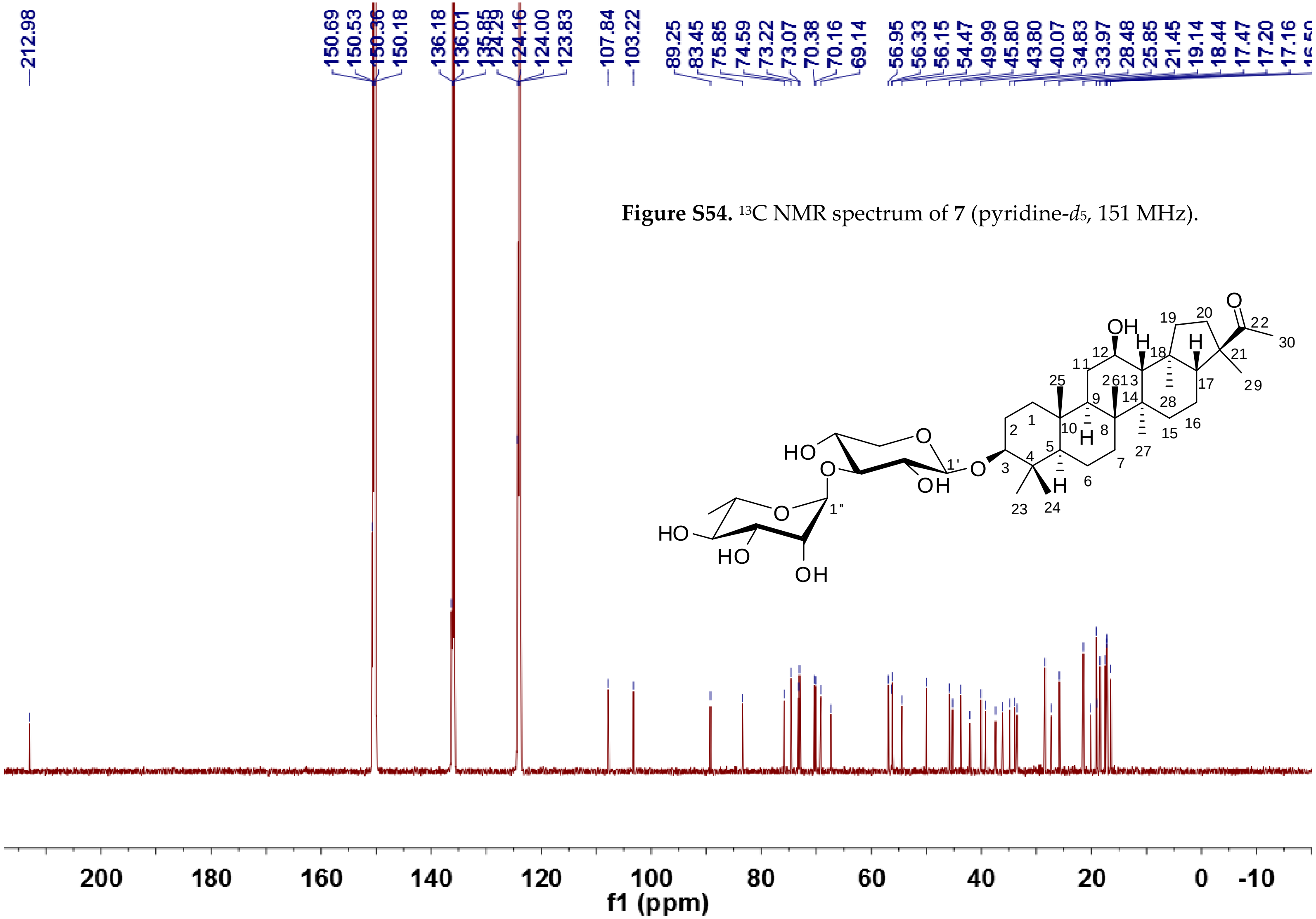

Figure S54. ^{13}C NMR spectrum of **7** (pyridine-*d*$_5$, 151 MHz).

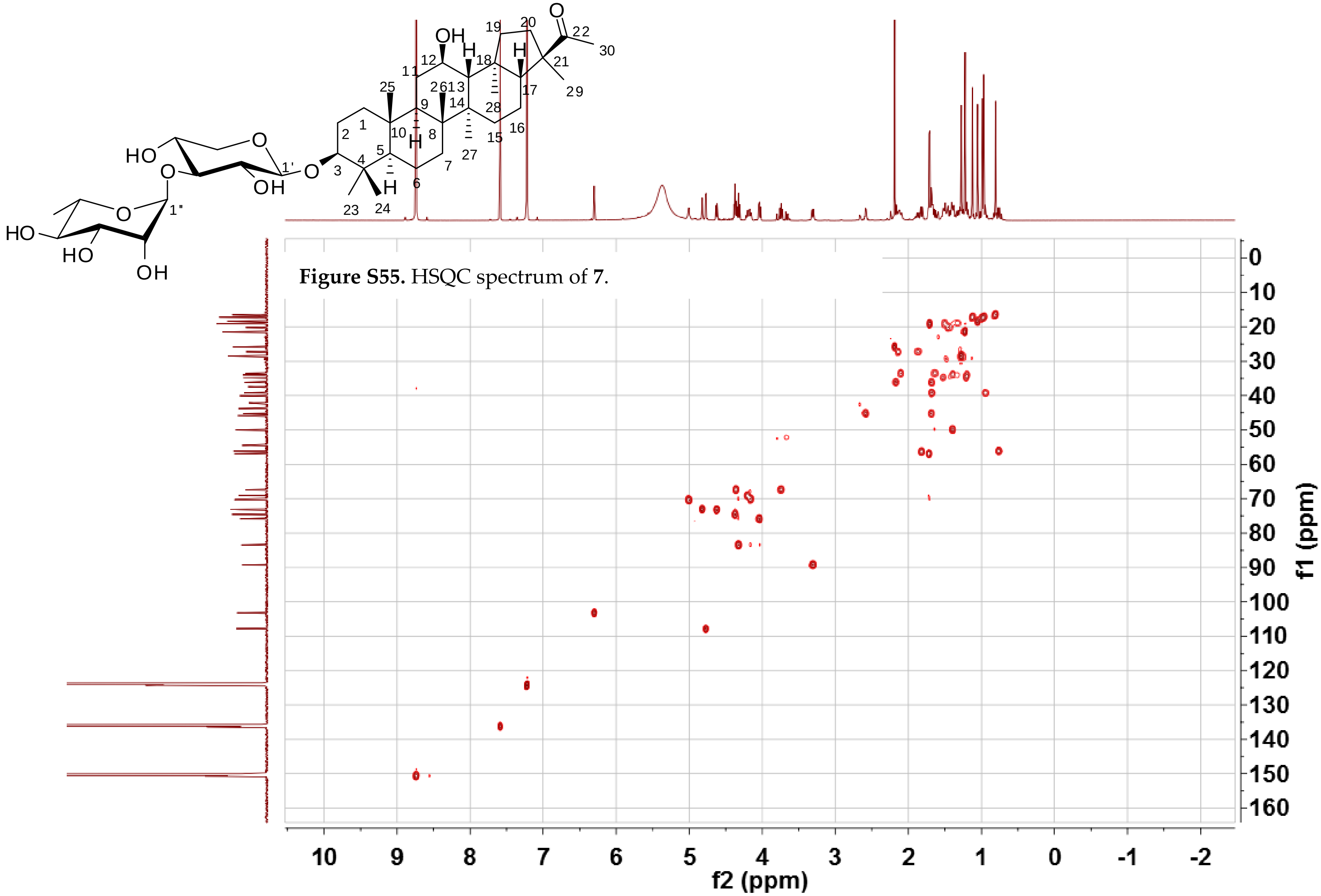

Figure S55. HSQC spectrum of **7**.

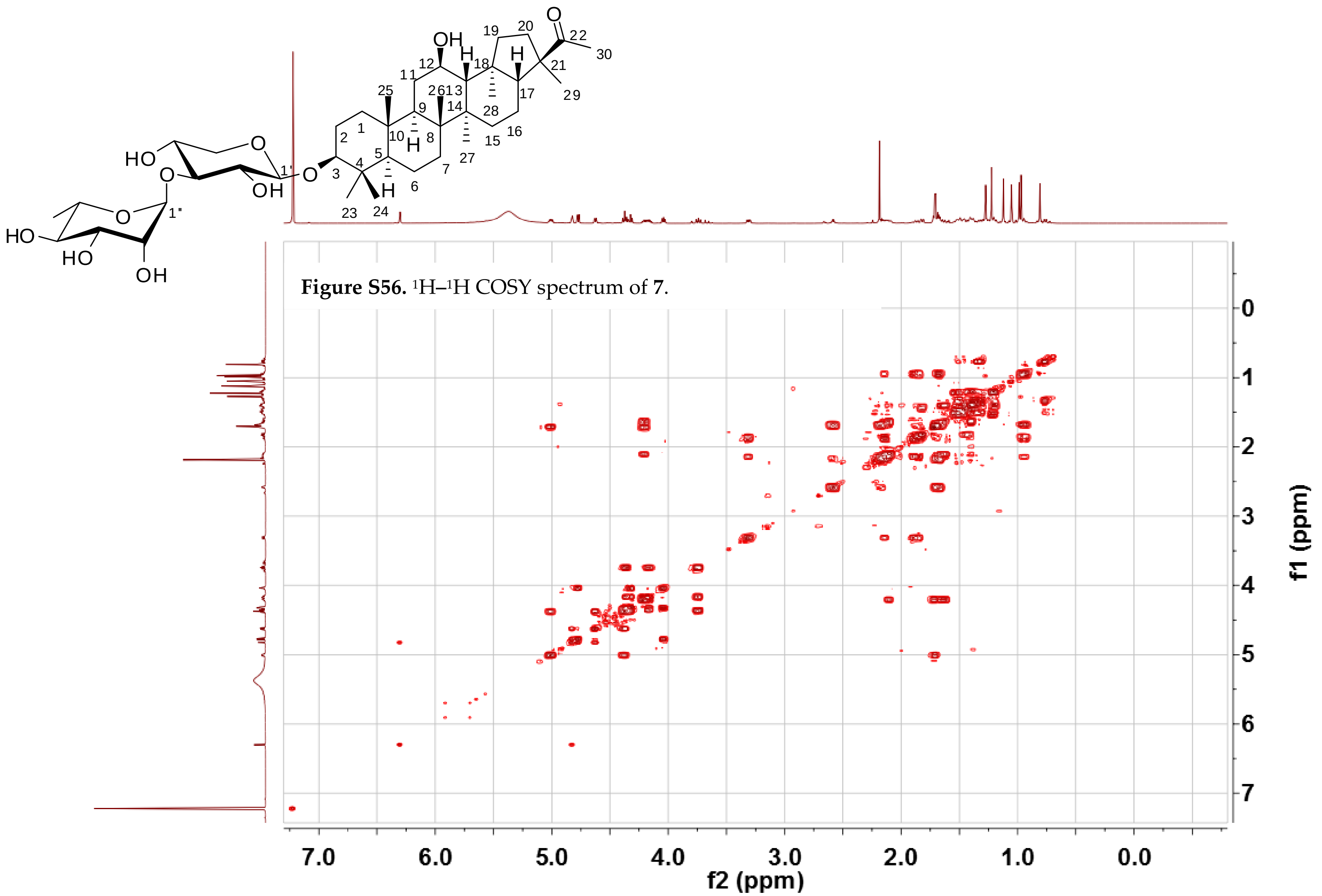

Figure S56. ^1H–^1H COSY spectrum of **7**.

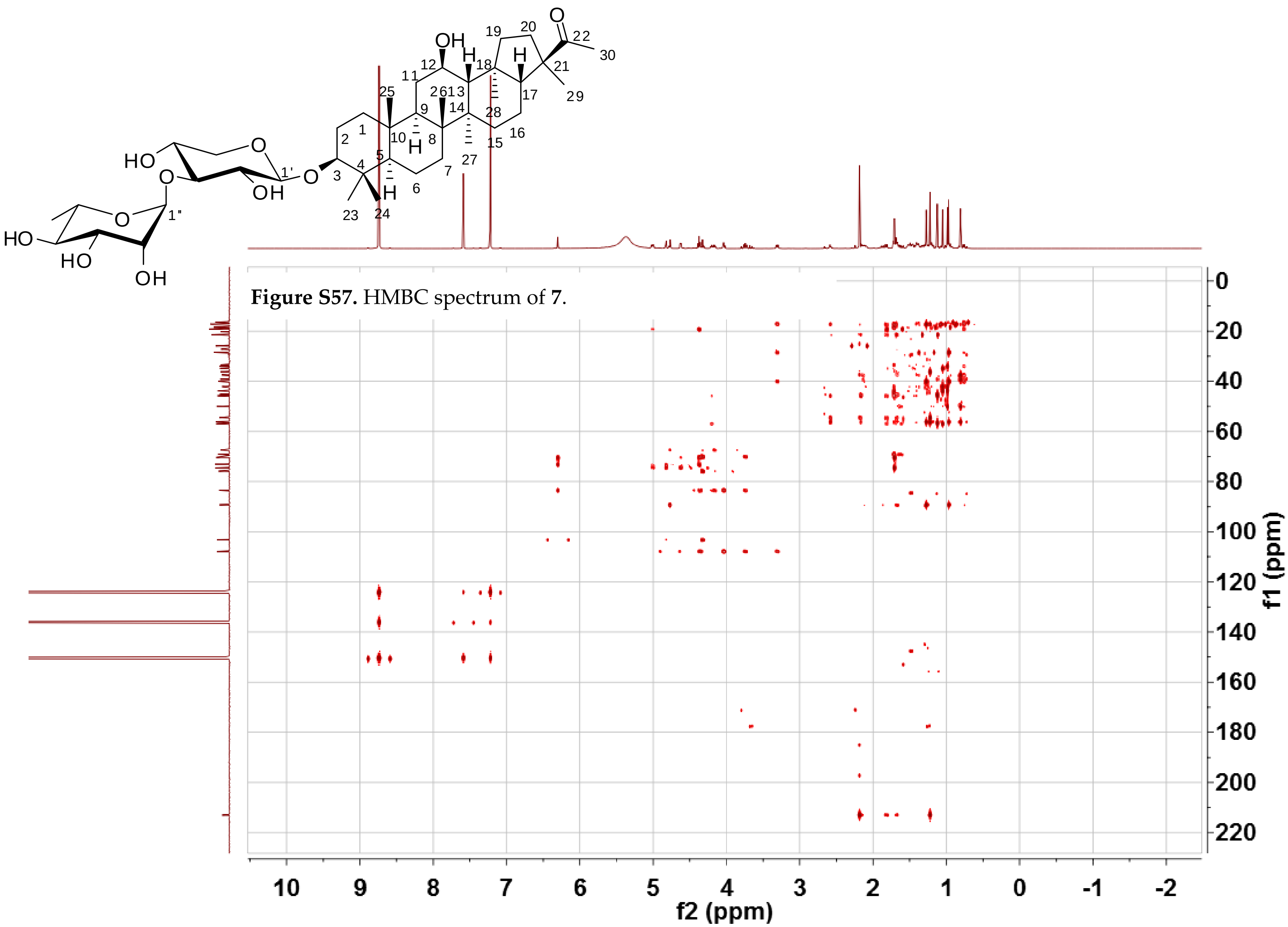

Figure S57. HMBC spectrum of **7**.

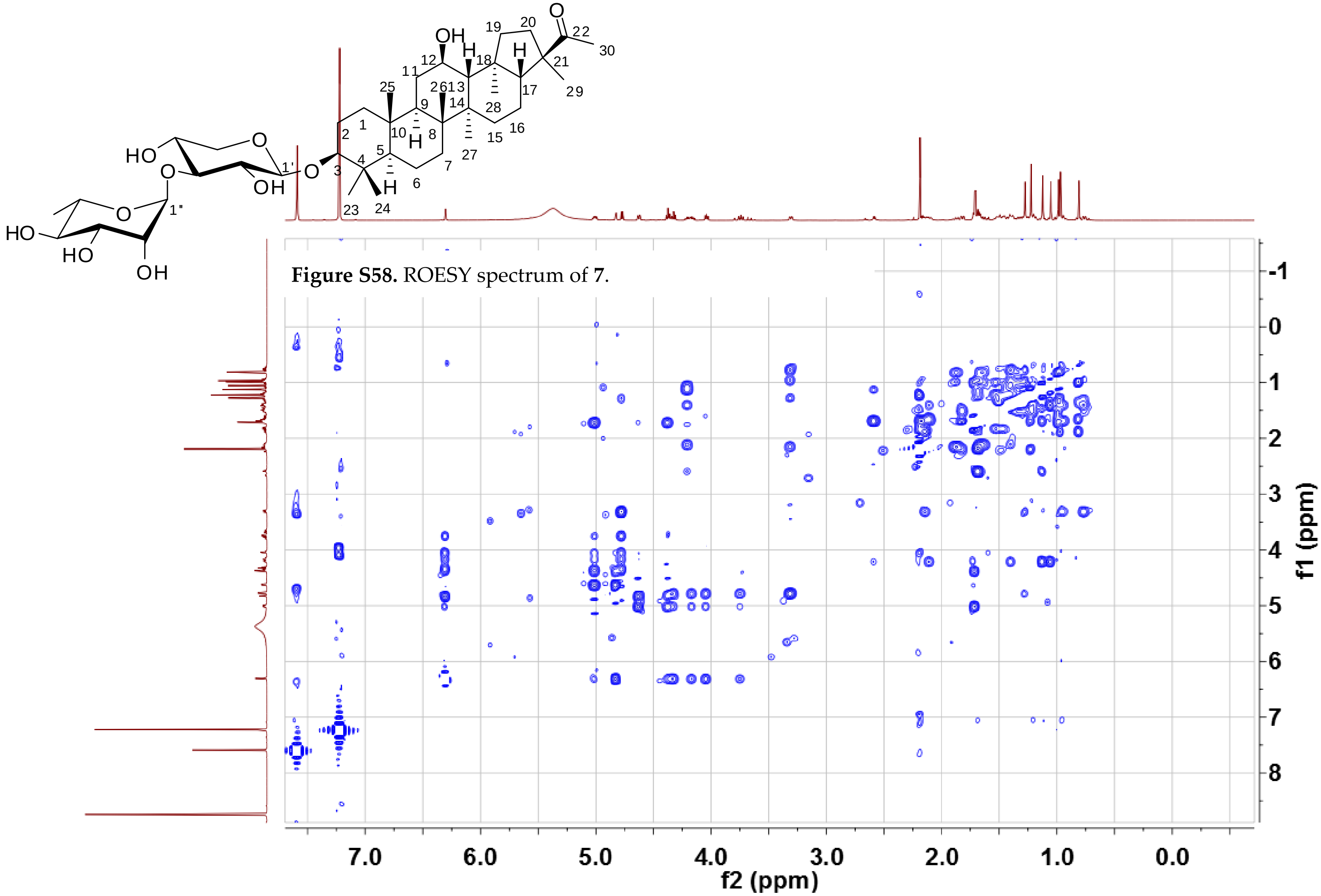

Figure S58. ROESY spectrum of **7**.

**Data Filename**	180905ESIA1.d	**Sample Name**	pdt55a
**Sample Type**	Sample	**Position**	
**Instrument Name**	Agilent G6230 TOF MS	**User Name**	KIB
**Acq Method**	ESI.m	**Acquired Time**	9/5/2018 9:29:38 AM
**IRM Calibration Status**	Success	**DA Method**	ESI.m
**Comment**			

Sample Group **Info.**

Acquisition SW 6200 series TOF/6500 series

Version Q-TOF B.05.01 (B5125.2)

User Spectra

Fragmentor Voltage	Collision Energy	Ionization Mode
200	0	ESI

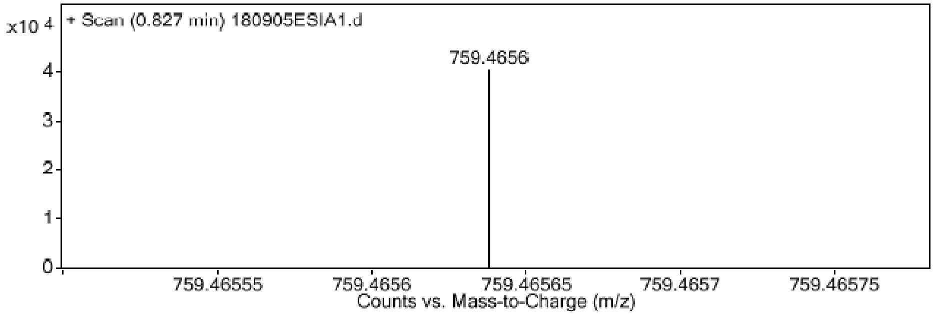

Peak List

m/z	z	Abund	Formula	Ion
105.0429	1	33201.03		
112.1875	1	32109.09		
118.1227		15357.97		
121.0509		187790.47		
759.4656	1	40396.77	C41 H68 Na O11	M+
760.4691	1	18545.26	C41 H68 Na O11	M+
922.0098	1	81067		
923.012	1	15091.32		
1495.9385	1	16909.07		
1496.9434	1	14735.45		

Formula Calculator Element Limits

Element	Min	Max
C	0	200
H	0	400
O	7	15
Na	1	1

Formula Calculator Results

Formula	CalculatedMass	Mz	Diff.(mDa)	Diff. (ppm)	DBE
C41 H68 Na O11	759.4659	759.4656	0.3	0.4	7.5

--- End Of Report ---

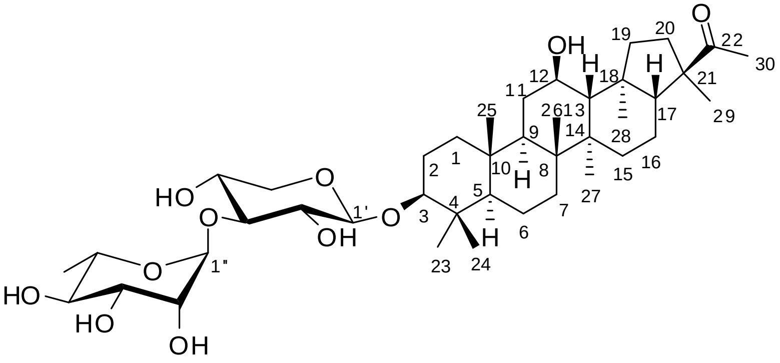

Figure S59. HRESIMS spectrum of **7**.

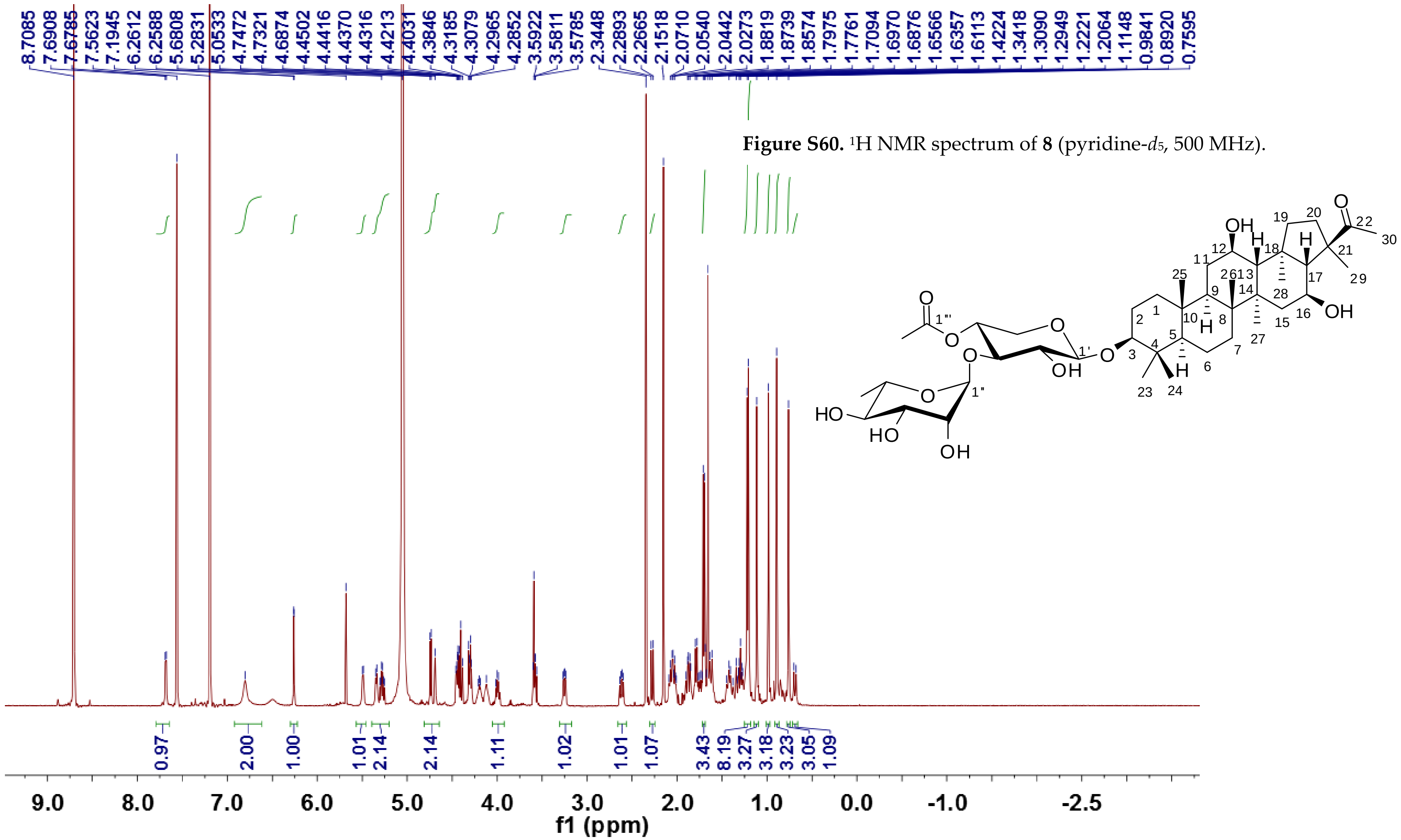

Figure S60. ^{1}H NMR spectrum of **8** (pyridine-*d*5, 500 MHz).

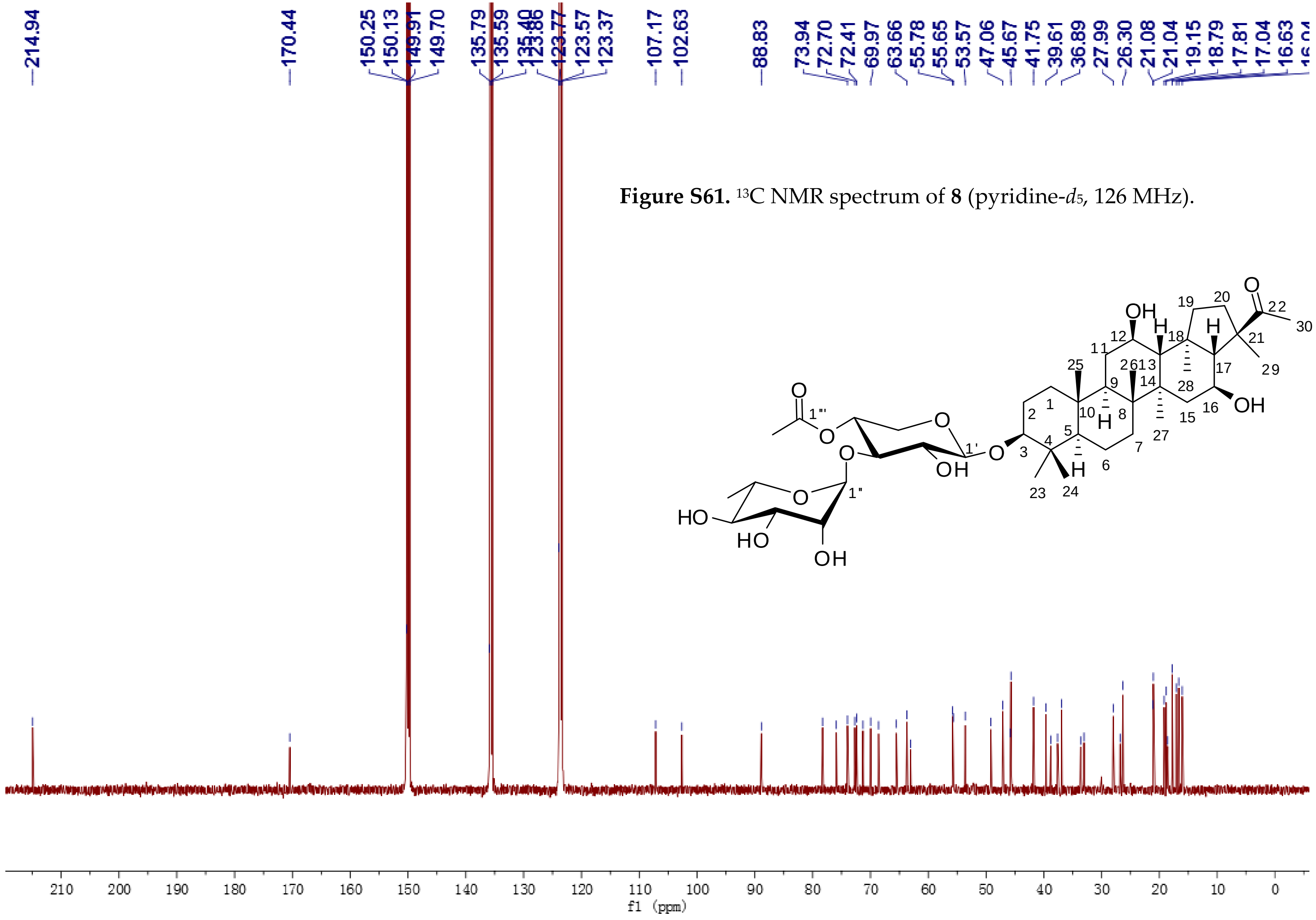

Figure S61. ^{13}C NMR spectrum of **8** (pyridine-*d*$_5$, 126 MHz).

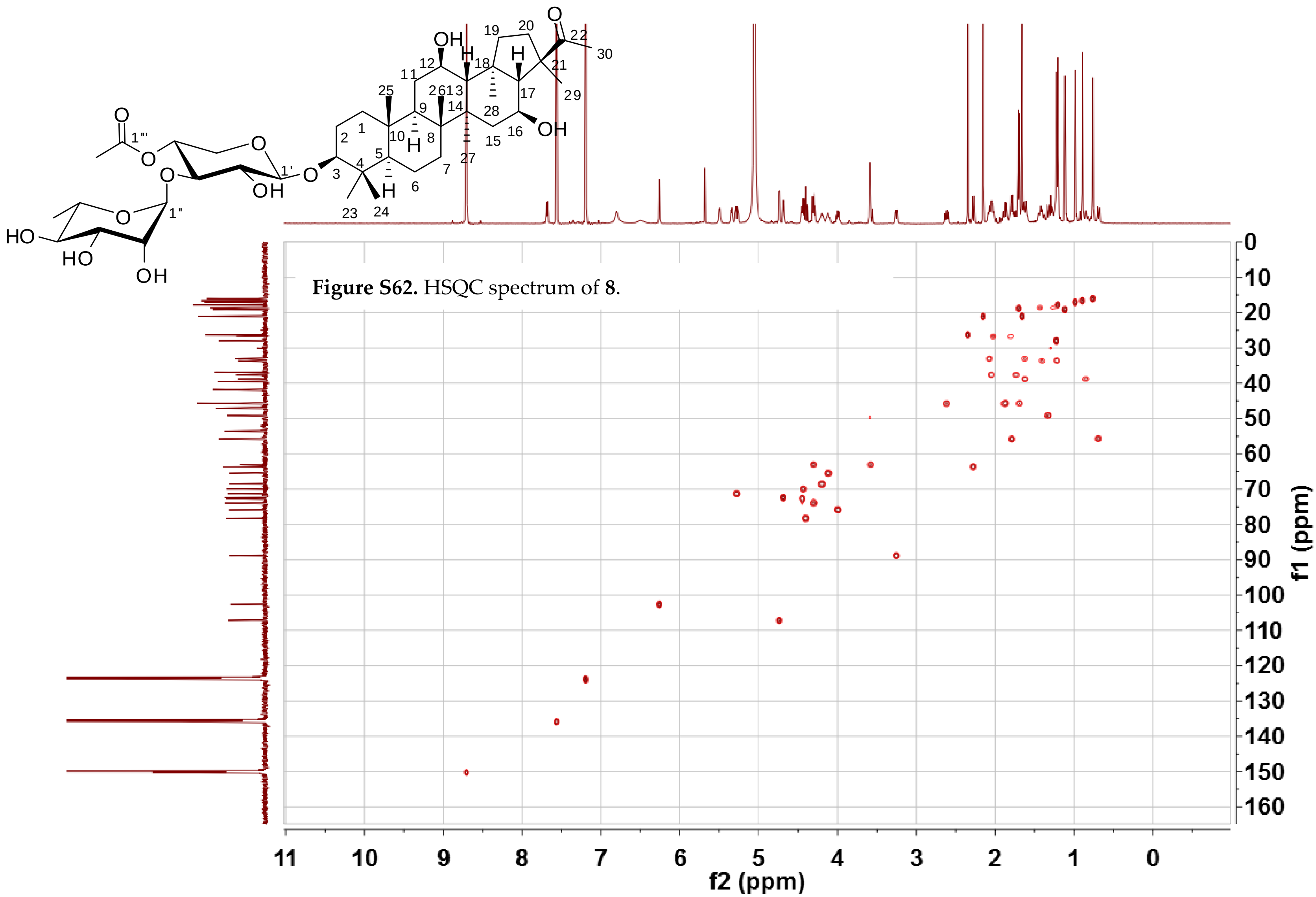

Figure S62. HSQC spectrum of **8**.

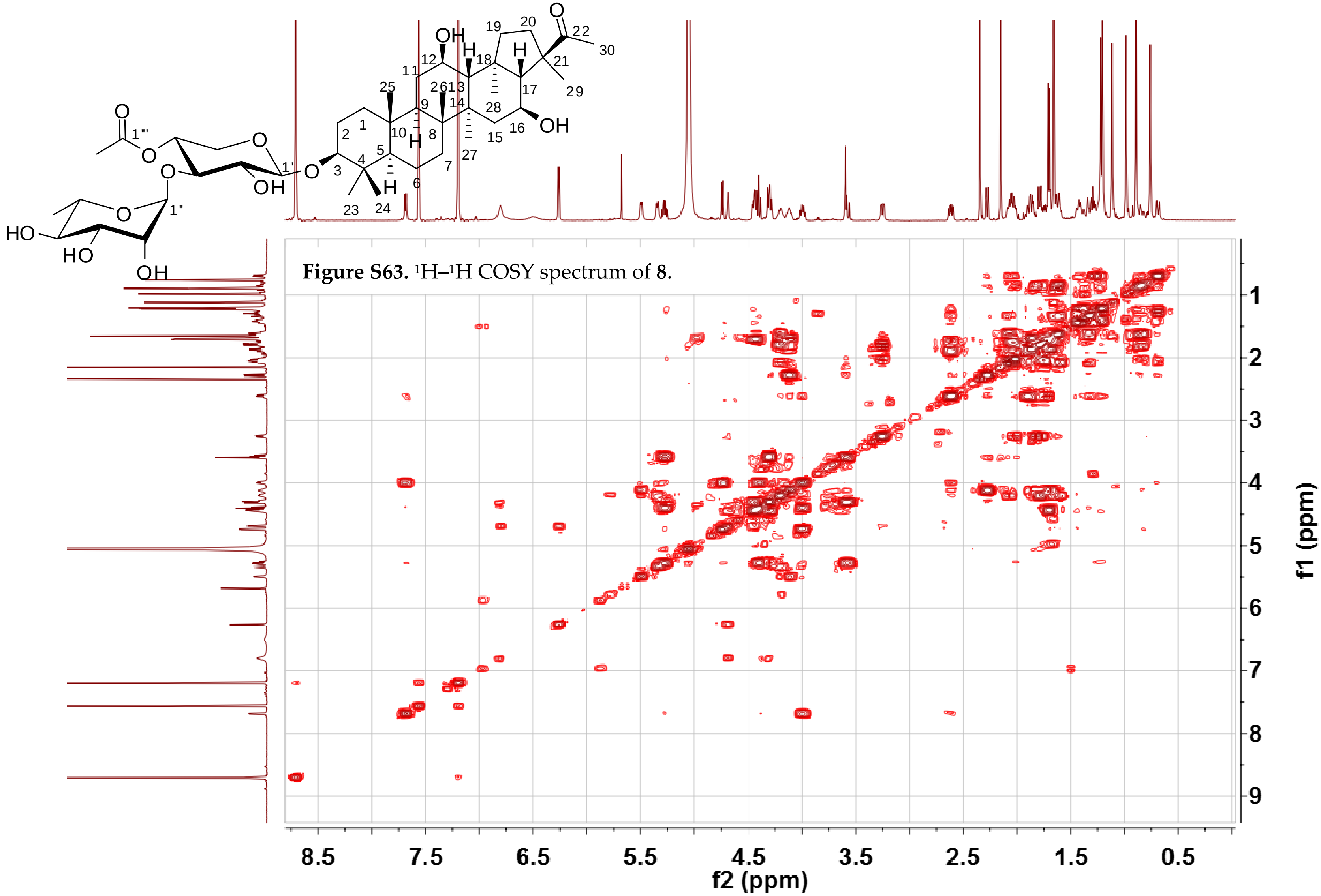

Figure S63. ^{1}H–^{1}H COSY spectrum of **8**.

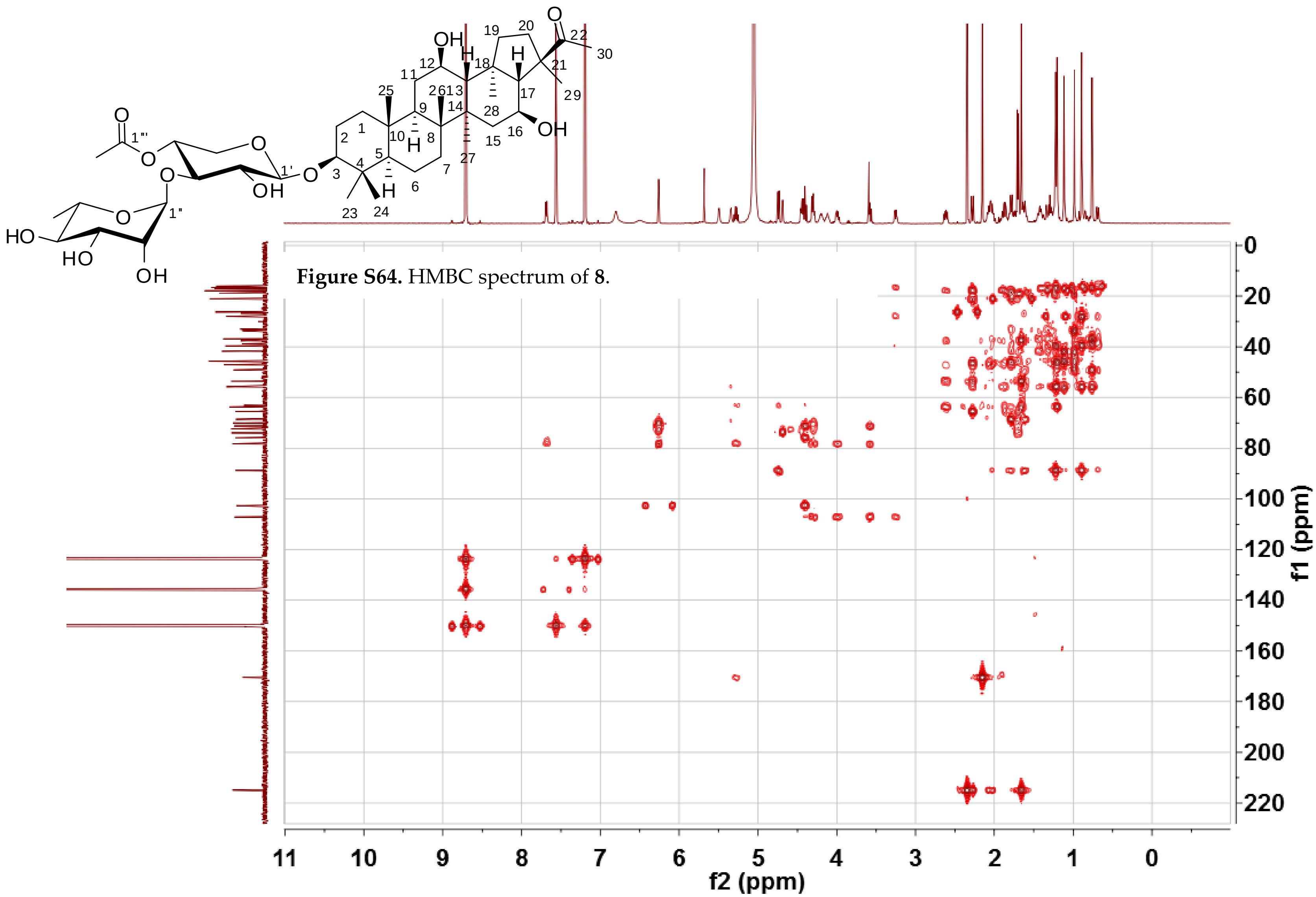

Figure S64. HMBC spectrum of **8**.

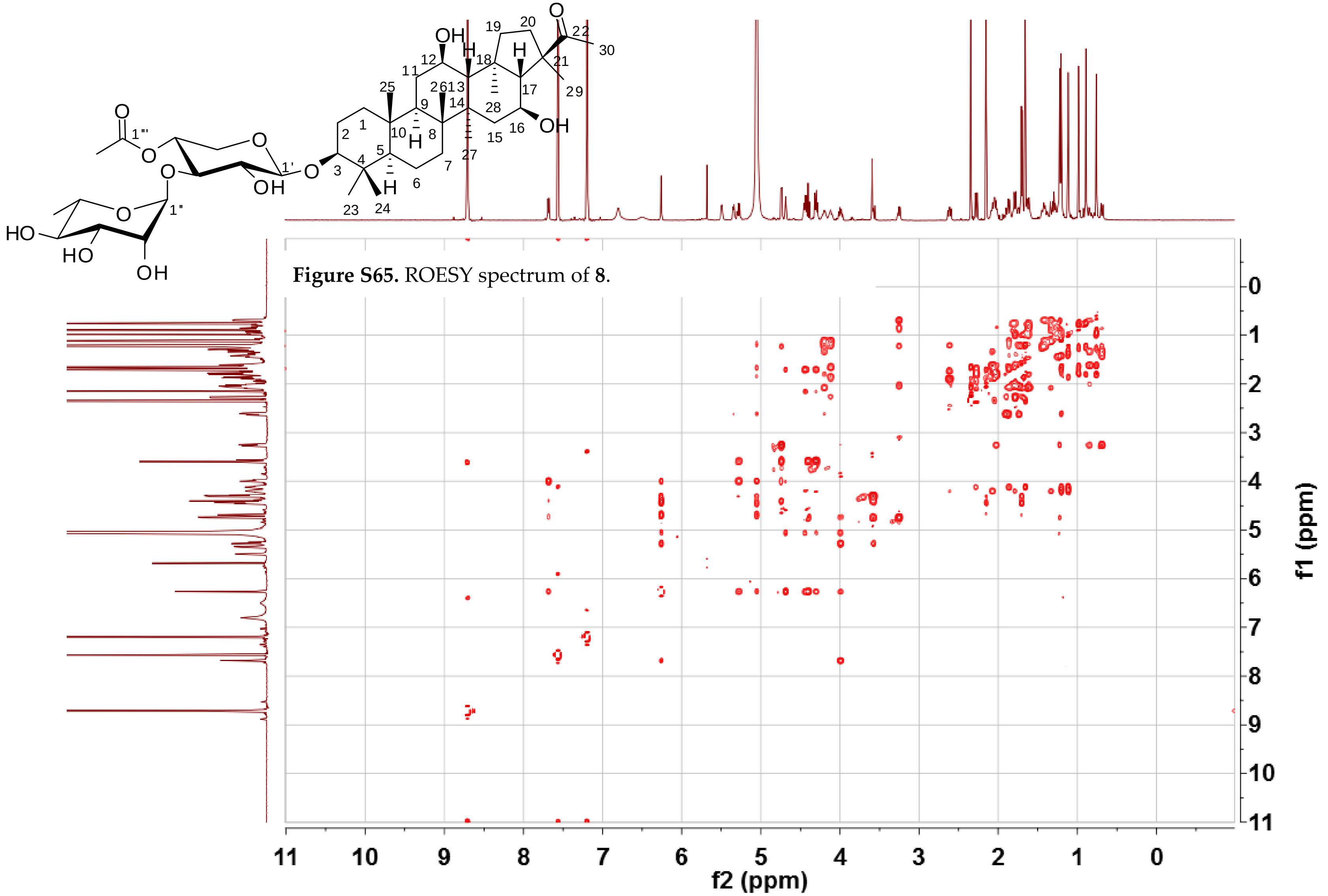

Figure S65. ROESY spectrum of **8**.

**Data Filename**	180409ESIA1.d	**Sample Name**	pdt 25
**Sample Type**	Sample	**Position**	
**Instrument Name**	Agilent G6230 TOF MS	**User Name**	KIB
**Acq Method**	ESI.m	**Acquired Time**	4/9/2018 10:11:10 AM
**IRM Calibration Status**	Success	**DA Method**	ESI.m
**Comment**			

Sample Group **Info.**

Acquisition SW Version 6200 series TOF/6500 series Q-TOF B.05.01 (B5125.2)

User Spectra

Fragmentor Voltage	Collision Energy	Ionization Mode
280	0	ESI

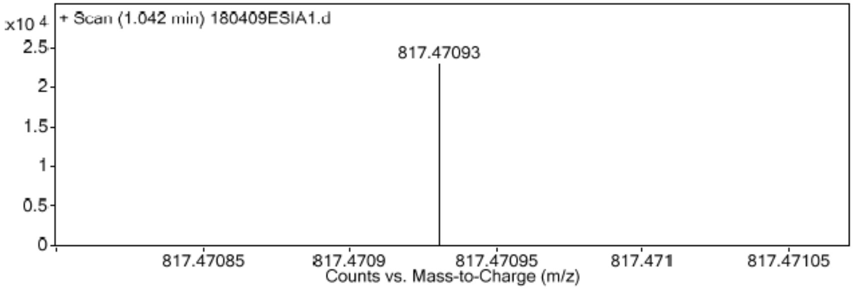

Peak List

m/z	z	Abund	Formula	Ion
105.04381	1	7861.56		
107.0607	1	9758.46		
121.05087		10394.14		
437.21141	1	4743.33		
619.5291	1	6658.52		
817.47093	1	22933.7	C43 H70 Na O13	M+
818.47367	1	11609.89	C43 H70 Na O13	M+
922.00983	1	182465.73		
923.01244	1	33655.16		
924.01361	1	4635.13		

Formula Calculator Element Limits

Element	Min	Max
C	0	200
H	0	400
O	10	15
Na	1	1

Formula Calculator Results

Formula	CalculatedMass	Mz	Diff.(mDa)	Diff. (ppm)	DBE
C43 H70 Na O13	817.4714	817.4709	0.5	0.6	8.5

--- End Of Report ---

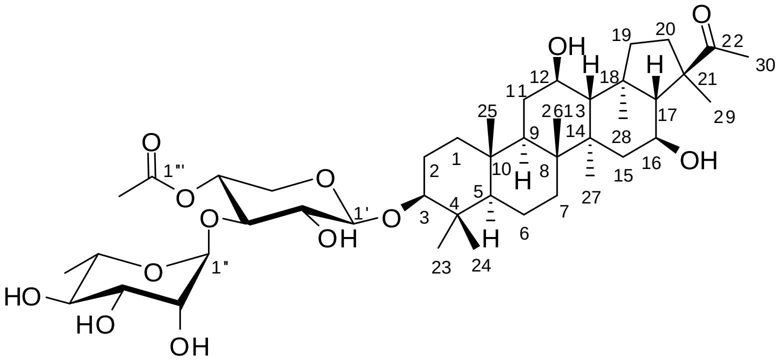

Figure S66. HRESIMS spectrum of **8**.

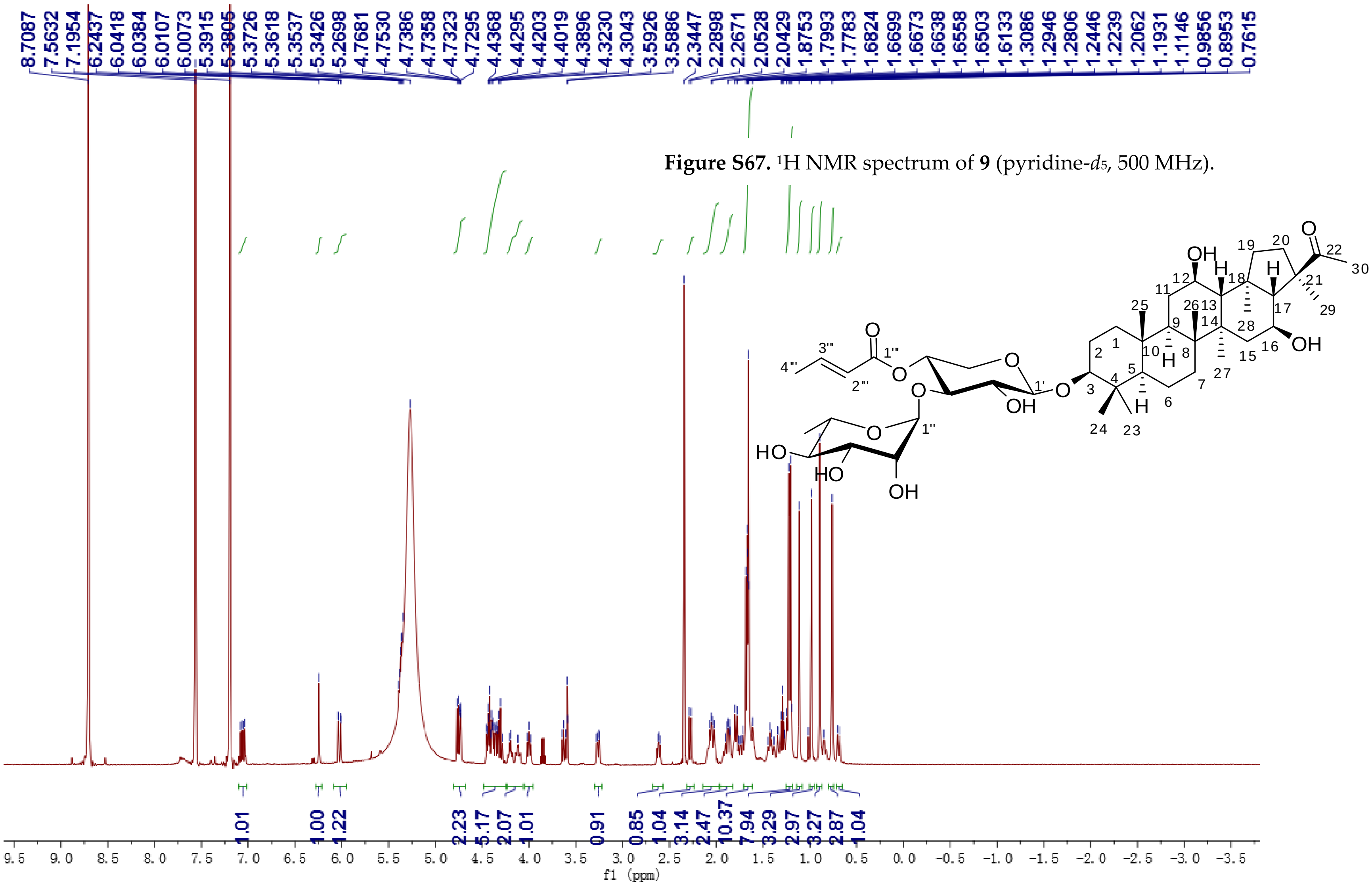

Figure S67. ^{1}H NMR spectrum of **9** (pyridine-d_5, 500 MHz).

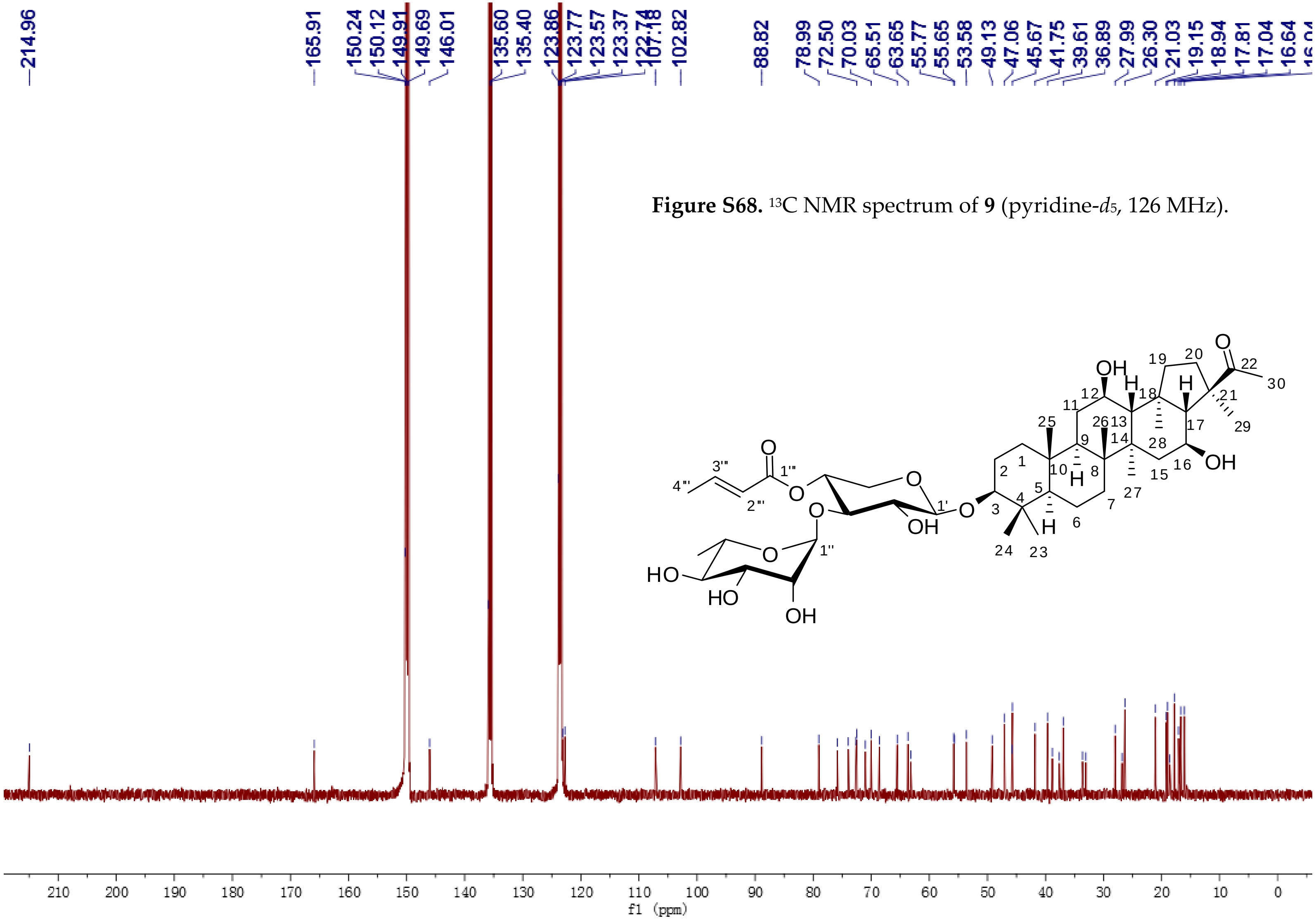

Figure S68. ^{13}C NMR spectrum of **9** (pyridine-*d*$_5$, 126 MHz).

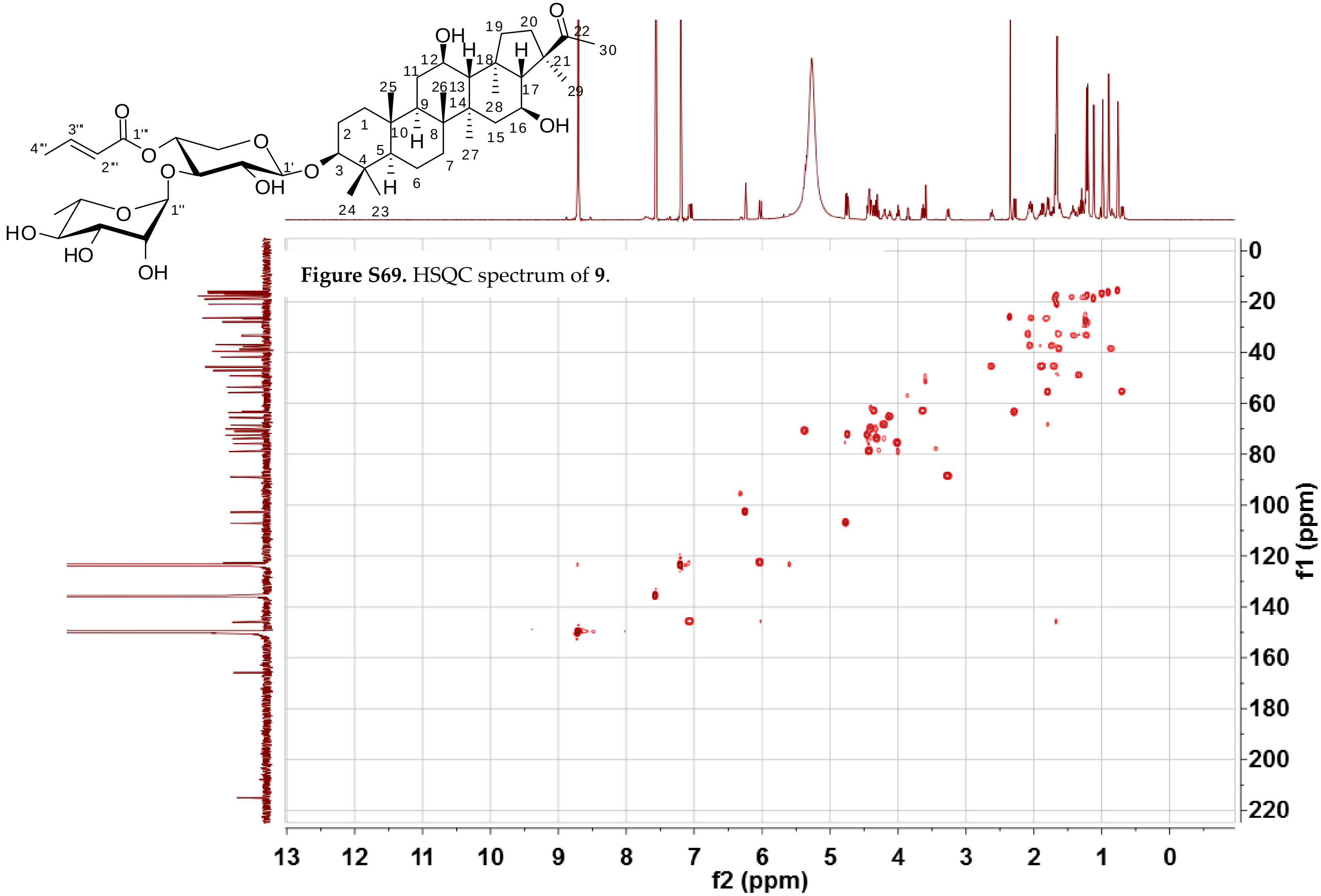

Figure S69. HSQC spectrum of **9**.

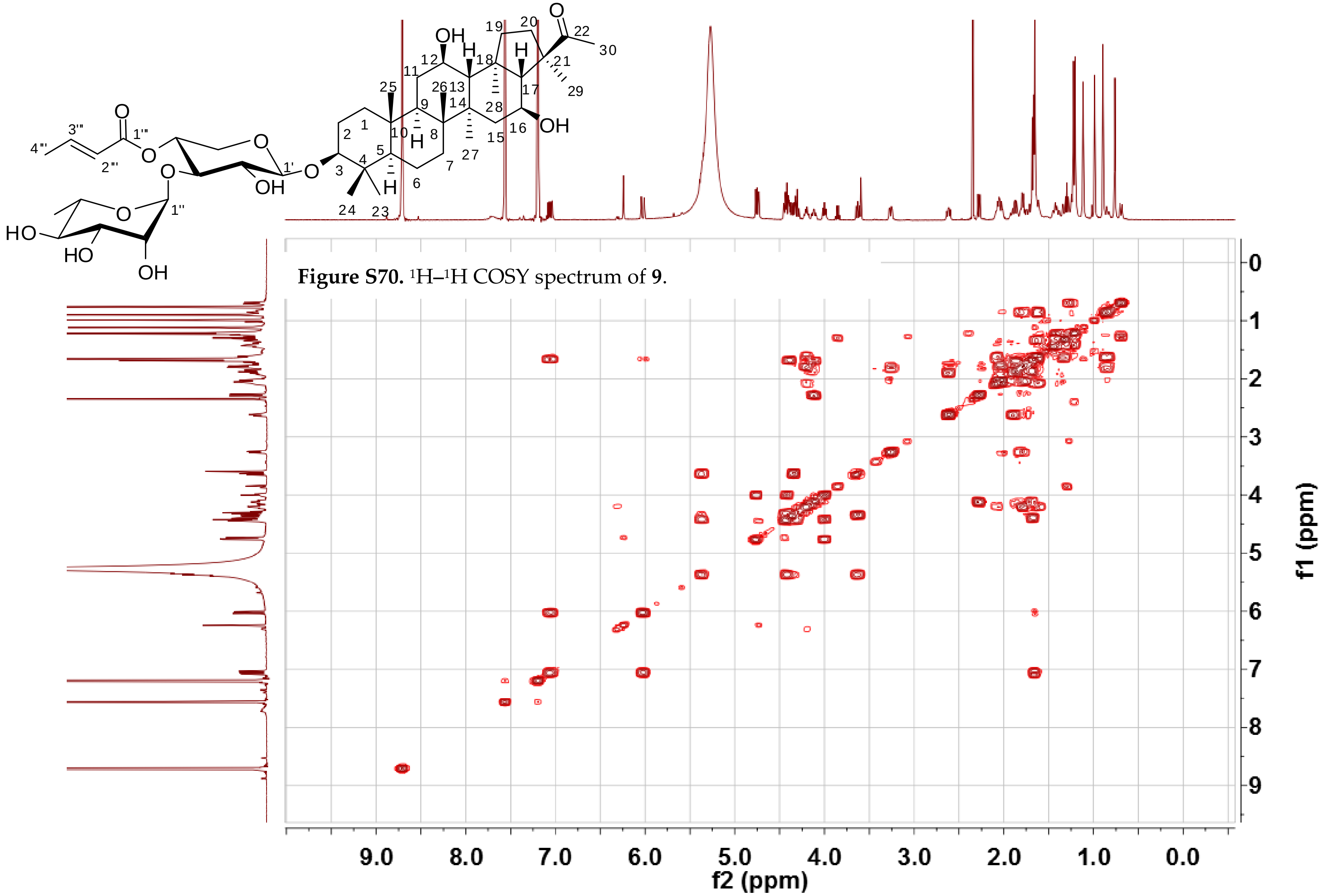

Figure S70. ^{1}H–^{1}H COSY spectrum of **9**.

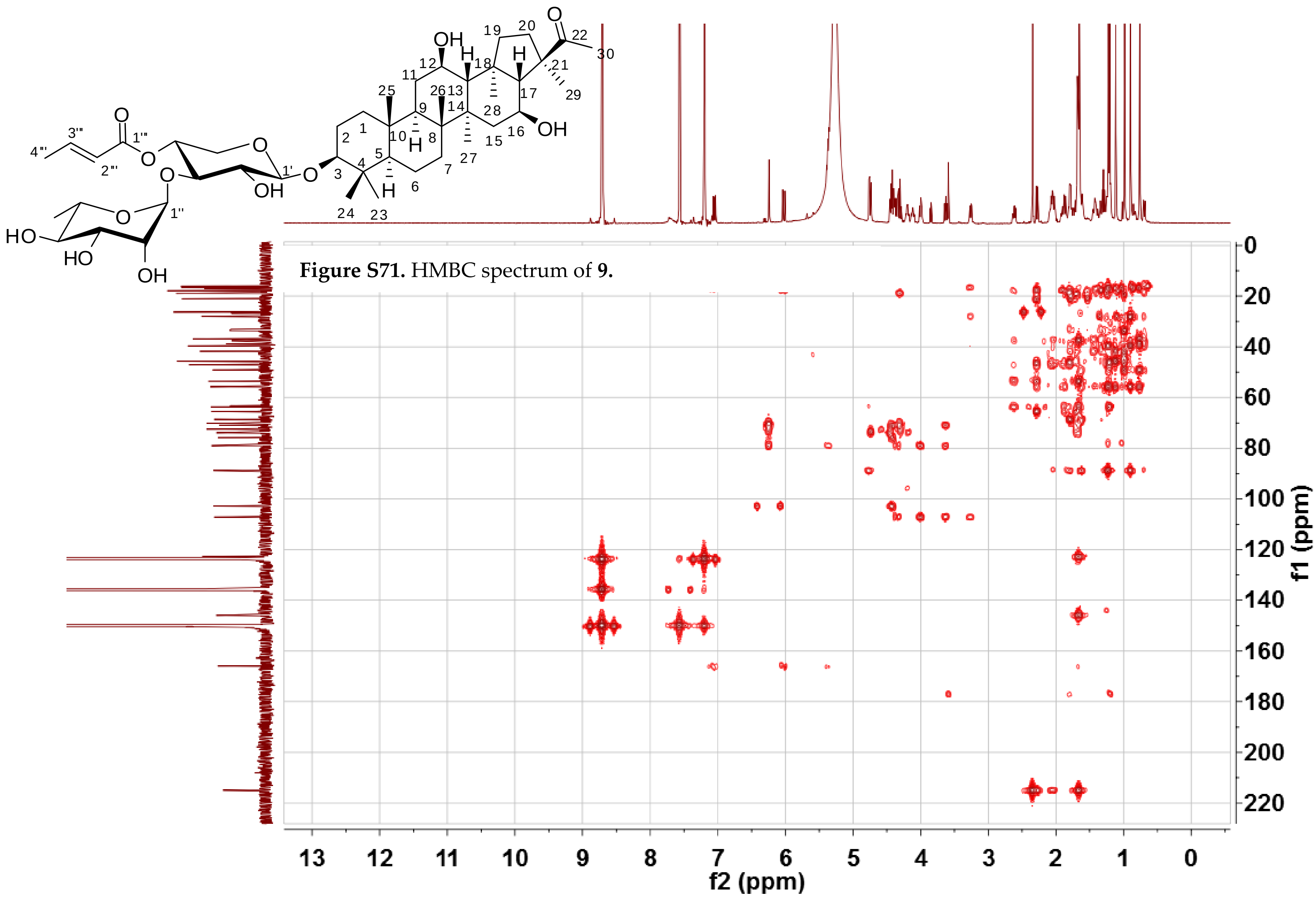

Figure S71. HMBC spectrum of **9**.

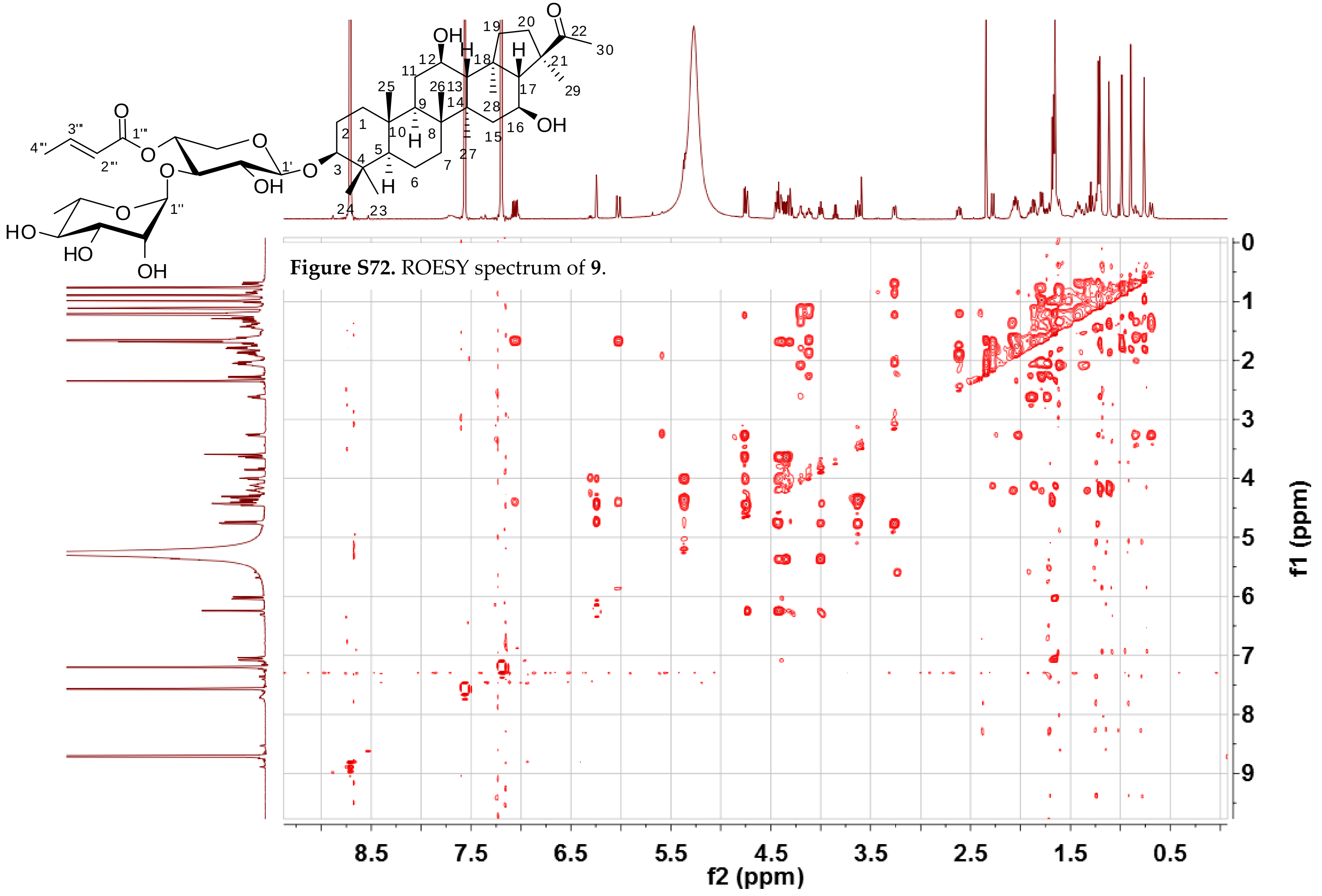

Figure S72. ROESY spectrum of **9**.

Data Filename	171023ESIA2.d	Sample Name	pdt21
Sample Type	Sample	Position	
Instrument Name	Agilent G6230 TOF MS	User Name	KIB
Acq Method	ESI.m	Acquired Time	10/23/2017 10:50:53 AM
IRM Calibration Status	Success	DA Method	ESI.m
Comment			

Sample Group		Info.
Acquisition SW	6200 series TOF/6500 series	
Version	Q-TOF B.05.01 (B5125.2)	

User Spectra

Fragmentor Voltage	Collision Energy	Ionization Mode
250	0	ESI

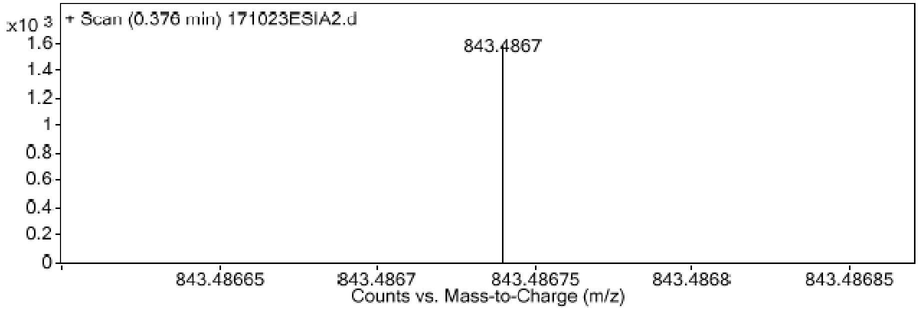

Peak List

m/z	z	Abund
112.1874	1	10026.1
141.0132	1	30683.75
166.0626	1	5483.01
182.0396	1	20365.15
218.9723		2794.6
289.4064		3495.92
290.4138	1	5943.82
406.9446	1	2901.76
973.4422	2	4264.08
1014.4671	1	2768.69

Formula Calculator Element Limits

Element	Min	Max
C	0	200
H	0	400
O	9	15
Na	1	1

Formula Calculator Results

Formula	CalculatedMass	Mz	Diff.(mDa)	Diff. (ppm)	DBE
C45 H72 Na O13	843.4871	843.4867	0.4	0.4	9.5

--- End Of Report ---

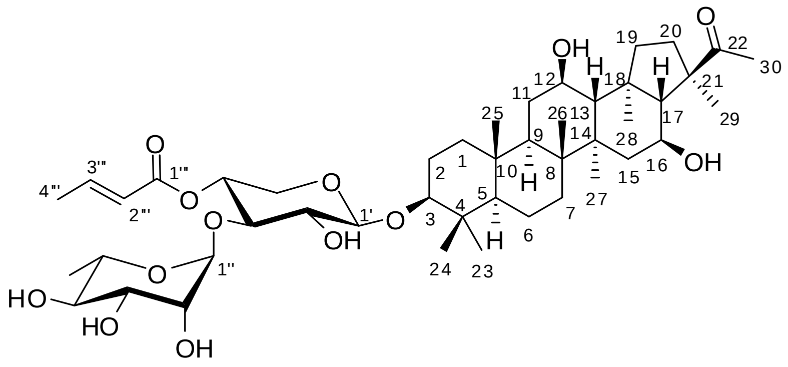

Figure S73. HRESIMS spectrum of **9**.

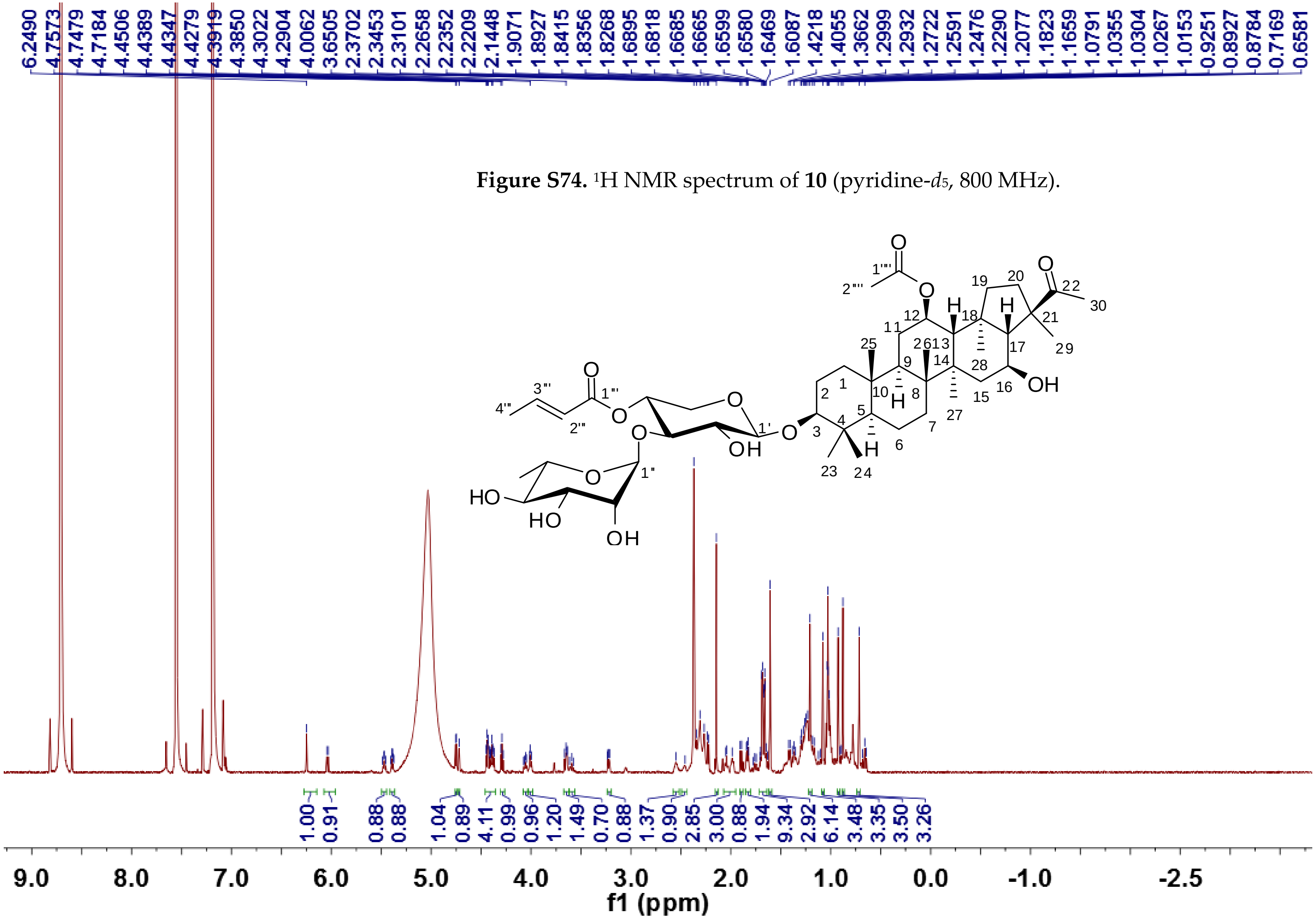

Figure S74. ^{1}H NMR spectrum of **10** (pyridine-d_5, 800 MHz).

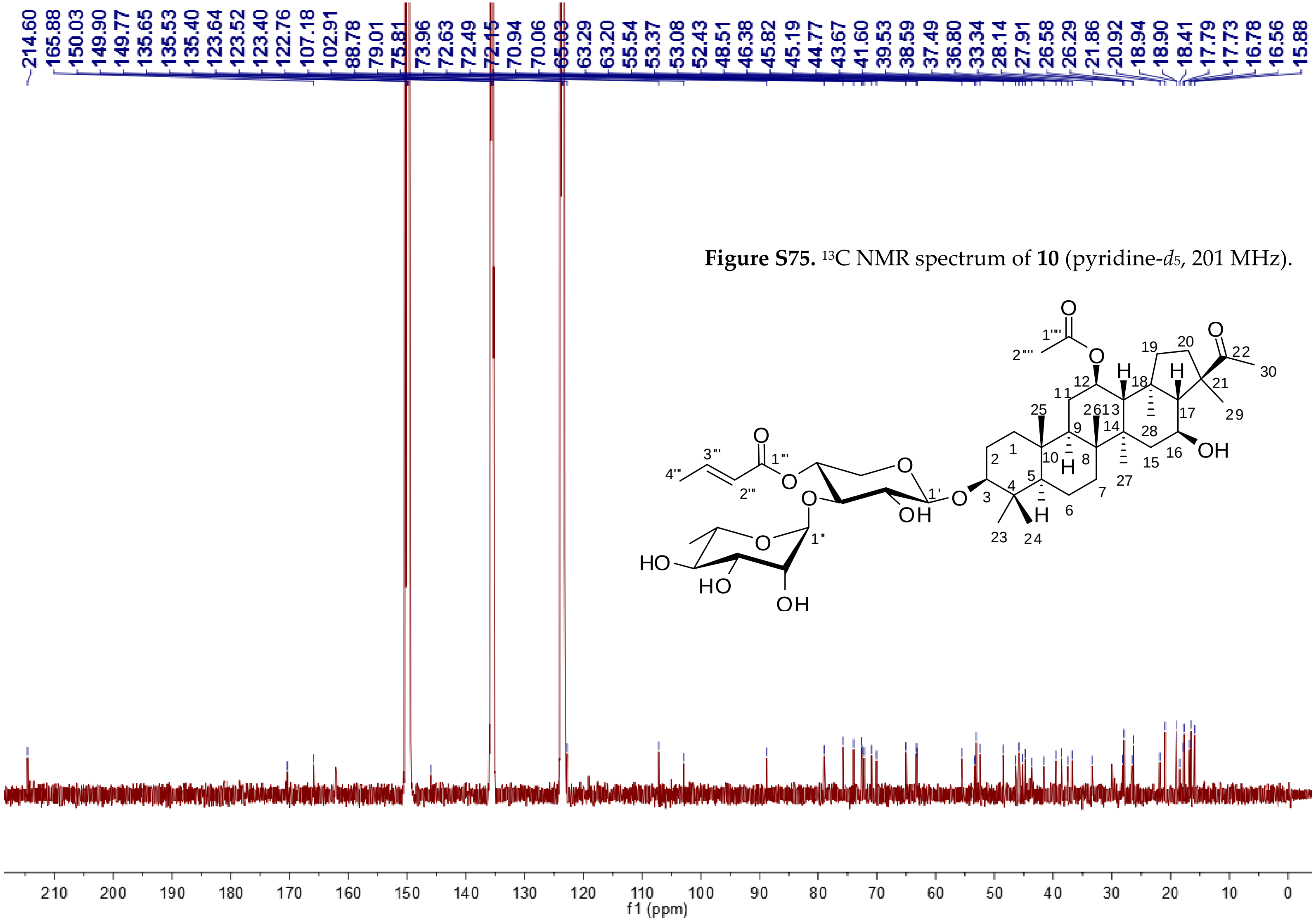

Figure S75. ^{13}C NMR spectrum of **10** (pyridine-*d*5, 201 MHz).

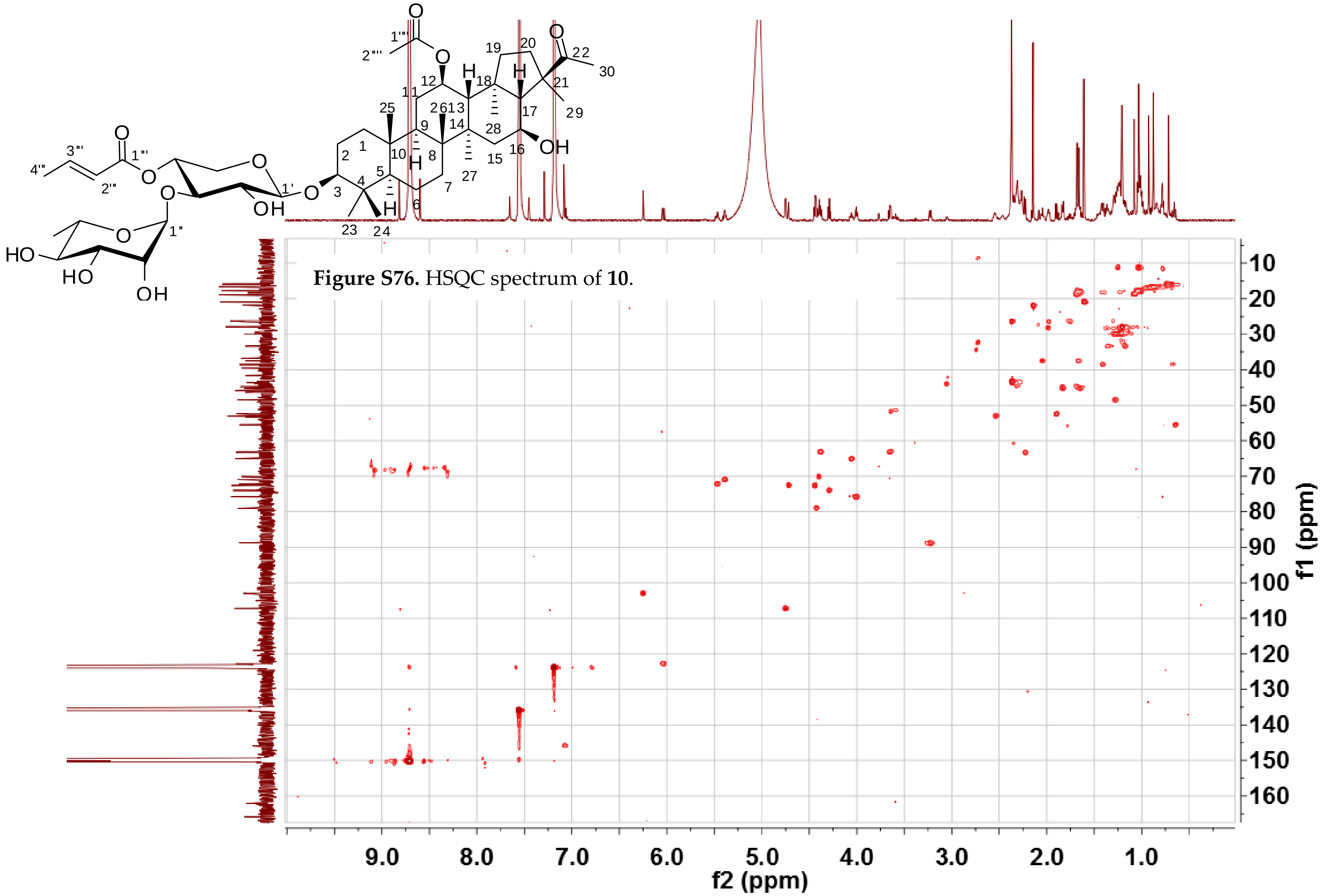

Figure S76. HSQC spectrum of **10**.

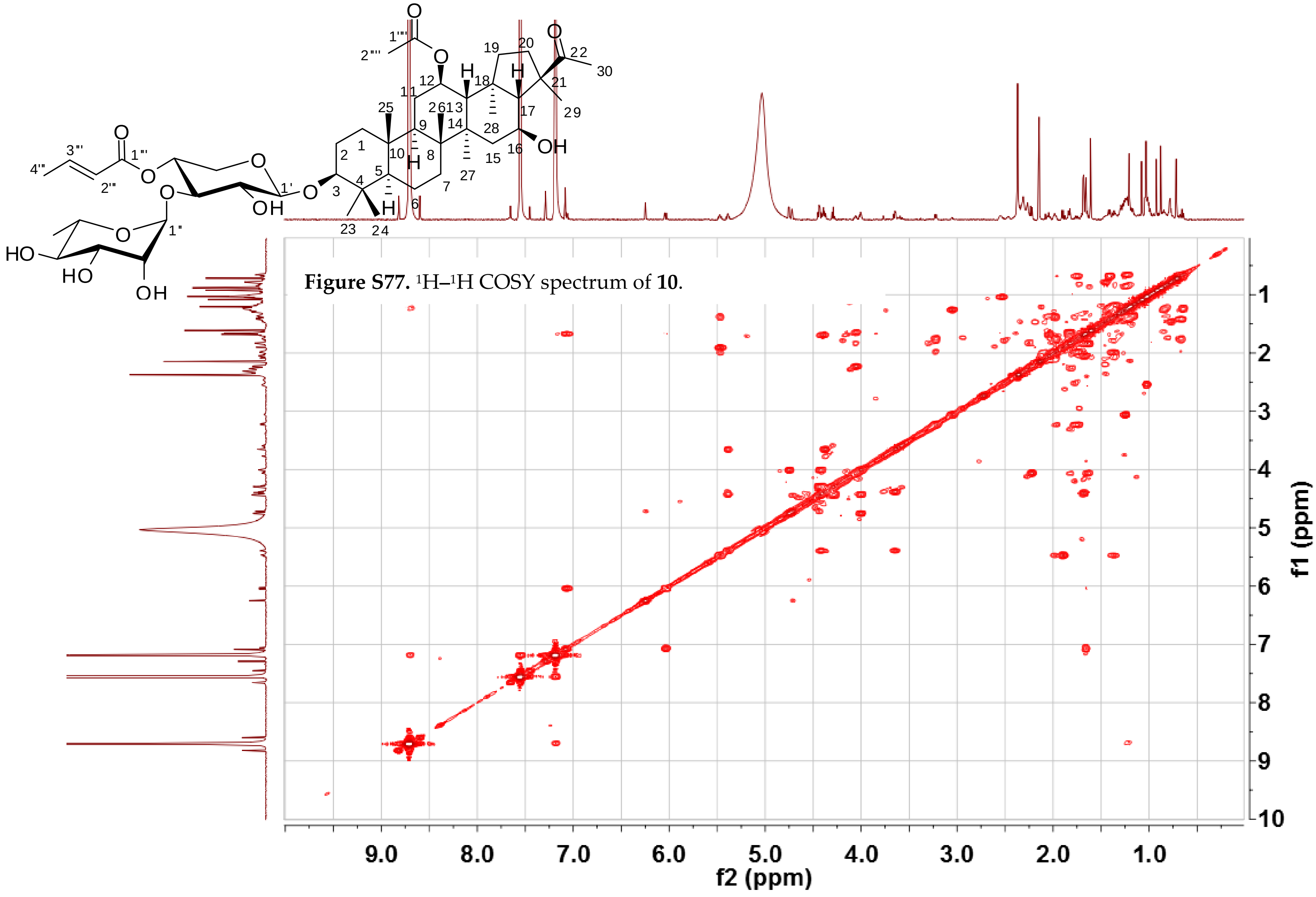

Figure S77. ^{1}H–^{1}H COSY spectrum of **10**.

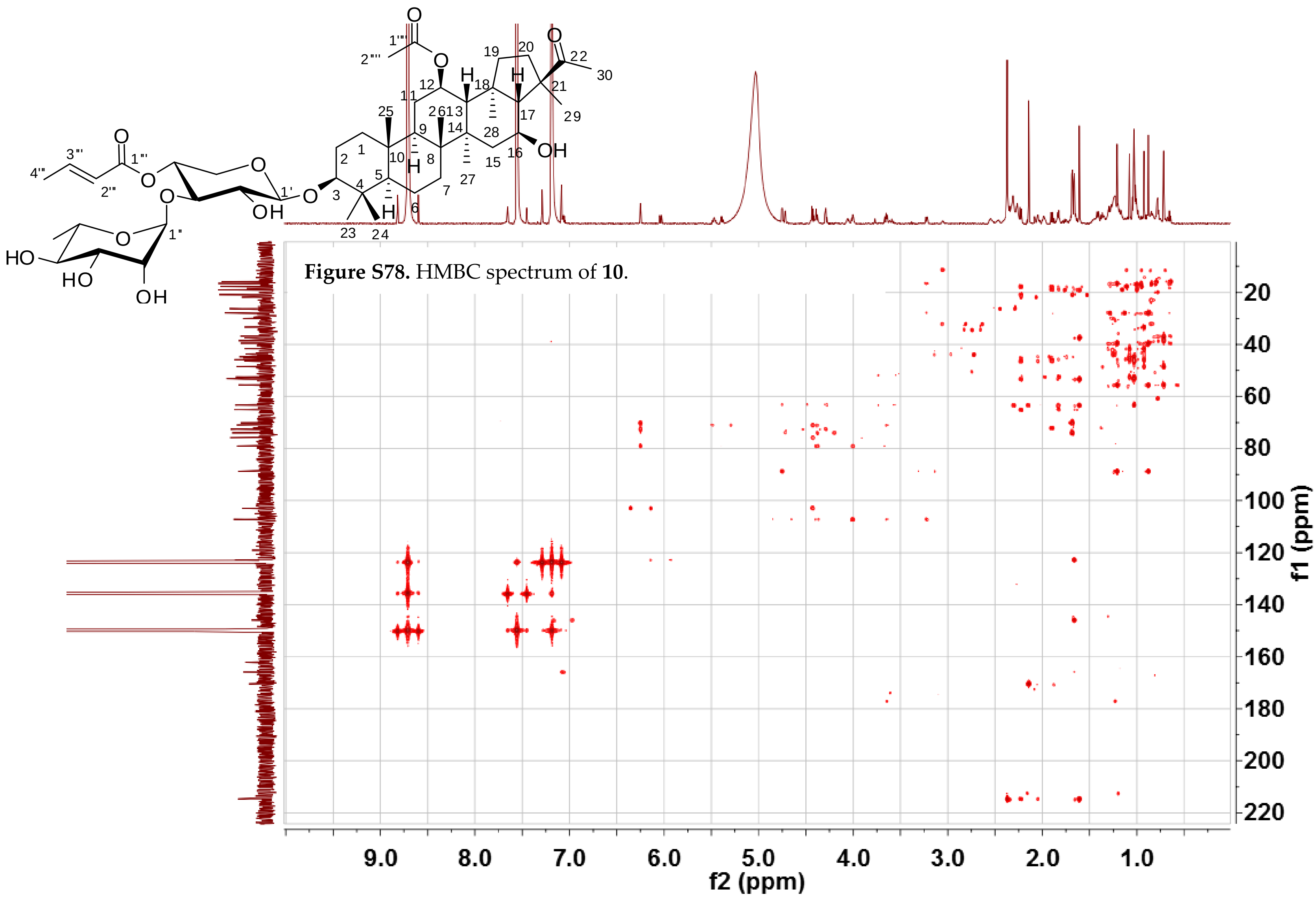

Figure S78. HMBC spectrum of **10**.

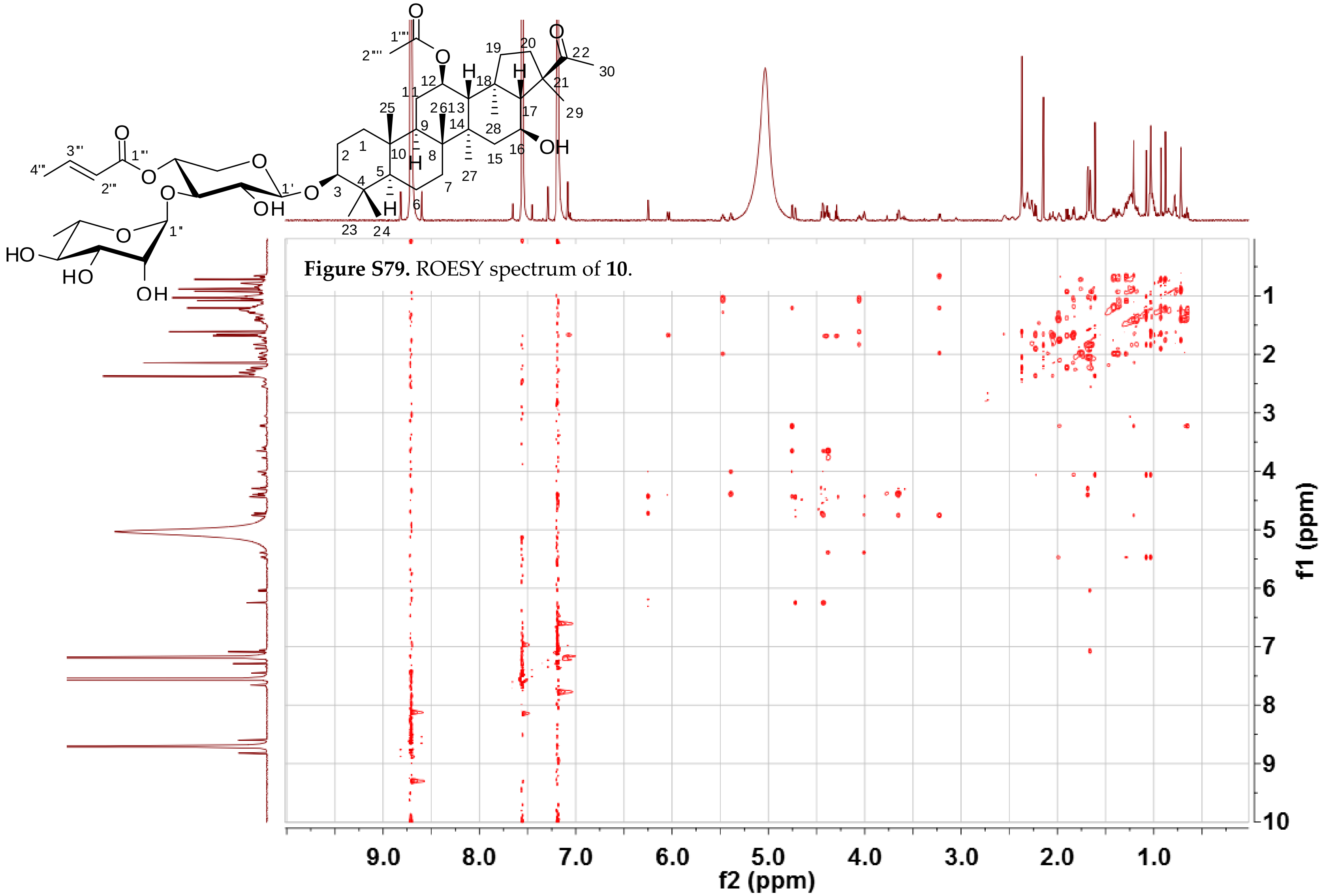

Figure S79. ROESY spectrum of **10**.

**Data Filename**	180824ESIA5.d	**Sample Name**	pdt 36a
**Sample Type**	Sample	**Position**	
**Instrument Name**	Agilent G6230 TOF MS	**User Name**	KIB
**Acq Method**	ESI.m	**Acquired Time**	8/24/2018 2:21:16 PM
**IRM Calibration Status**	Success	**DA Method**	ESI.m
**Comment**			

**Sample Group**		**Info.**
**Acquisition SW Version**	6200 series TOF/6500 series Q-TOF B.05.01 (B5125.2)	

User Spectra

Fragmentor Voltage	Collision Energy	Ionization Mode
340	0	ESI

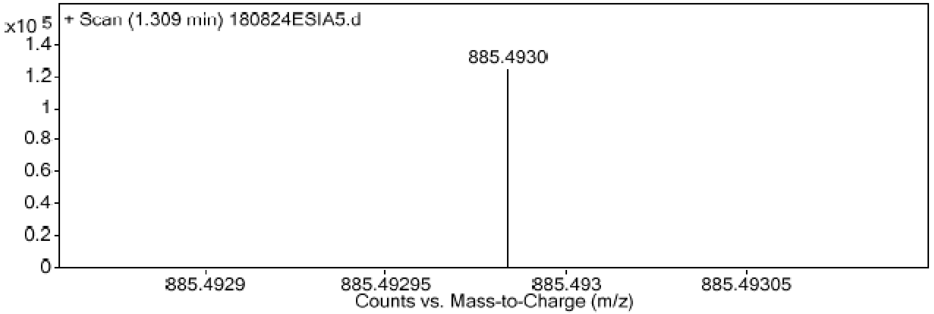

Figure S80. HRESIMS spectrum of **10**.

Peak List

m/z	z	Abund	Formula	Ion
112.1865	1	57330.41		
885.493	1	124318	C47 H74 Na O14	M+
886.4952	1	59689.01	C47 H74 Na O14	M+
887.4997	1	19585.49	C47 H74 Na O14	M+
901.4633	1	46731.28		
902.4662	1	23420.69		
947.5848	1	506711.25		
948.5882	1	291532.06		
949.5898	1	87454.73		
950.5916	1	20514.56		

Formula Calculator Element Limits

Element	Min	Max
C	0	200
H	0	400
O	10	20
Na	1	1

Formula Calculator Results

Formula	CalculatedMass	Mz	Diff.(mDa)	Diff. (ppm)	DBE
C47 H74 Na O14	885.4976	885.4930	4.6	5.2	10.5

--- End Of Report ---

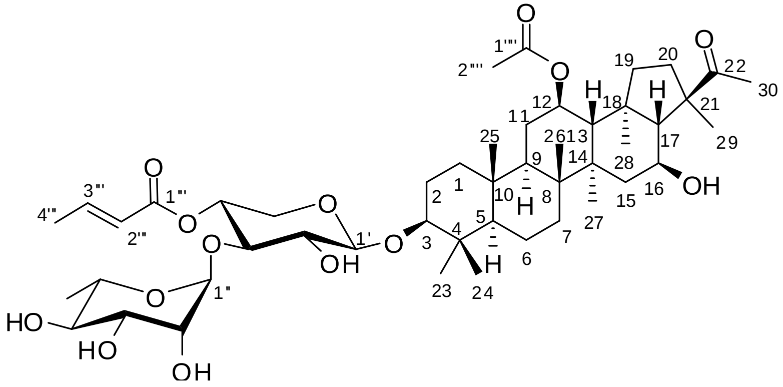

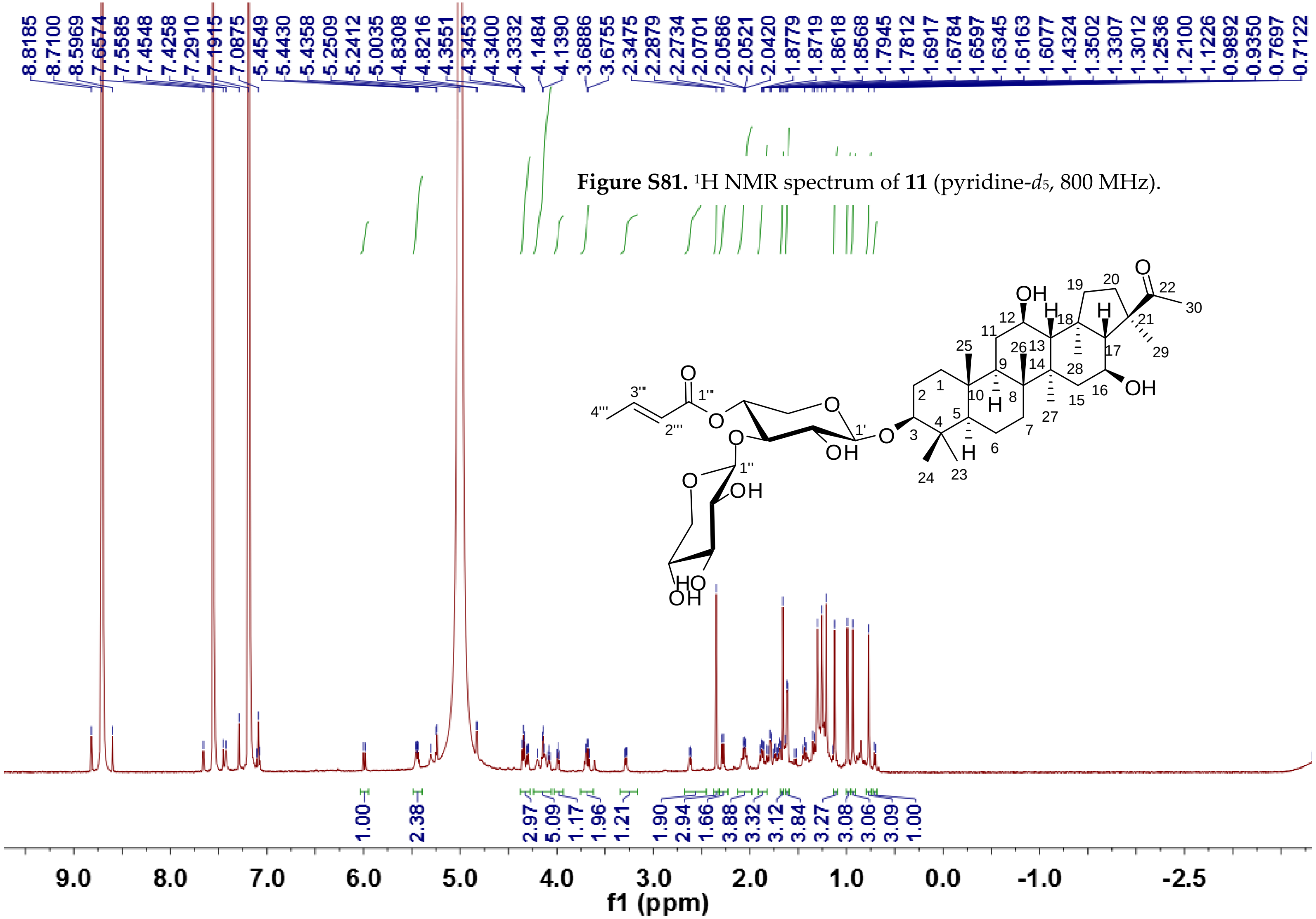

Figure S81. ^{1}H NMR spectrum of **11** (pyridine-d_5, 800 MHz).

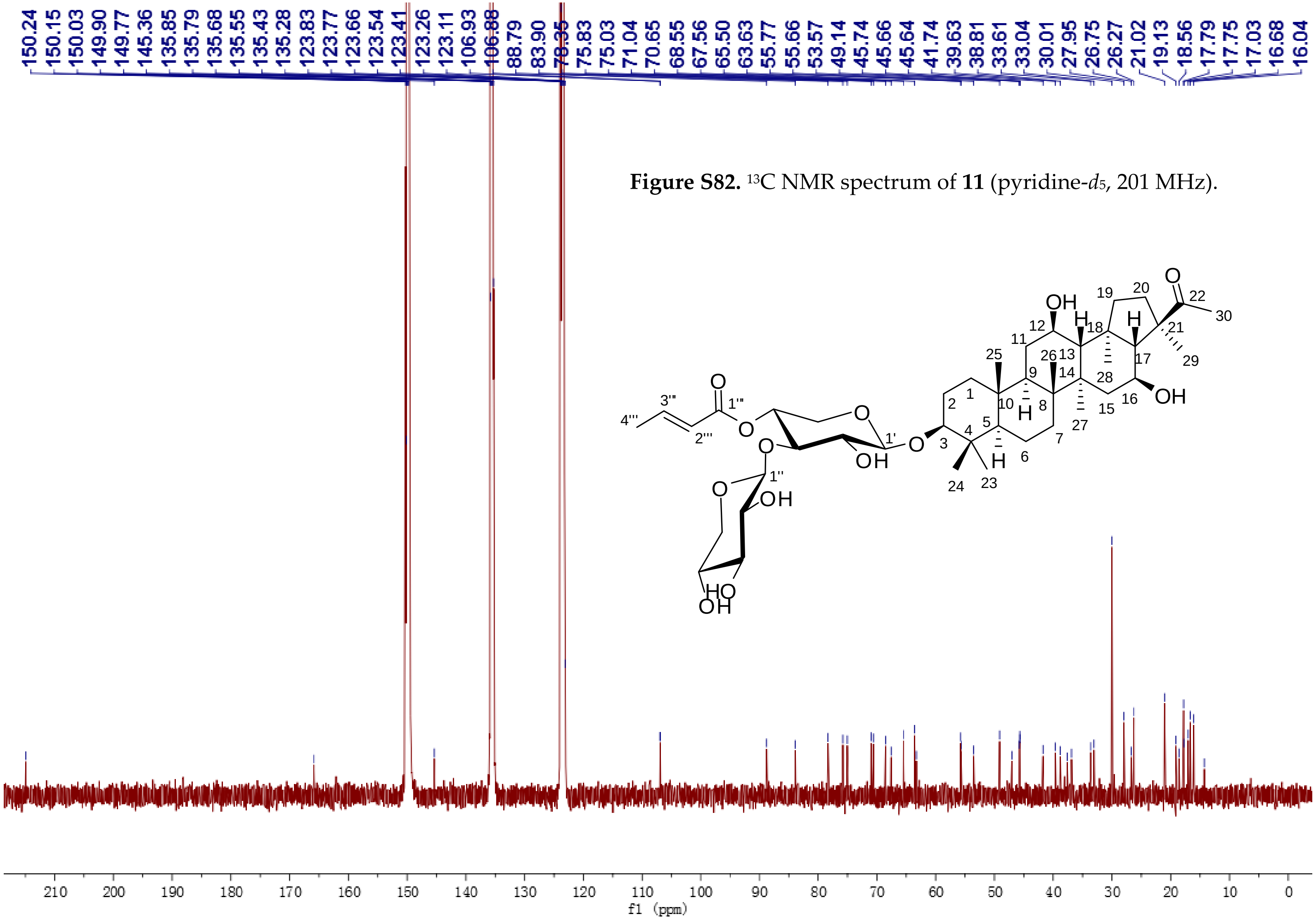

Figure S82. ^{13}C NMR spectrum of **11** (pyridine-d_5, 201 MHz).

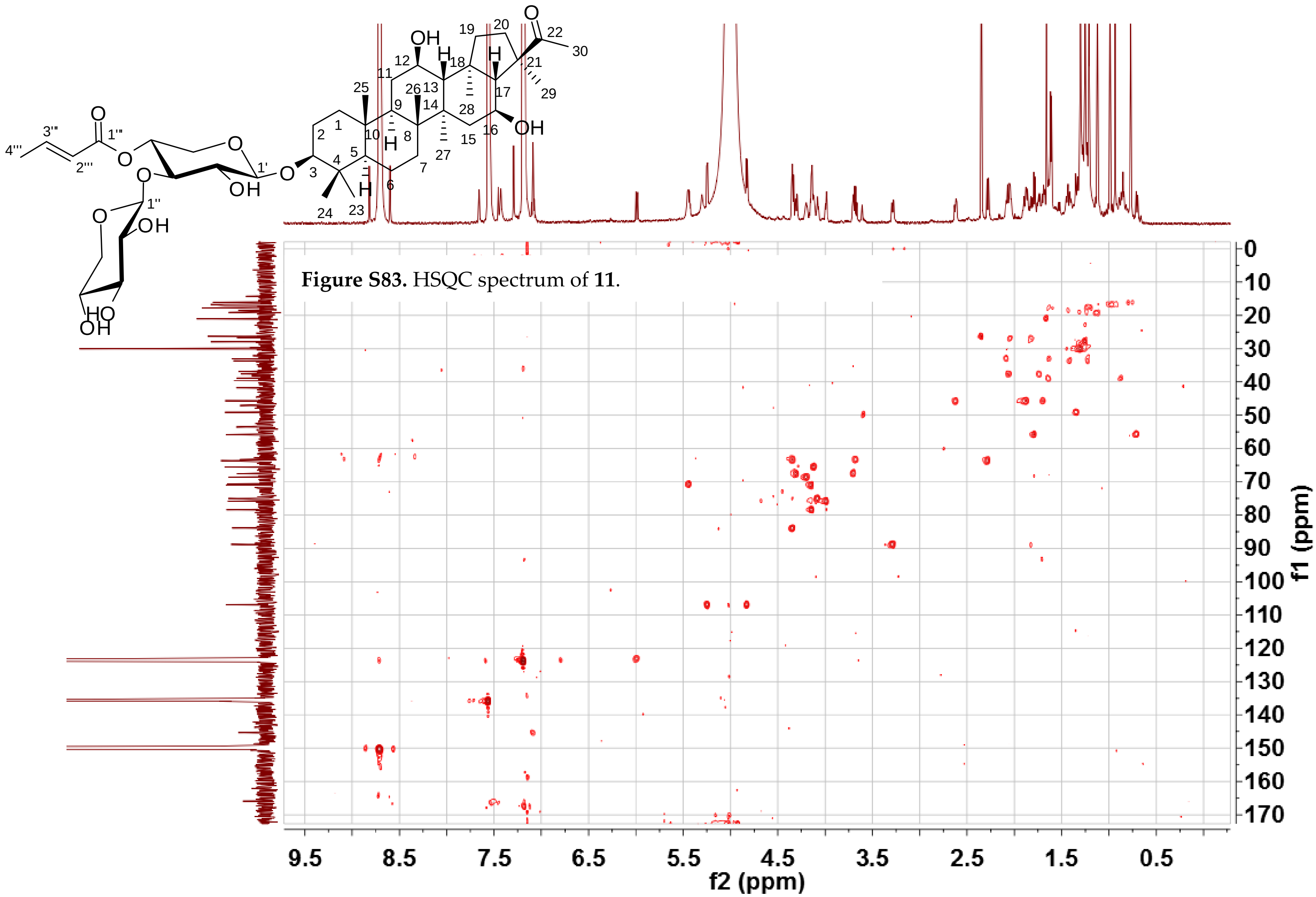

Figure S83. HSQC spectrum of **11**.

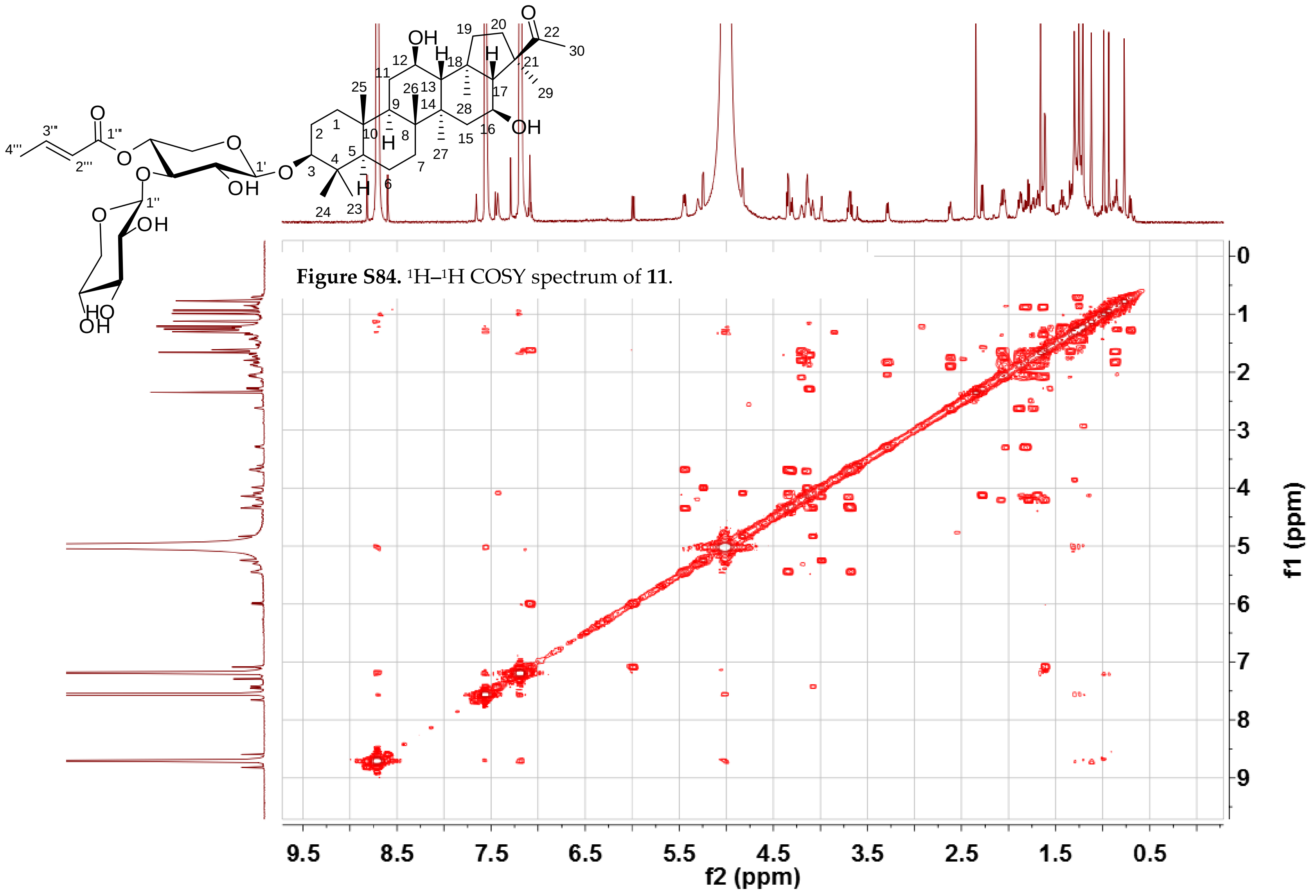

Figure S84. ¹H–¹H COSY spectrum of **11**.

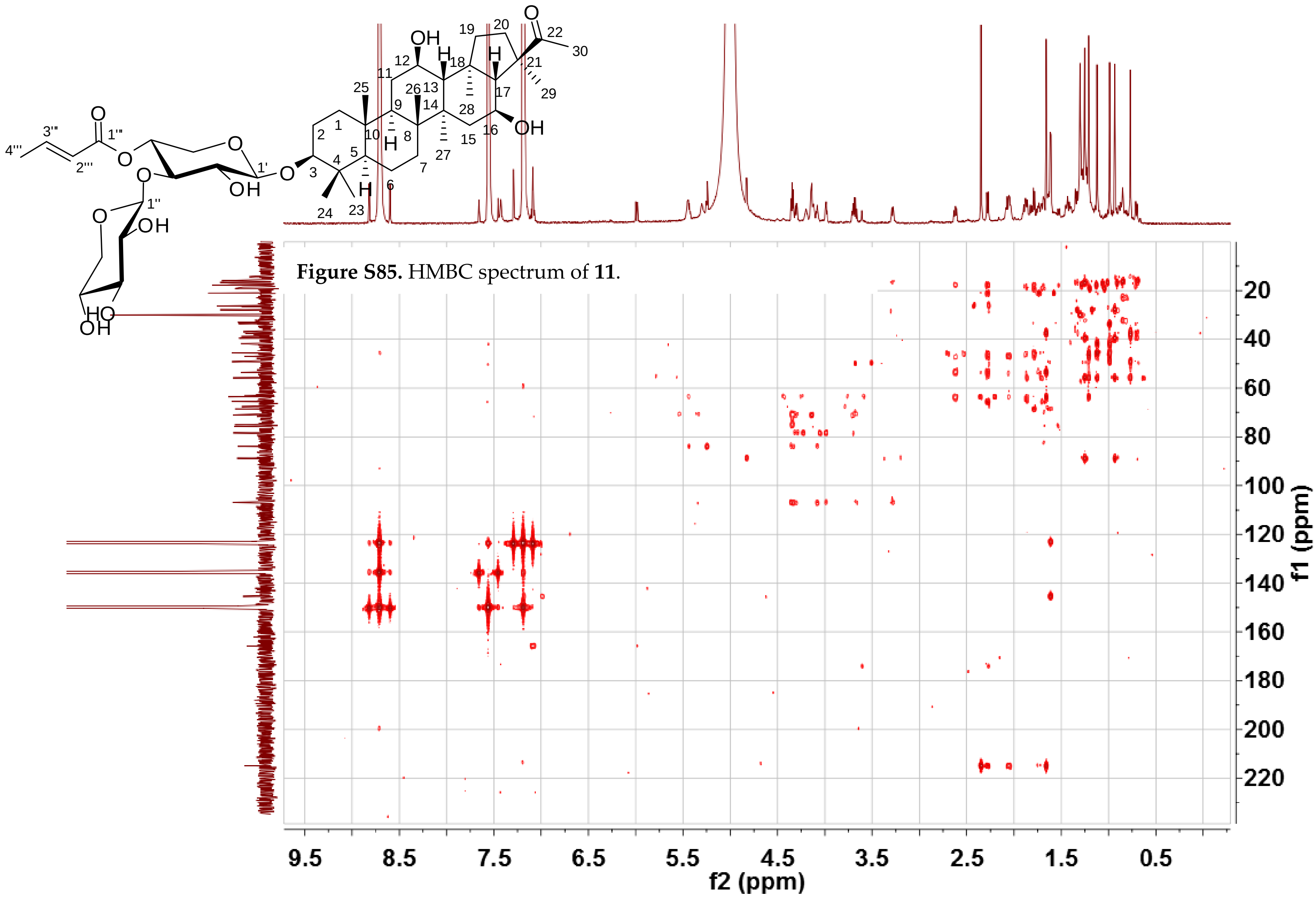

Figure S85. HMBC spectrum of **11**.

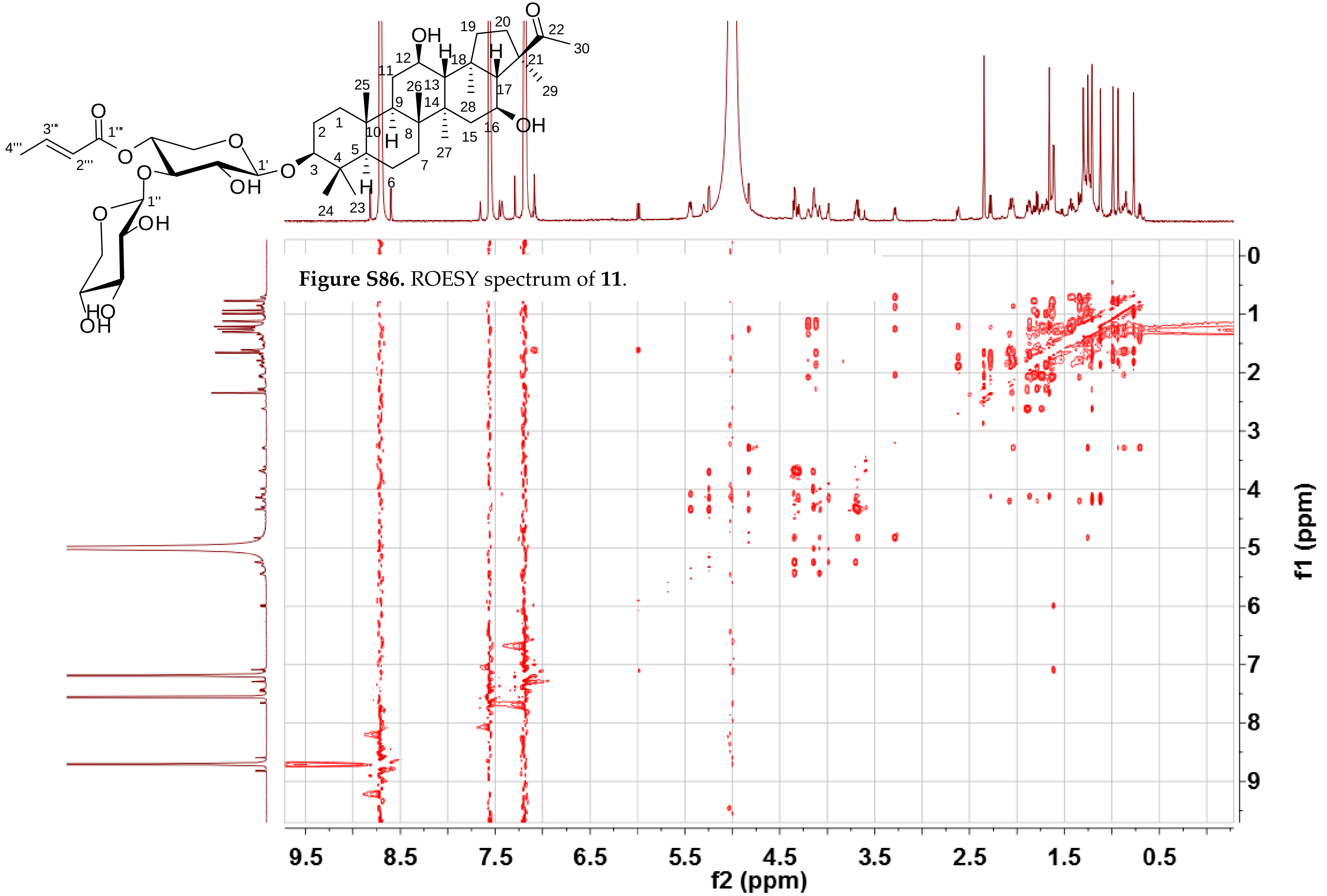

Figure S86. ROESY spectrum of **11**.

**Data Filename**	180409ESIA2.d	**Sample Name**	pdt 24
**Sample Type**	Sample	**Position**	
**Instrument Name**	Agilent G6230 TOF MS	**User Name**	KIB
**Acq Method**	ESI.m	**Acquired Time**	4/9/2018 10:13:33 AM
**IRM Calibration Status**	Success	**DA Method**	ESI.m
**Comment**			

**Sample Group**		**Info.**
**Acquisition SW Version**	6200 series TOF/6500 series Q-TOF B.05.01 (B5125.2)	

User Spectra

Fragmentor Voltage	Collision Energy	Ionization Mode
300	0	ESI

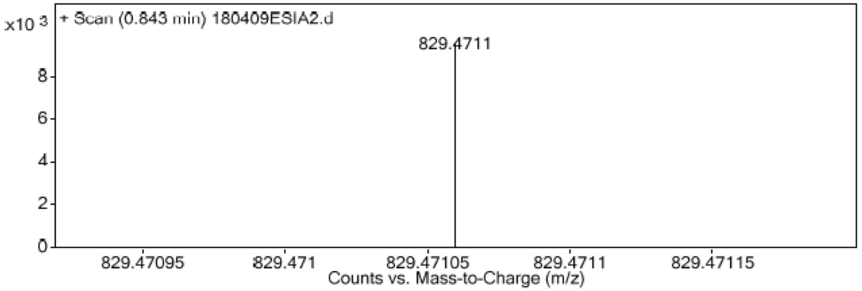

Figure S87. HRESIMS spectrum of **11**.

Peak List

m/z	z	Abund	Formula	Ion
106.086	1	5518.7		
437.1971	1	20021.2		
591.4976	1	6450.84		
619.5286		20222.86		
620.5325	2	8158.89		
647.5596	1	14319.98		
648.5633	1	6012.22		
829.4711	1	9490.67	C44 H70 Na O13	M+
922.0098	1	134531.05		
923.0134	1	23301.78		

Formula Calculator Element Limits

Element	Min	Max
C	0	200
H	0	400
O	10	15
Na	1	1

Formula Calculator Results

Formula	CalculatedMass	Mz	Diff.(mDa)	Diff. (ppm)	DBE
C44 H70 Na O13	829.4714	829.4711	0.3	0.4	9.5

--- End Of Report ---

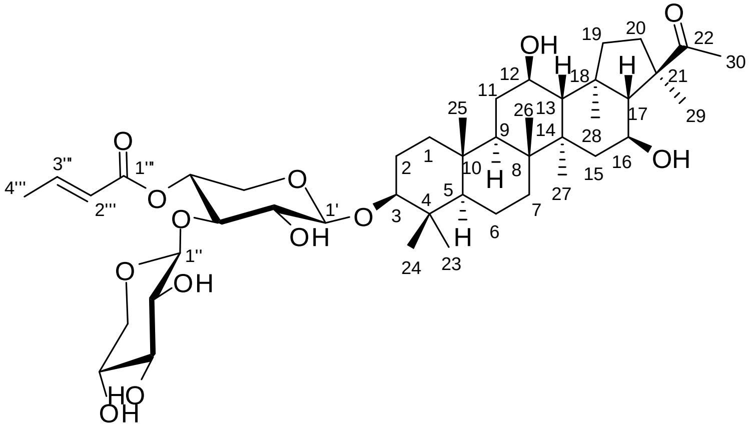

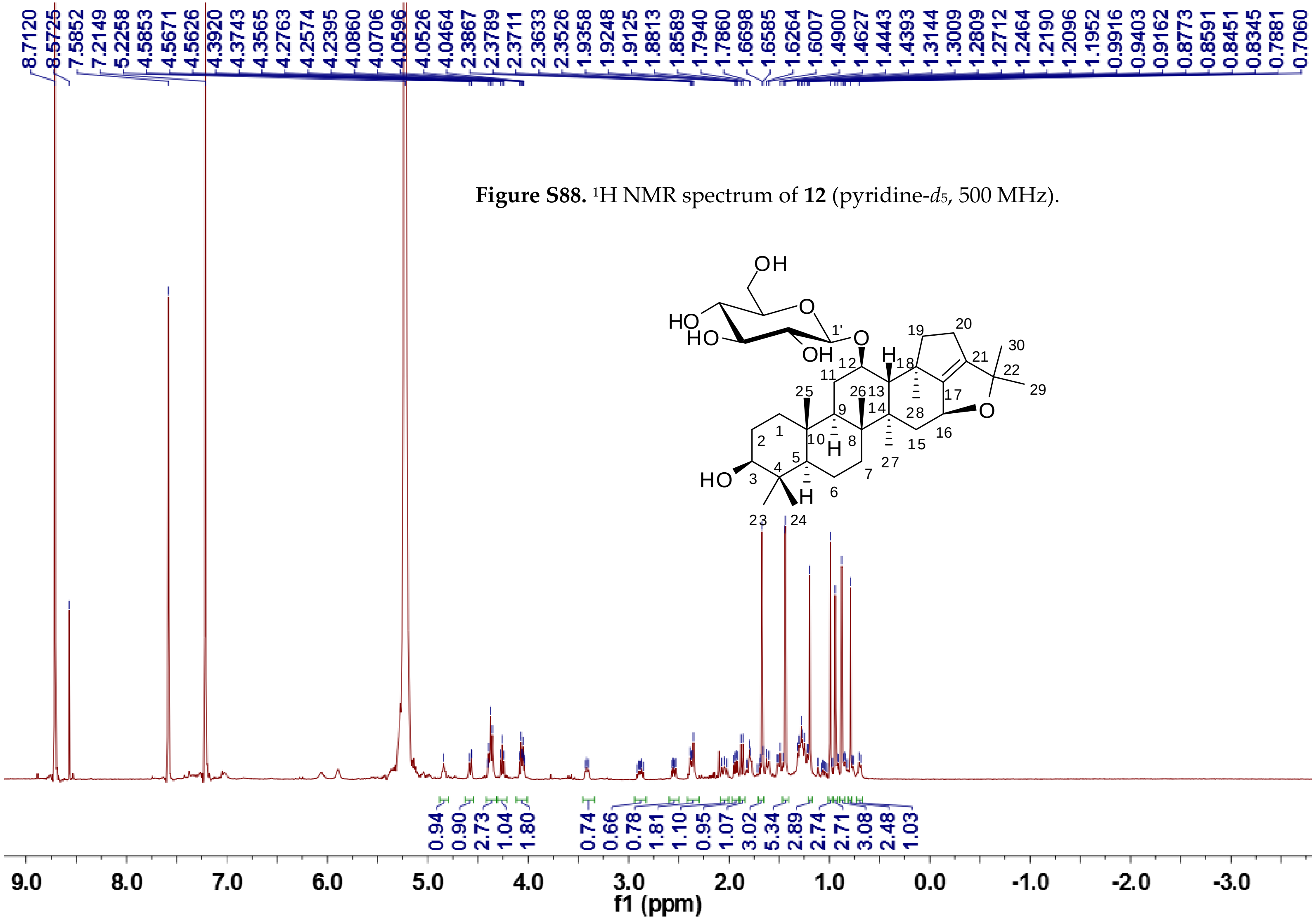

Figure S88. ^{1}H NMR spectrum of **12** (pyridine-d_5, 500 MHz).

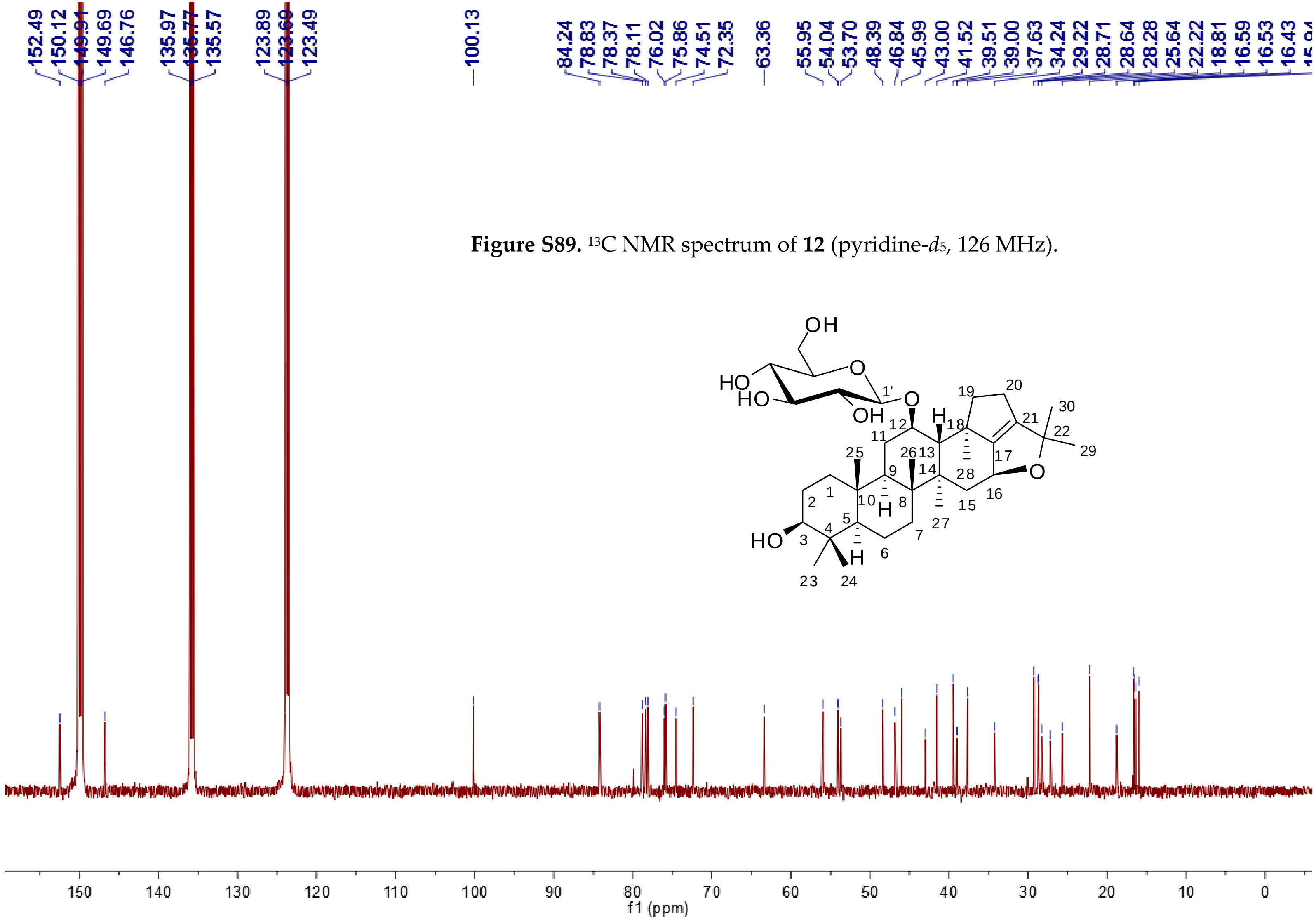

Figure S89. ^{13}C NMR spectrum of **12** (pyridine-d_5, 126 MHz).

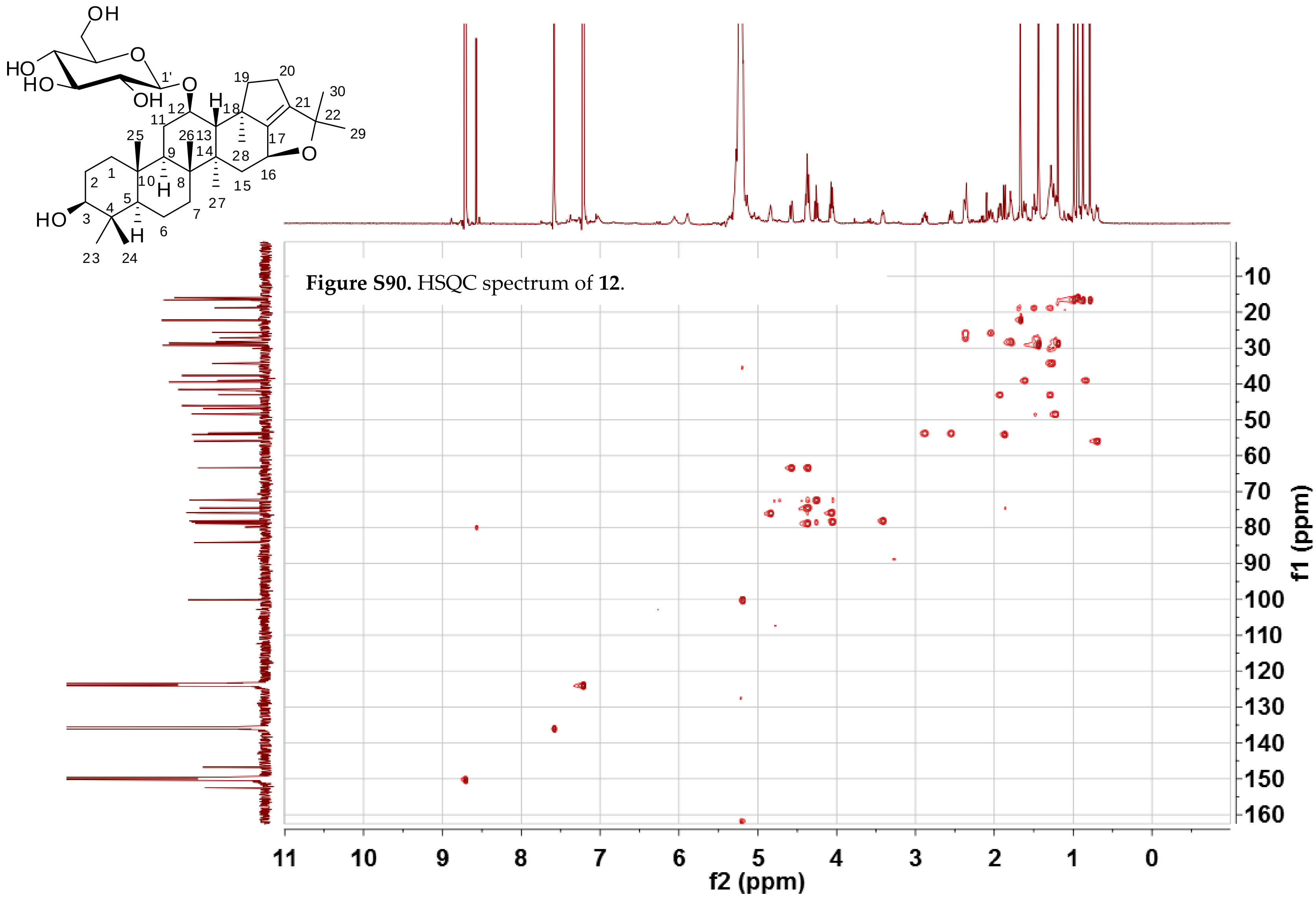

Figure S90. HSQC spectrum of **12**.

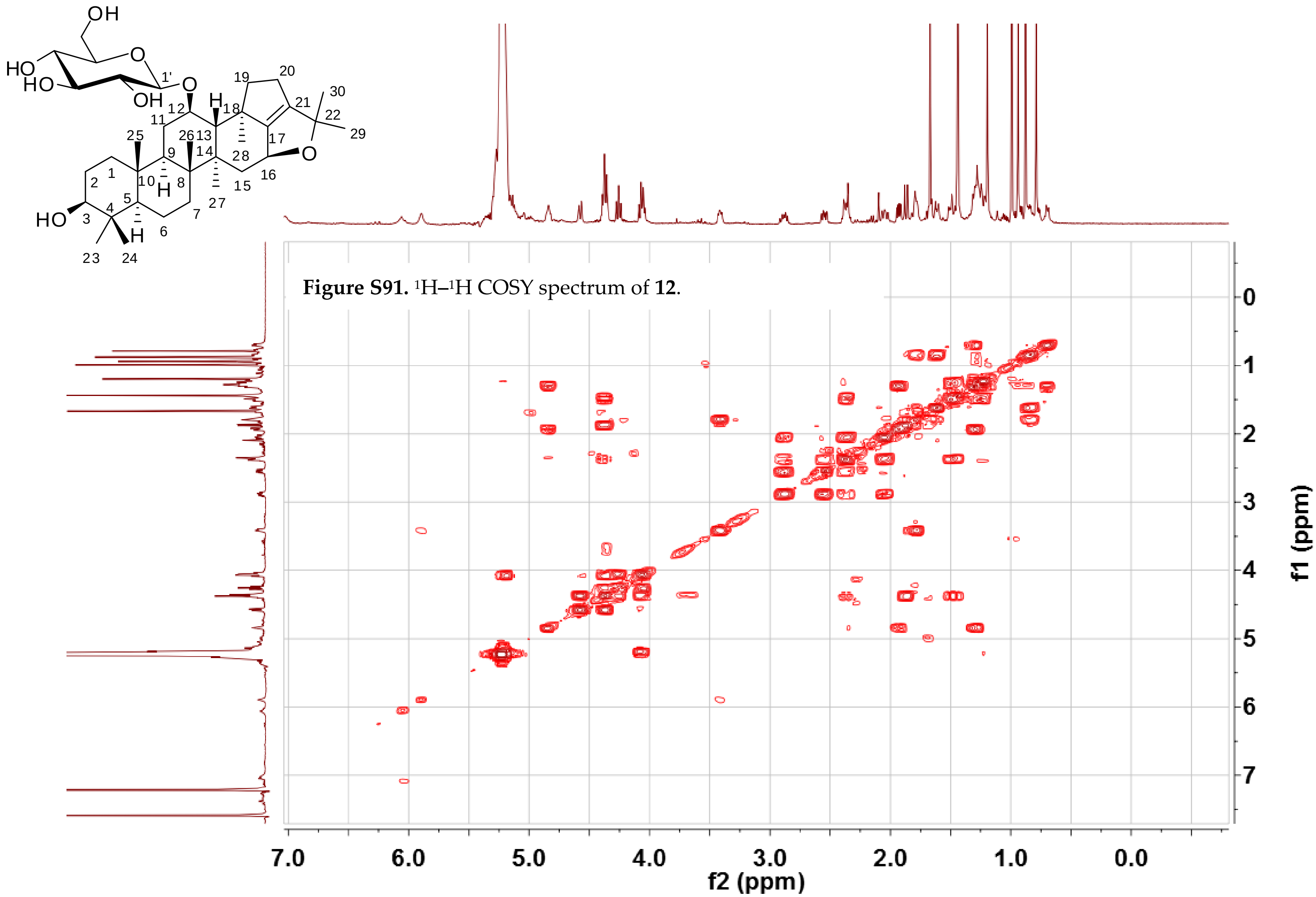

Figure S91. ^{1}H–^{1}H COSY spectrum of **12**.

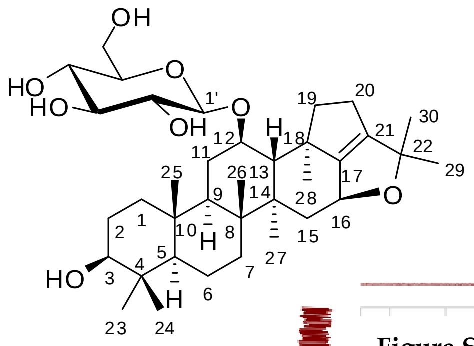

Figure S92. HMBC spectrum of **12**.

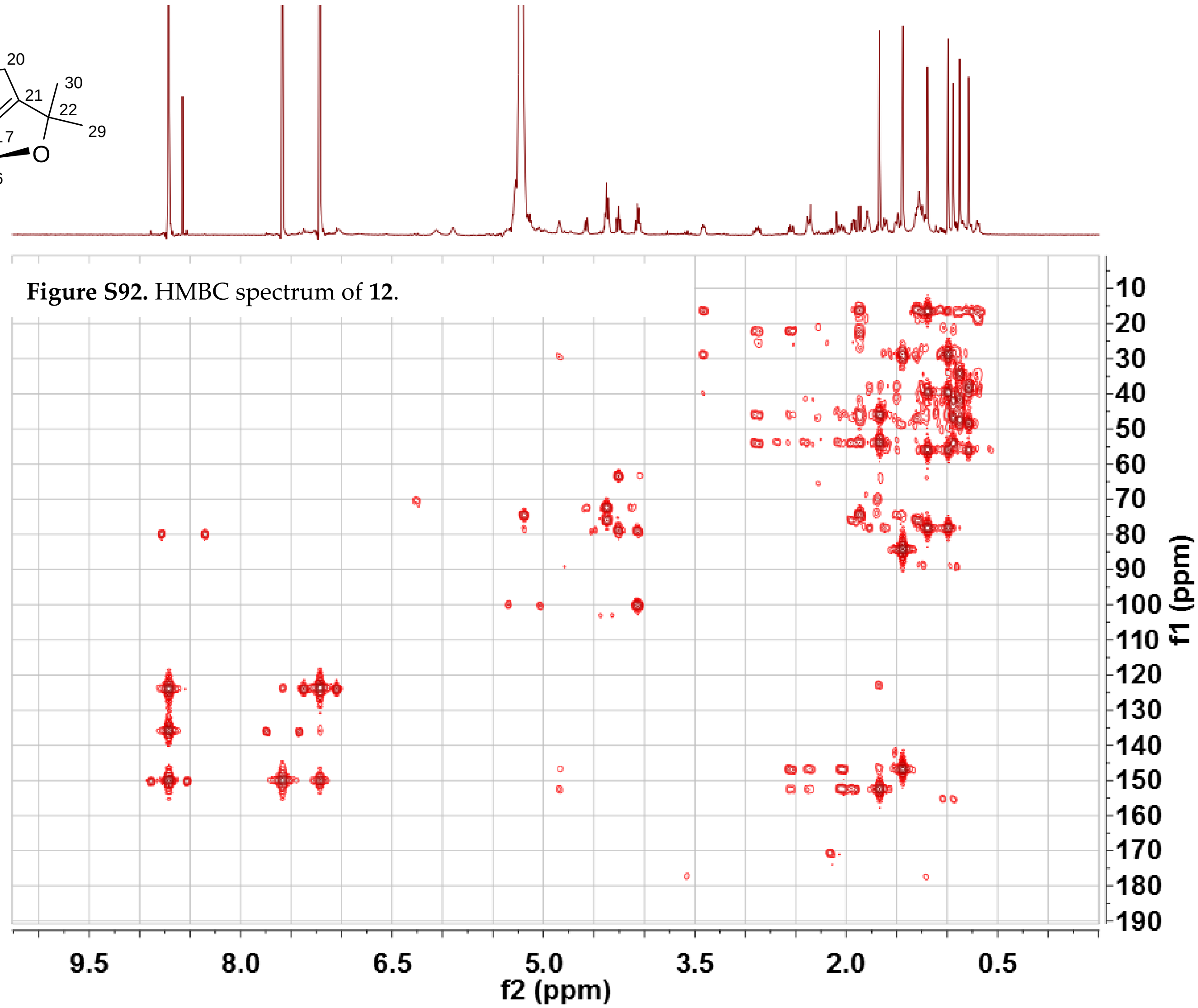

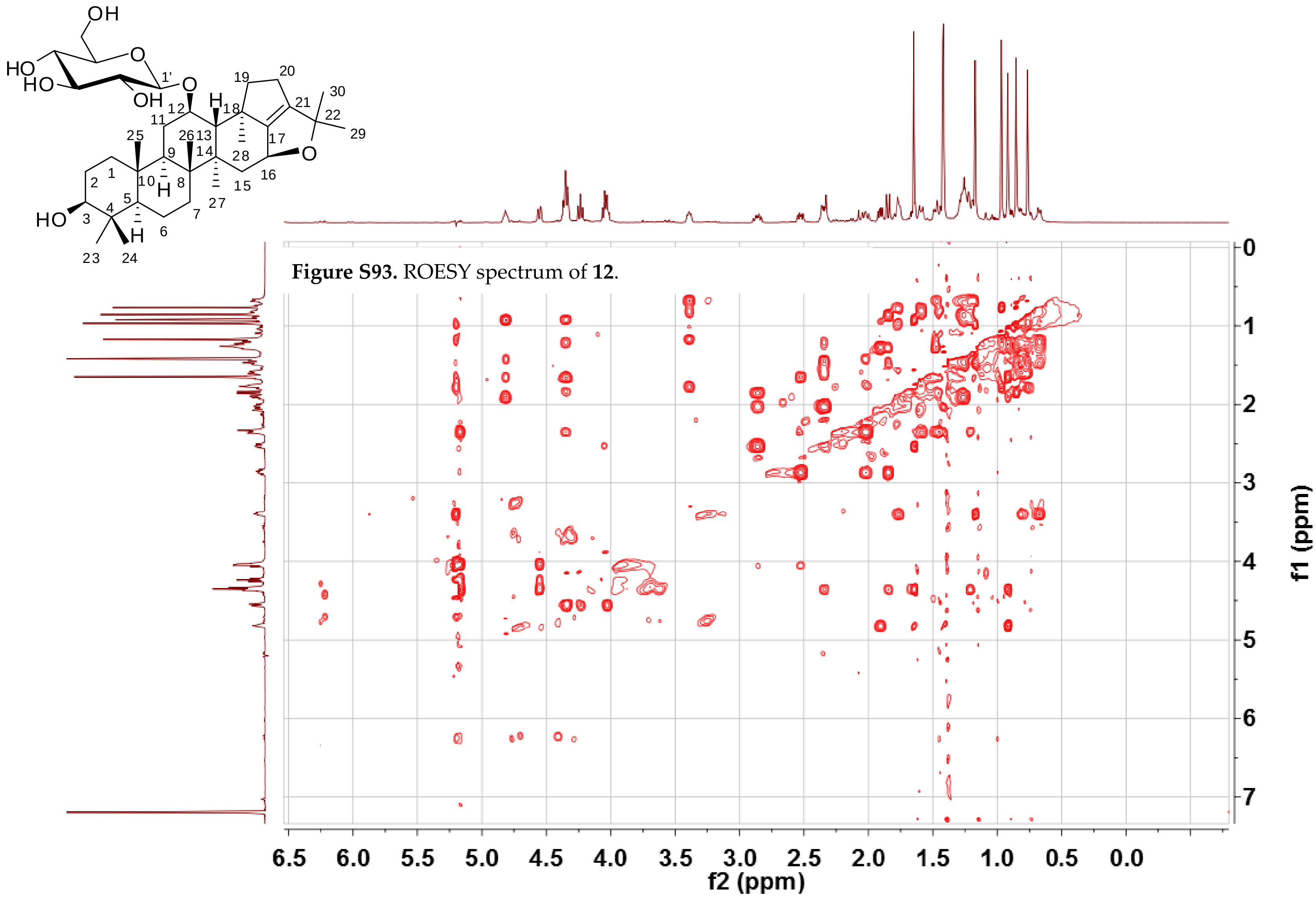

Figure S93. ROESY spectrum of **12**.

Figure S94. HRESIMS spectrum of **12**.

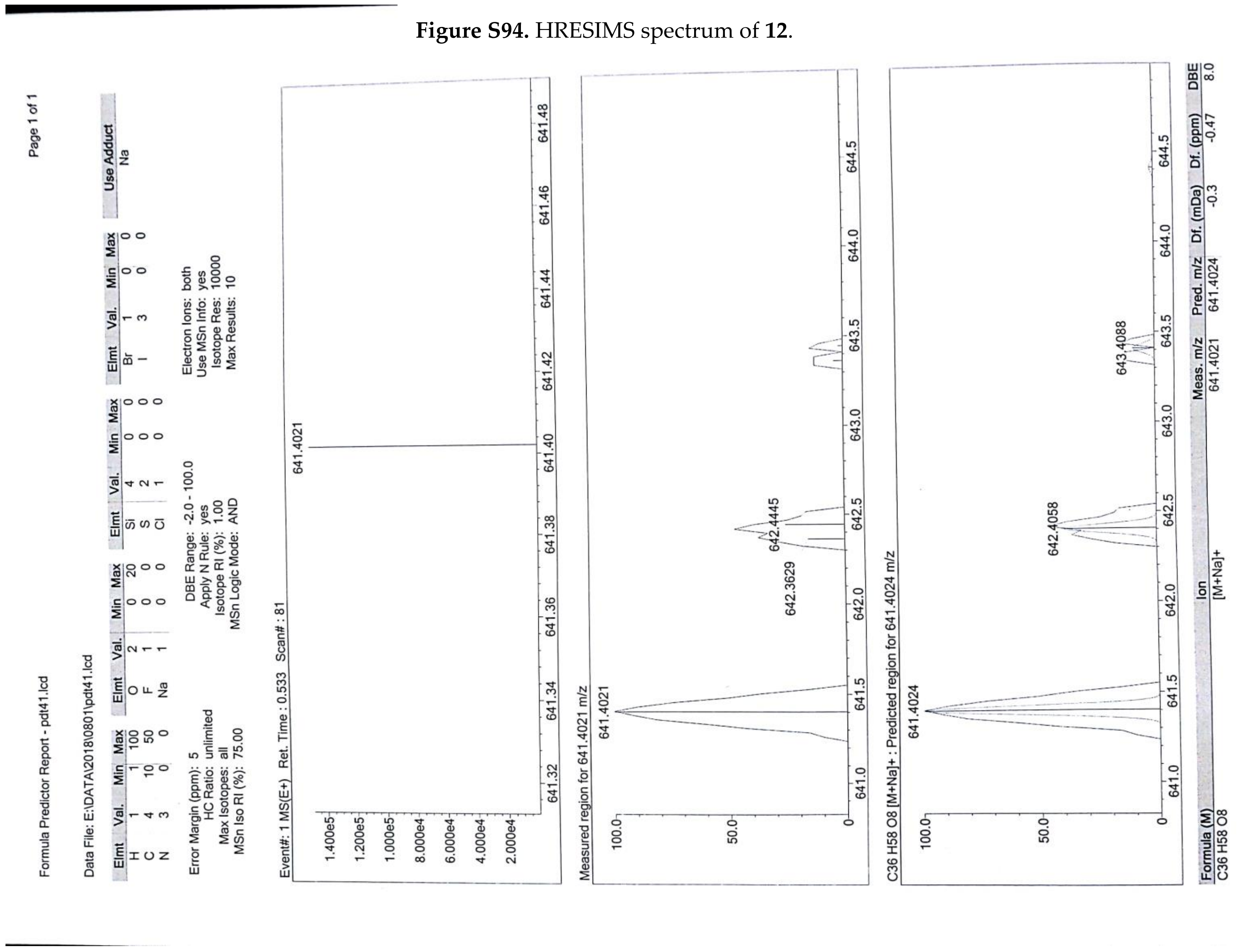

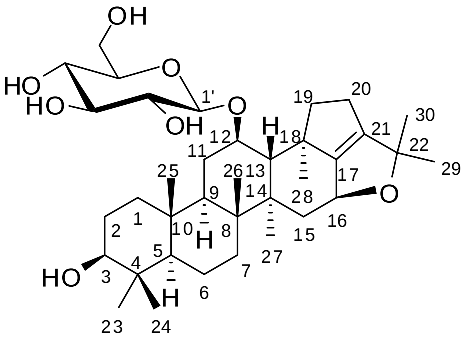

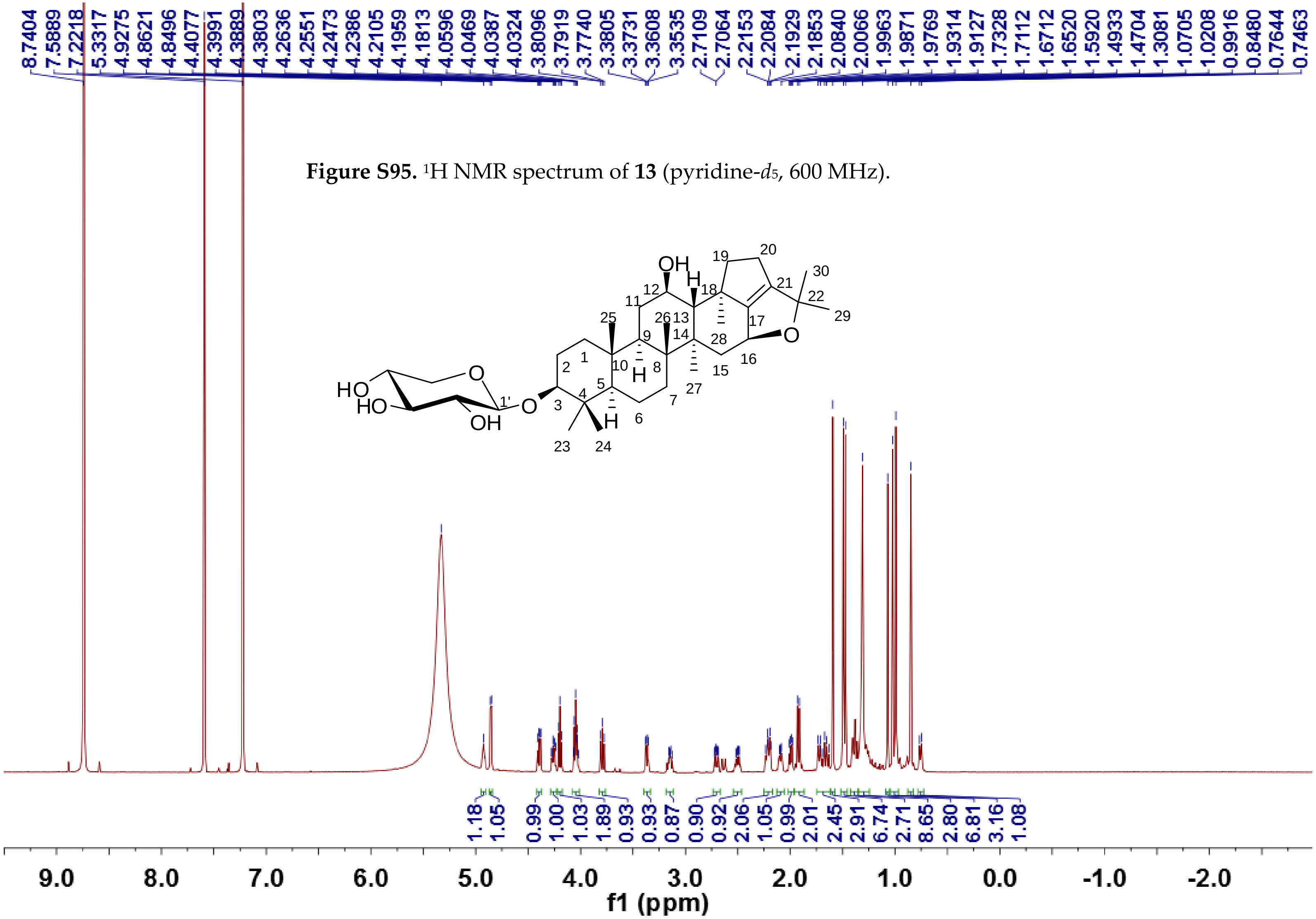

Figure S95. ¹H NMR spectrum of **13** (pyridine-*d*5, 600 MHz).

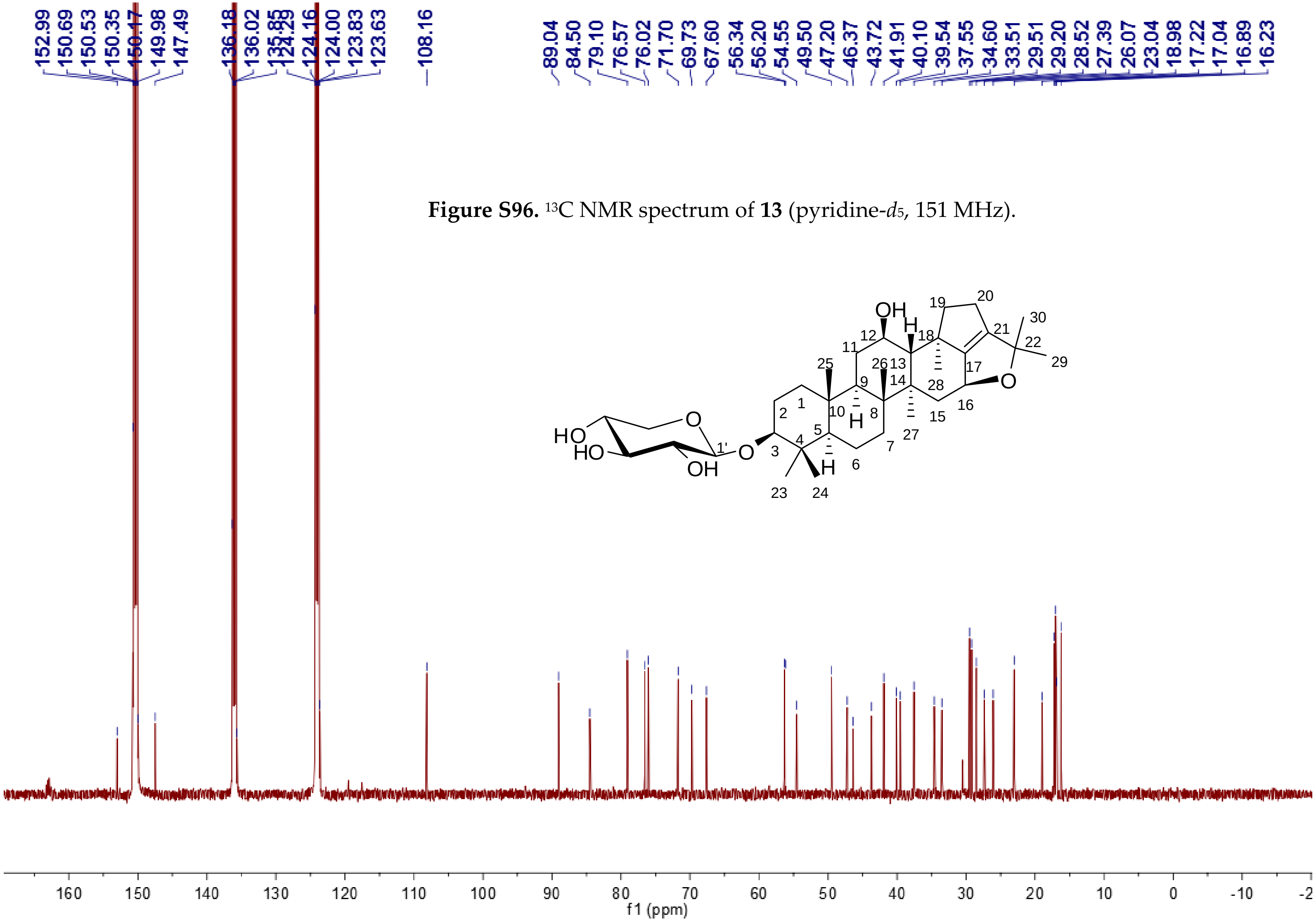

Figure S96. ^{13}C NMR spectrum of **13** (pyridine-*d*$_5$, 151 MHz).

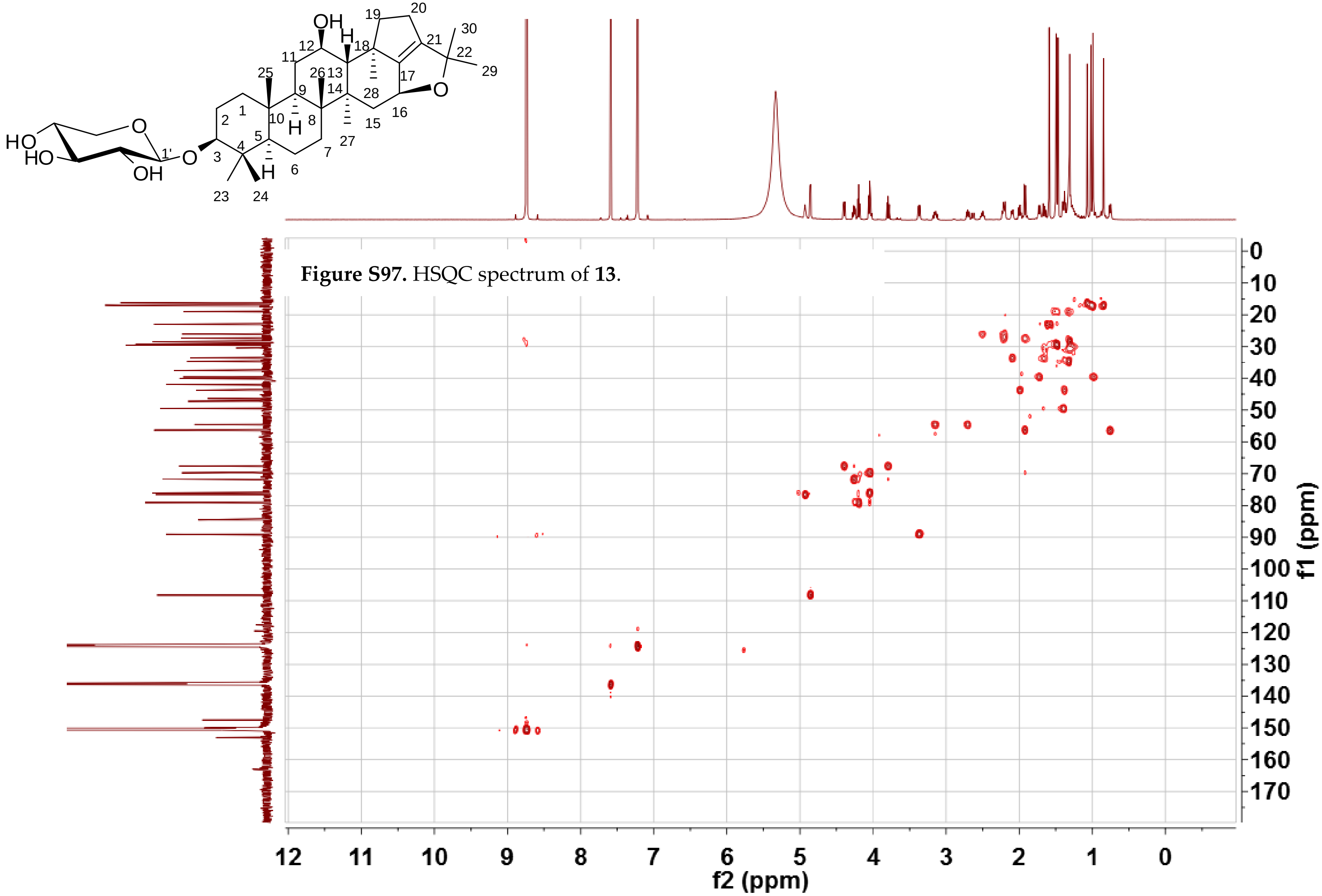

Figure S97. HSQC spectrum of **13**.

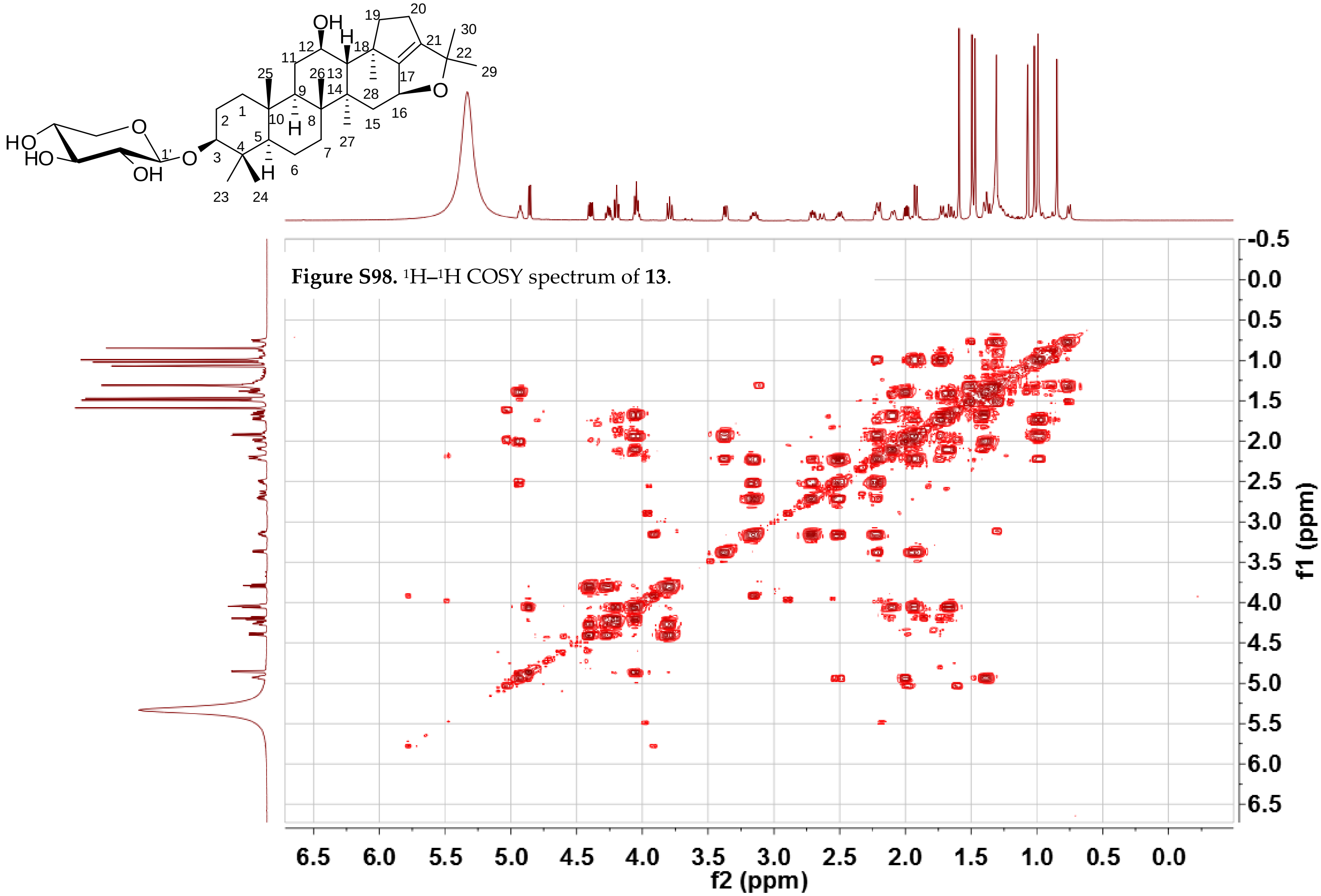

Figure S98. ^{1}H–^{1}H COSY spectrum of **13**.

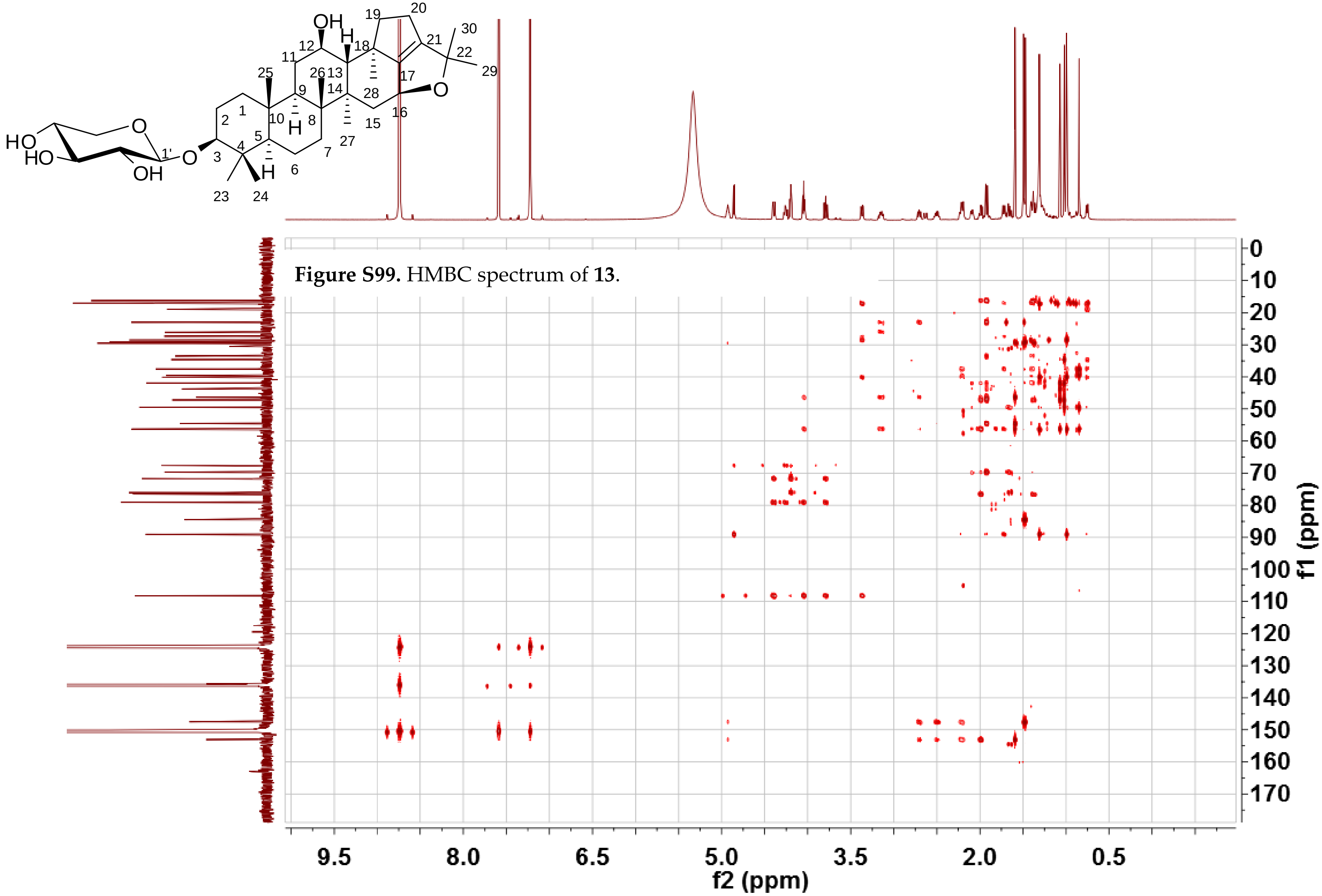

Figure S99. HMBC spectrum of **13**.

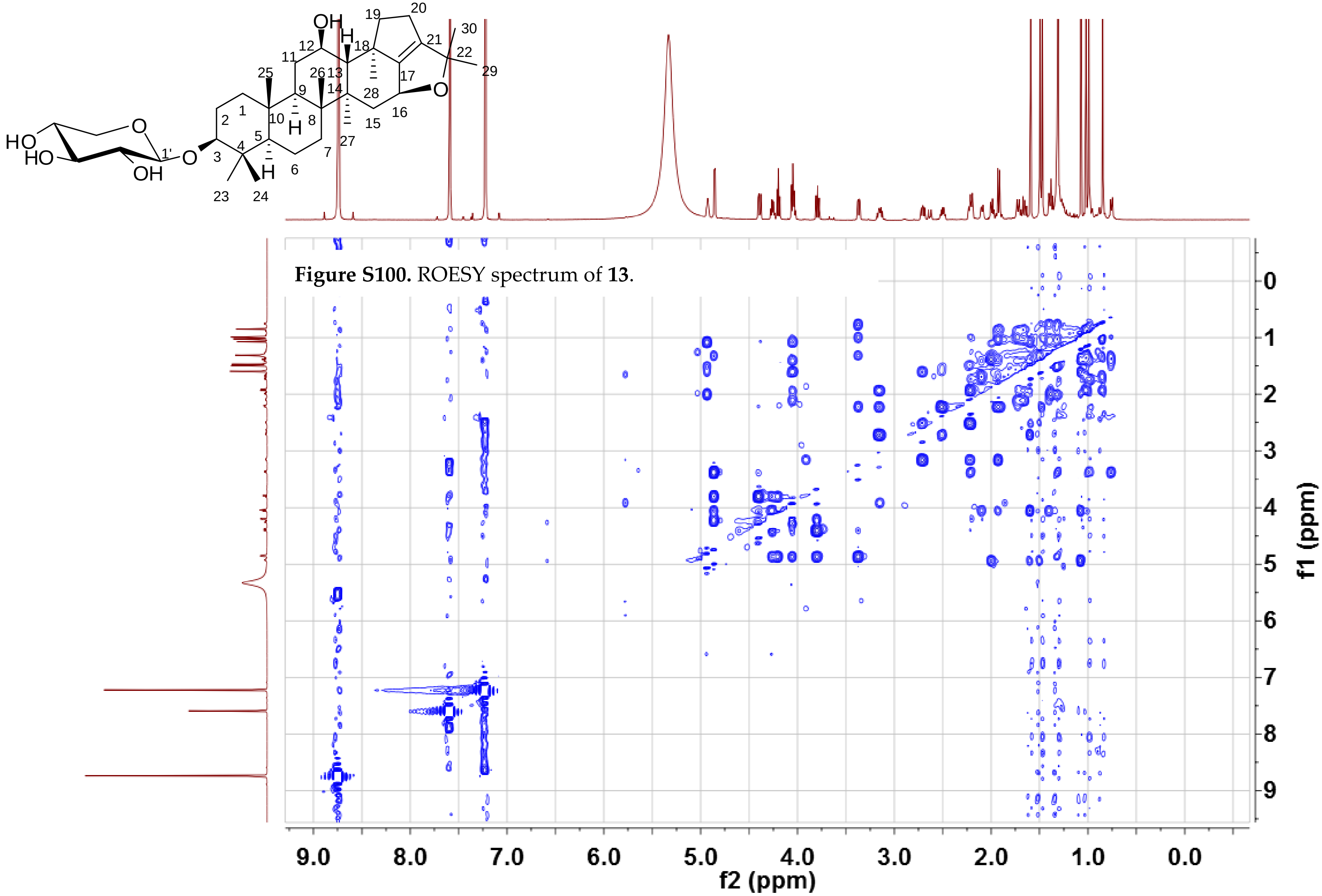

Figure S100. ROESY spectrum of **13**.

**Data Filename**	180824ESIA4.d	**Sample Name**	pdt 54a
**Sample Type**	Sample	**Position**	
**Instrument Name**	Agilent G6230 TOF MS	**User Name**	KIB
**Acq Method**	ESI.m	**Acquired Time**	8/24/2018 2:17:49 PM
**IRM Calibration Status**	Success	**DA Method**	ESI.m
**Comment**			

Sample Group **Info.**

Acquisition SW Version 6200 series TOF/6500 series Q-TOF B.05.01 (B5125.2)

User Spectra

Fragmentor Voltage	Collision Energy	Ionization Mode
260	0	ESI

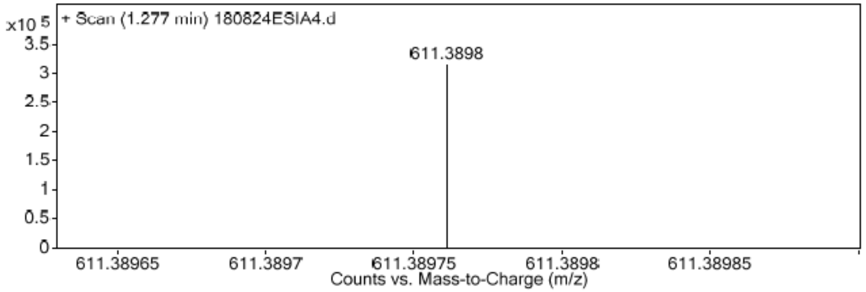

Figure S101. HRESIMS spectrum of **13**.

Peak List

m/z	z	Abund	Formula	Ion
136.9188		104846.75		
230.905		112693.76		
611.3898	1	314712.94	C35 H56 Na O7	M+
612.3924	1	113185.77	C35 H56 Na O7	M+
627.3625	1	84164.83		
687.3748	1	88003.44		
741.3473	1	410123.28		
742.3507	1	159524.27		
1329.7461	1	95505.48		
1330.7493	1	82139.52		

Formula Calculator Element Limits

Element	Min	Max
C	0	200
H	0	400
O	3	10
Na	1	1

Formula Calculator Results

Formula	CalculatedMass	Mz	Diff.(mDa)	Diff. (ppm)	DBE
C35 H56 Na O7	611.3924	611.3898	2.6	4.2	7.5

--- End Of Report ---

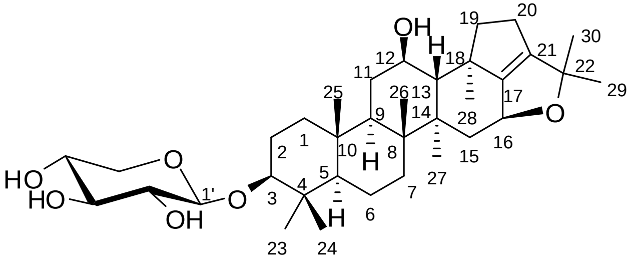

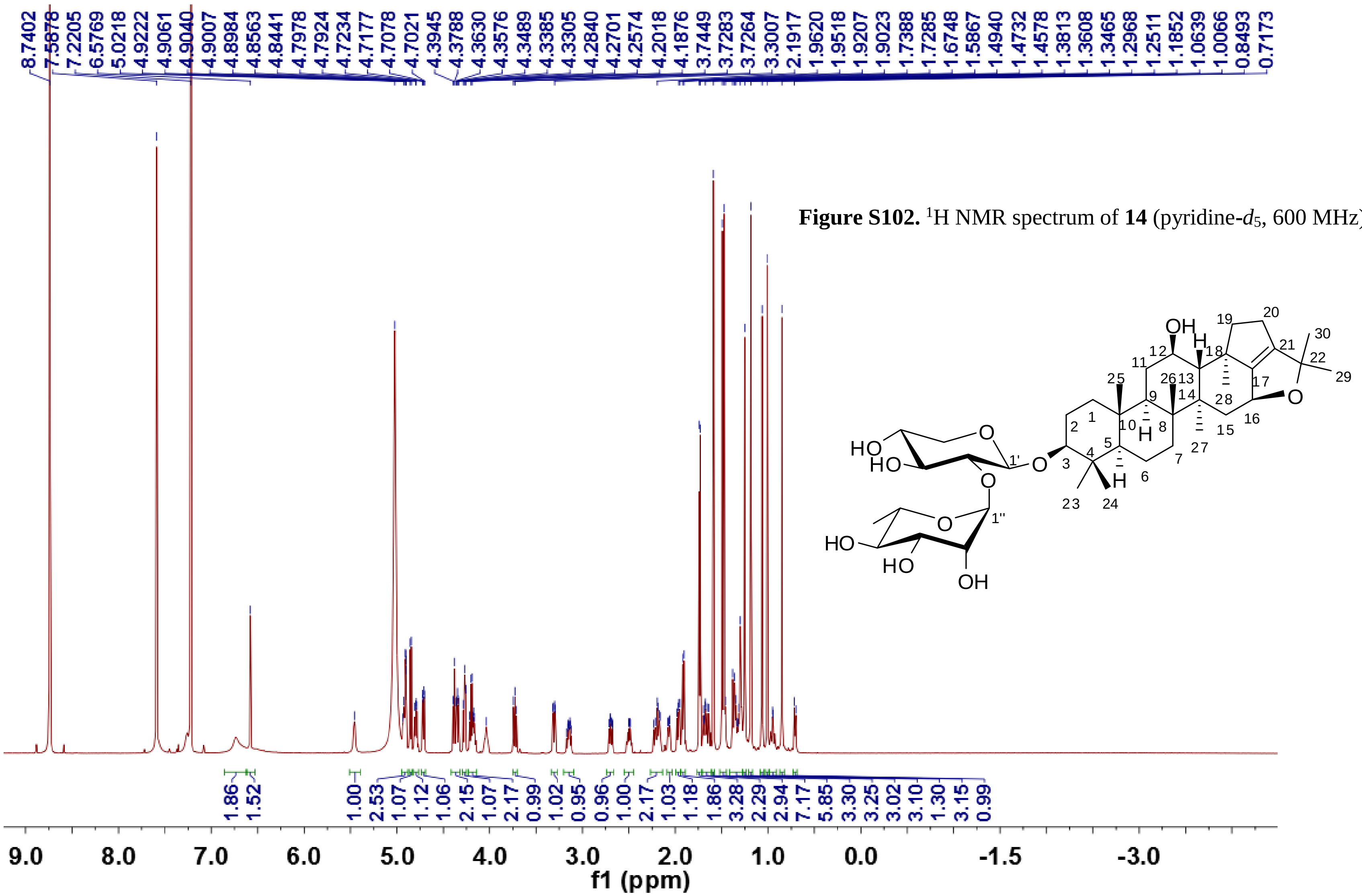

Figure S102. ^{1}H NMR spectrum of **14** (pyridine-d_5, 600 MHz).

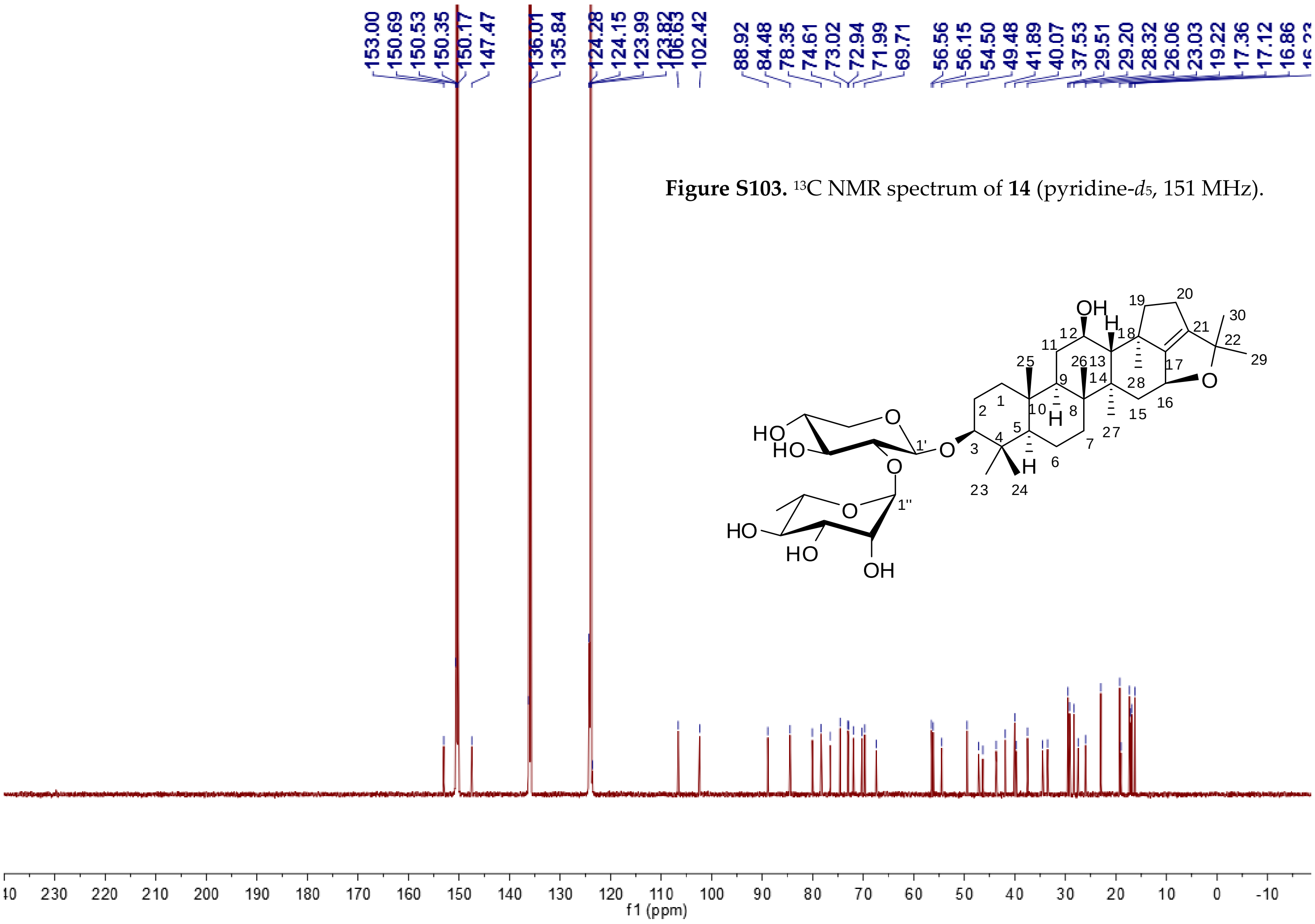

Figure S103. ^{13}C NMR spectrum of **14** (pyridine-d_5, 151 MHz).

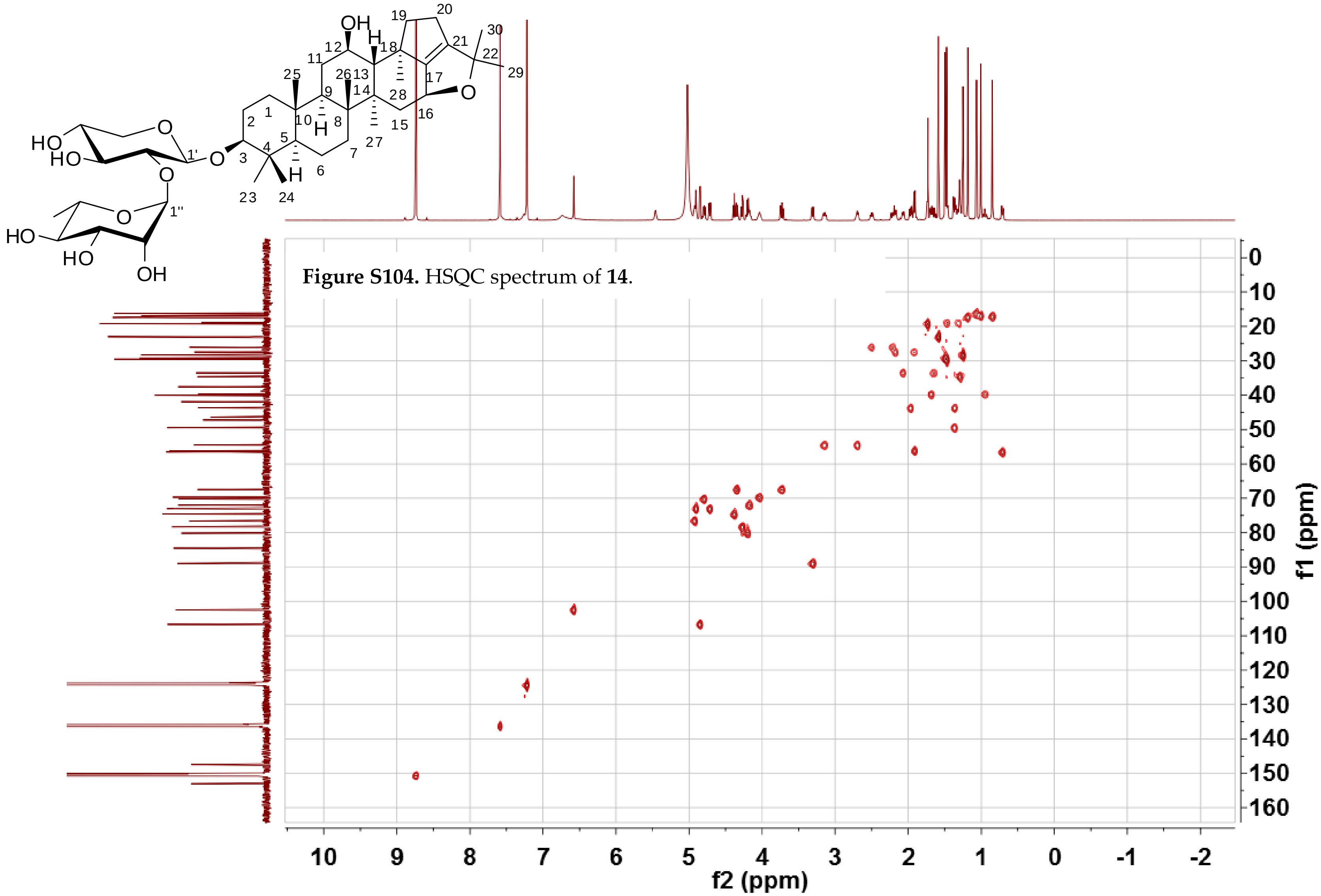

Figure S104. HSQC spectrum of **14**.

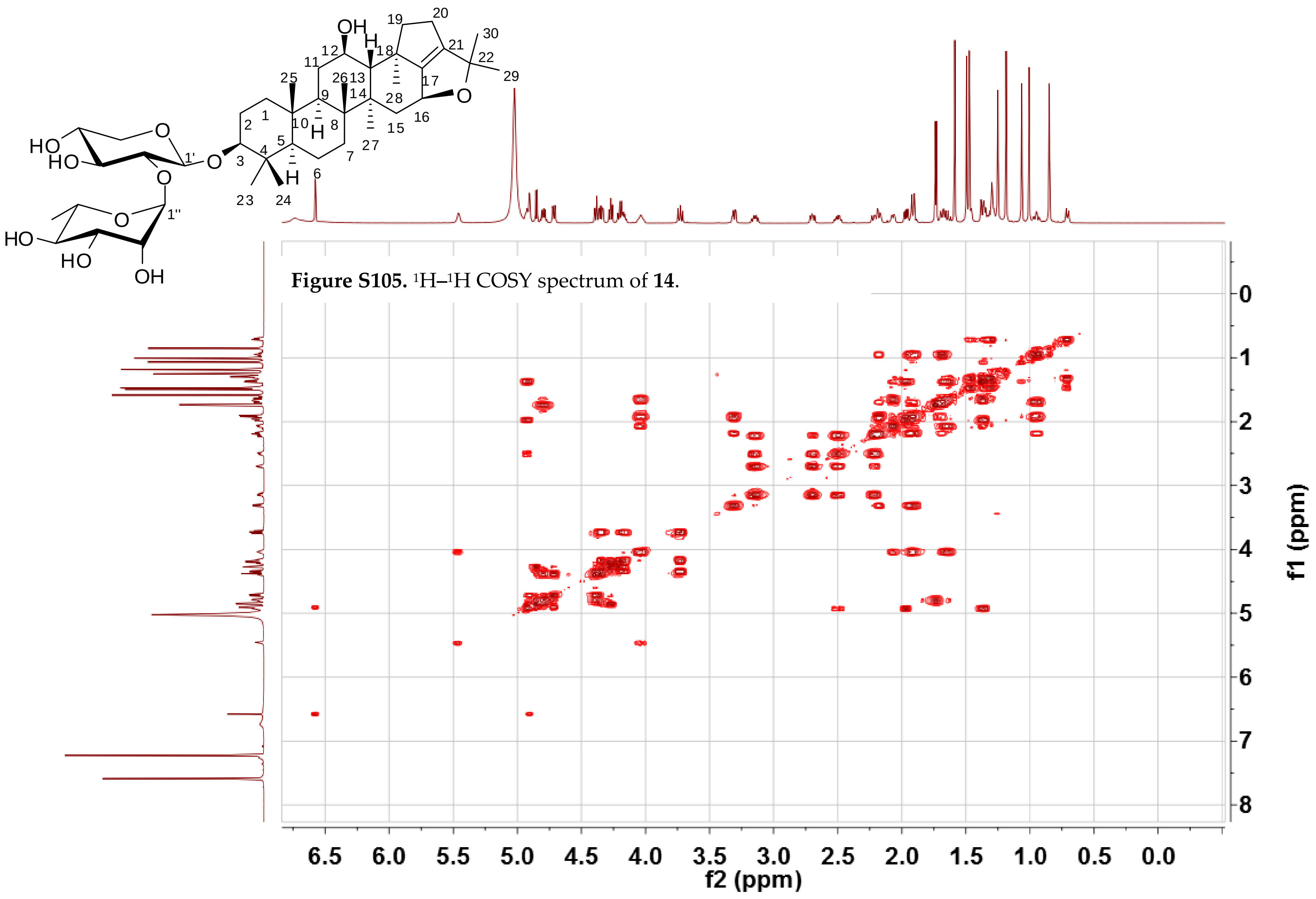

Figure S105. ¹H–¹H COSY spectrum of **14**.

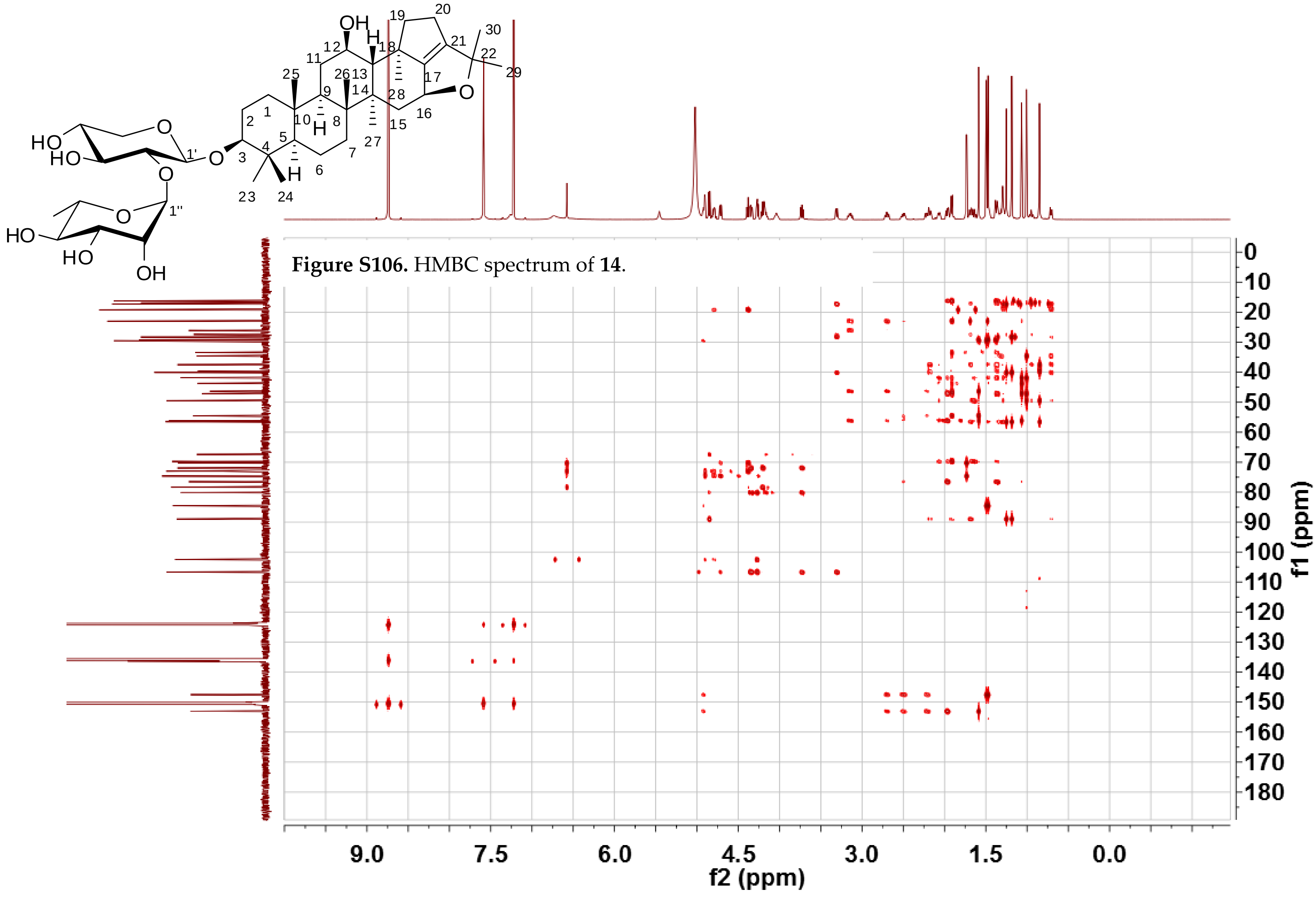

Figure S106. HMBC spectrum of **14**.

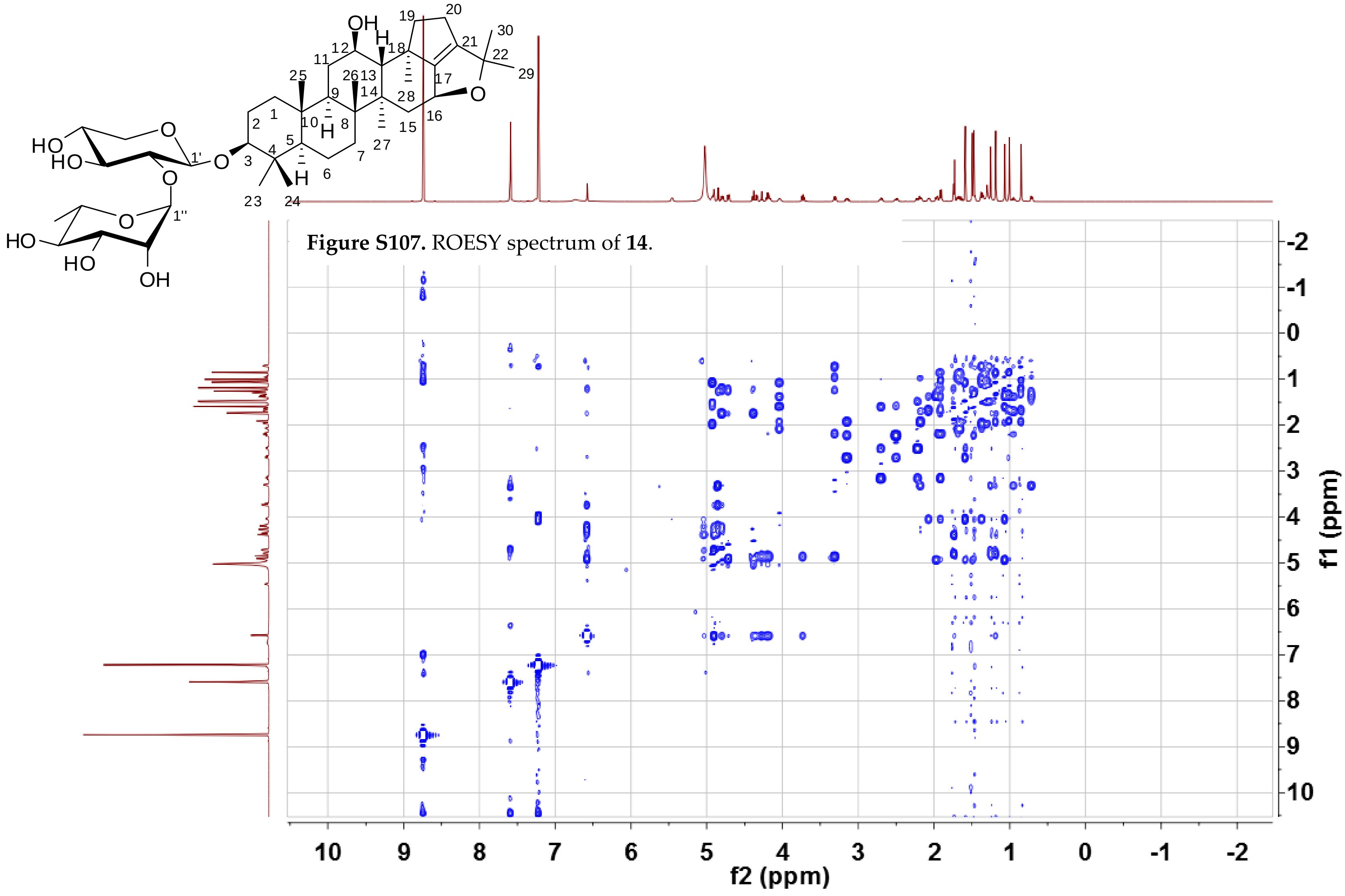

Figure S107. ROESY spectrum of **14**.

**Data Filename**	180828ESIA5.d	**Sample Name**	pdt53
**Sample Type**	Sample	**Position**	
**Instrument Name**	Agilent G6230 TOF MS	**User Name**	KIB
**Acq Method**	ESI.m	**Acquired Time**	8/28/2018 9:58:56 AM
**IRM Calibration Status**	Success	**DA Method**	ESI.m
**Comment**			

**Sample Group**		**Info.**
**Acquisition SW Version**	6200 series TOF/6500 series Q-TOF B.05.01 (B5125.2)	

User Spectra

Fragmentor Voltage	Collision Energy	Ionization Mode
200	0	ESI

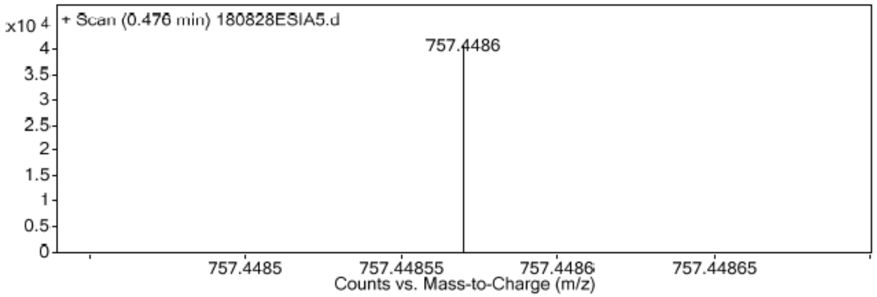

Peak List

m/z	z	Abund	Formula	Ion
112.1872	1	21524.53		
145.2493	1	27599.95		
274.2731	1	24295.63		
318.2995	1	25232.83		
619.5259	1	26303.13		
757.4486	1	40351.51	C41 H66 Na O11	M+
1188.0319	1	28121.41		
1189.0342	1	21680.37		
1216.0623	1	25862.58		
1217.066	1	21227.8		

Formula Calculator Element Limits

Element	Min	Max
C	0	200
H	0	400
O	7	15
Na	1	1

Formula Calculator Results

Formula	CalculatedMass	Mz	Diff.(mDa)	Diff. (ppm)	DBE
C41 H66 Na O11	757.4503	757.4486	1.7	2.2	8.5

--- End Of Report ---

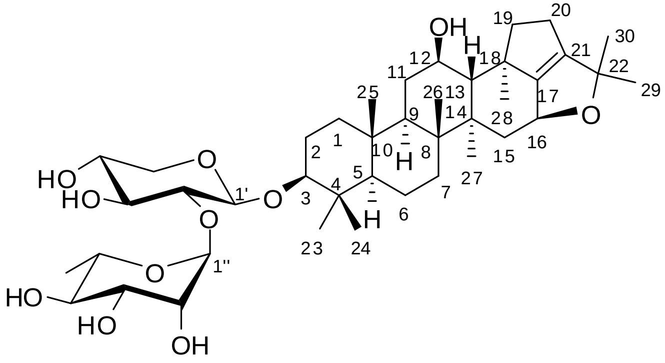

Figure S108. HRESIMS spectrum of **14**.

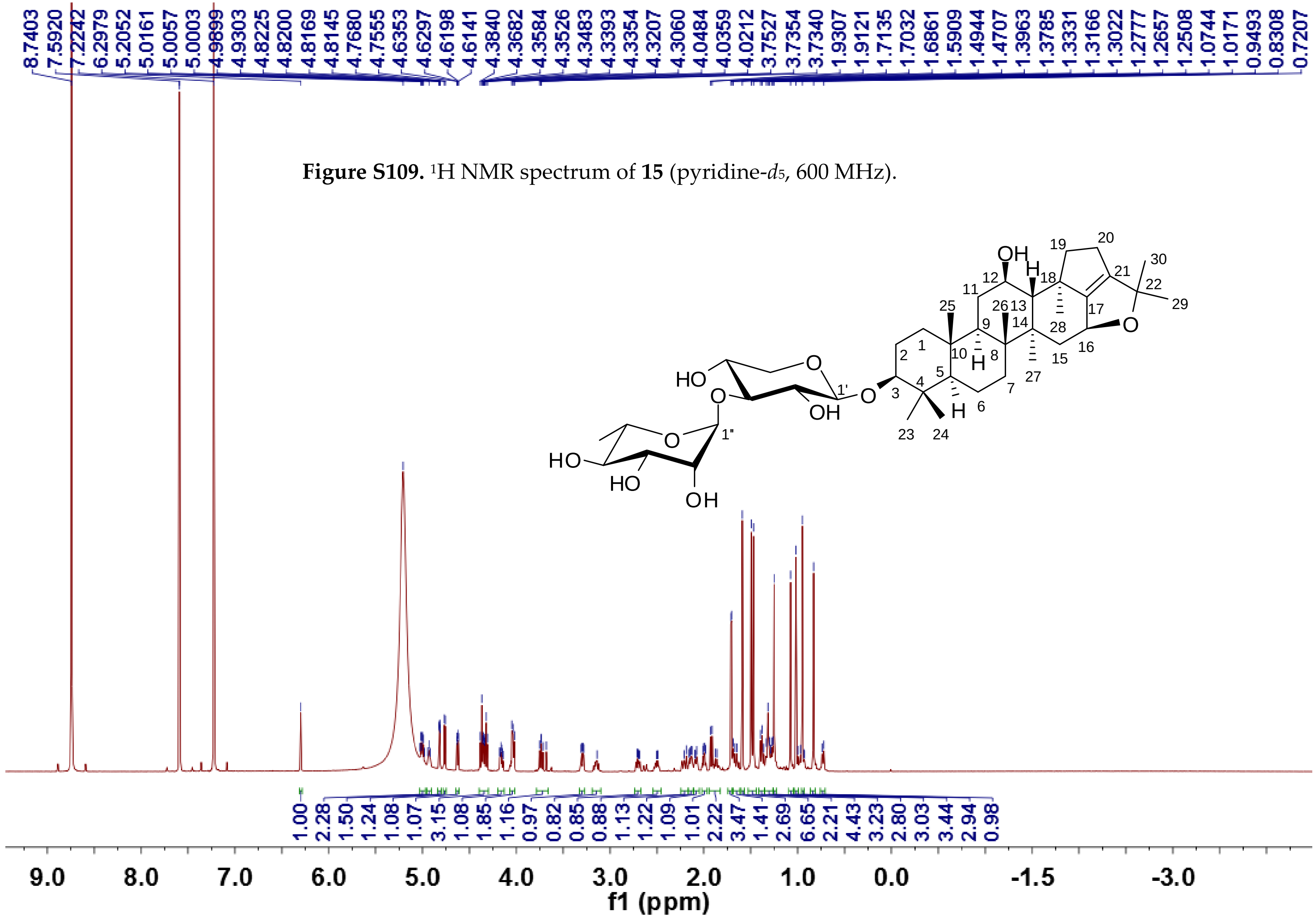

Figure S109. ^{1}H NMR spectrum of **15** (pyridine-d_5, 600 MHz).

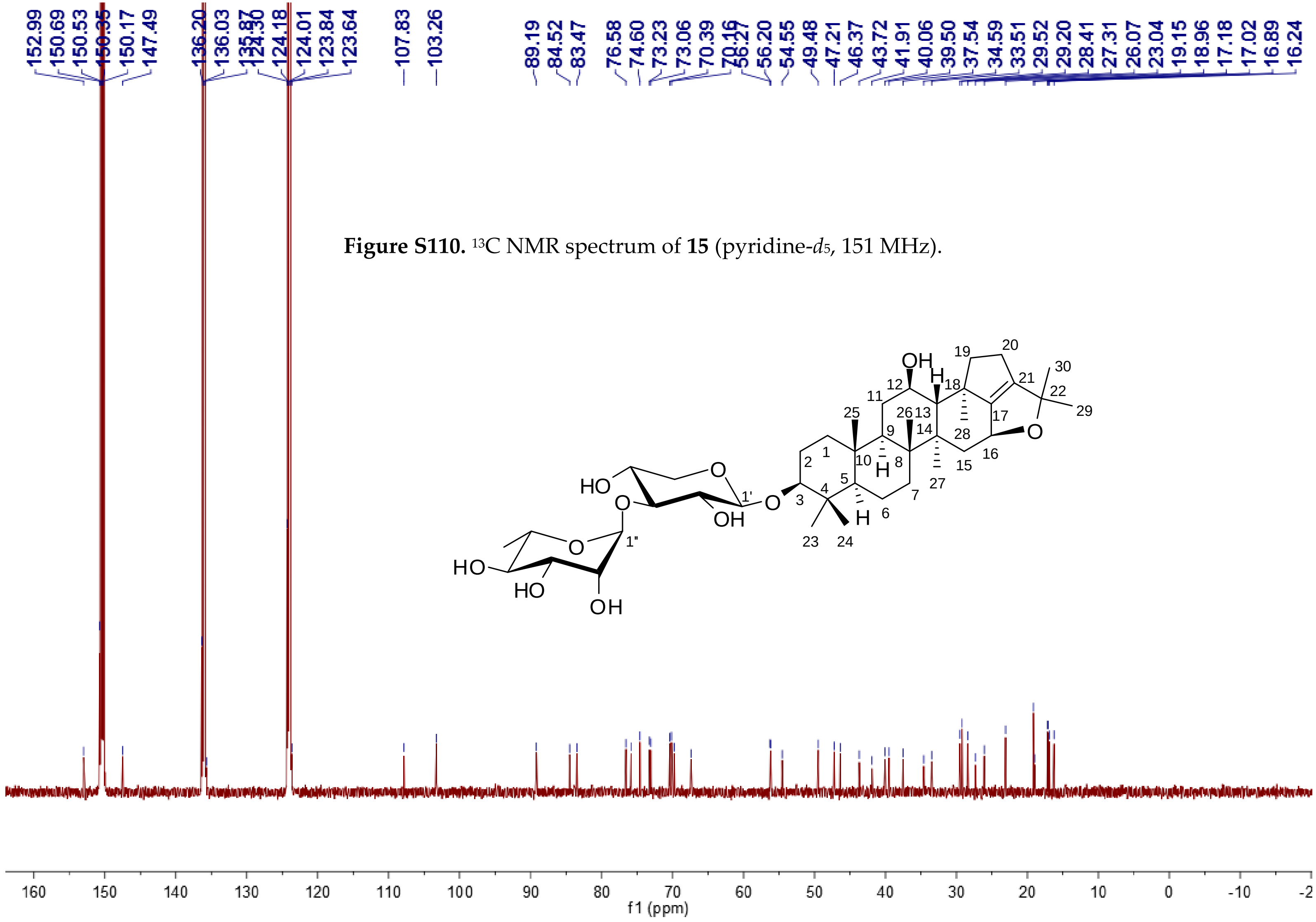

Figure S110. ^{13}C NMR spectrum of **15** (pyridine-d_5, 151 MHz).

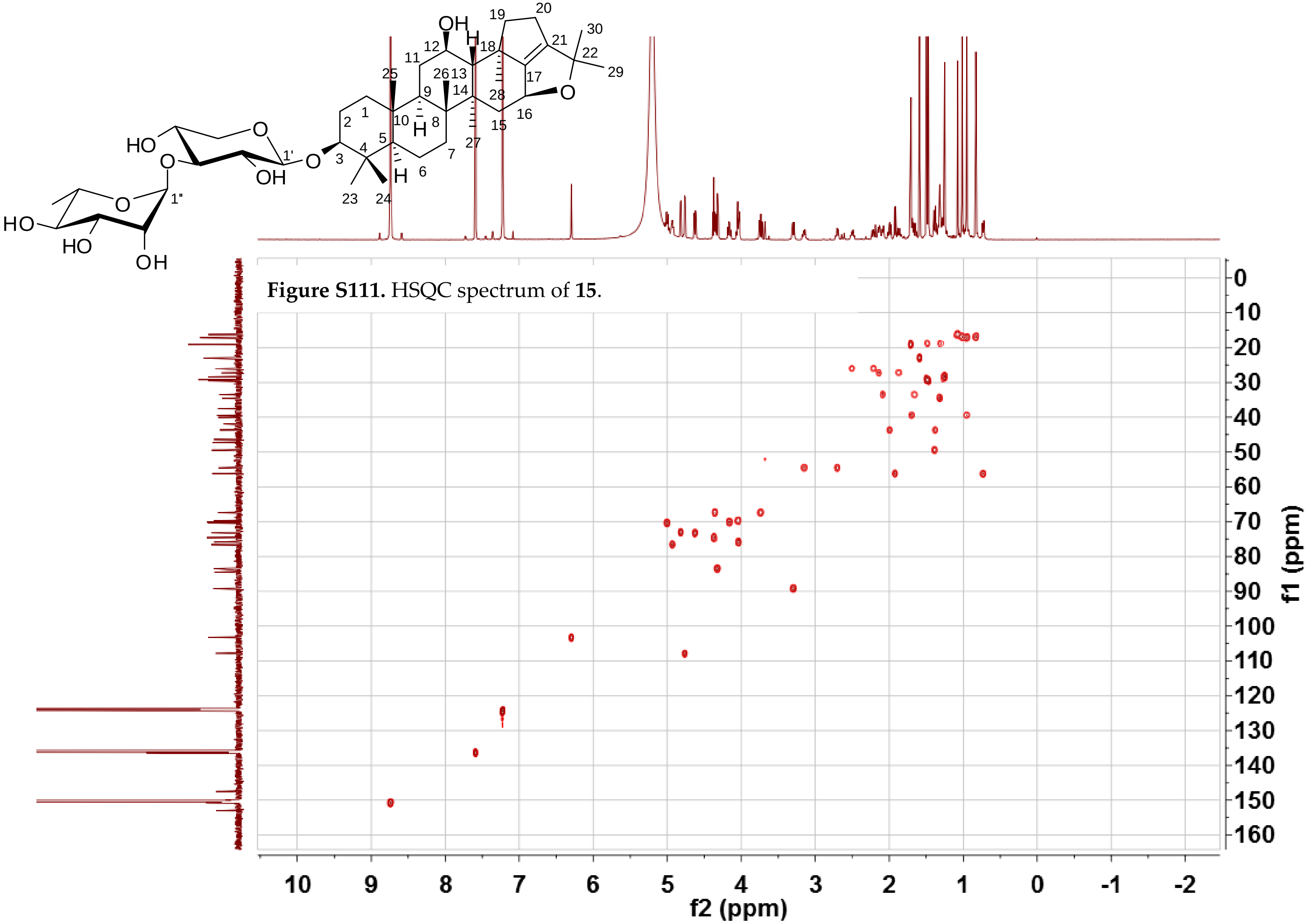

Figure S111. HSQC spectrum of **15**.

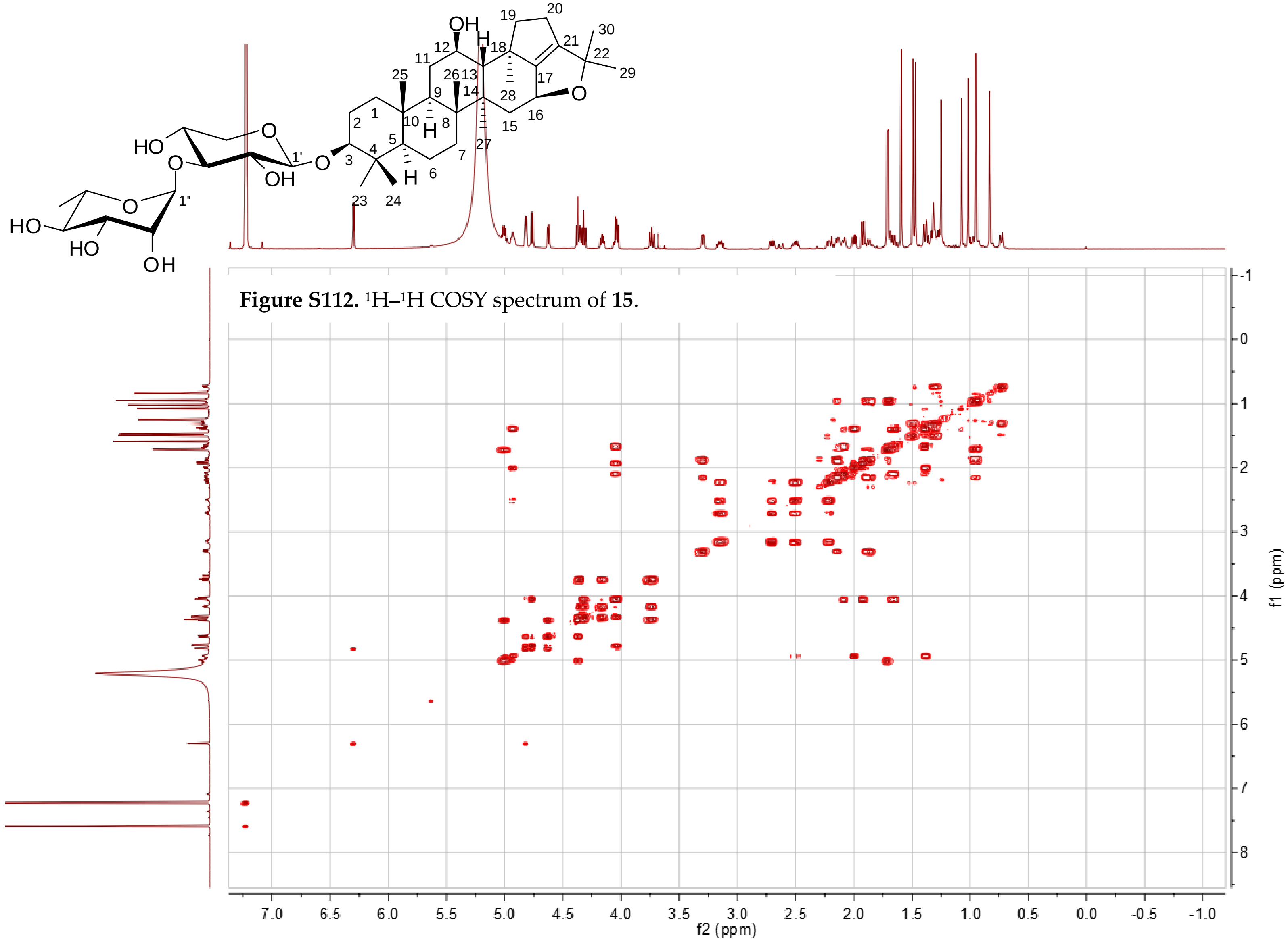

Figure S112. ^{1}H–^{1}H COSY spectrum of **15**.

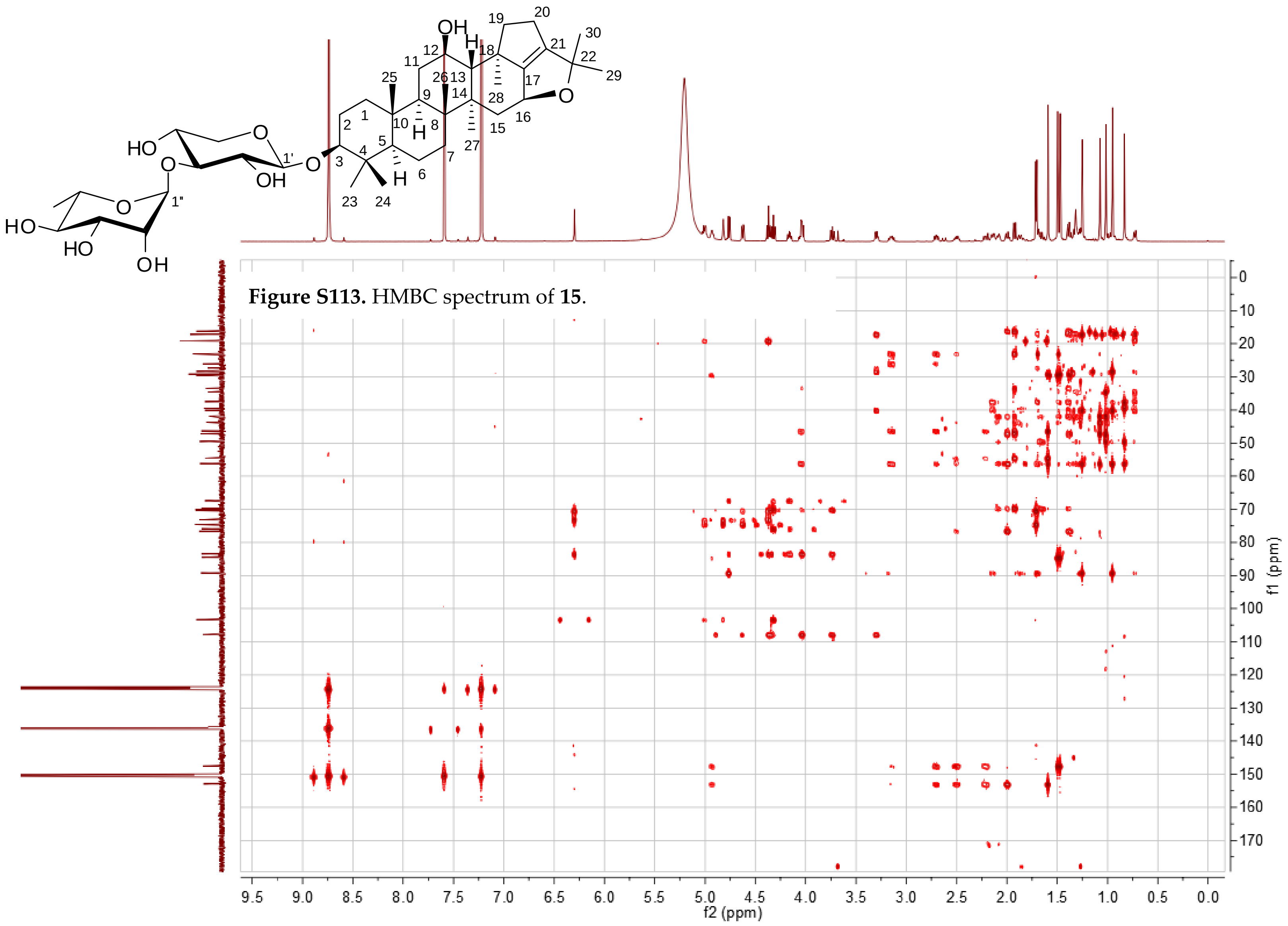

Figure S113. HMBC spectrum of **15**.

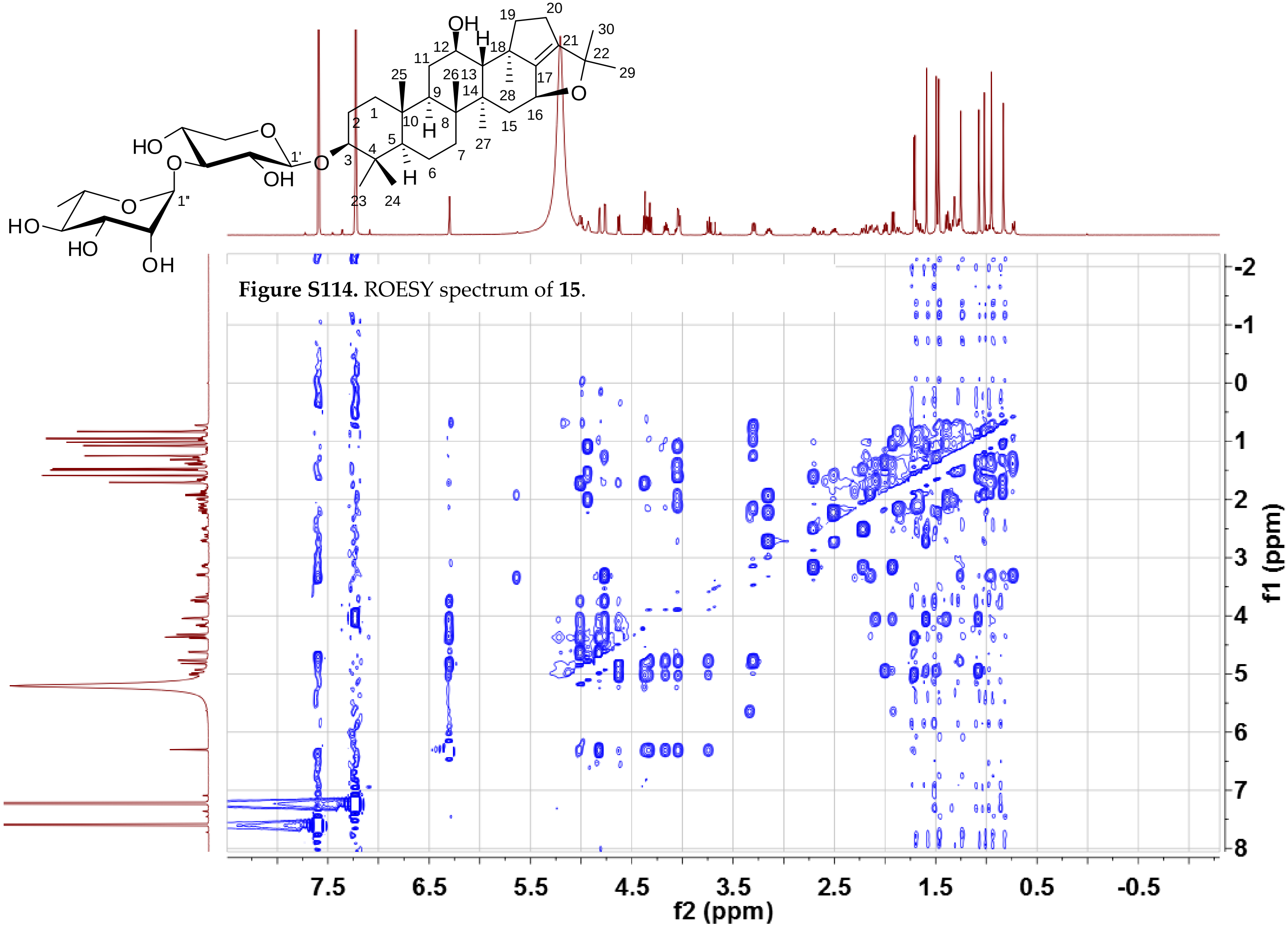

Figure S114. ROESY spectrum of **15**.

**Data Filename**	180829ESIA7.d	**Sample Name**	pdt56
**Sample Type**	Sample	**Position**	
**Instrument Name**	Agilent G6230 TOF MS	**User Name**	KIB
**Acq Method**	ESI.m	**Acquired Time**	8/29/2018 1:53:43 PM
**IRM Calibration Status**	Success	**DA Method**	ESI.m
**Comment**			

Sample Group **Info.**
Acquisition SW Version 6200 series TOF/6500 series Q-TOF B.05.01 (B5125.2)

User Spectra

Fragmentor Voltage	Collision Energy	Ionization Mode
200	0	ESI

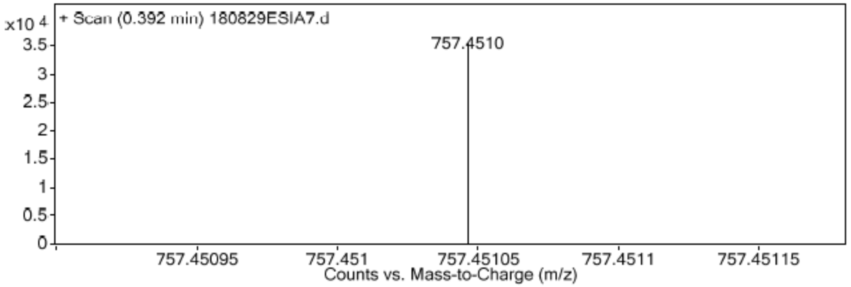

Peak List

m/z	z	Abund	Formula	Ion
107.0364	1	14841.42		
112.188	1	21820.84		
145.2501		9138.86		
152.9476		8863.32		
754.4438	2	10091.41		
754.9467	2	9362.35		
757.451	1	35198.19	C41 H66 Na O11	M+
758.455	1	17220.5	C41 H66 Na O11	M+
773.4255	1	9346.08		
887.409	1	13063.82		

Formula Calculator Element Limits

Element	Min	Max
C	0	200
H	0	400
O	7	15
Na	1	1

Formula Calculator Results

Formula	CalculatedMass	Mz	Diff.(mDa)	Diff. (ppm)	DBE
C41 H66 Na O11	757.4503	757.4510	-0.7	1.0	8.5

--- End Of Report ---

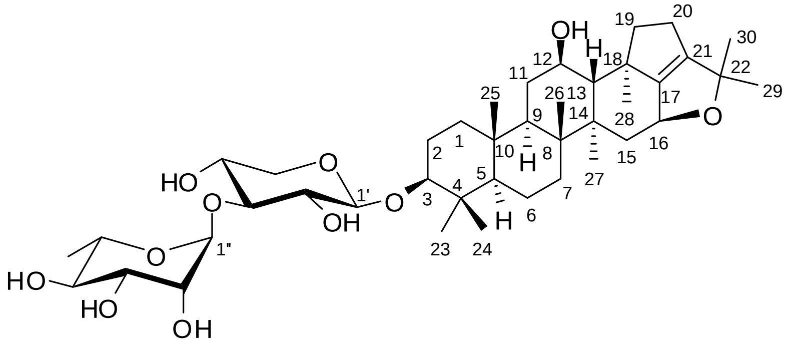

Figure S115. HRESIMS spectrum of **15**.

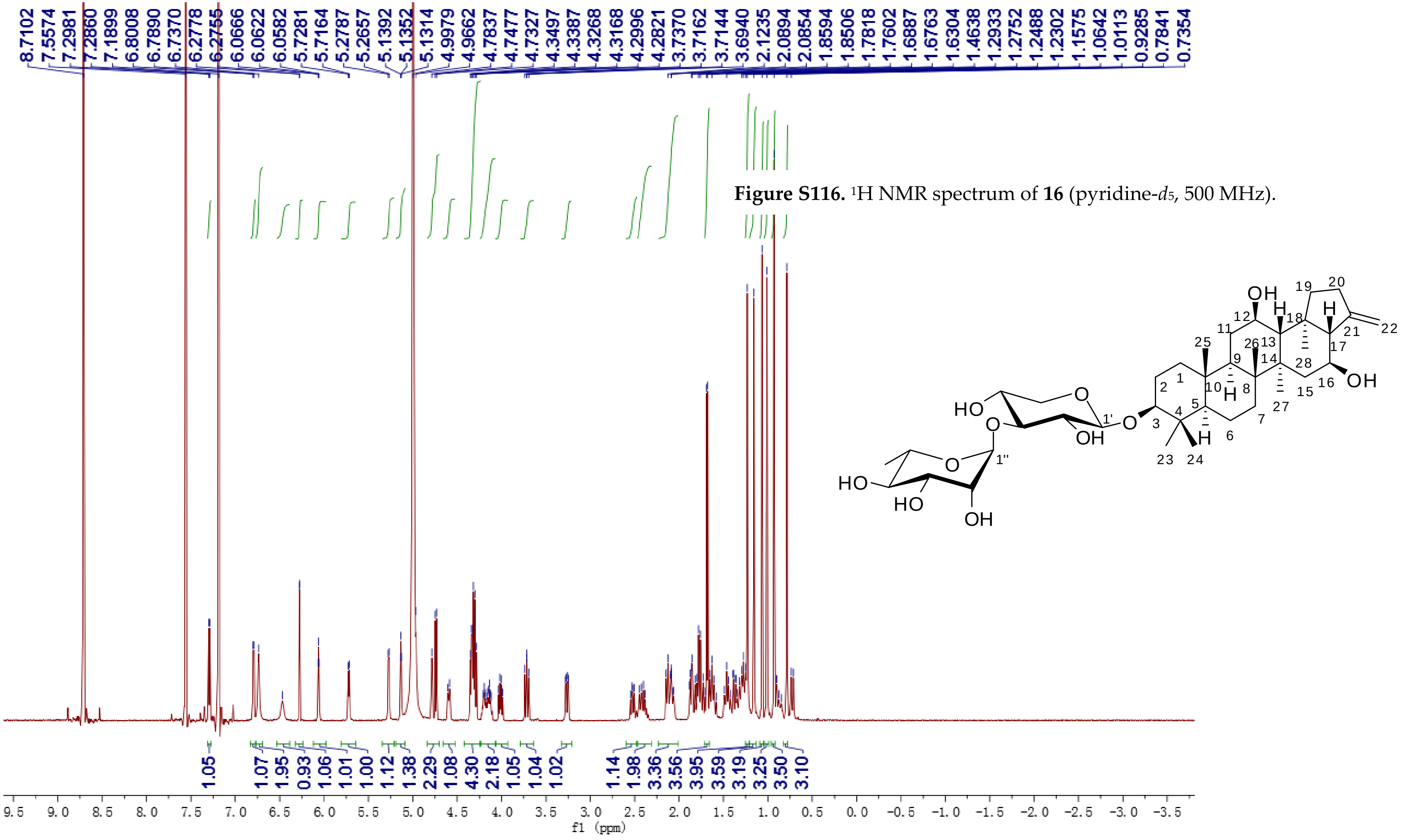

Figure S116. ^{1}H NMR spectrum of **16** (pyridine-*d*5, 500 MHz).

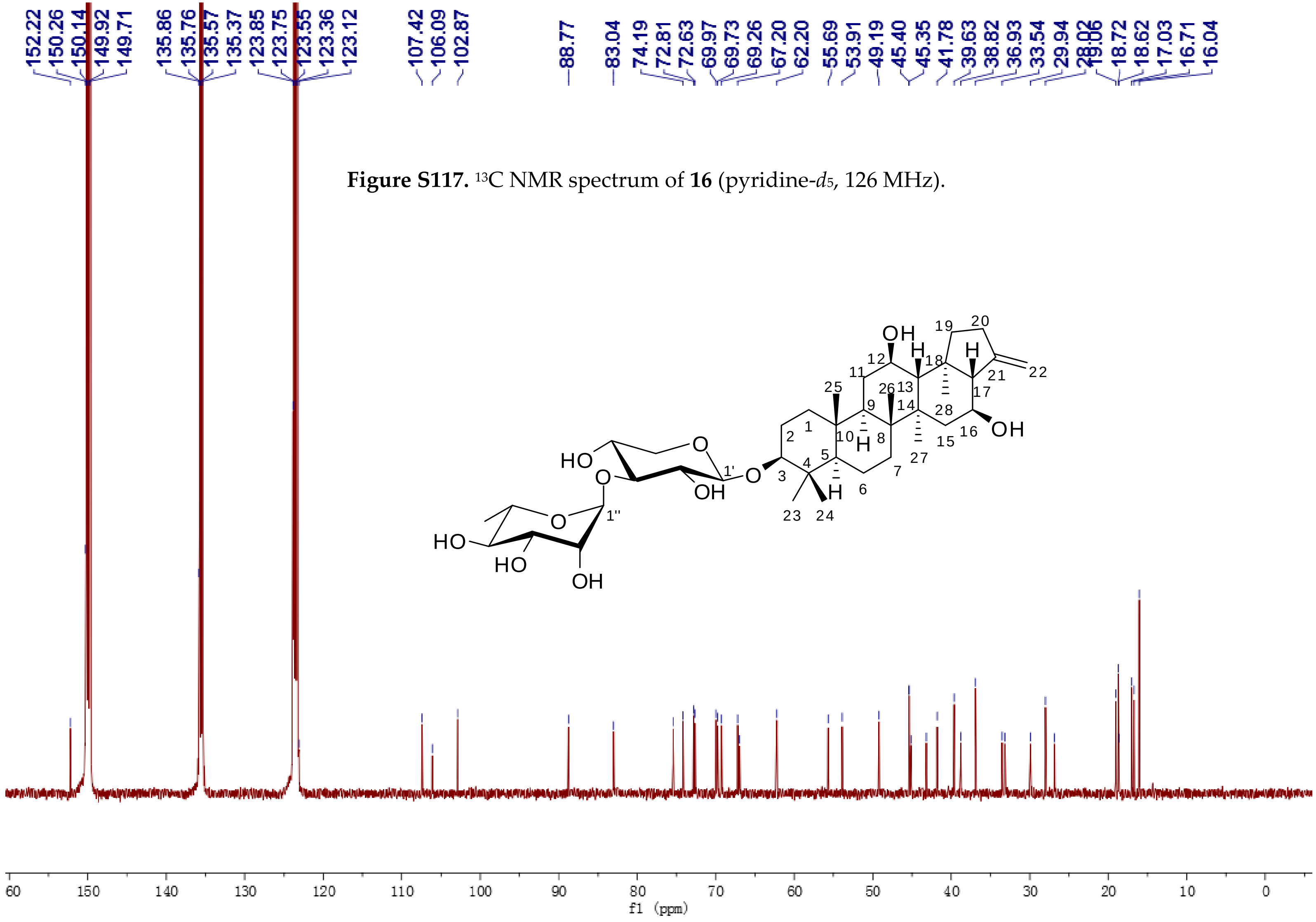

Figure S117. ^{13}C NMR spectrum of **16** (pyridine-d_5, 126 MHz).

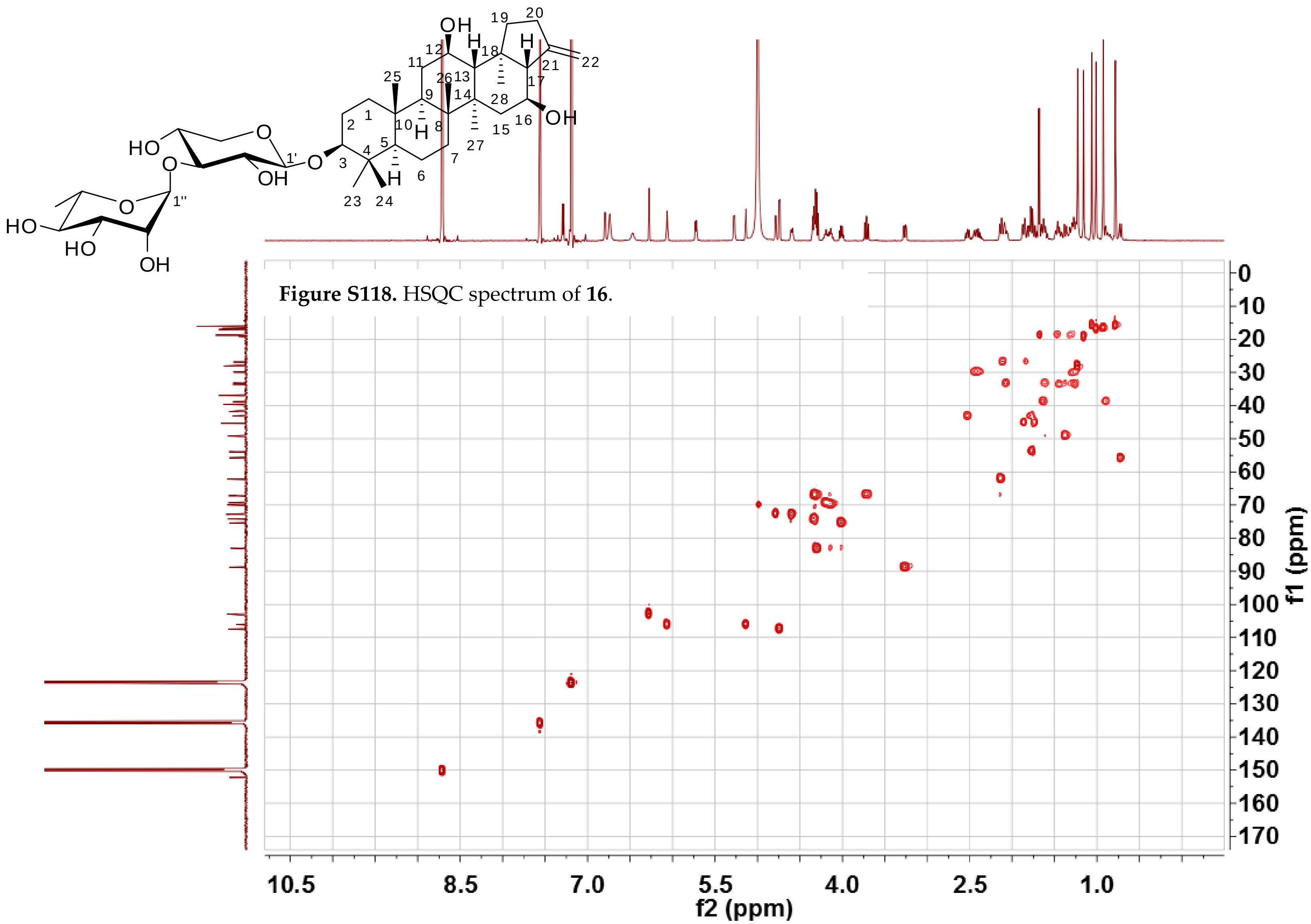

Figure S118. HSQC spectrum of **16**.

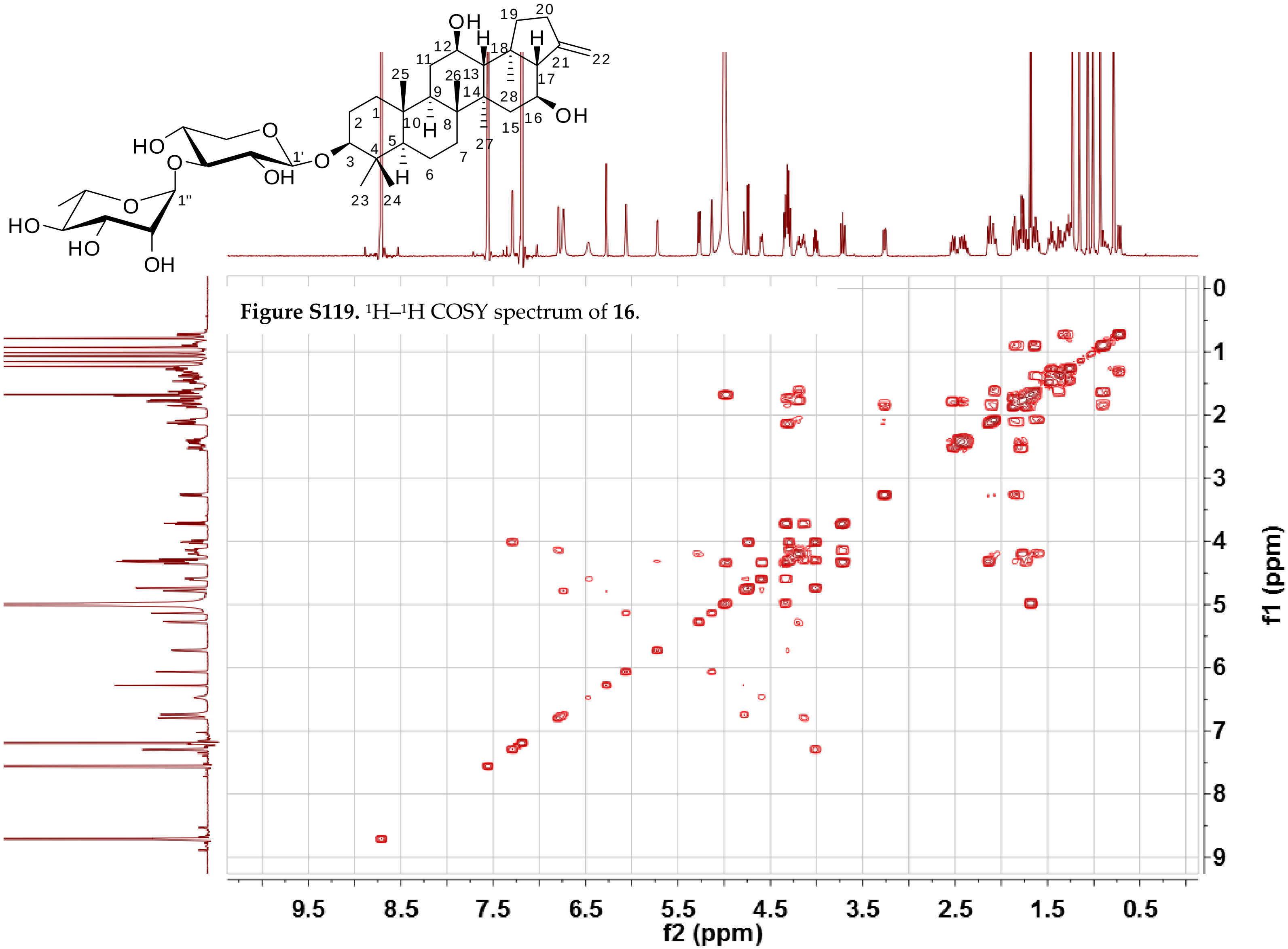

Figure S119. ^{1}H–^{1}H COSY spectrum of **16**.

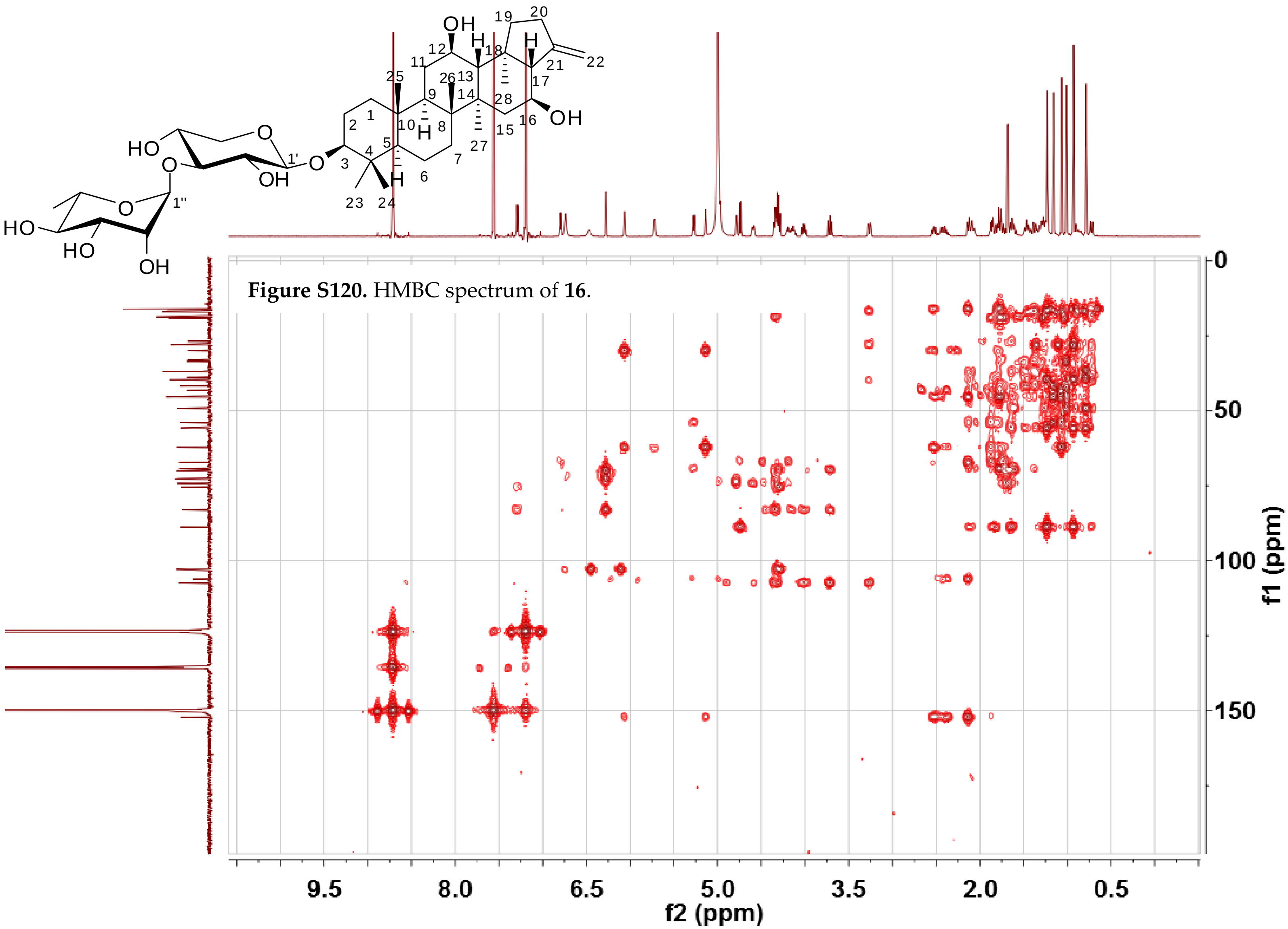

Figure S120. HMBC spectrum of **16**.

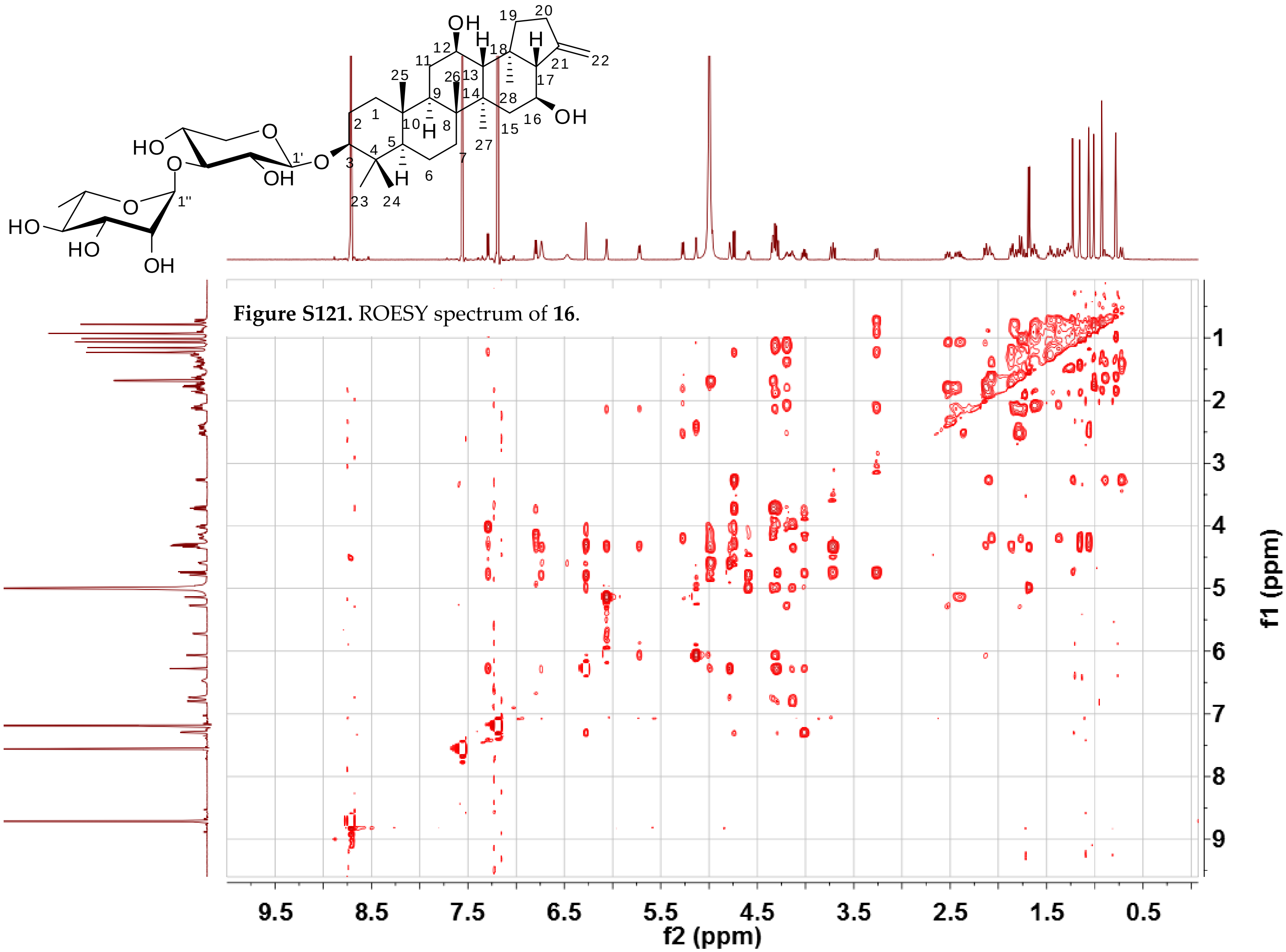

Figure S121. ROESY spectrum of **16**.

**Data Filename**	171020ESIA4.d	**Sample Name**	pdt4
**Sample Type**	Sample	**Position**	
**Instrument Name**	Agilent G6230 TOF MS	**User Name**	KIB
**Acq Method**	ESI.m	**Acquired Time**	10/23/2017 10:43:40 AM
**IRM Calibration Status**	Success	**DA Method**	ESI.m
**Comment**			

**Sample Group**		**Info.**
**Acquisition SW Version**	6200 series TOF/6500 series Q-TOF B.05.01 (B5125.2)	

User Spectra

Fragmentor Voltage	Collision Energy	Ionization Mode
230	0	ESI

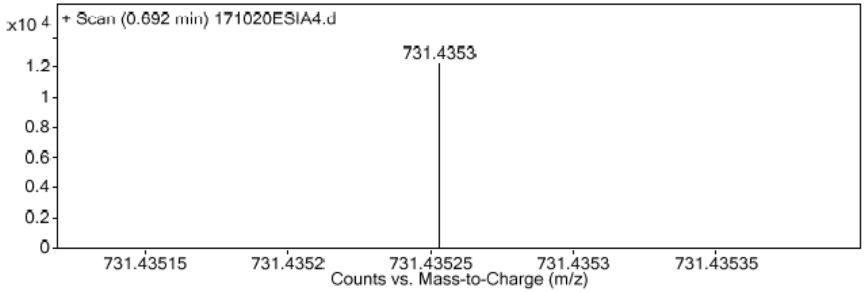

Figure S122. HRESIMS spectrum of **16**.

Peak List

m/z	z	Abund	Formula	Ion
142.1588		6618.62		
230.2478		3655.01		
242.284	1	24682.75		
243.2871	1	3987.93		
340.2823	1	4079.48		
384.309	1	3931.27		
437.2179	1	3262.22		
731.4353	1	12223.29	C39 H64 Na O11	M+
732.4388	1	4466.36	C39 H64 Na O11	M+
733.4218	1	3921.16	C39 H64 Na O11	M+

Formula Calculator Element Limits

Element	Min	Max
C	0	200
H	0	400
O	7	13
Na	1	1

Formula Calculator Results

Formula	CalculatedMass	Mz	Diff.(mDa)	Diff. (ppm)	DBE
C39 H64 Na O11	731.4346	731.4353	-0.7	0.9	7.5

--- End Of Report ---

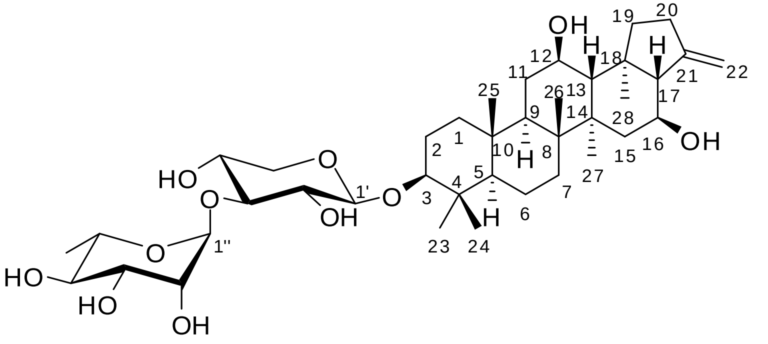

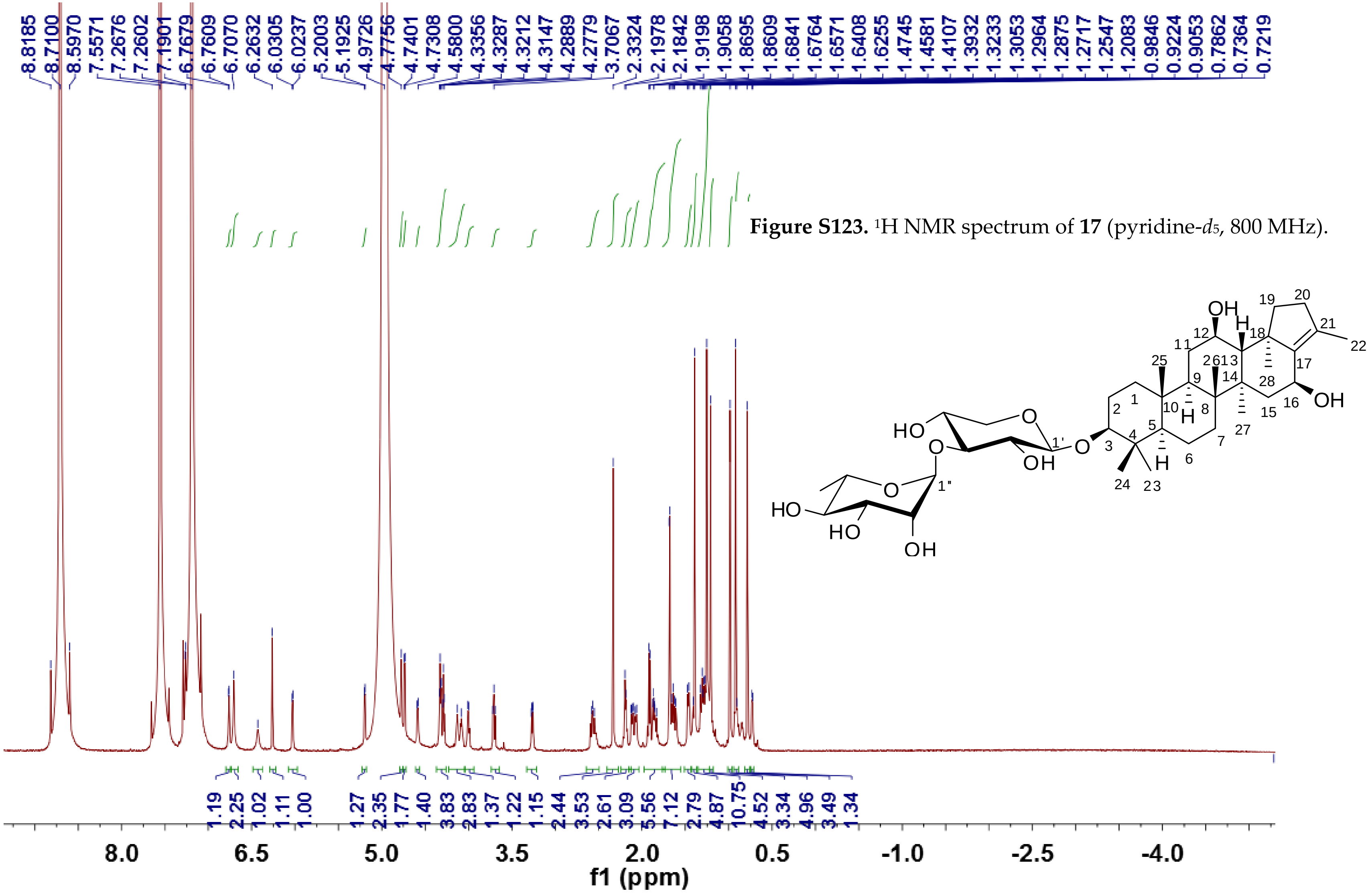

Figure S123. ^{1}H NMR spectrum of **17** (pyridine-d_5, 800 MHz).

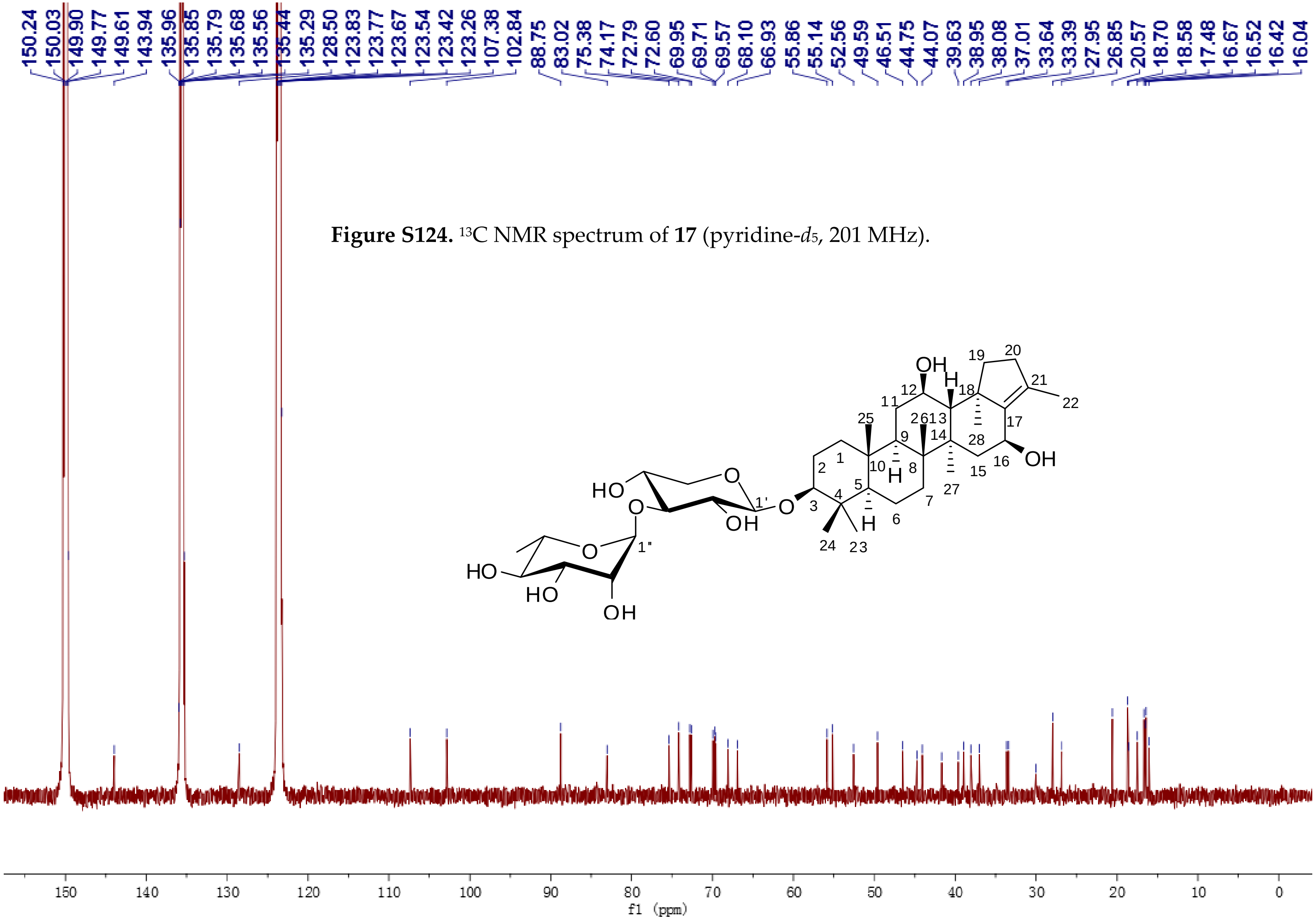

Figure S124. ^{13}C NMR spectrum of **17** (pyridine-d_5, 201 MHz).

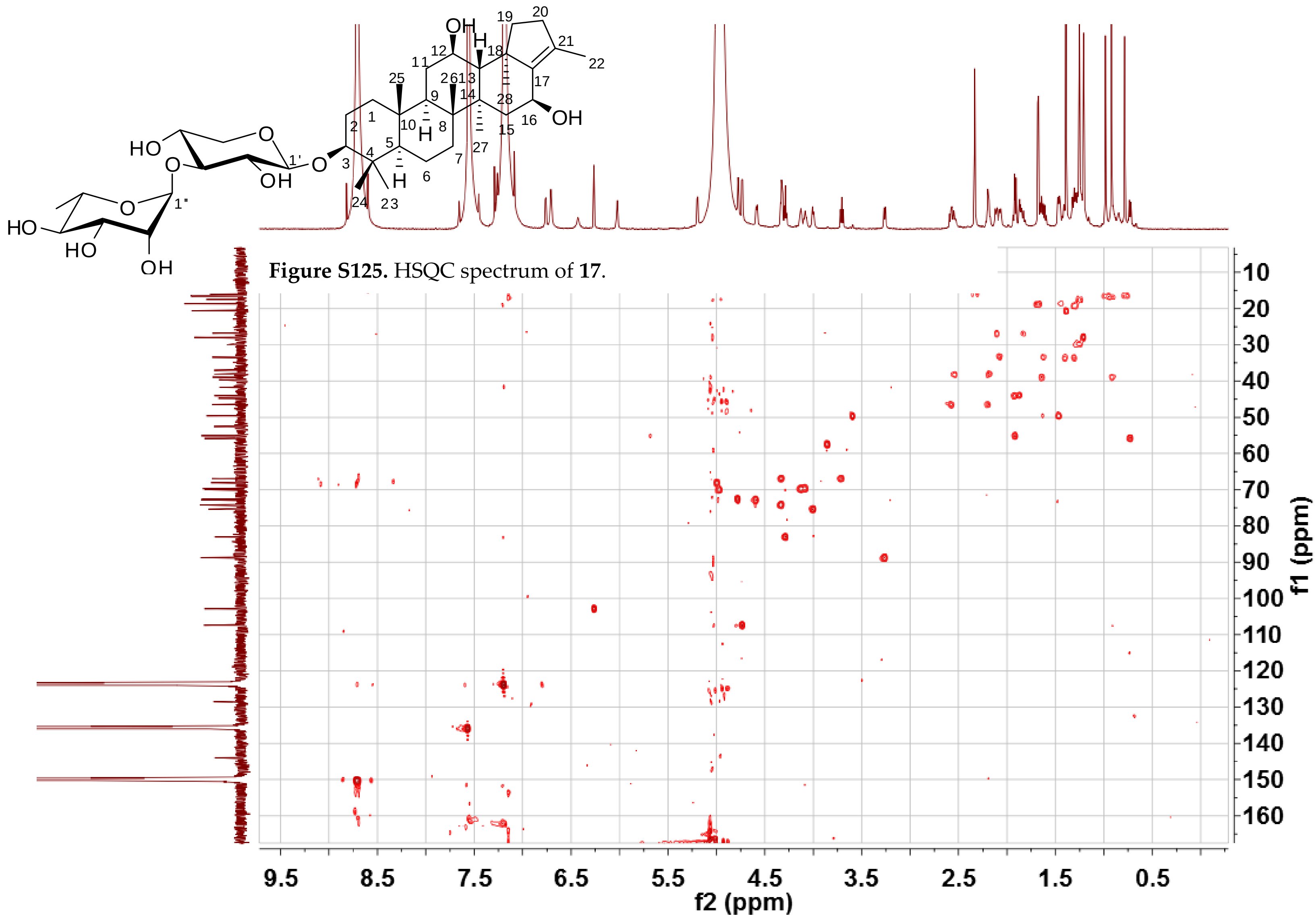

Figure S125. HSQC spectrum of **17**.

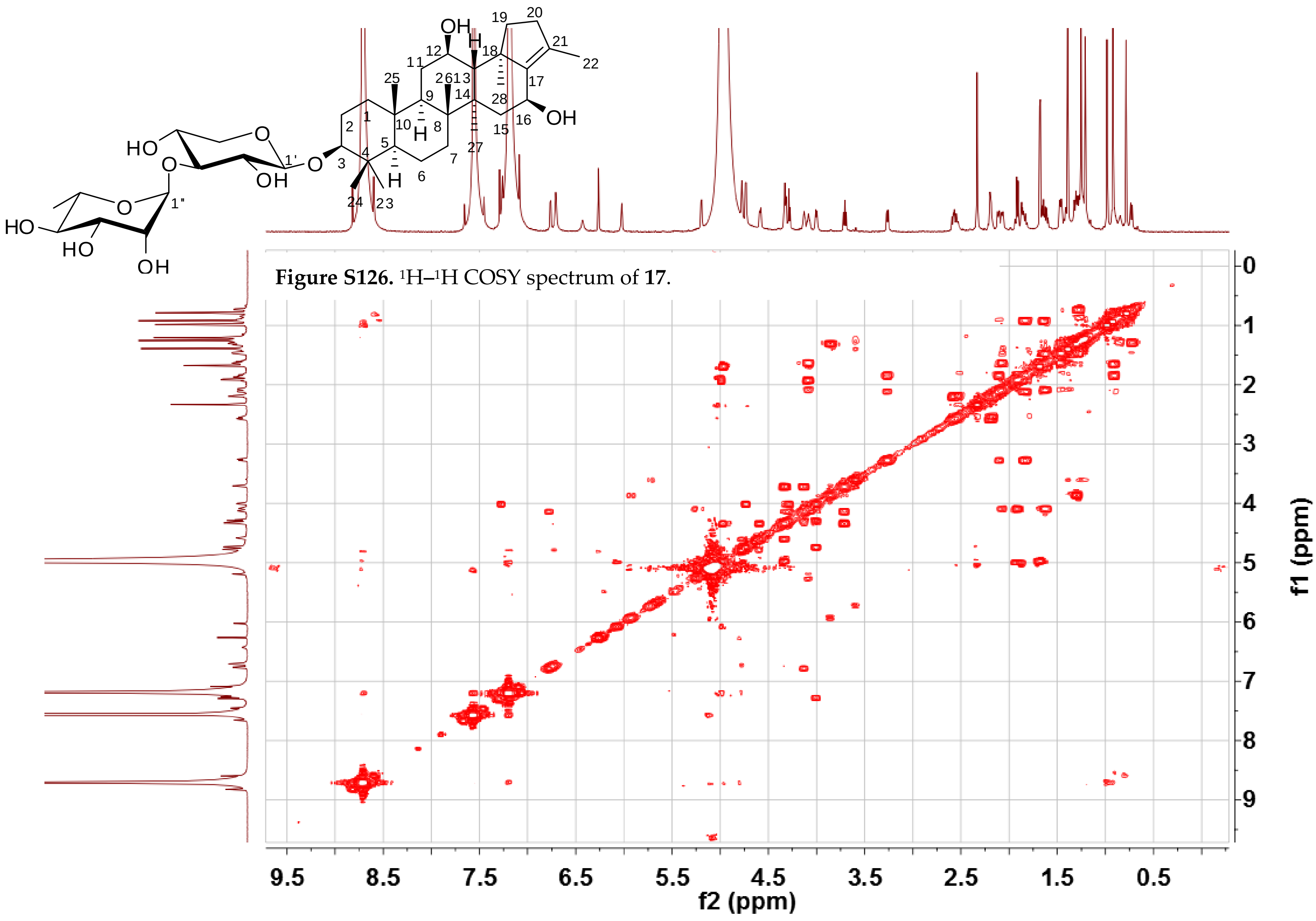

Figure S126. ^{1}H–^{1}H COSY spectrum of **17**.

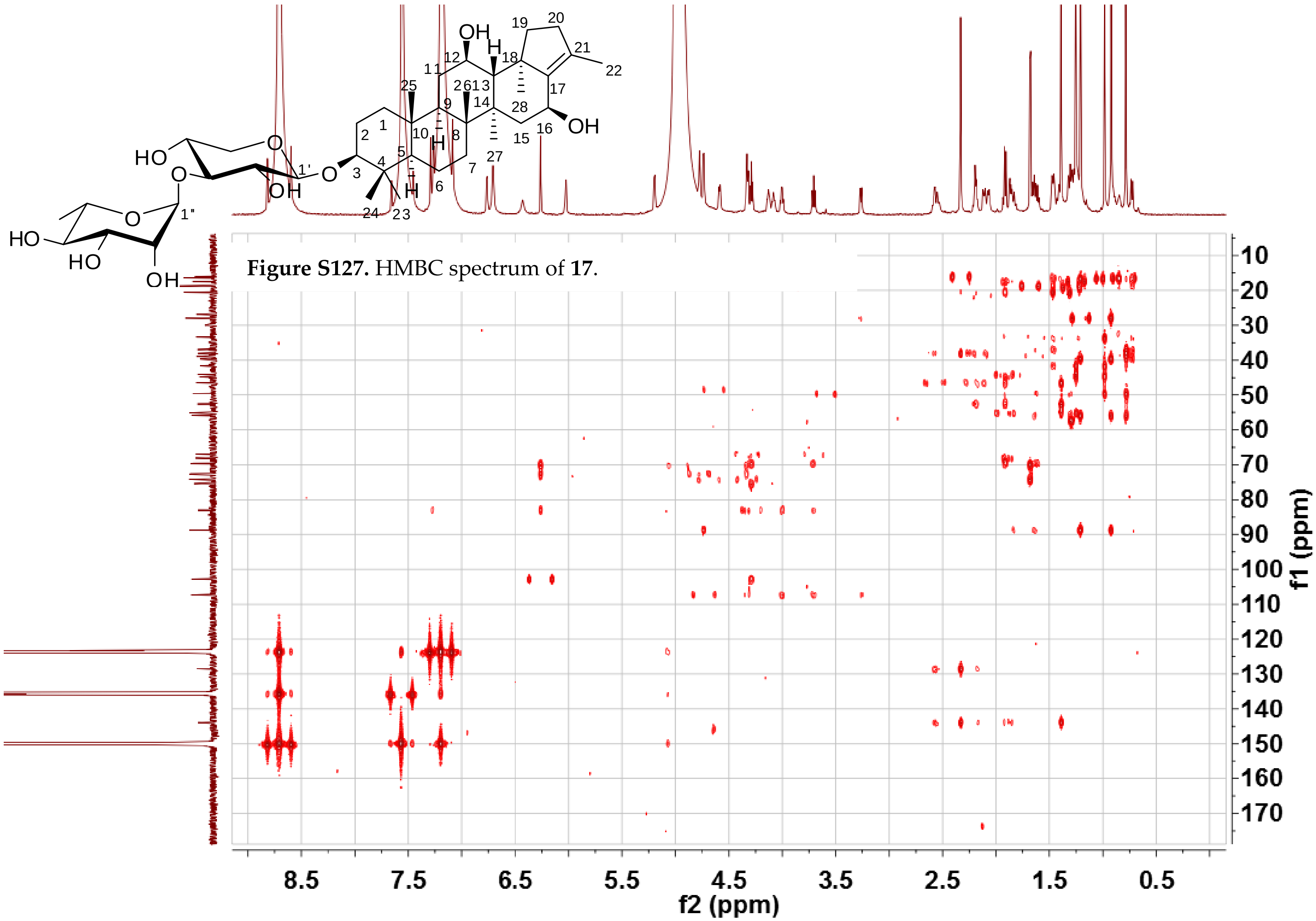

Figure S127. HMBC spectrum of **17**.

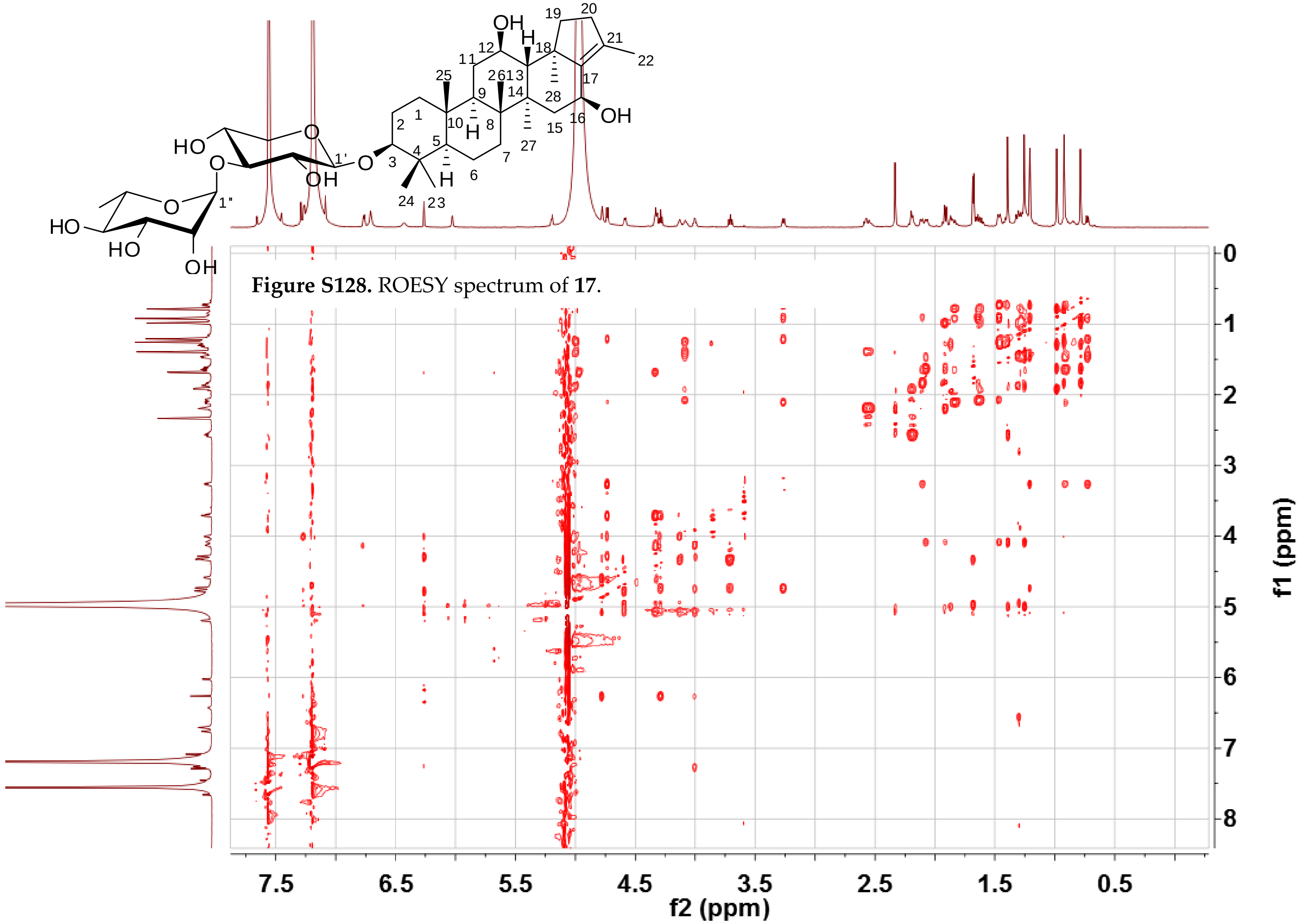

Figure S128. ROESY spectrum of **17**.

**Data Filename**	171023ESIA1.d	**Sample Name**	pdt18
**Sample Type**	Sample	**Position**	
**Instrument Name**	Agilent G6230 TOF MS	**User Name**	KIB
**Acq Method**	ESI.m	**Acquired Time**	10/23/2017 10:48:38 AM
**IRM Calibration Status**	Success	**DA Method**	ESI.m
**Comment**			

**Sample Group**		**Info.**
**Acquisition SW Version**	6200 series TOF/6500 series Q-TOF B.05.01 (B5125.2)	

User Spectra

Fragmentor Voltage	Collision Energy	Ionization Mode
260	0	ESI

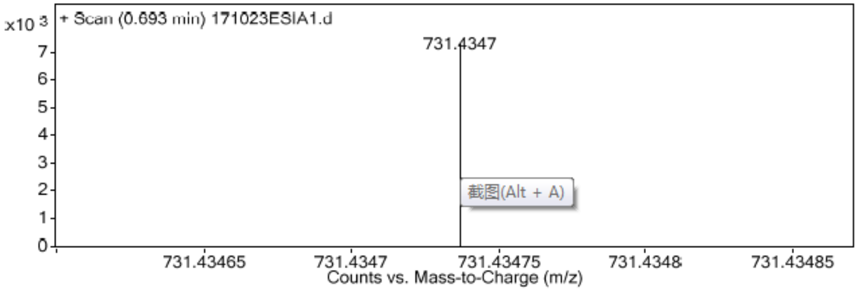

Figure S129. HRESIMS spectrum of **17**.

Peak List

m/z	z	Abund
112.1874	1	15398.25
178.2277	1	17871.92
182.256	1	21448.32
273.3796		10947.24
274.3865	1	32553.89
287.3954		11728.15
288.4024		48627.63
289.4091		195593.86
290.4155	1	464535.06
291.4184	1	66284.01

Formula Calculator Element Limits

Element	Min	Max
C	0	200
H	0	400
O	7	13
Na	1	1

Formula Calculator Results

Formula	CalculatedMass	Mz	Diff.(mDa)	Diff. (ppm)	DBE
C39 H64 Na O11	731.4346	731.4347	-0.1	0.1	7.5

--- End Of Report ---

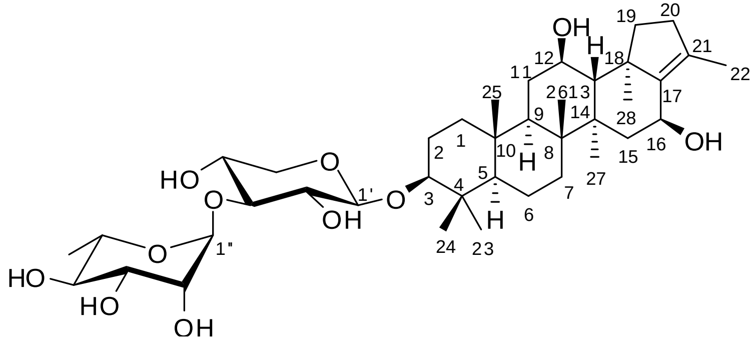

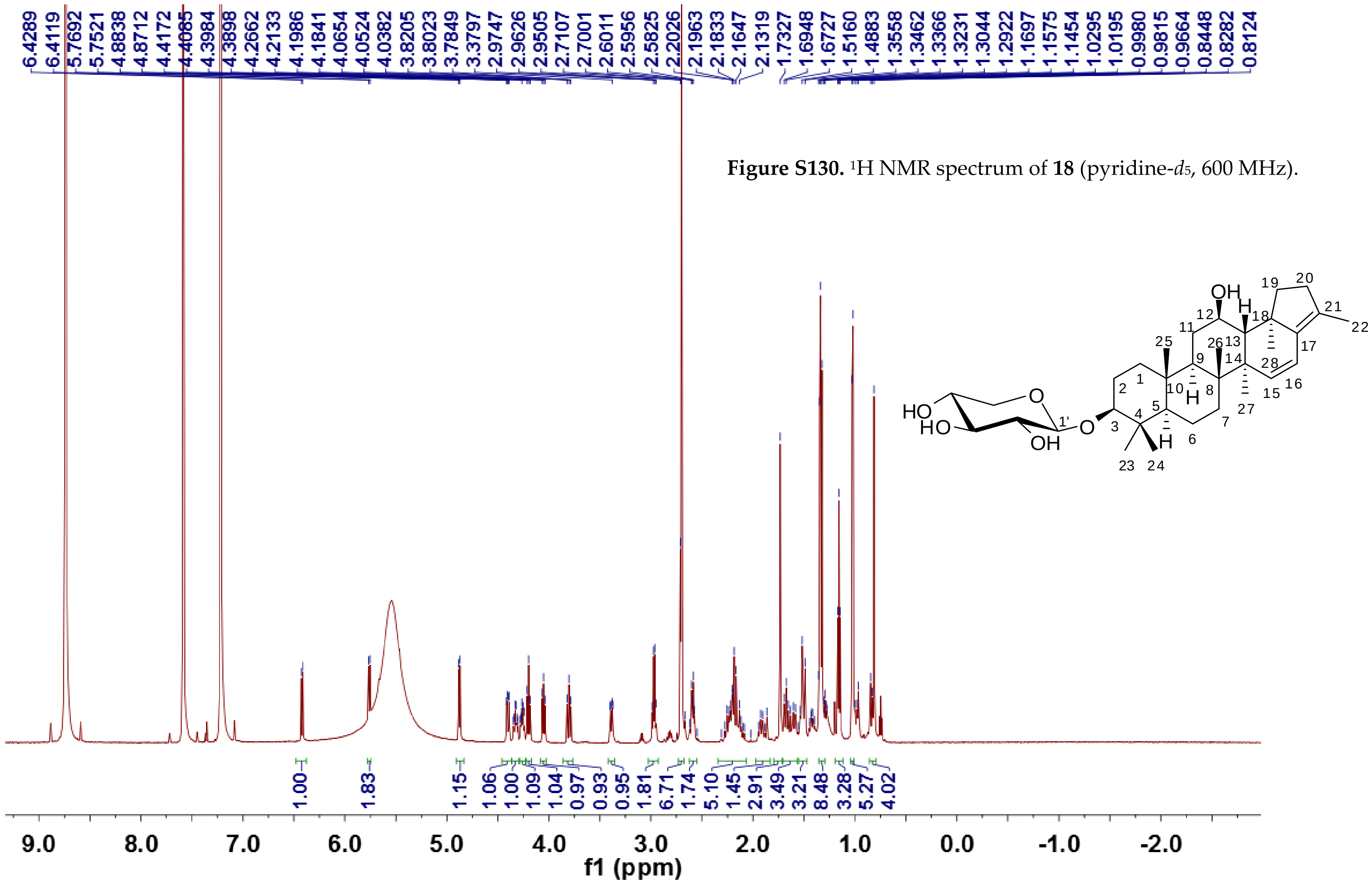

Figure S130. ^{1}H NMR spectrum of **18** (pyridine-d_5, 600 MHz).

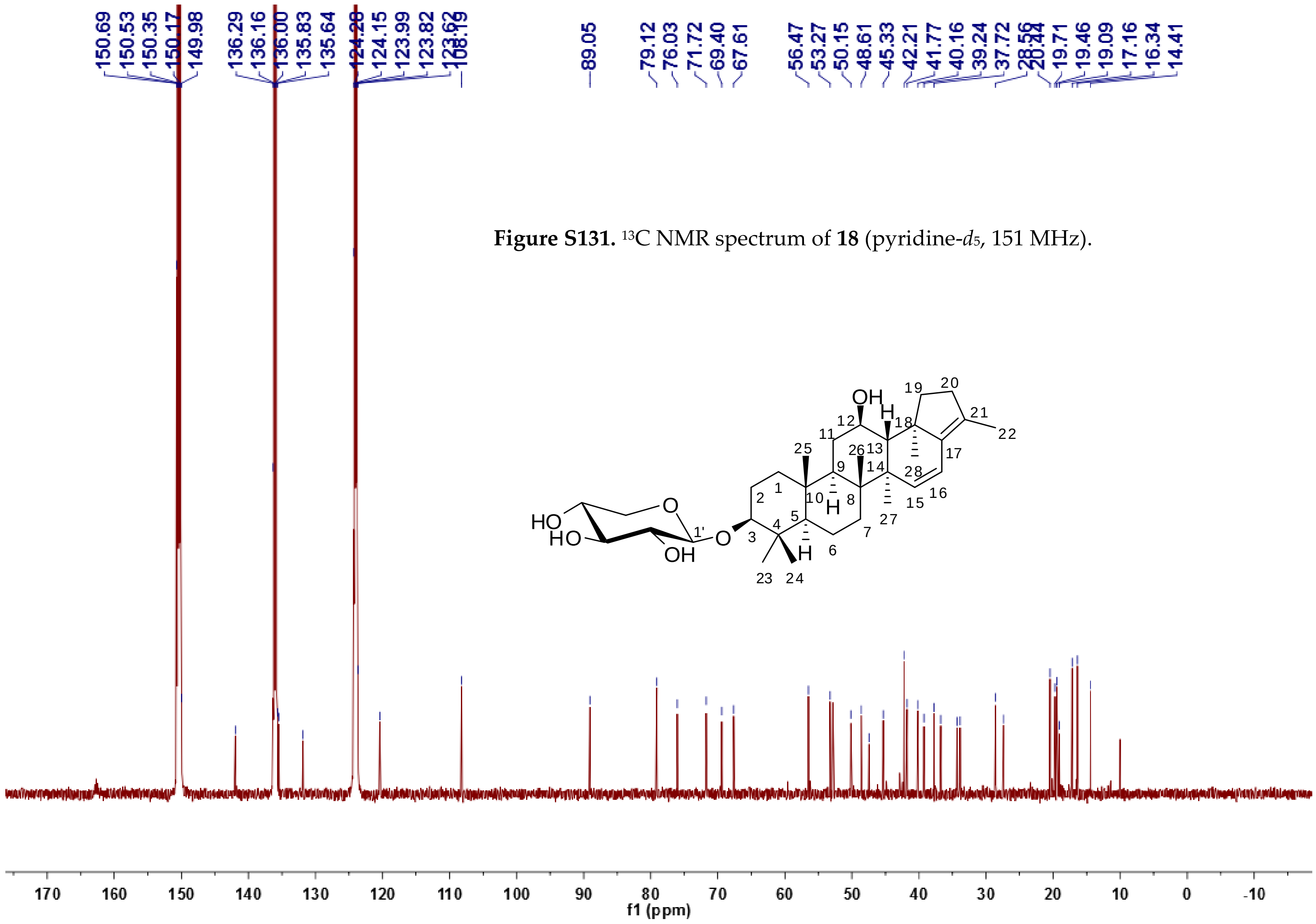

Figure S131. ^{13}C NMR spectrum of **18** (pyridine-*d*$_5$, 151 MHz).

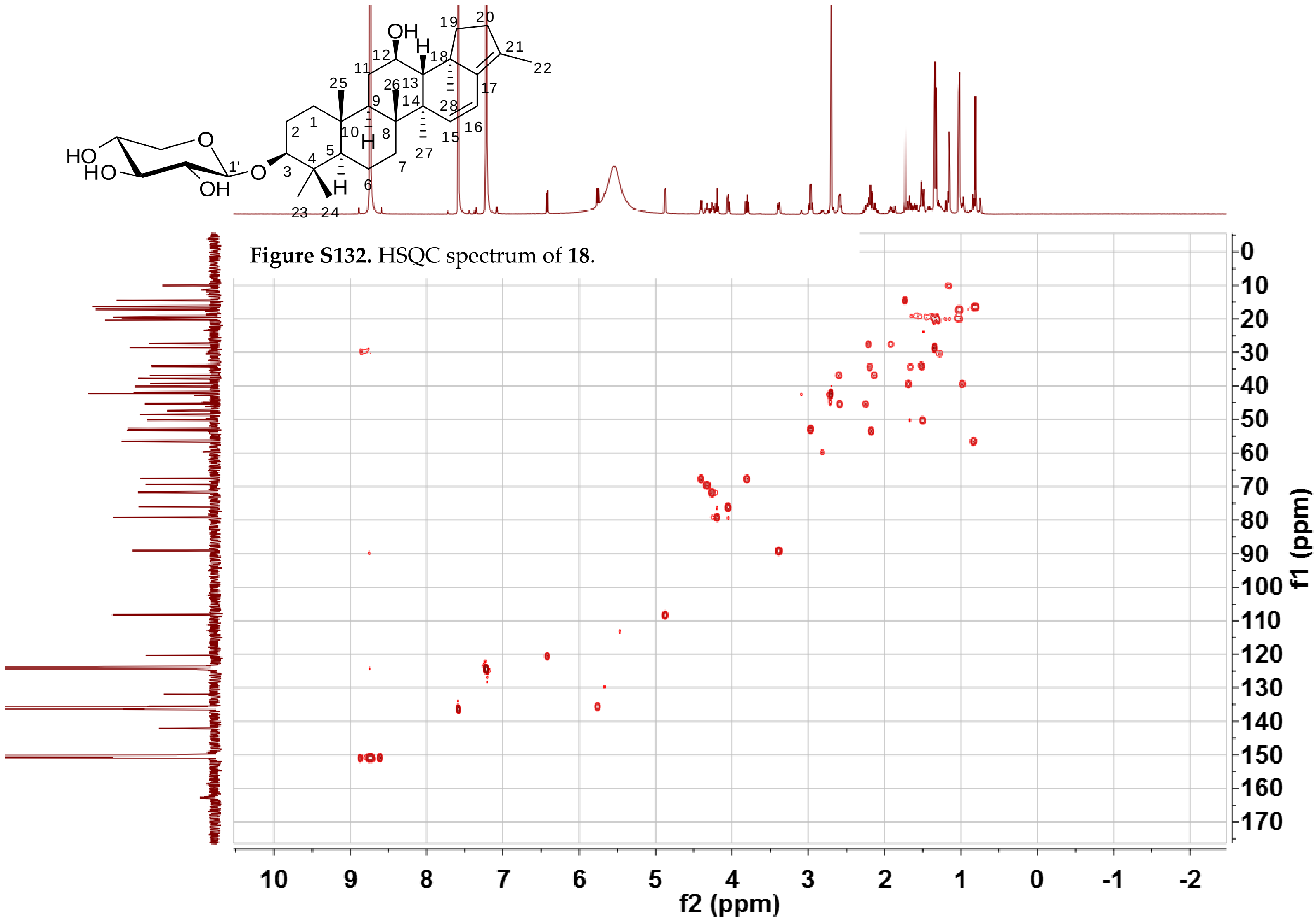

Figure S132. HSQC spectrum of **18**.

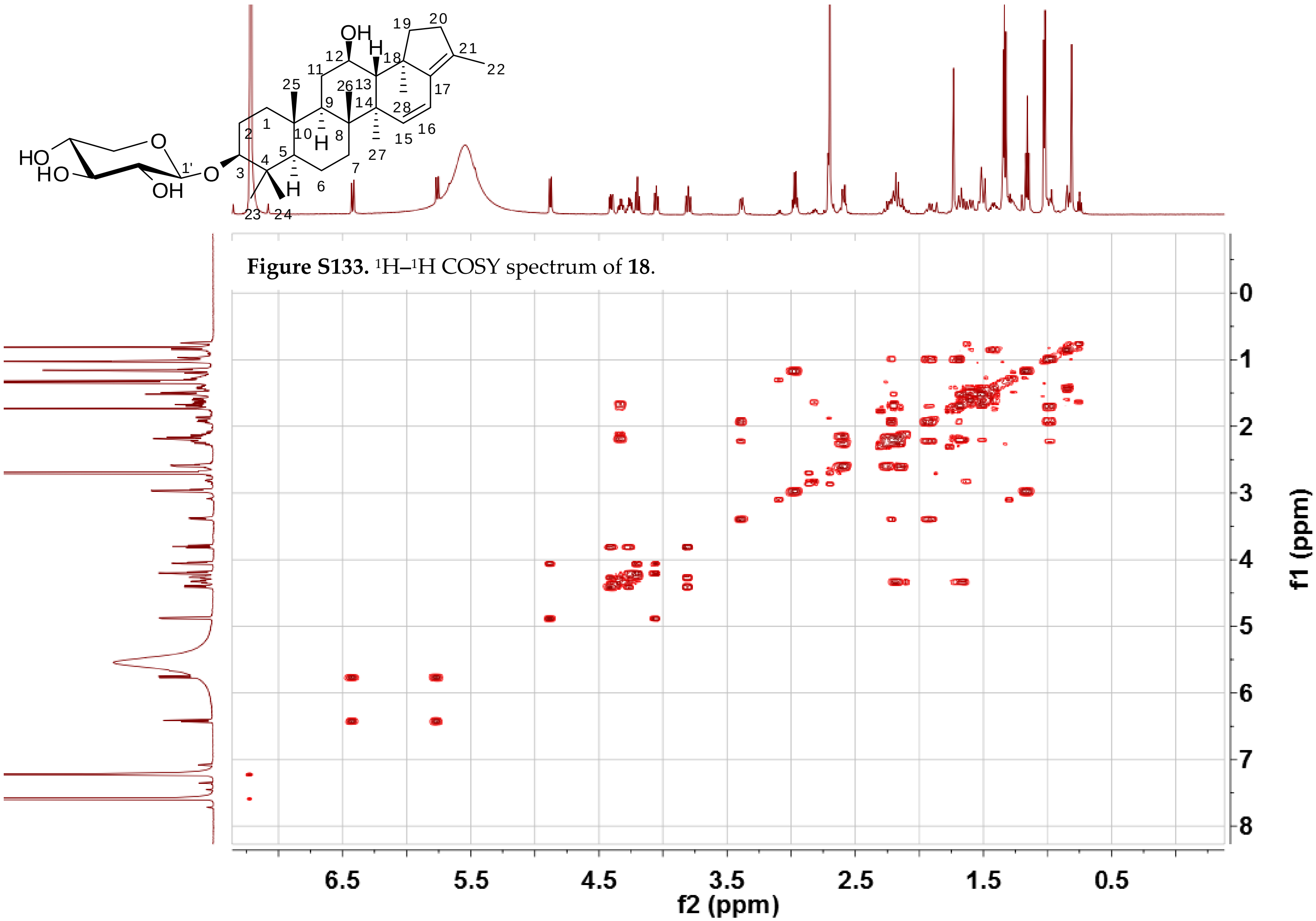

Figure S133. ^1H–^1H COSY spectrum of **18**.

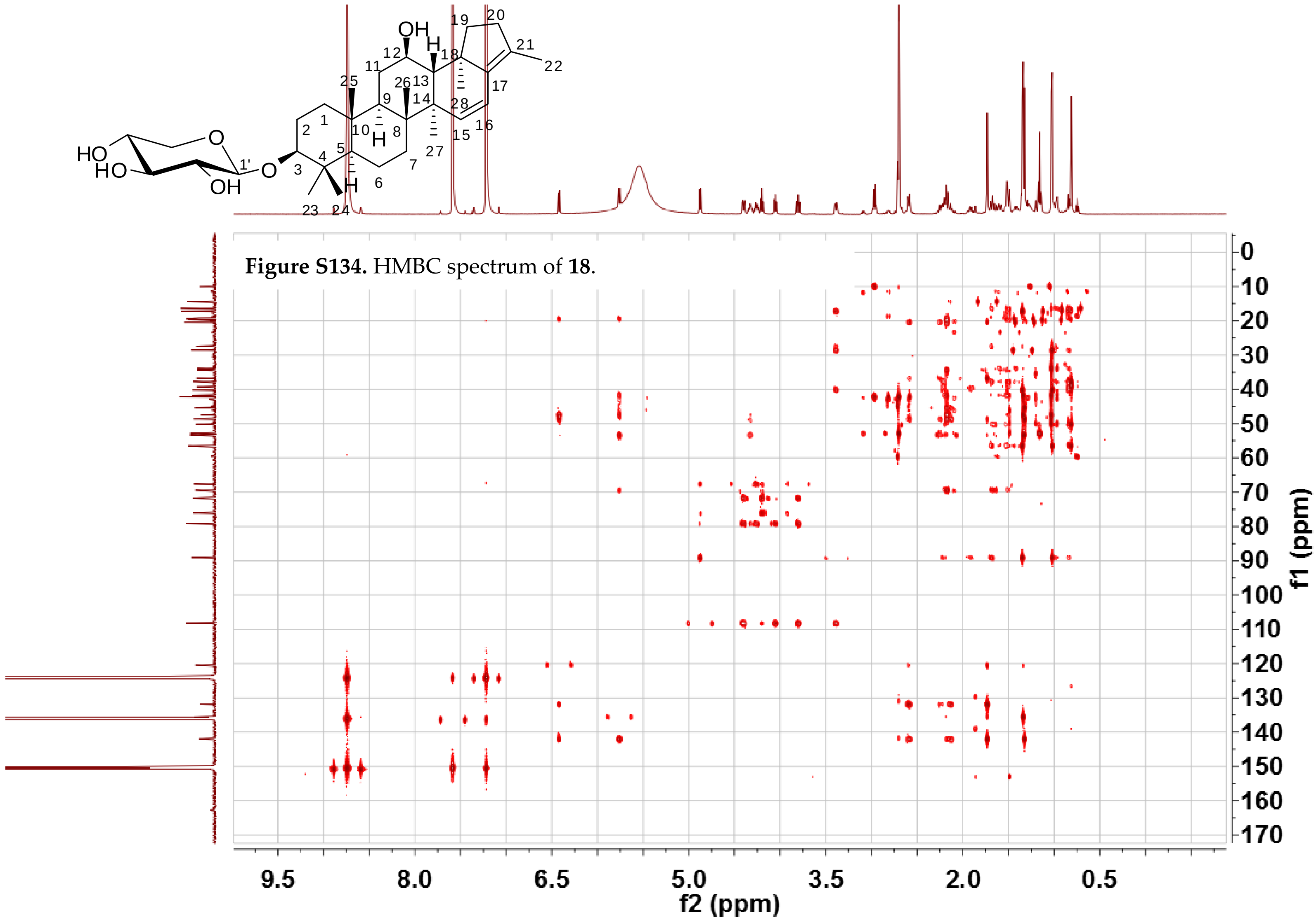

Figure S134. HMBC spectrum of **18**.

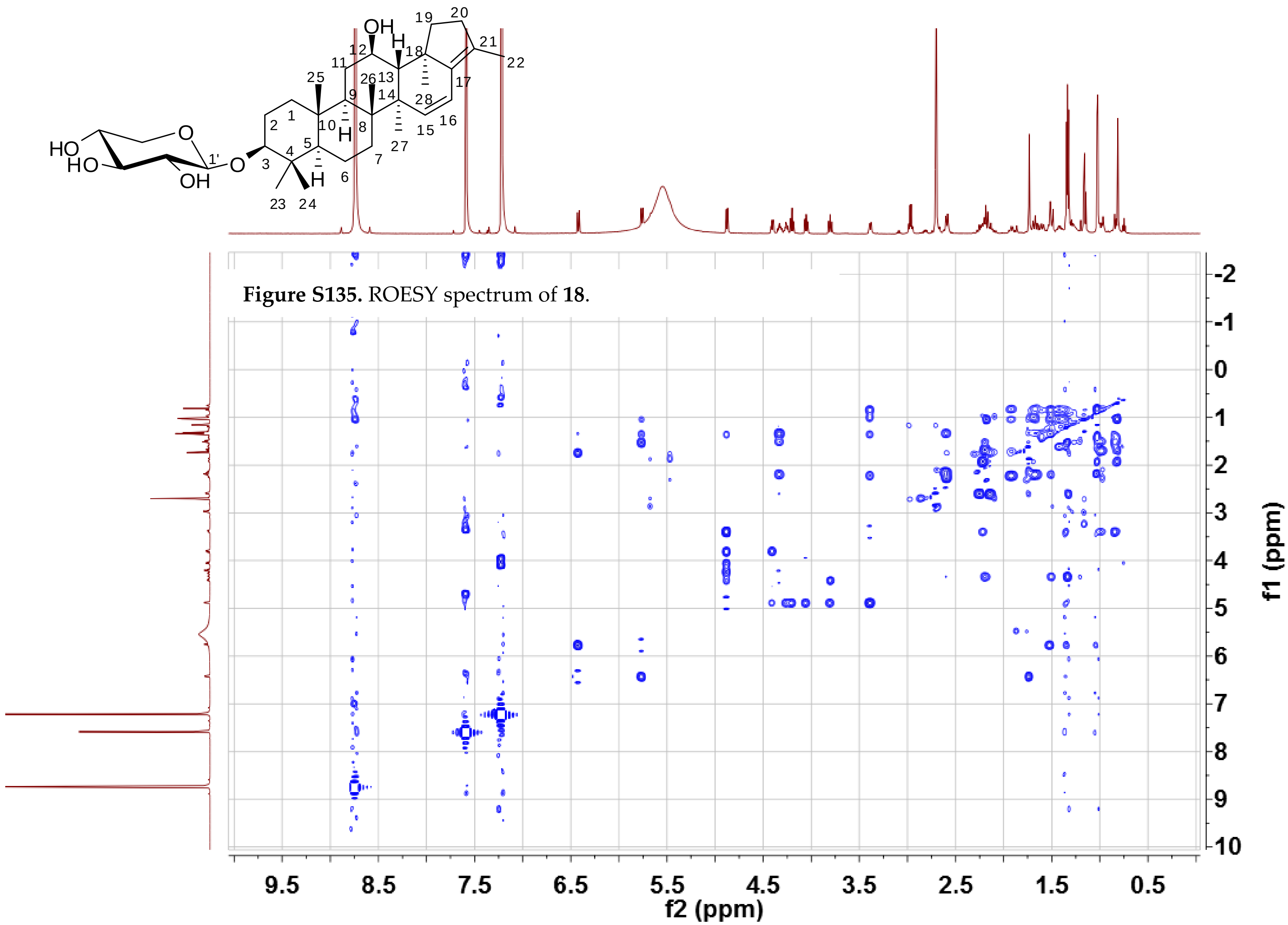

Figure S135. ROESY spectrum of **18**.

Figure S136. HRESIMS spectrum of **18**.

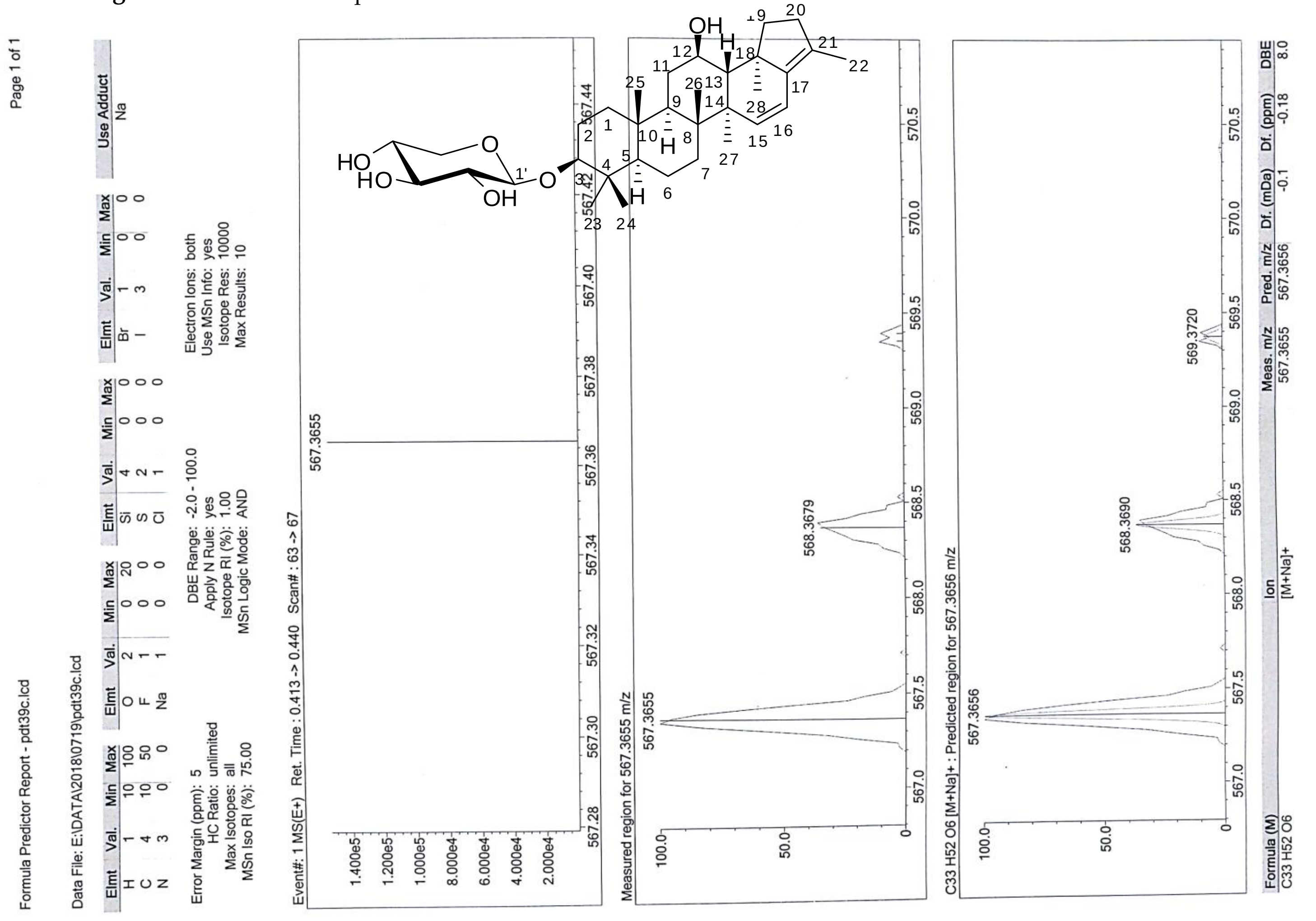

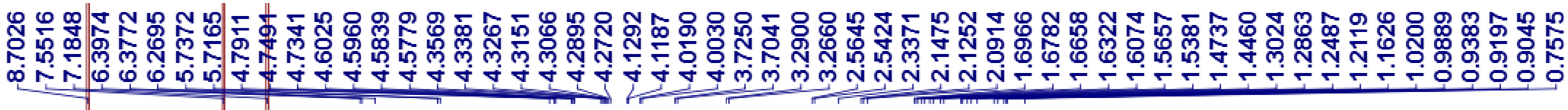

Figure S137. ^{1}H NMR spectrum of **19** (pyridine-d_5, 500 MHz).

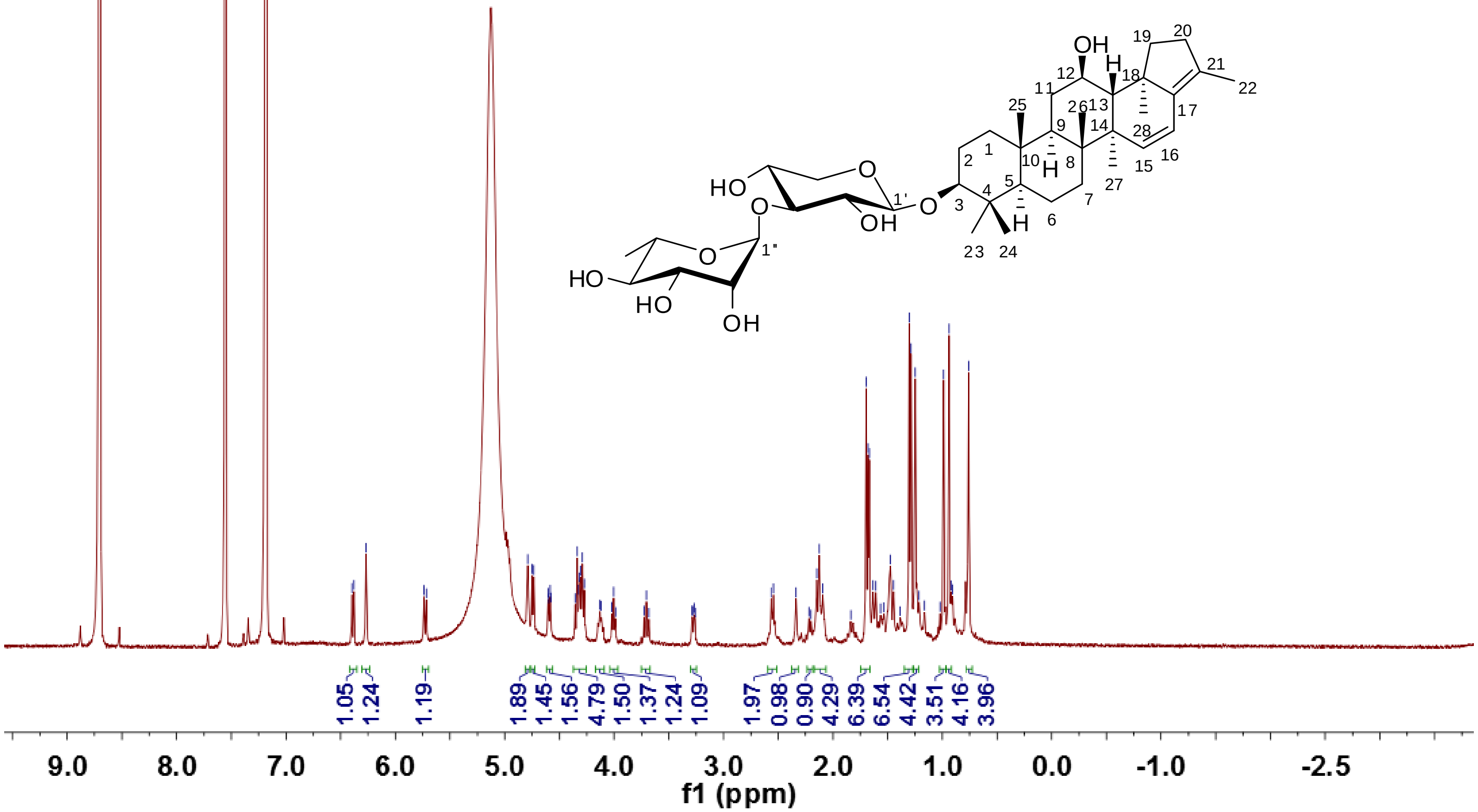

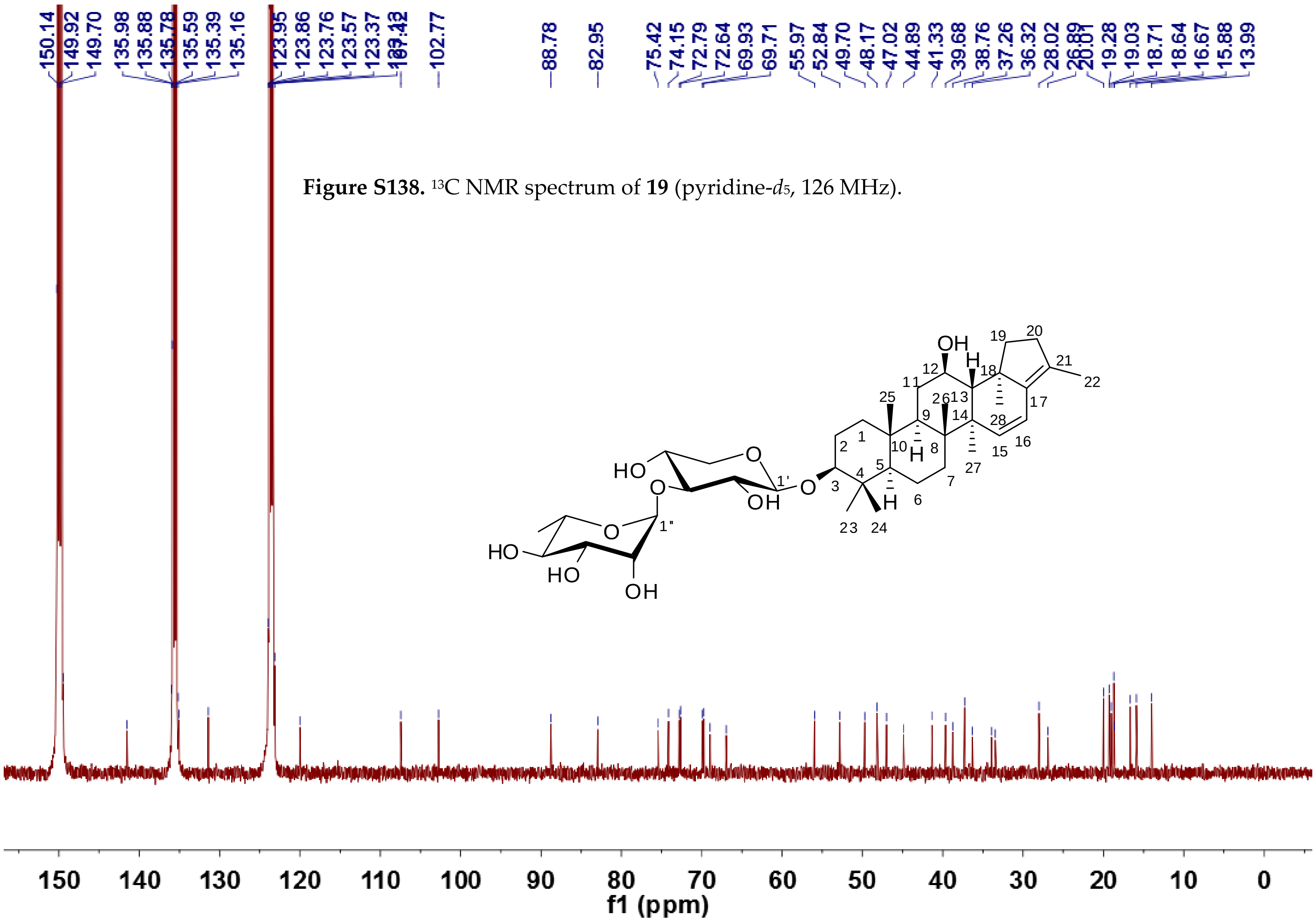

Figure S138. ^{13}C NMR spectrum of **19** (pyridine-d_5, 126 MHz).

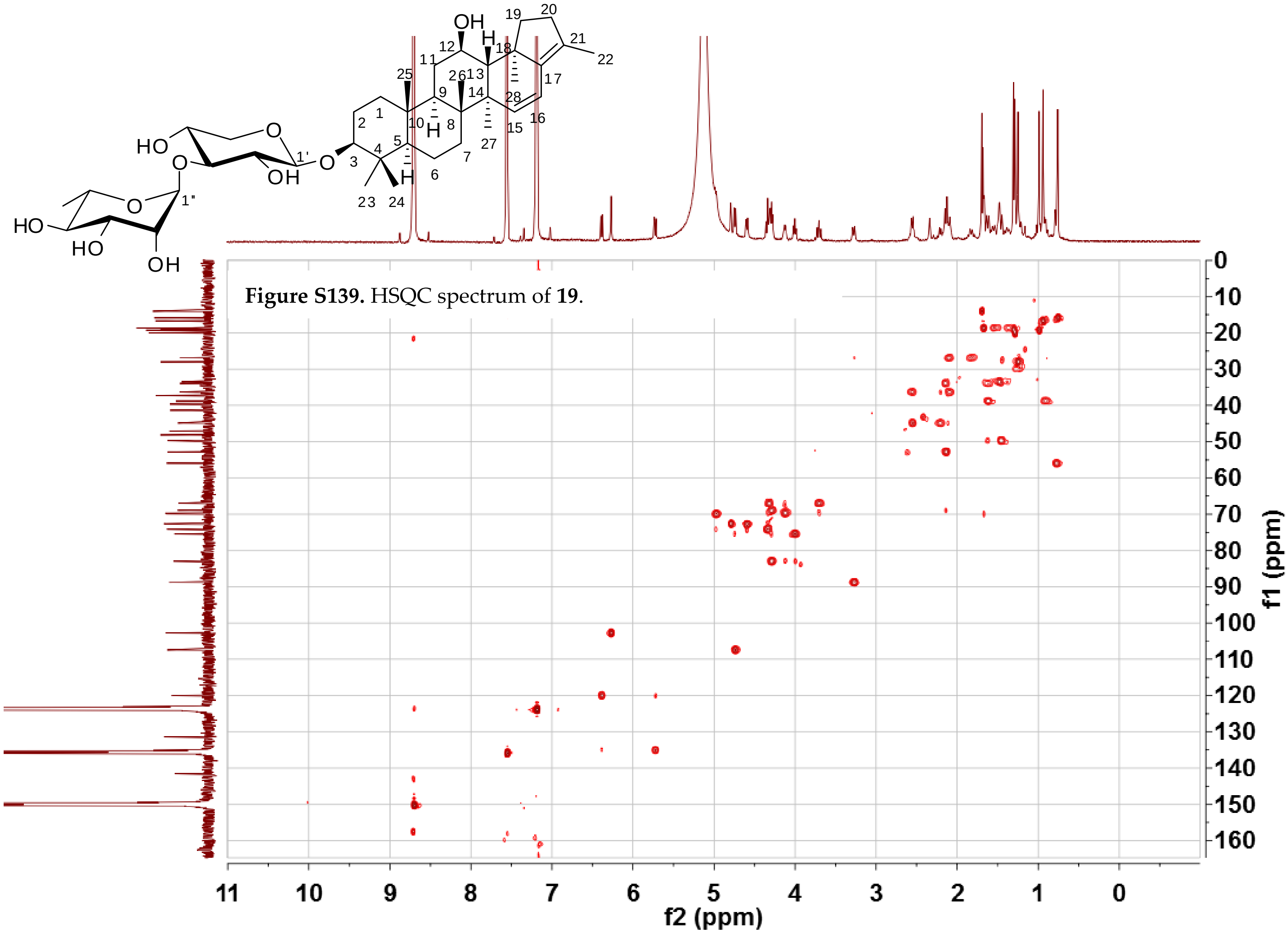

Figure S139. HSQC spectrum of **19**.

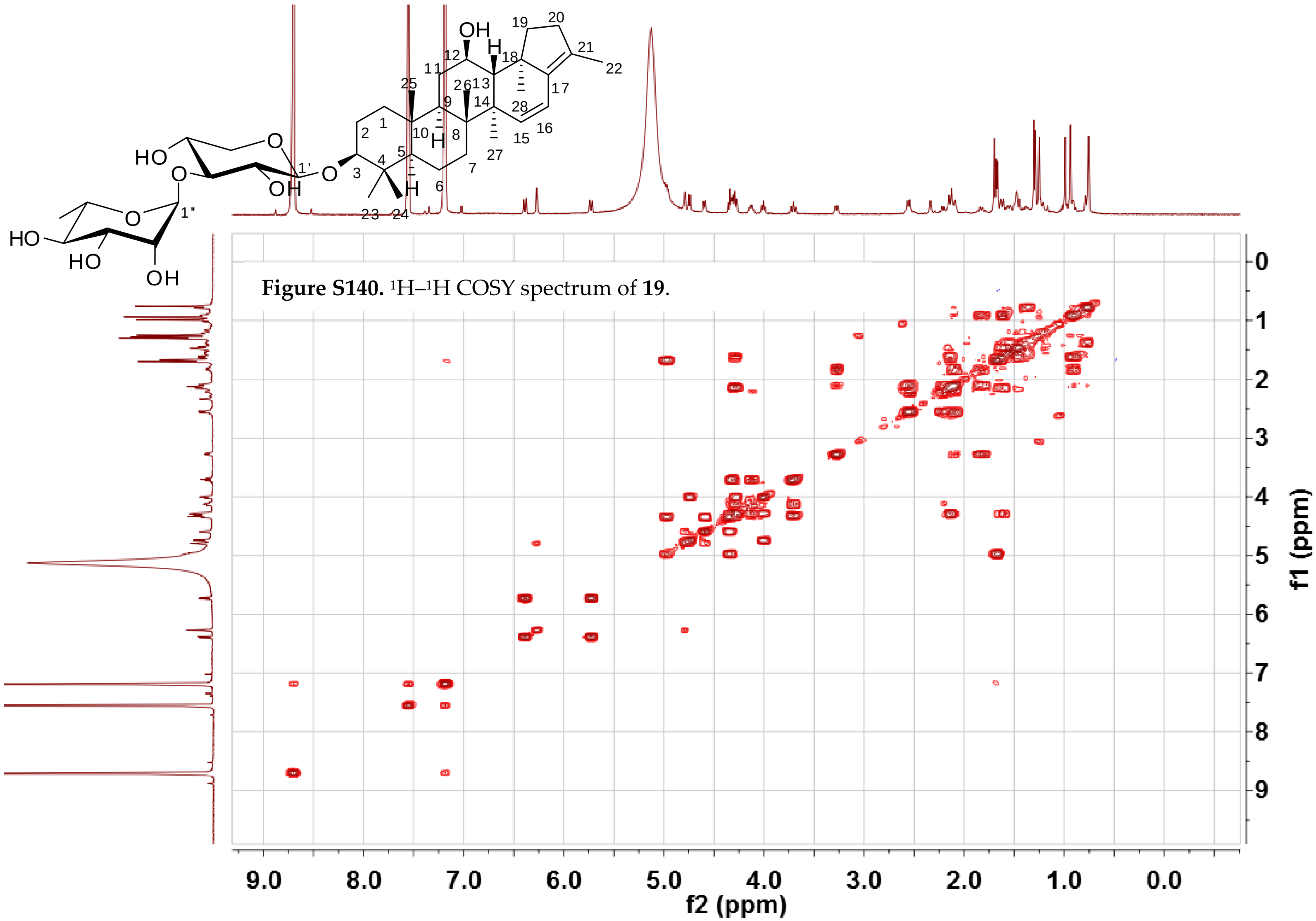

Figure S140. ¹H–¹H COSY spectrum of **19**.

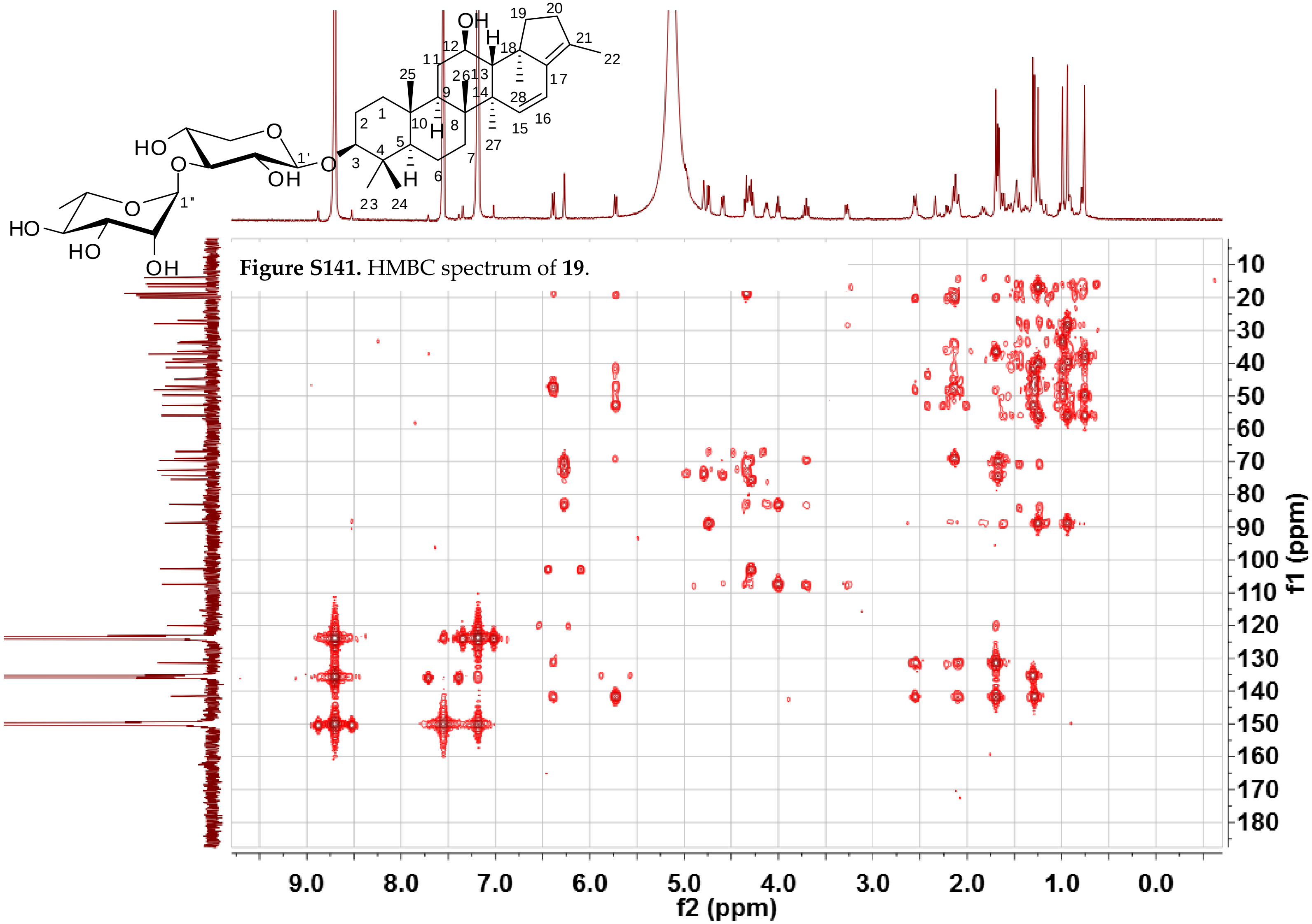

Figure S141. HMBC spectrum of **19**.

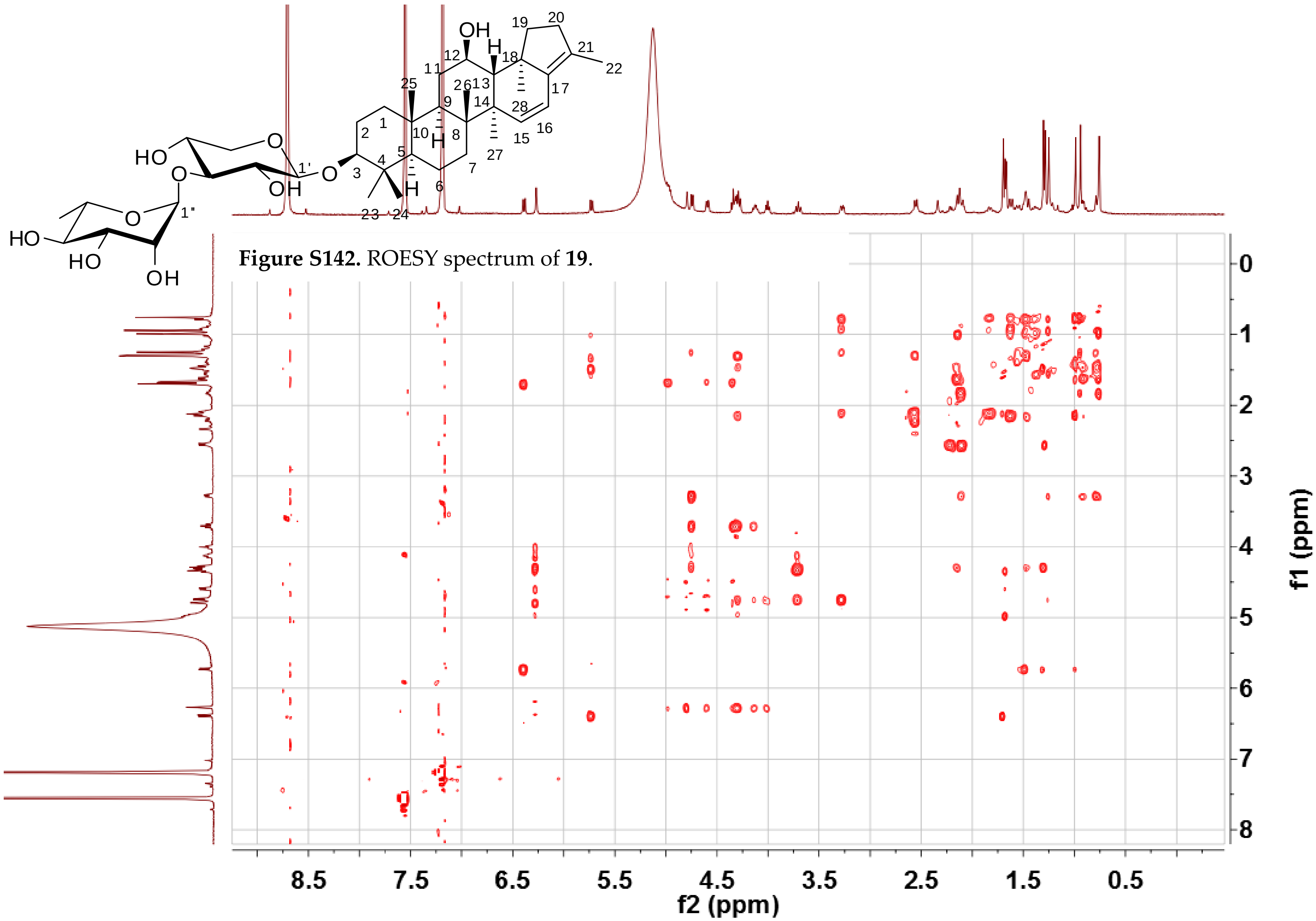

Figure S142. ROESY spectrum of **19**.

**Data Filename**	180824ESIA6.d	**Sample Name**	pdt46
**Sample Type**	Sample	**Position**	
**Instrument Name**	Agilent G6230 TOF MS	**User Name**	KIB
**Acq Method**	ESI.m	**Acquired Time**	8/24/2018 2:26:56 PM
**IRM Calibration Status**	Success	**DA Method**	ESI.m
**Comment**			

Sample Group **Info.**

Acquisition SW Version 6200 series TOF/6500 series Q-TOF B.05.01 (B5125.2)

User Spectra

Fragmentor Voltage	Collision Energy	Ionization Mode
300	0	ESI

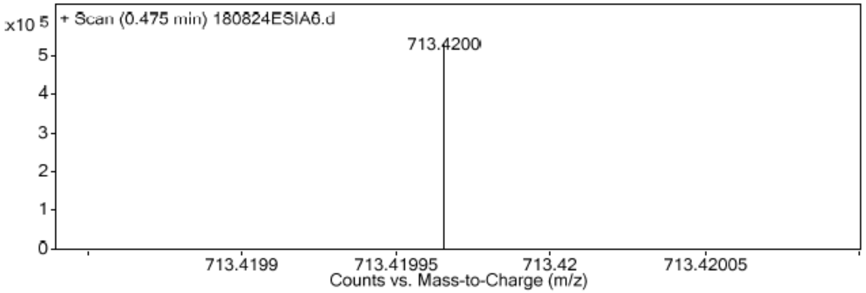

Peak List

m/z	z	Abund	Formula	Ion
112.1867	1	127159.51		
713.42	1	525814.06	C39 H62 Na O10	M+
714.4231	1	214905.25	C39 H62 Na O10	M+
729.3914	1	94761.45		
843.3773	1	502550.69		
844.3808	1	215964.44		
845.3827	1	57689.6		
1403.8481	1	57332.28		
1533.8059	1	88705.18		
1534.8091	1	78950.48		

Formula Calculator Element Limits

Element	Min	Max
C	0	200
H	0	400
O	7	15
Na	1	1

Formula Calculator Results

Formula	CalculatedMass	Mz	Diff.(mDa)	Diff. (ppm)	DBE
C39 H62 Na O10	713.4241	713.4200	4.1	5.7	8.5

--- End Of Report ---

Figure S143. HRESIMS spectrum of **19**.

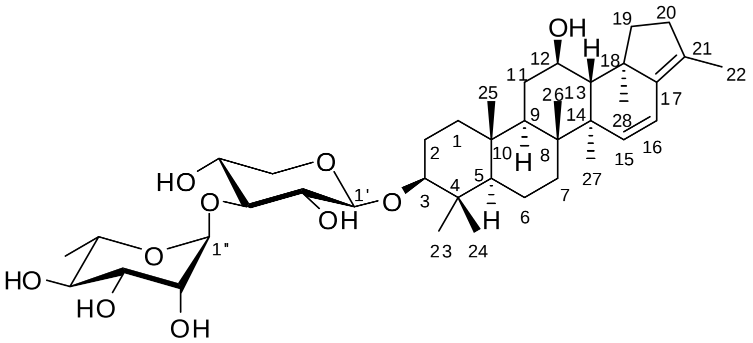

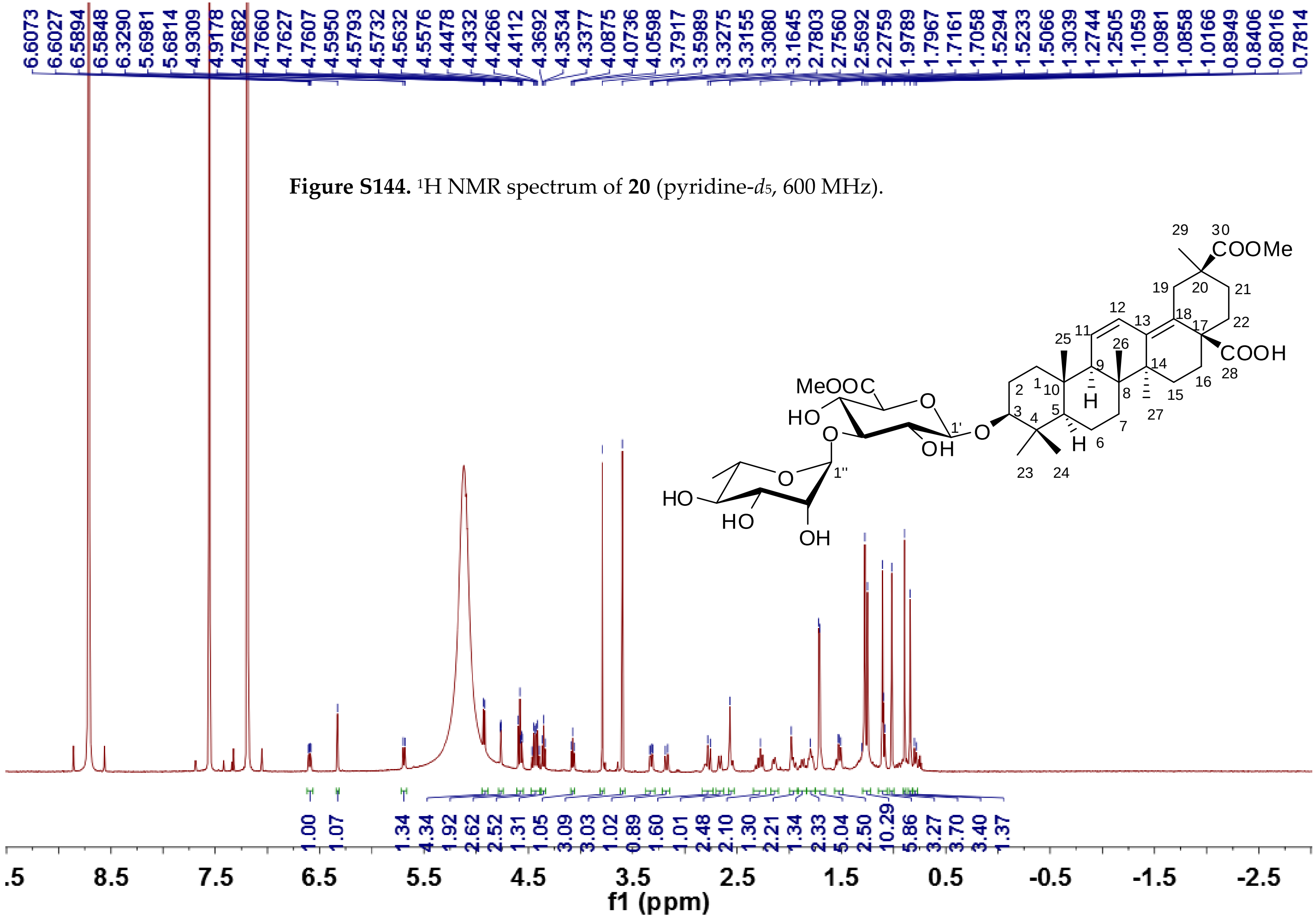

Figure S144. ^{1}H NMR spectrum of **20** (pyridine-d_5, 600 MHz).

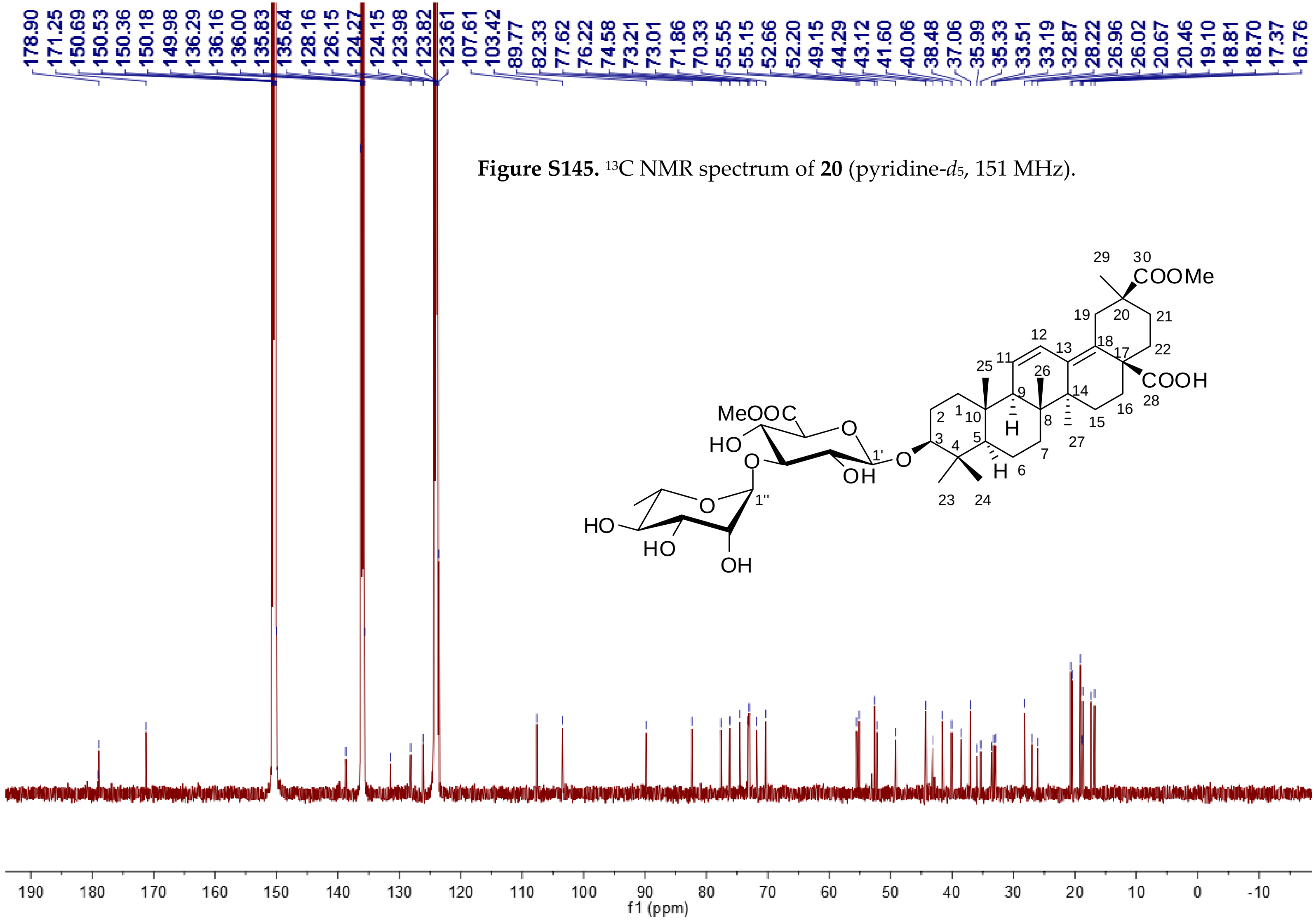

Figure S145. ^{13}C NMR spectrum of **20** (pyridine-d_5, 151 MHz).

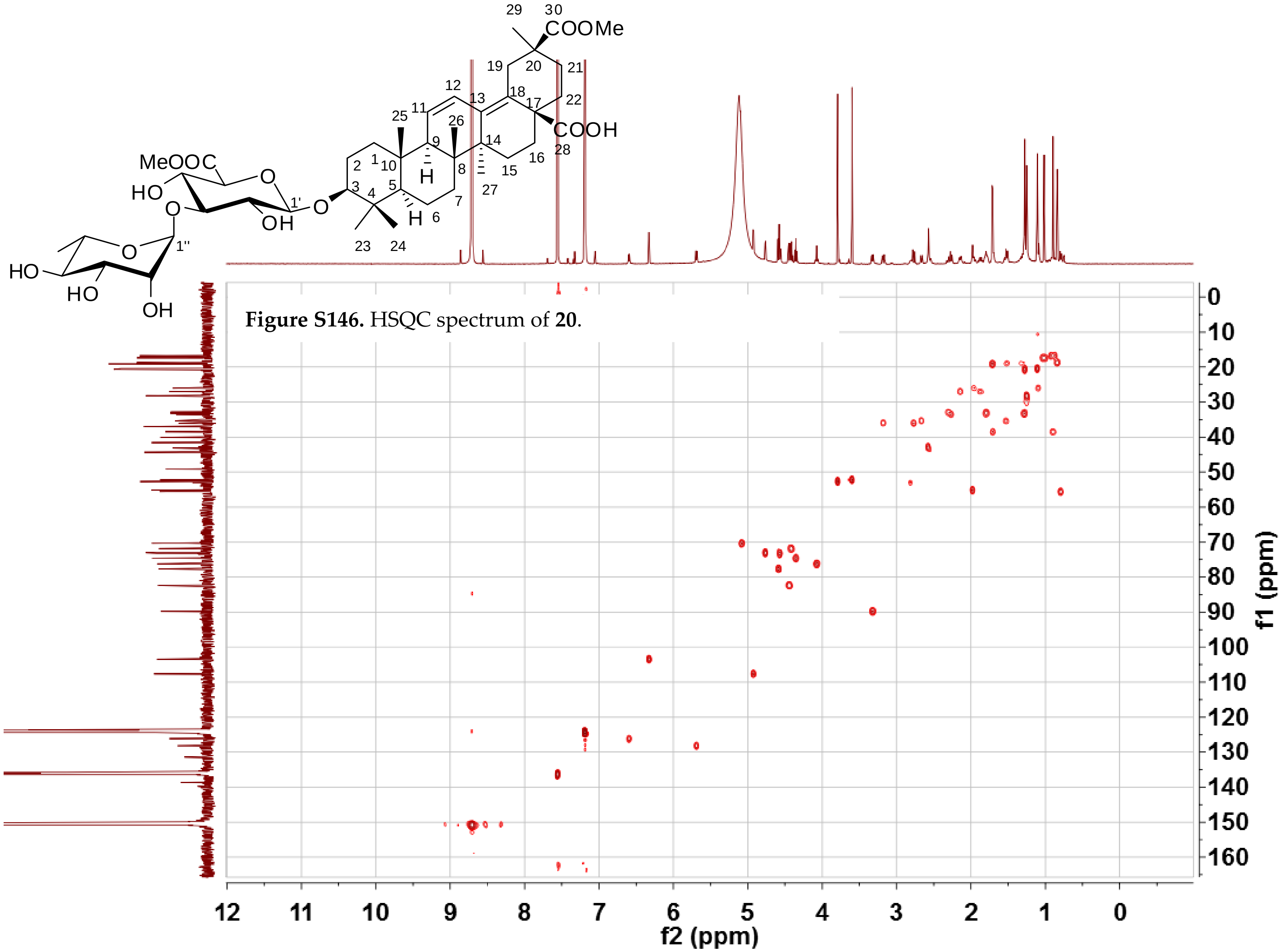

Figure S146. HSQC spectrum of **20**.

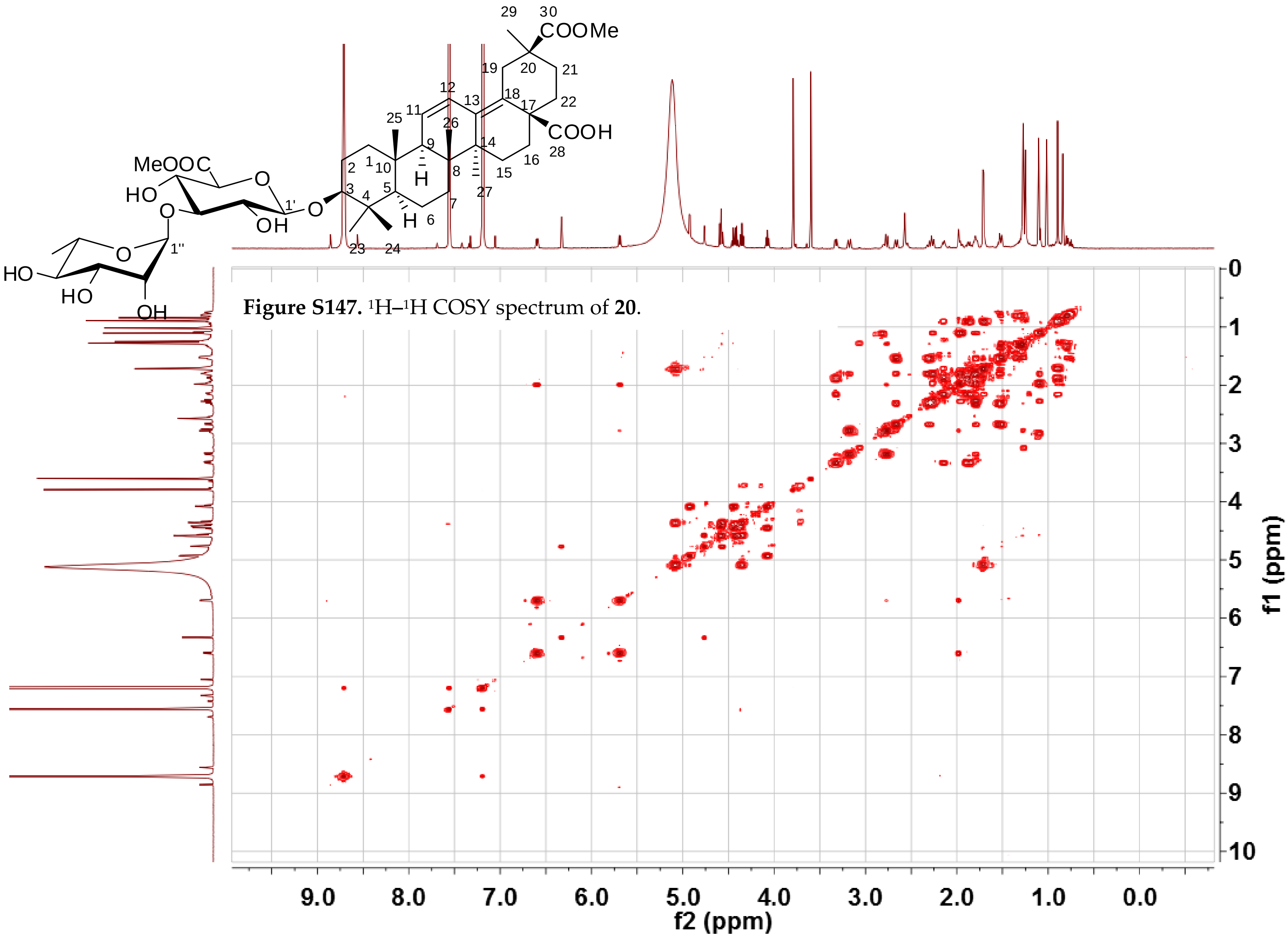

Figure S147. ^{1}H–^{1}H COSY spectrum of **20**.

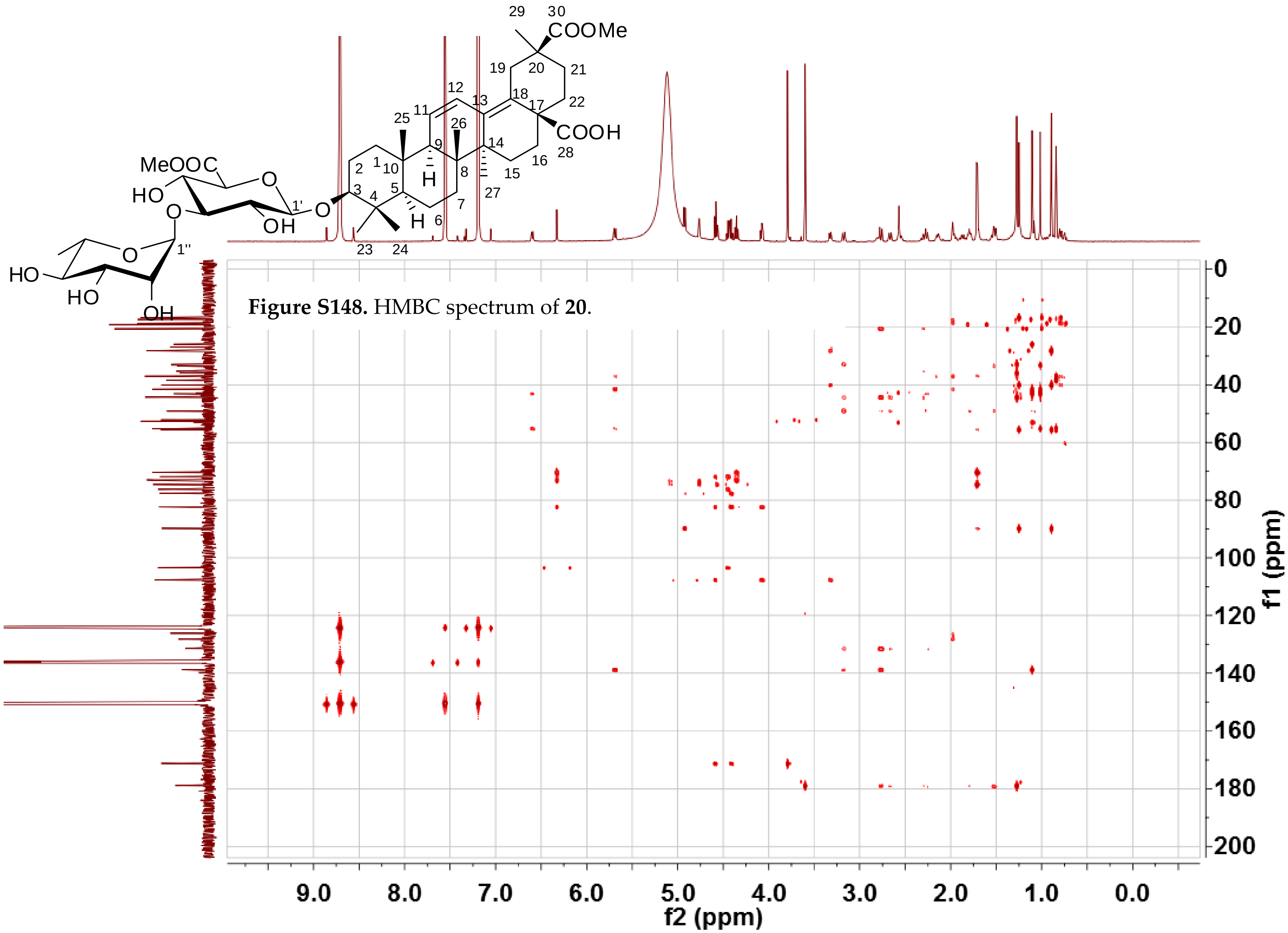

Figure S148. HMBC spectrum of **20**.

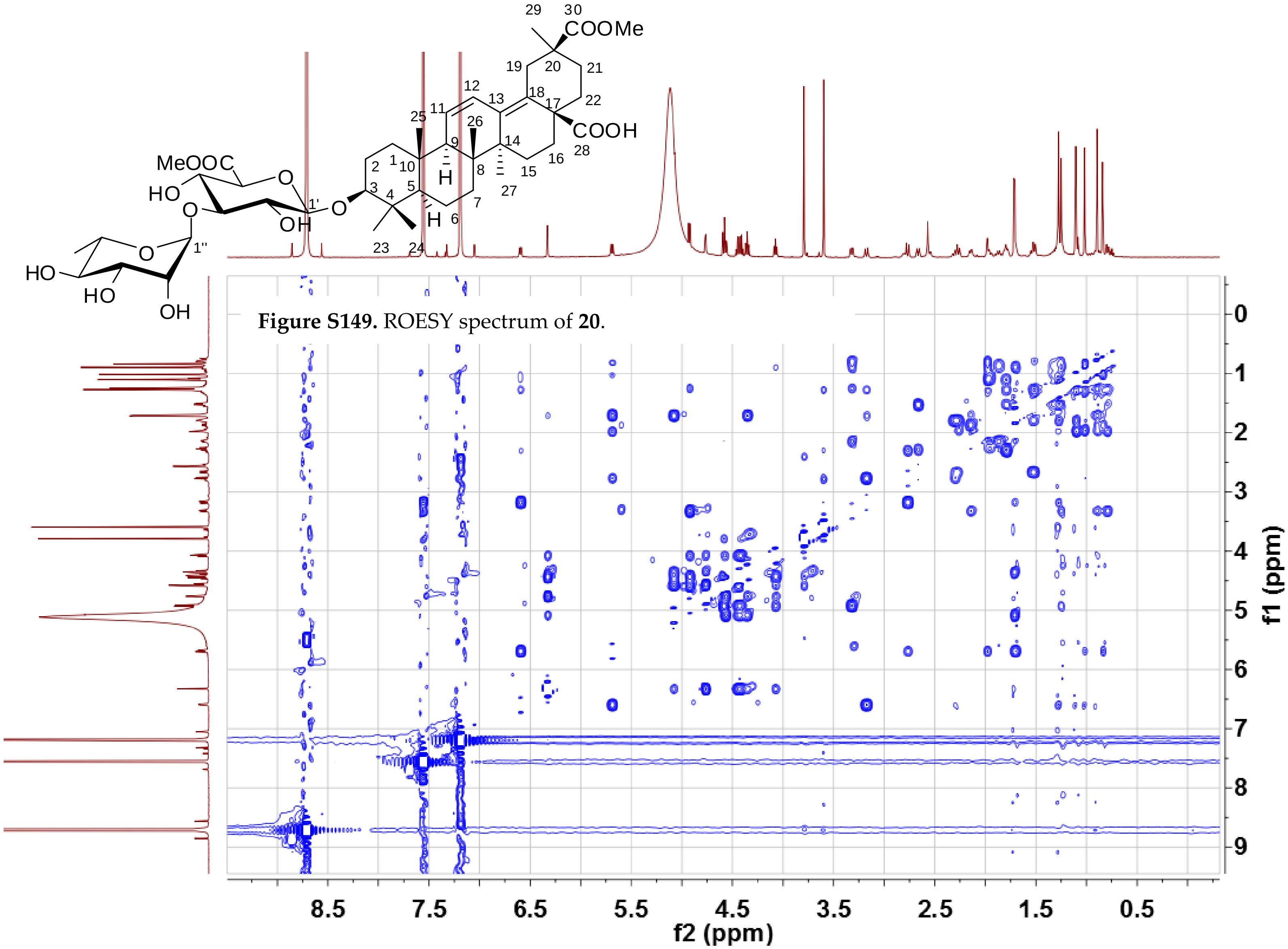

Figure S149. ROESY spectrum of **20**.

**Data Filename**	180828ESIA3.d	**Sample Name**	pdt47
**Sample Type**	Sample	**Position**	
**Instrument Name**	Agilent G6230 TOF MS	**User Name**	KIB
**Acq Method**	ESI.m	**Acquired Time**	8/28/2018 9:56:07 AM
**IRM Calibration Status**	Success	**DA Method**	ESI.m
**Comment**			

Sample Group **Info.**

Acquisition SW 6200 series TOF/6500 series
Version Q-TOF B.05.01 (B5125.2)

User Spectra

Fragmentor Voltage	Collision Energy	Ionization Mode
200	0	ESI

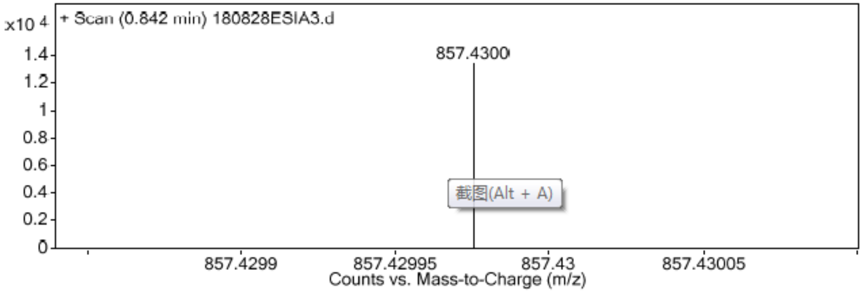

Peak List

m/z	z	Abund	Formula	Ion
102.1279	1	62256.19		
111.1809		17327.26		
112.1872	1	70639.46		
123.0914	1	13701.84		
144.0801	1	14600.05		
144.2429		35668.02		
145.2495	1	173775.77		
146.2524	1	14833.91		
152.2178	1	24149.91		
857.43	1	13353.48	C44 H66 Na O15	M+

Formula Calculator Element Limits

Element	Min	Max
C	0	200
H	0	400
O	11	19
Na	1	1

Formula Calculator Results

Formula	CalculatedMass	Mz	Diff.(mDa)	Diff. (ppm)	DBE
C44 H66 Na O15	857.4299	857.4300	-0.1	0.1	11.5

--- End Of Report ---

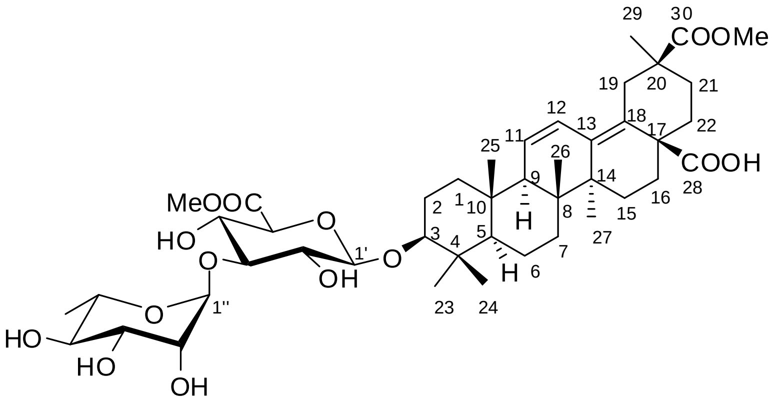

Figure S150. HRESIMS spectrum of **20**.

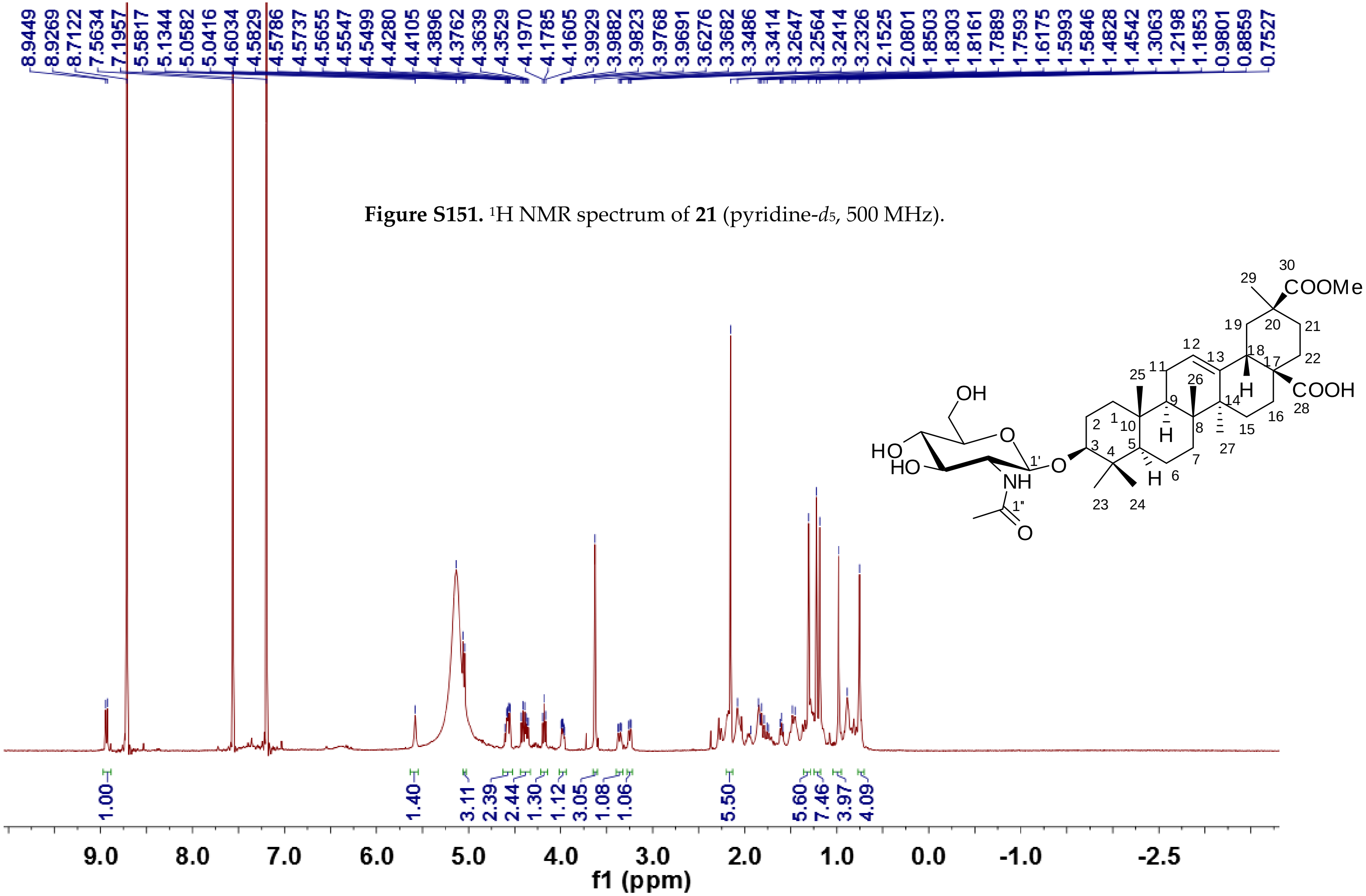

Figure S151. ^{1}H NMR spectrum of **21** (pyridine-d_5, 500 MHz).

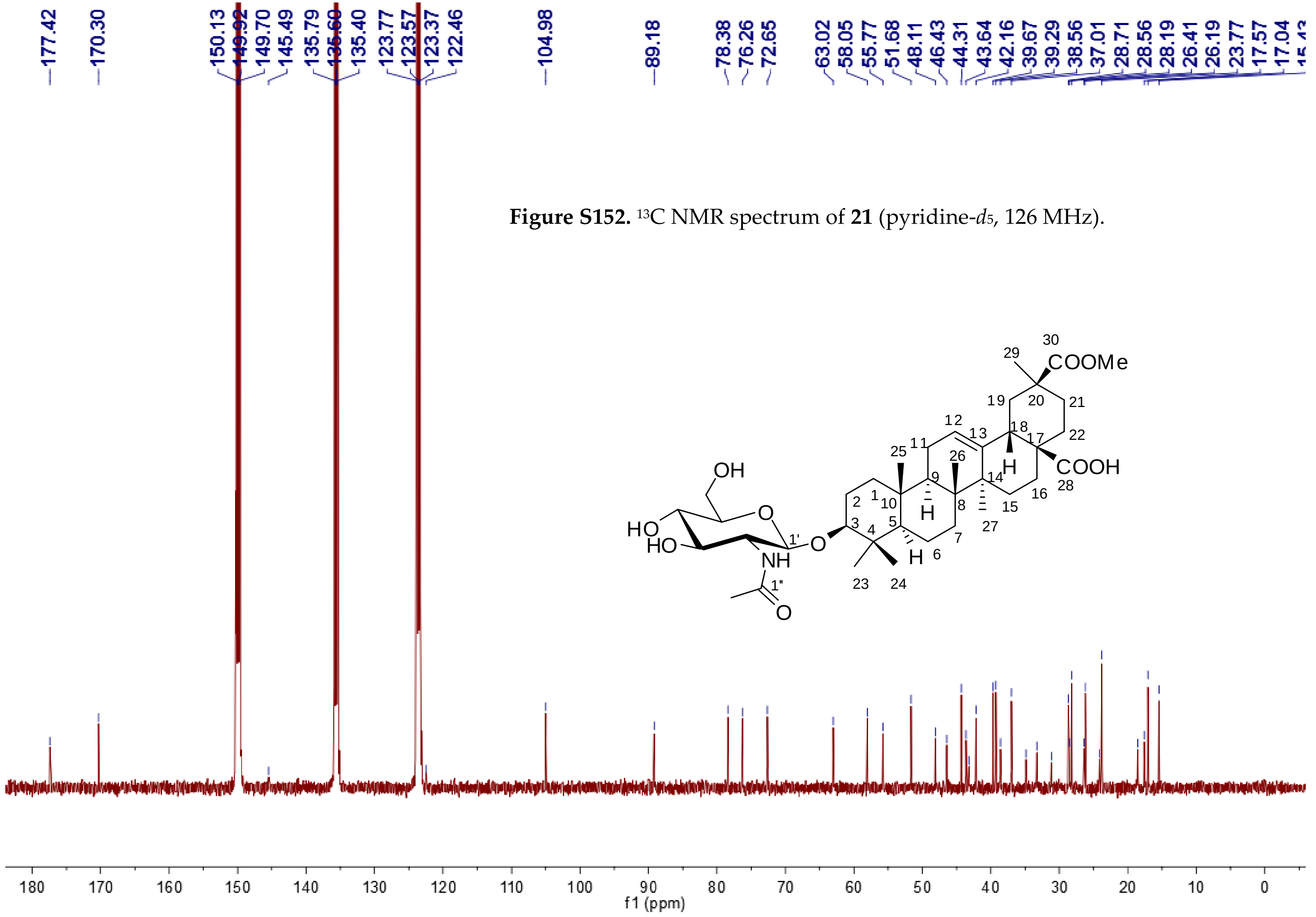

Figure S152. ^{13}C NMR spectrum of **21** (pyridine-*d*5, 126 MHz).

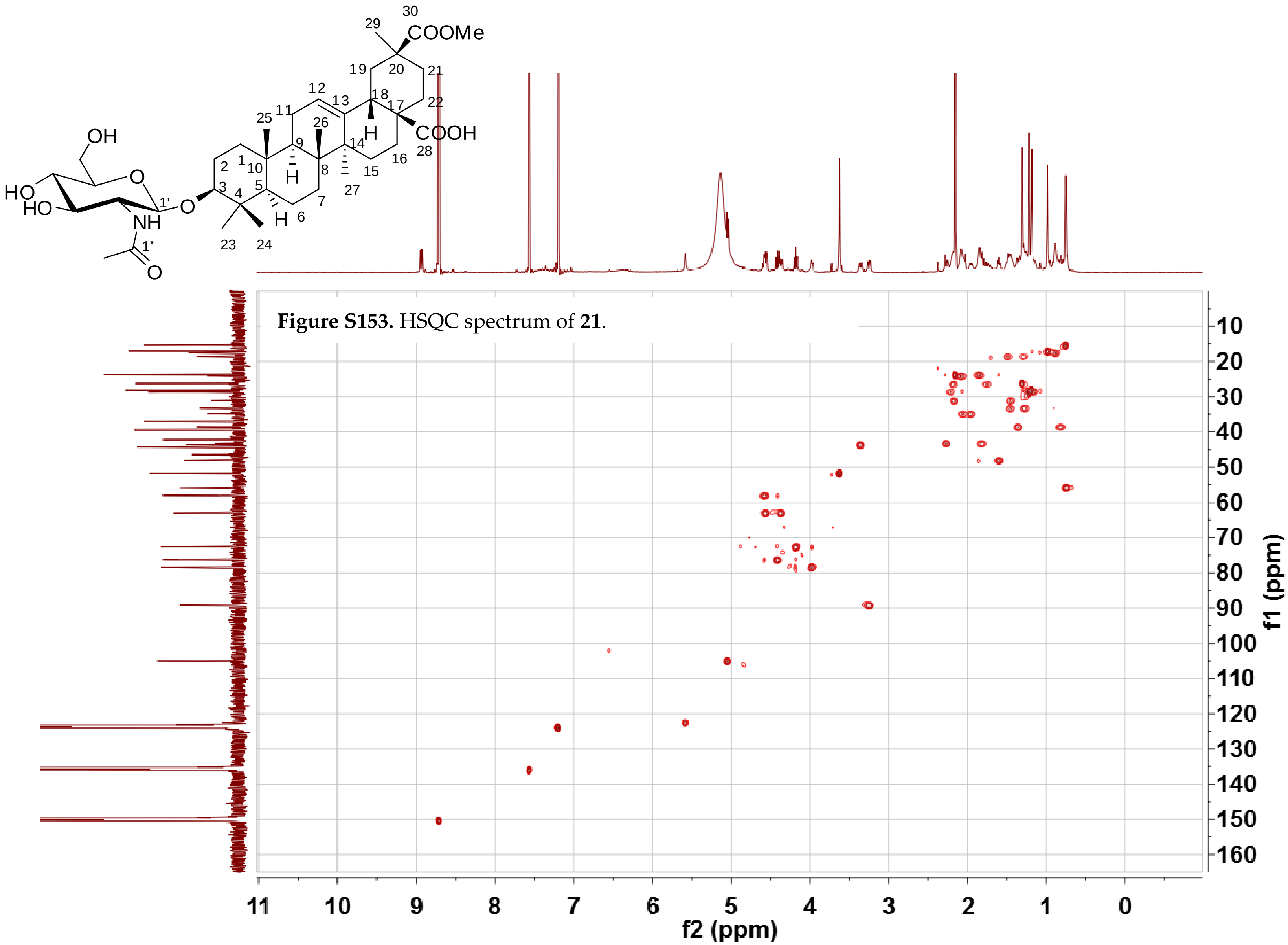

Figure S153. HSQC spectrum of **21**.

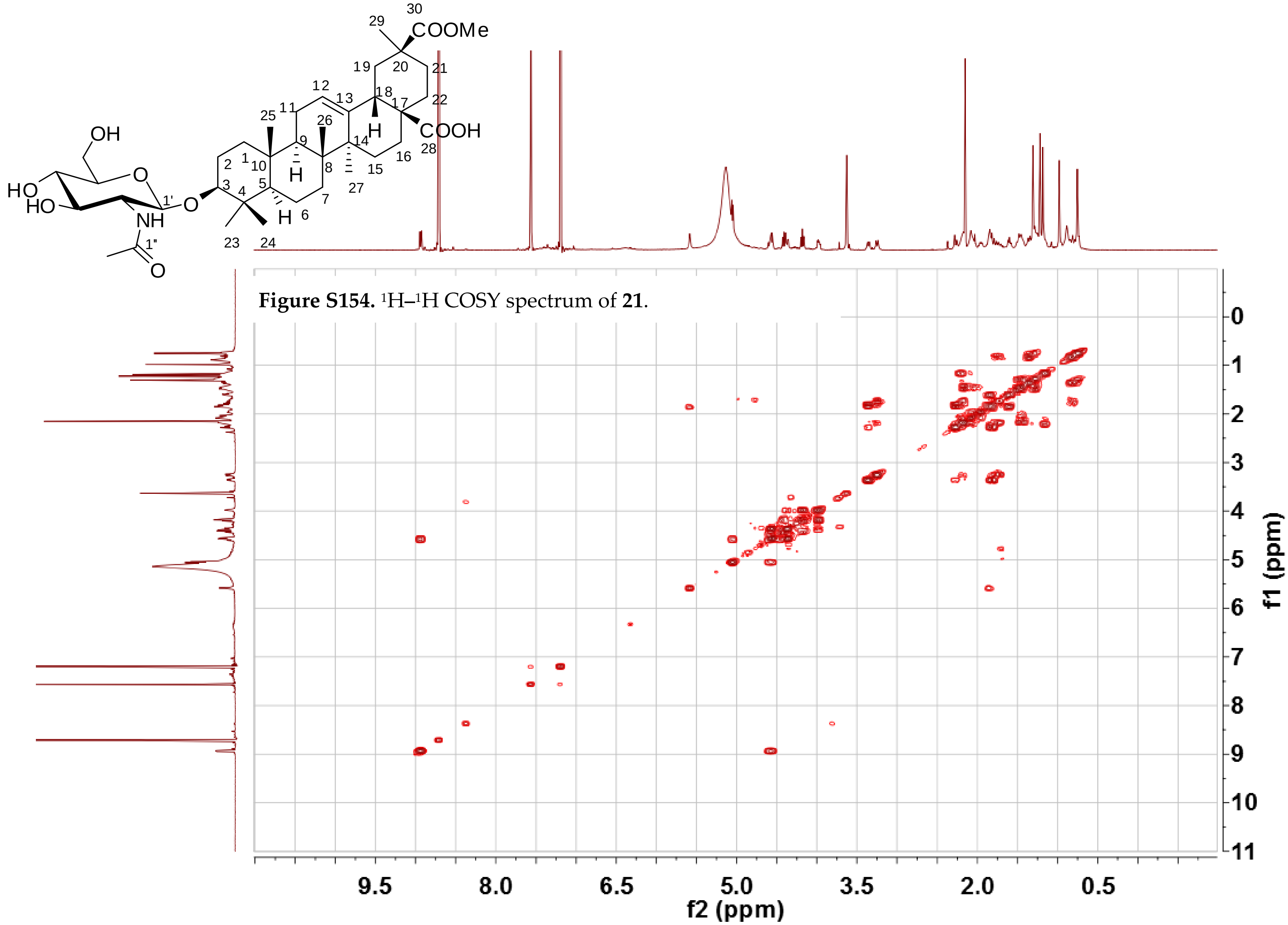

Figure S154. ^{1}H–^{1}H COSY spectrum of **21**.

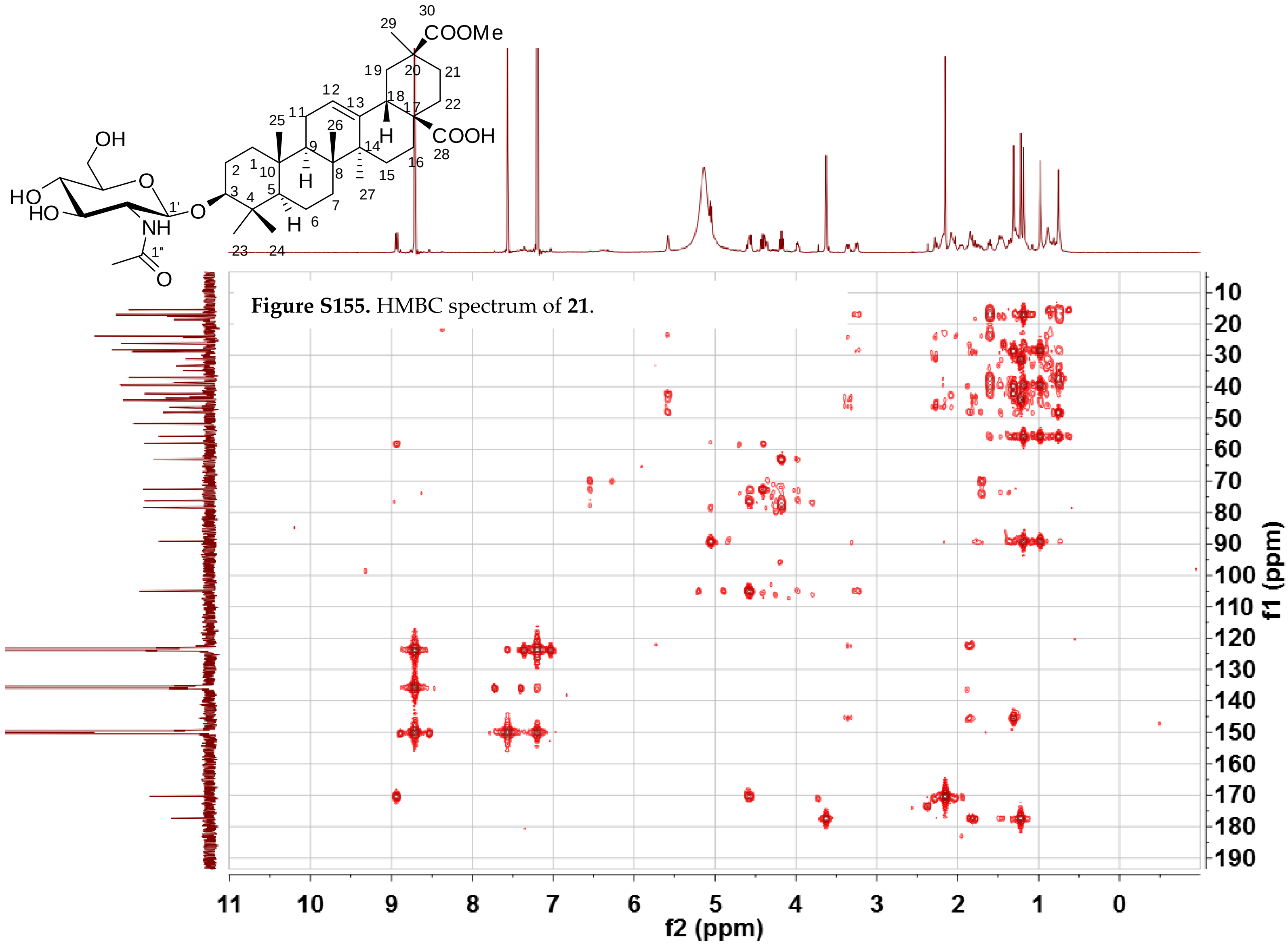

Figure S155. HMBC spectrum of **21**.

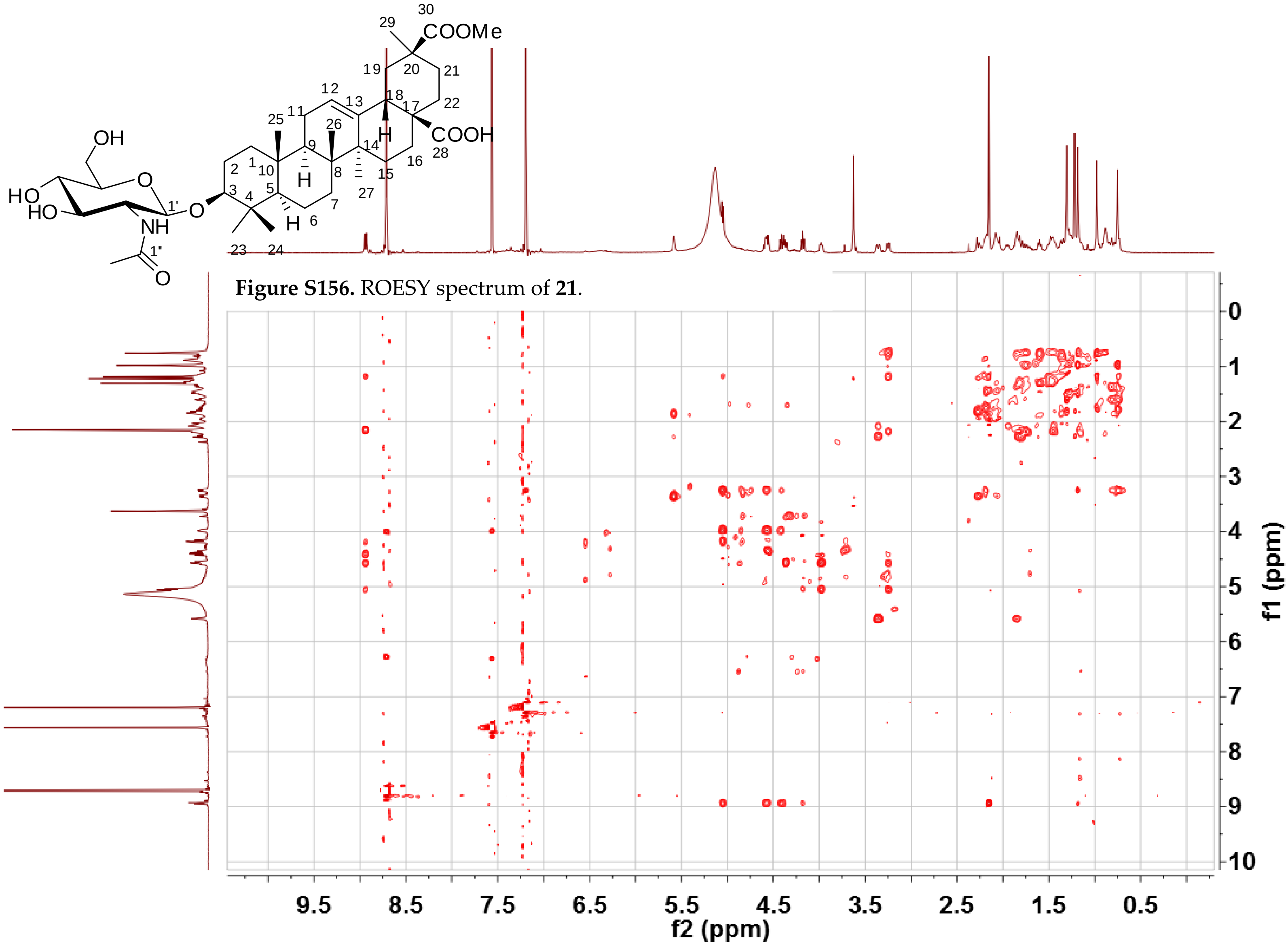

Figure S156. ROESY spectrum of **21**.

**Data Filename**	180829ESIA8.d	**Sample Name**	pdt51
**Sample Type**	Sample	**Position**	
**Instrument Name**	Agilent G6230 TOF MS	**User Name**	KIB
**Acq Method**	ESI.m	**Acquired Time**	8/29/2018 1:55:15 PM
**IRM Calibration Status**	Success	**DA Method**	ESI.m
**Comment**			

Sample Group **Info.**

Acquisition SW Version 6200 series TOF/6500 series Q-TOF B.05.01 (B5125.2)

Peak List

m/z	z	Abund	Formula	Ion
103.0077		5729.59		
107.037	1	5492.39		
111.1819		4861.88		
112.1882	1	26780.22		
145.2506	1	10904.01		
726.4201	1	46460.01	C39 H61 N Na O10	M+
727.4231	1	20077.11	C39 H61 N Na O10	M+
742.3912	2	8471.38		
759.4663	1	7527.42		
856.3787	1	5568.69		

Formula Calculator Element Limits

Element	Min	Max
C	0	200
H	0	400
O	7	14
Na	1	1
N	1	1

Formula Calculator Results

Formula	CalculatedMass	Mz	Diff.(mDa)	Diff. (ppm)	DBE
C39 H61 N Na O10	726.4193	726.4201	-0.8	1.1	9.5

--- End Of Report ---

User Spectra

Fragmentor Voltage	Collision Energy	Ionization Mode
200	0	ESI

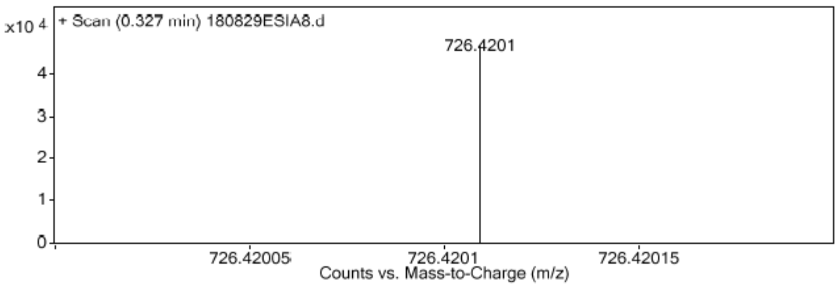

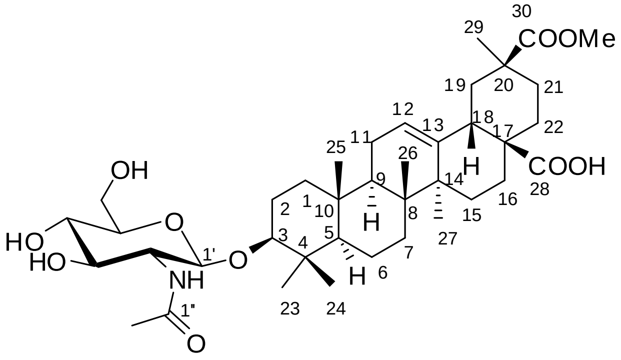

Figure S157. HRESIMS spectrum of **21**.

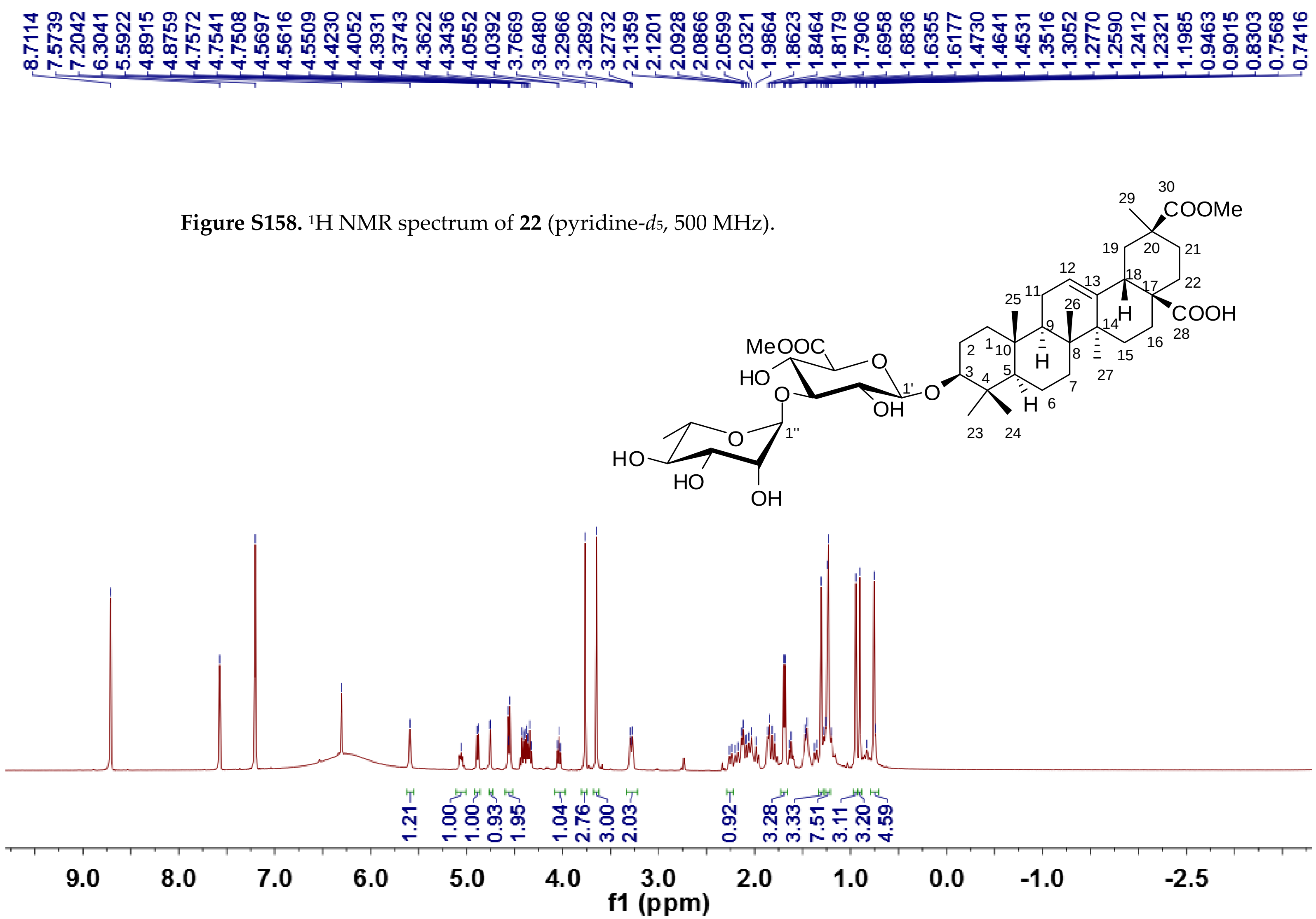

Figure S158. ^{1}H NMR spectrum of **22** (pyridine-*d*$_5$, 500 MHz).

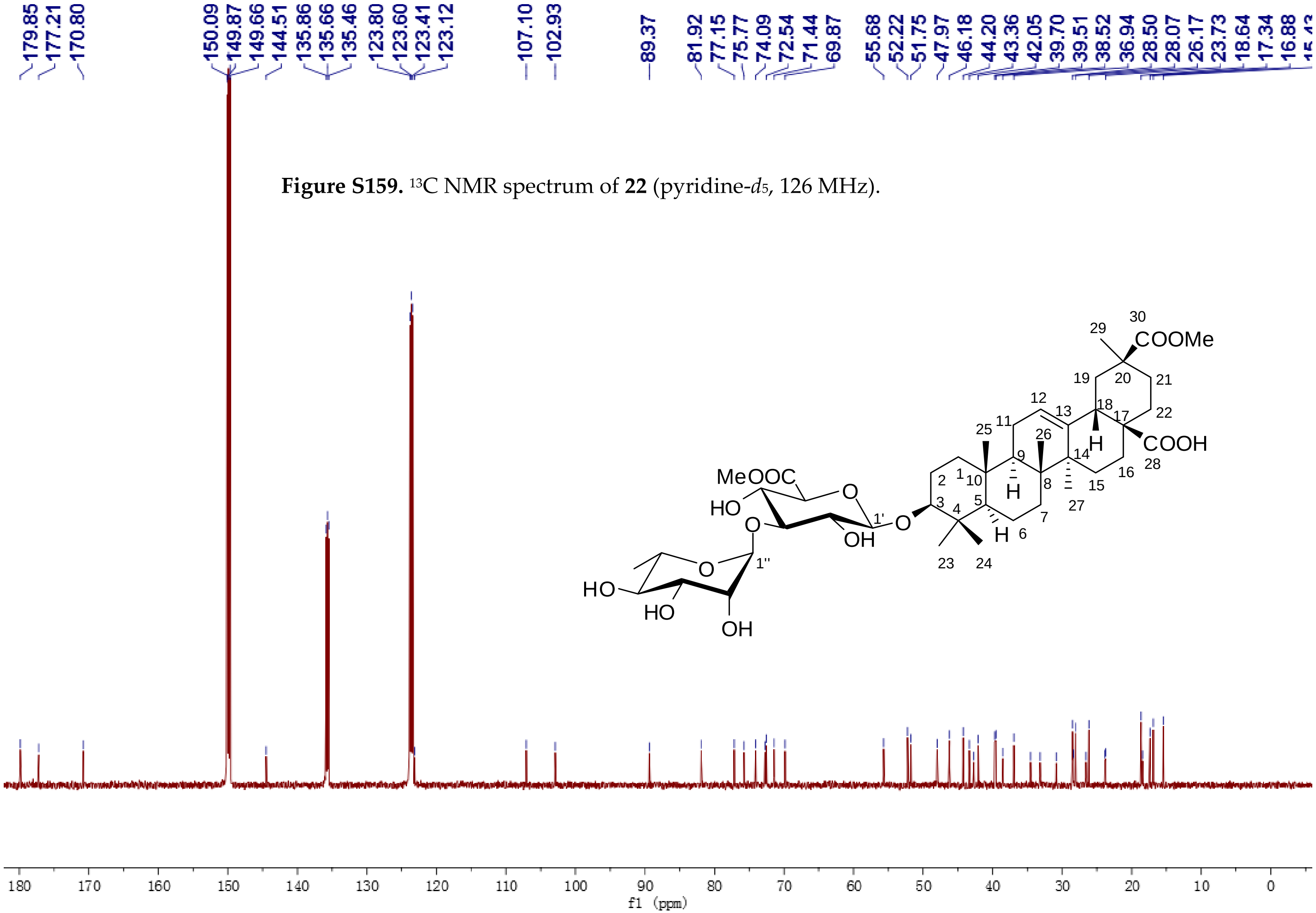

Figure S159. ^{13}C NMR spectrum of **22** (pyridine-d_5, 126 MHz).

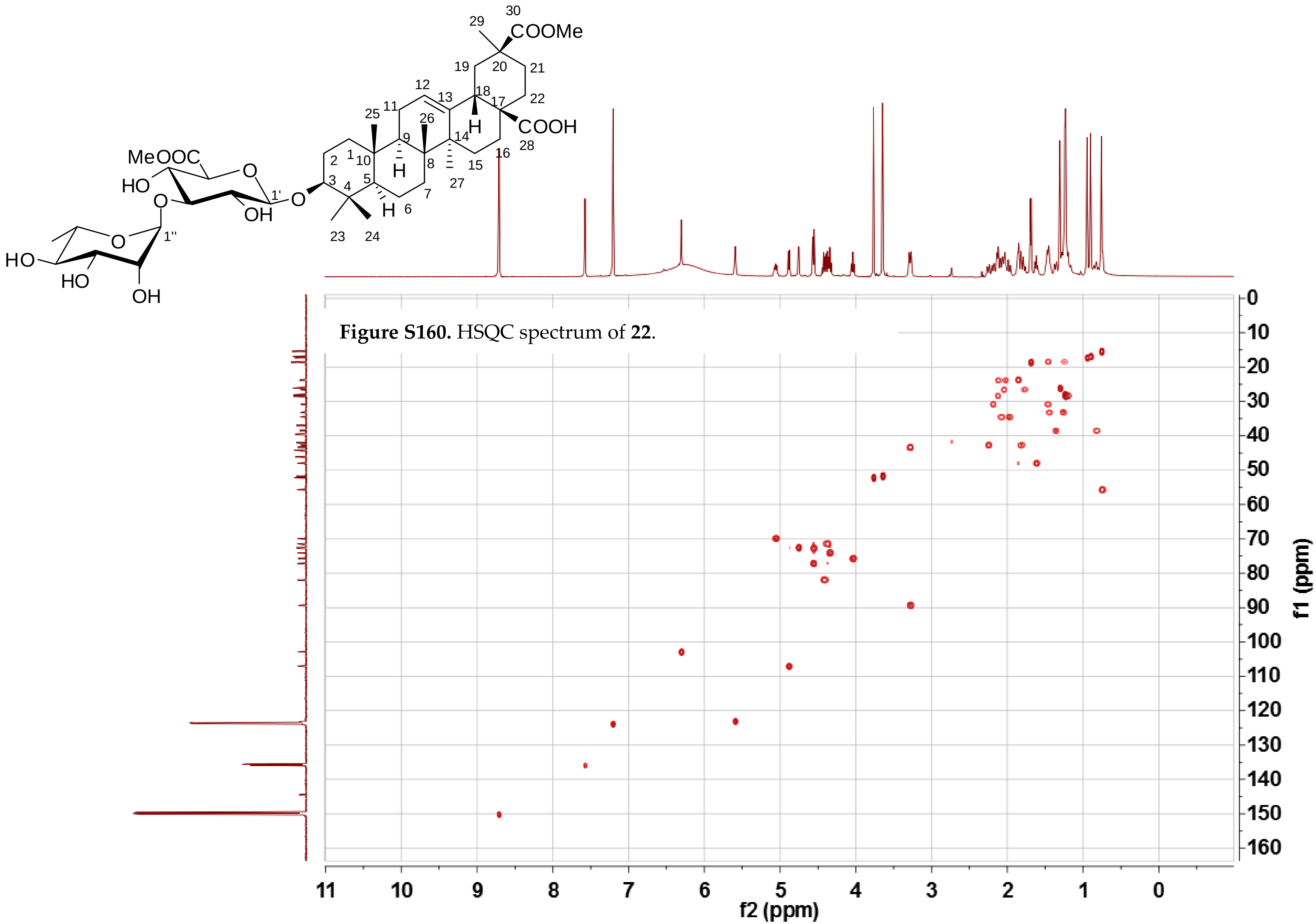

Figure S160. HSQC spectrum of **22**.

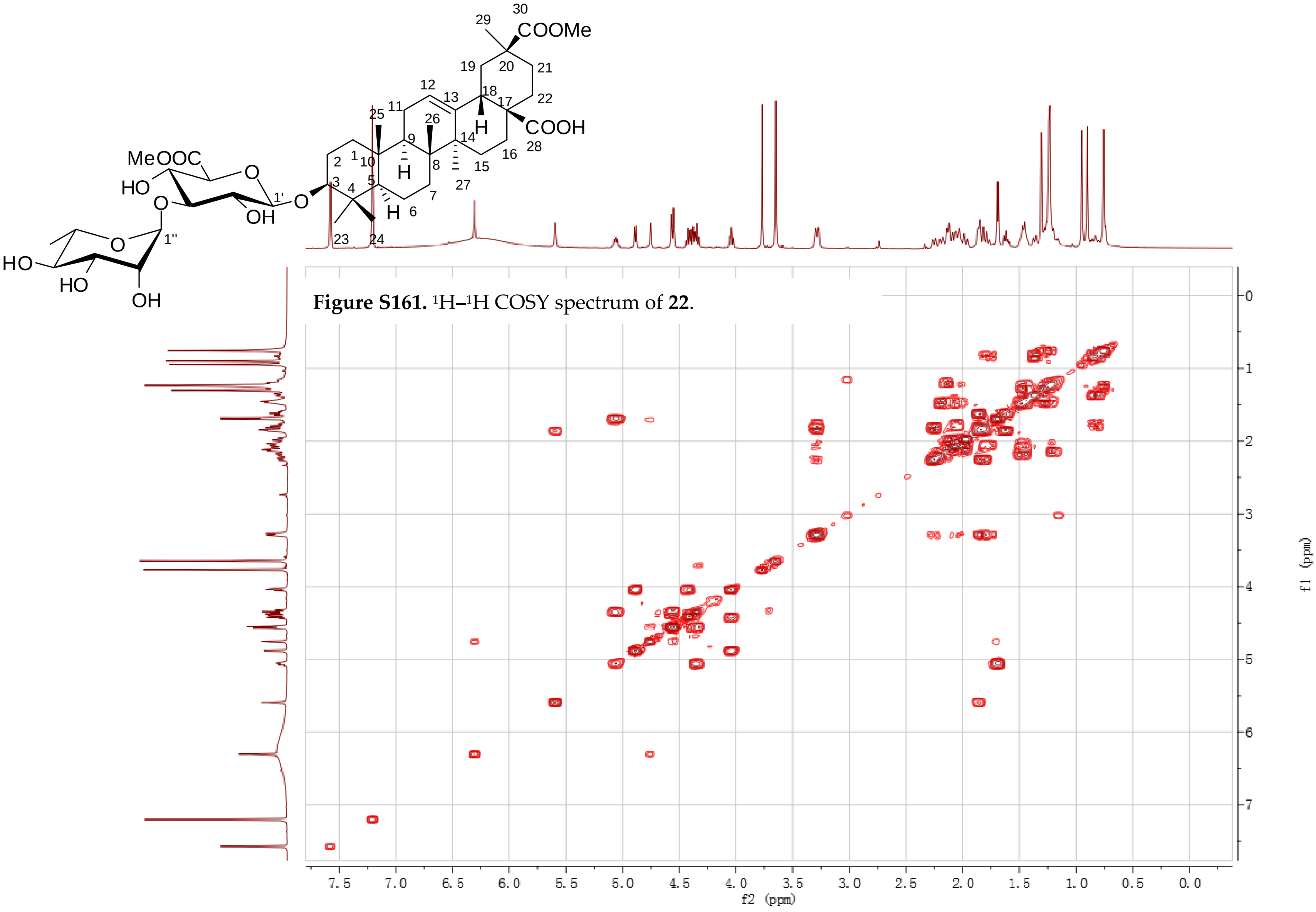

Figure S161. ^{1}H–^{1}H COSY spectrum of **22**.

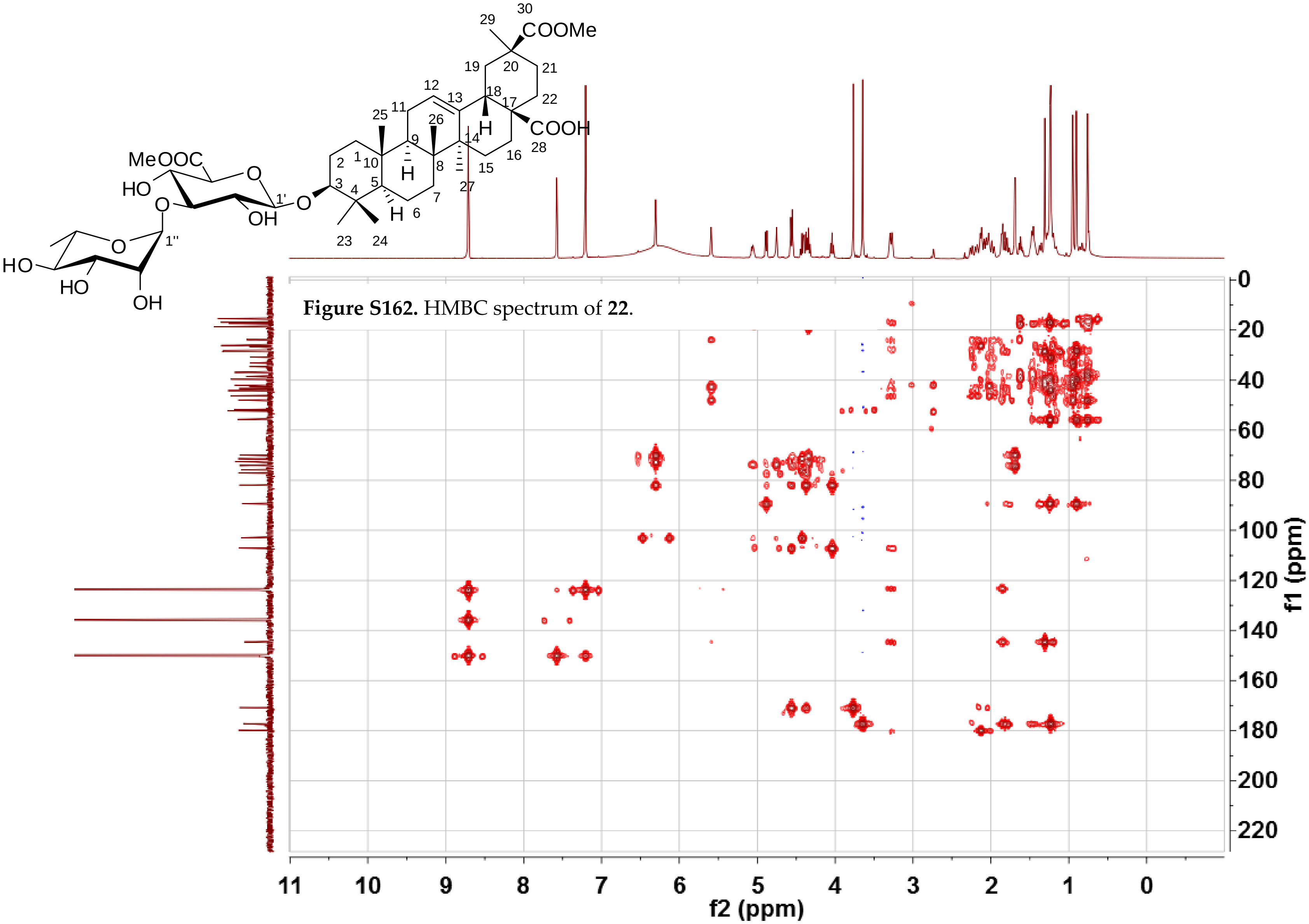

Figure S162. HMBC spectrum of **22**.

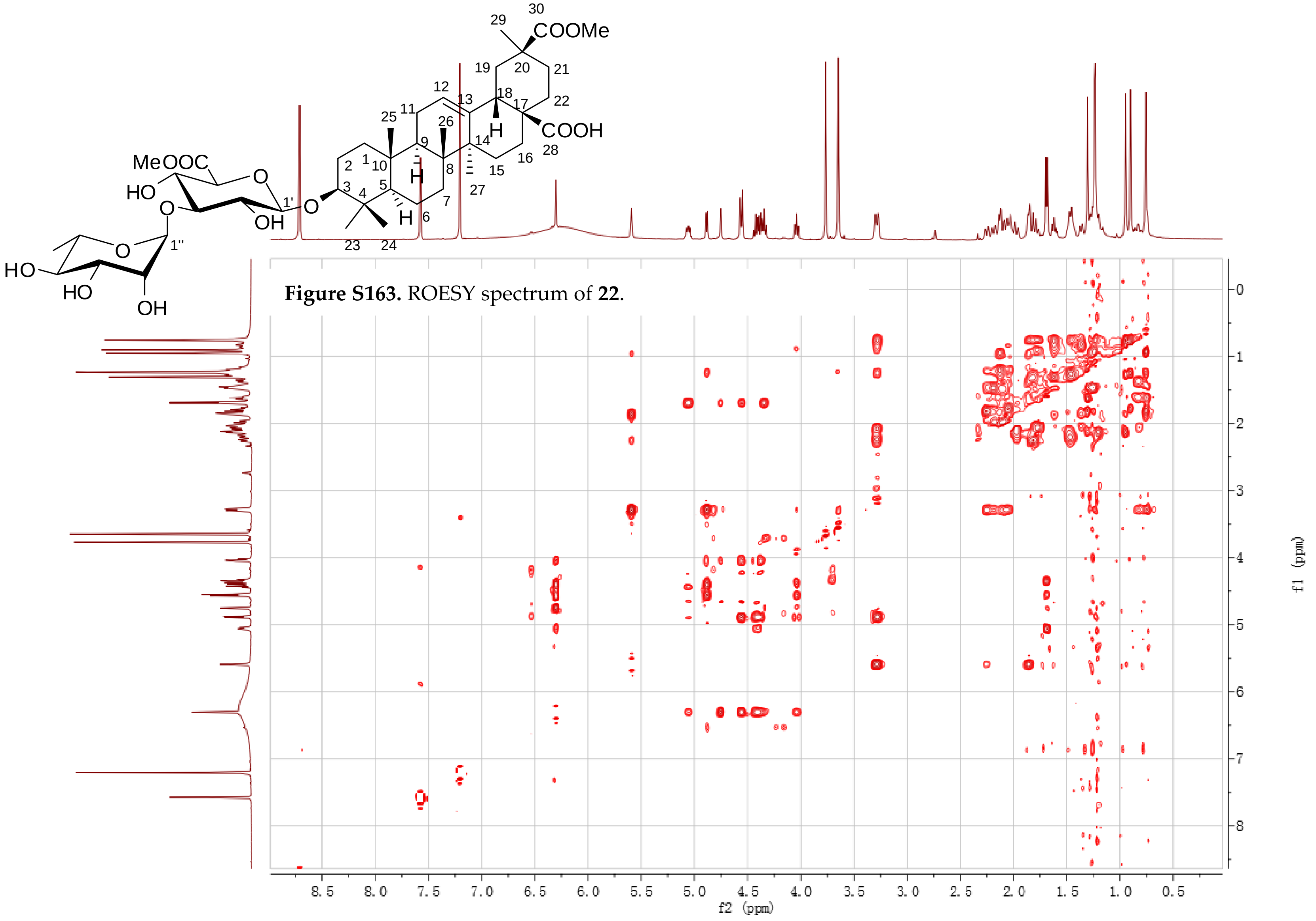

Figure S163. ROESY spectrum of **22**.

Figure S164. HRESIMS spectrum of **22**.

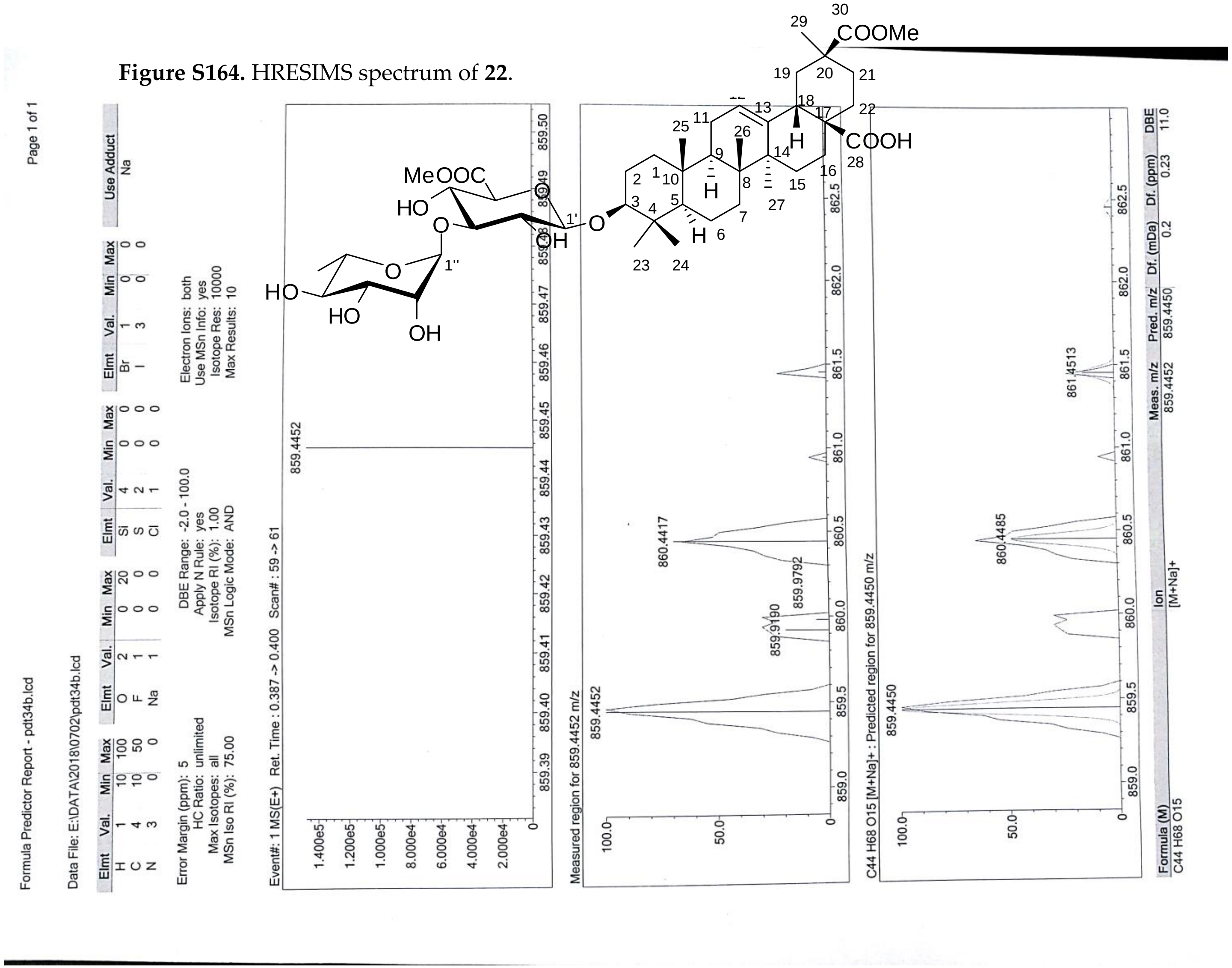

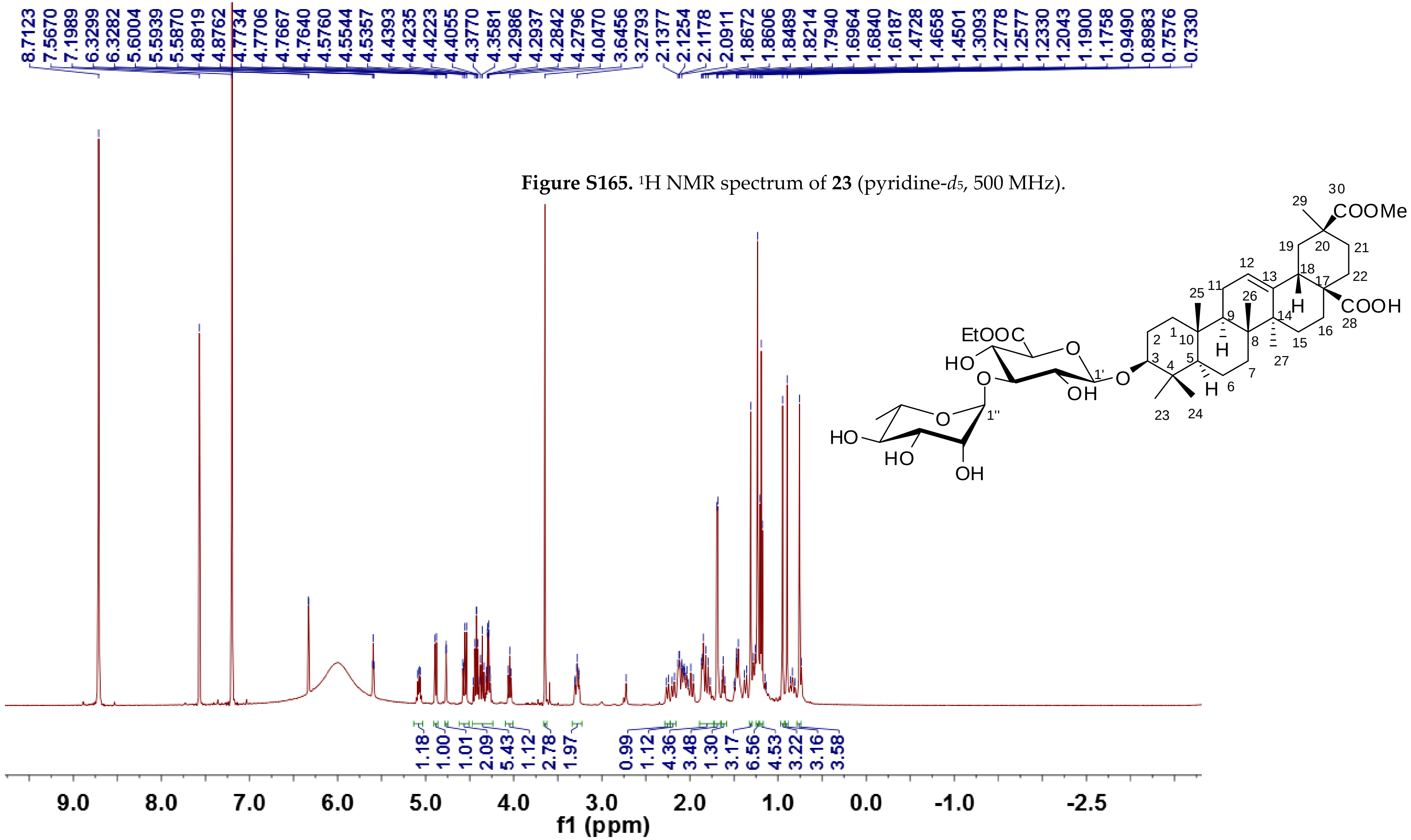

Figure S165. ^{1}H NMR spectrum of **23** (pyridine-d_5, 500 MHz).

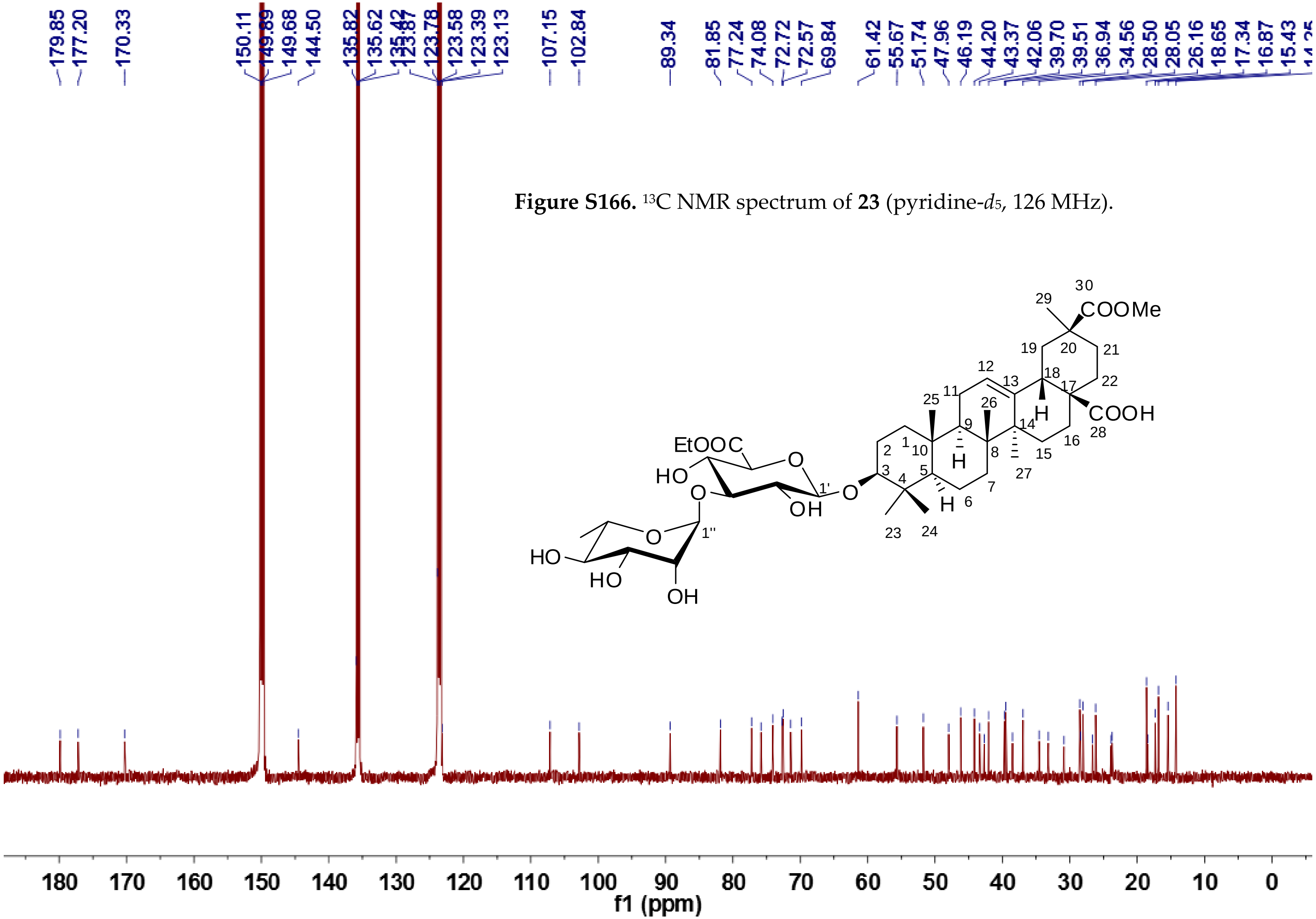

Figure S166. ^{13}C NMR spectrum of **23** (pyridine-d_5, 126 MHz).

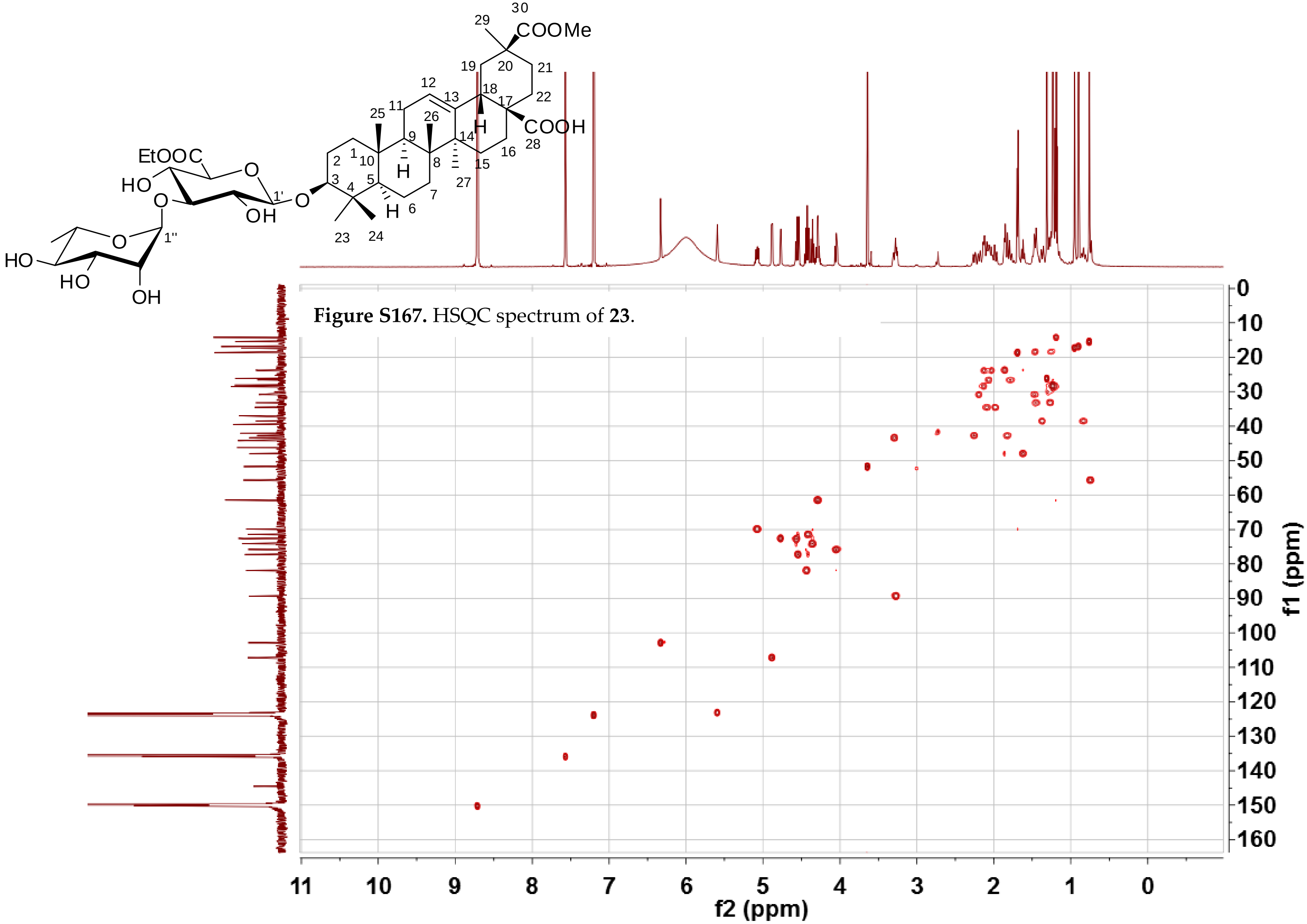

Figure S167. HSQC spectrum of **23**.

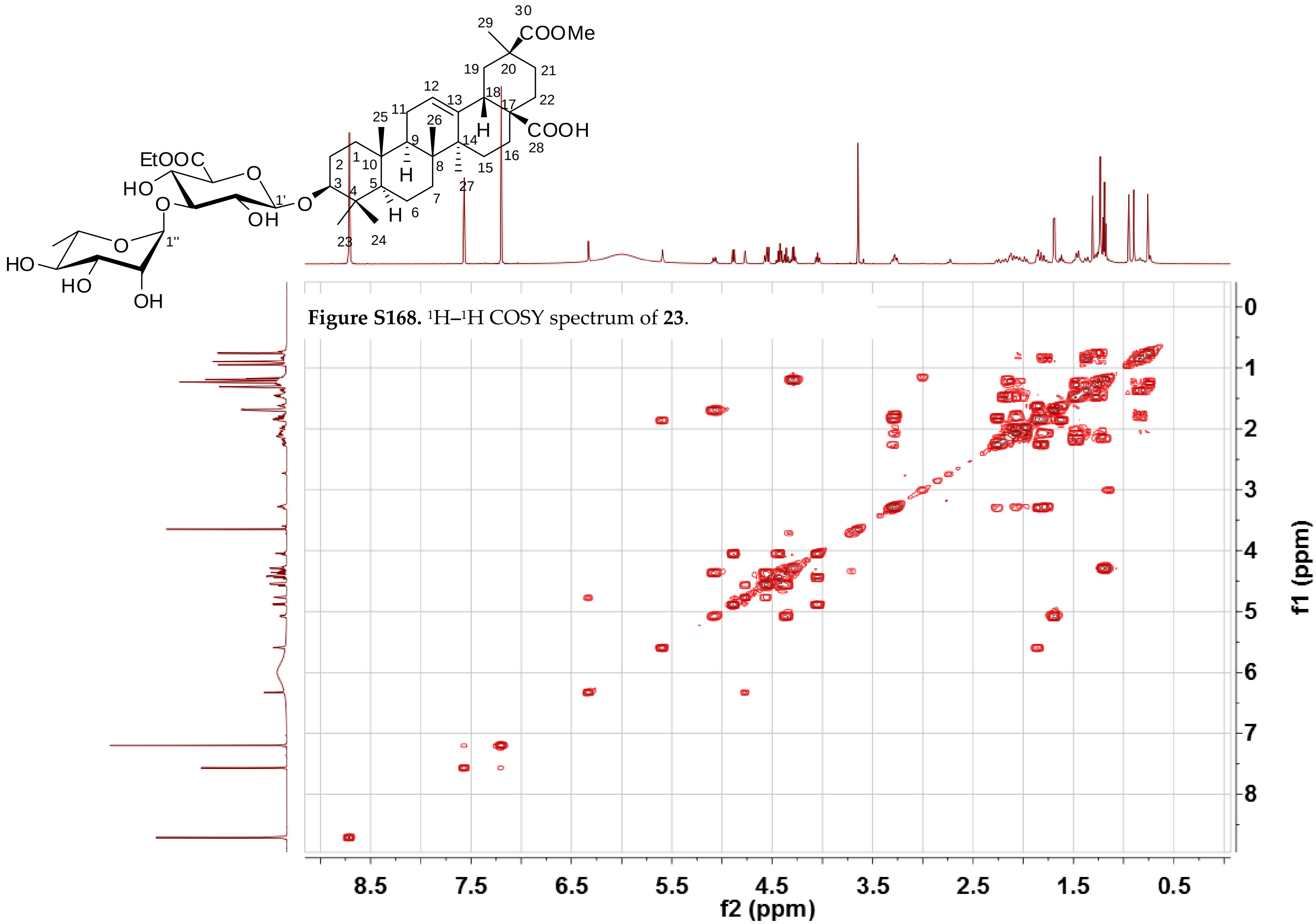

Figure S168. ^1H–^1H COSY spectrum of **23**.

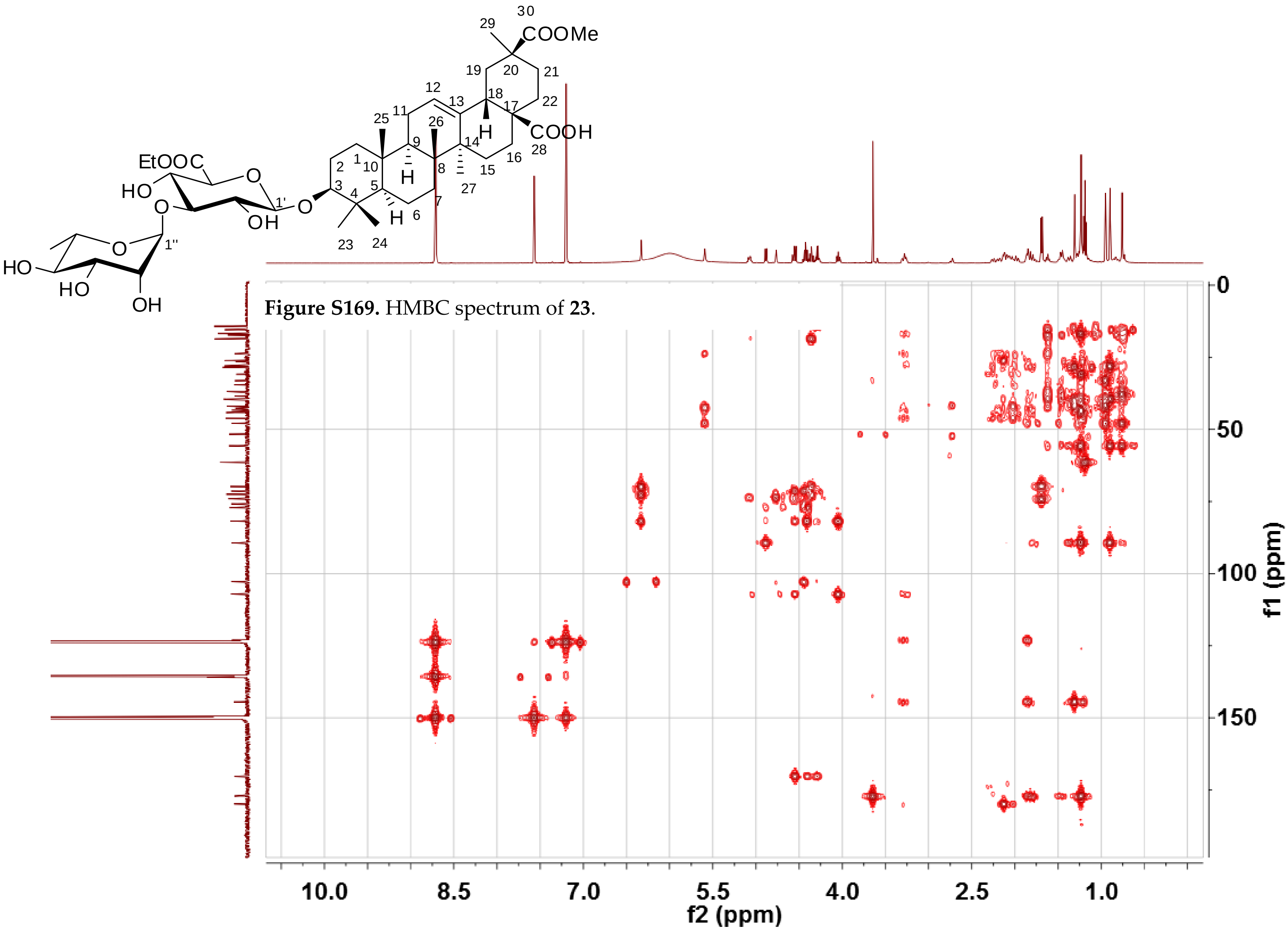

Figure S169. HMBC spectrum of **23**.

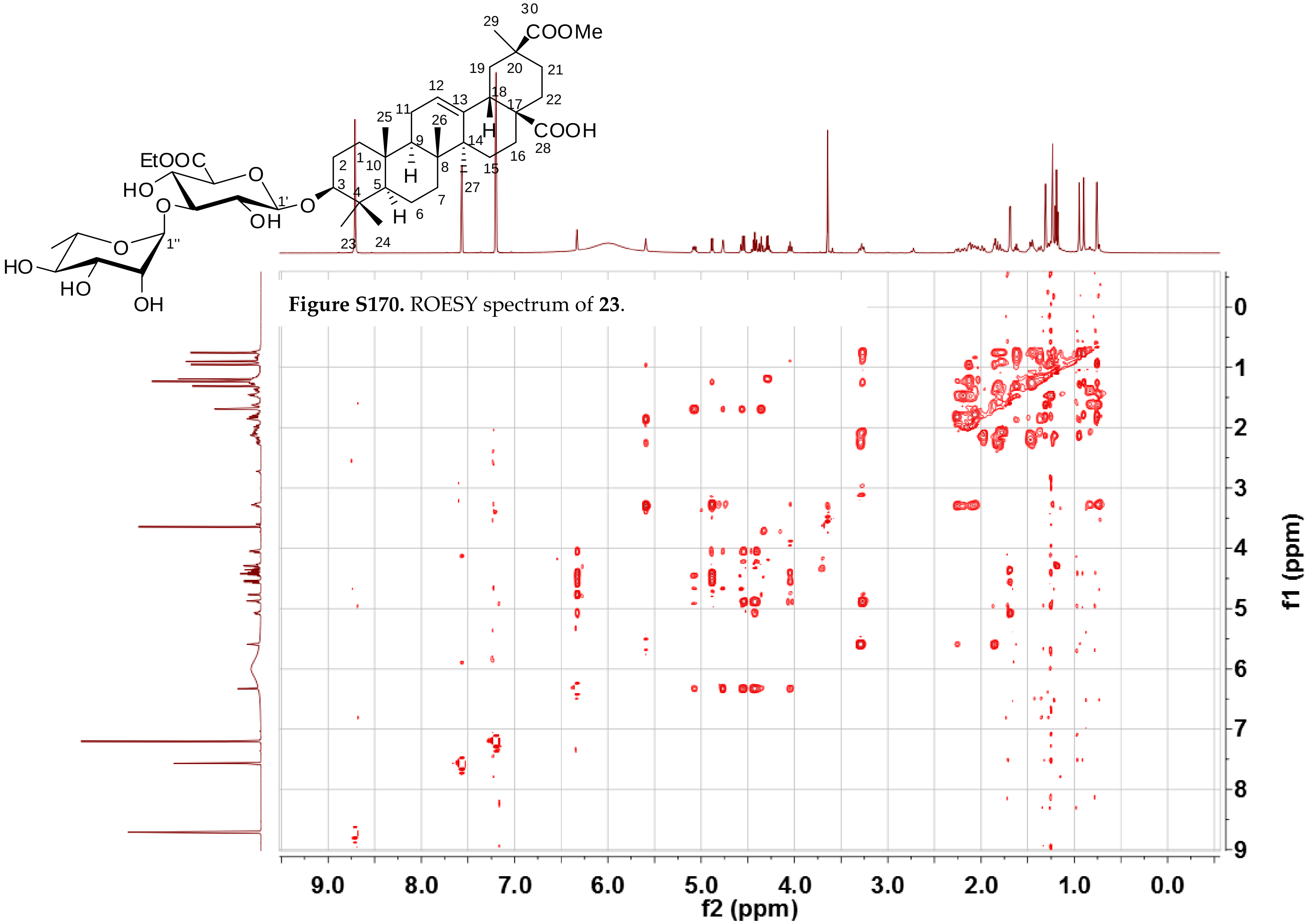

Figure S170. ROESY spectrum of **23**.

Figure S171. HRESIMS spectrum of **23**.

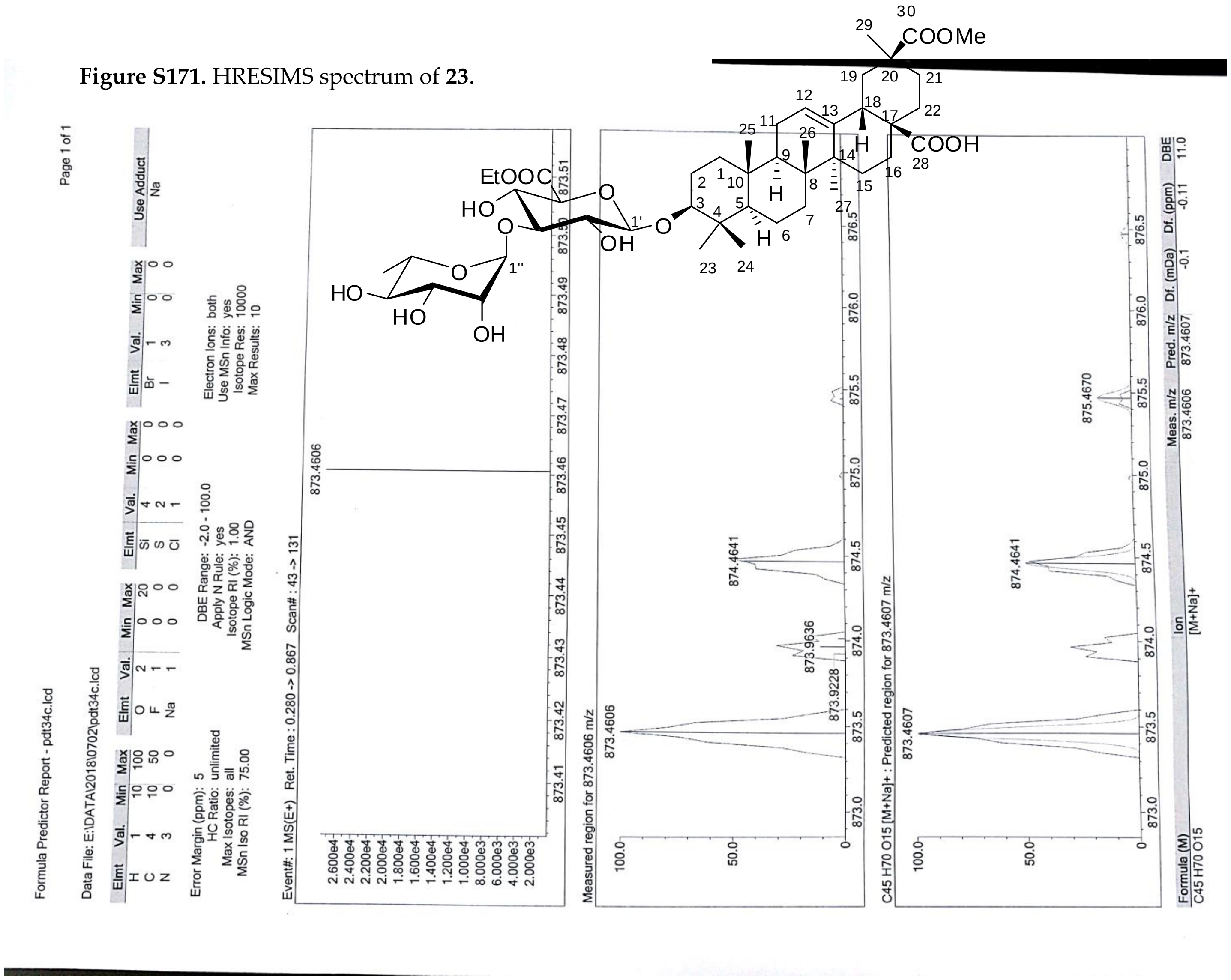

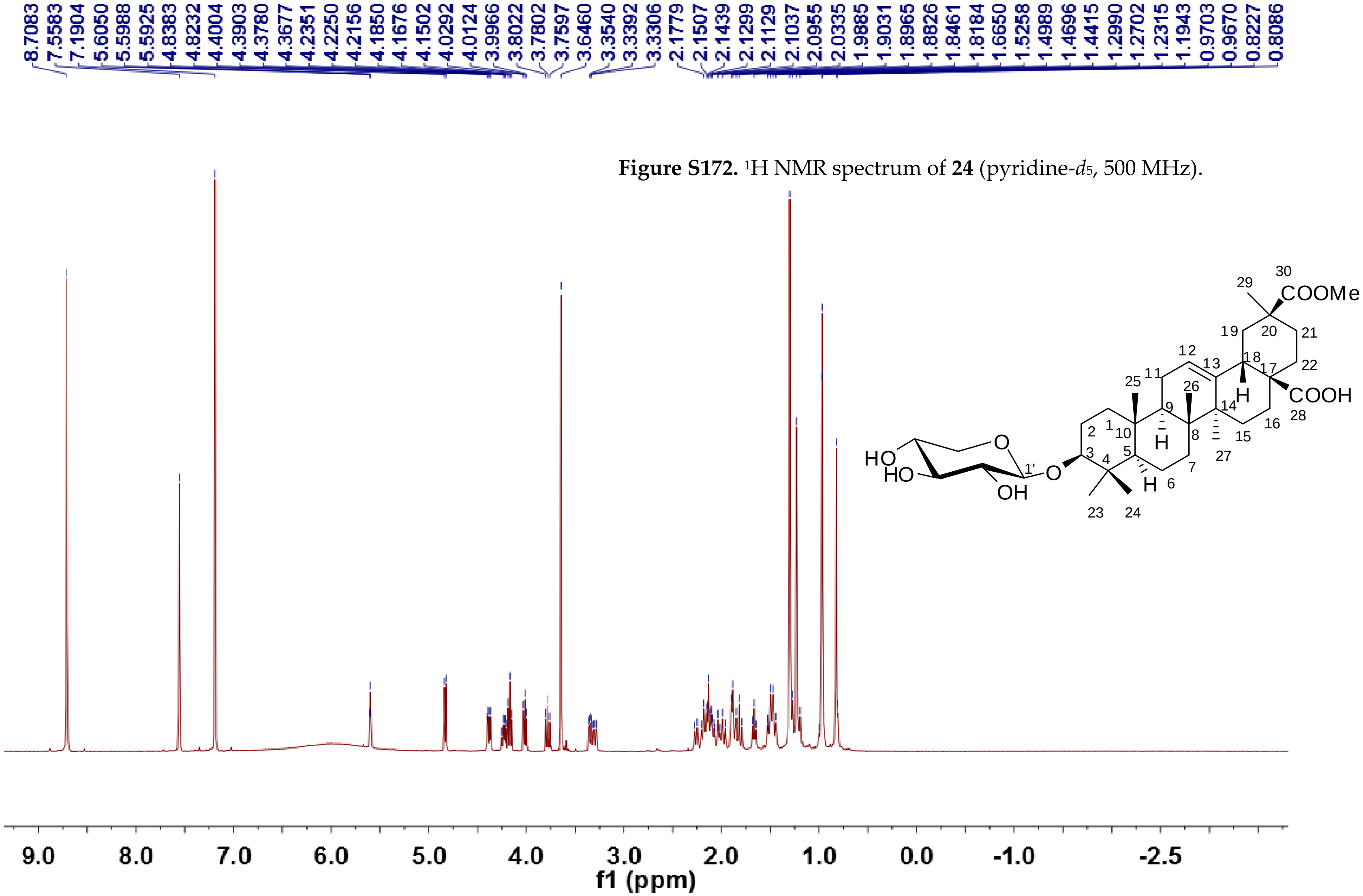

Figure S172. ^{1}H NMR spectrum of **24** (pyridine-*d*5, 500 MHz).

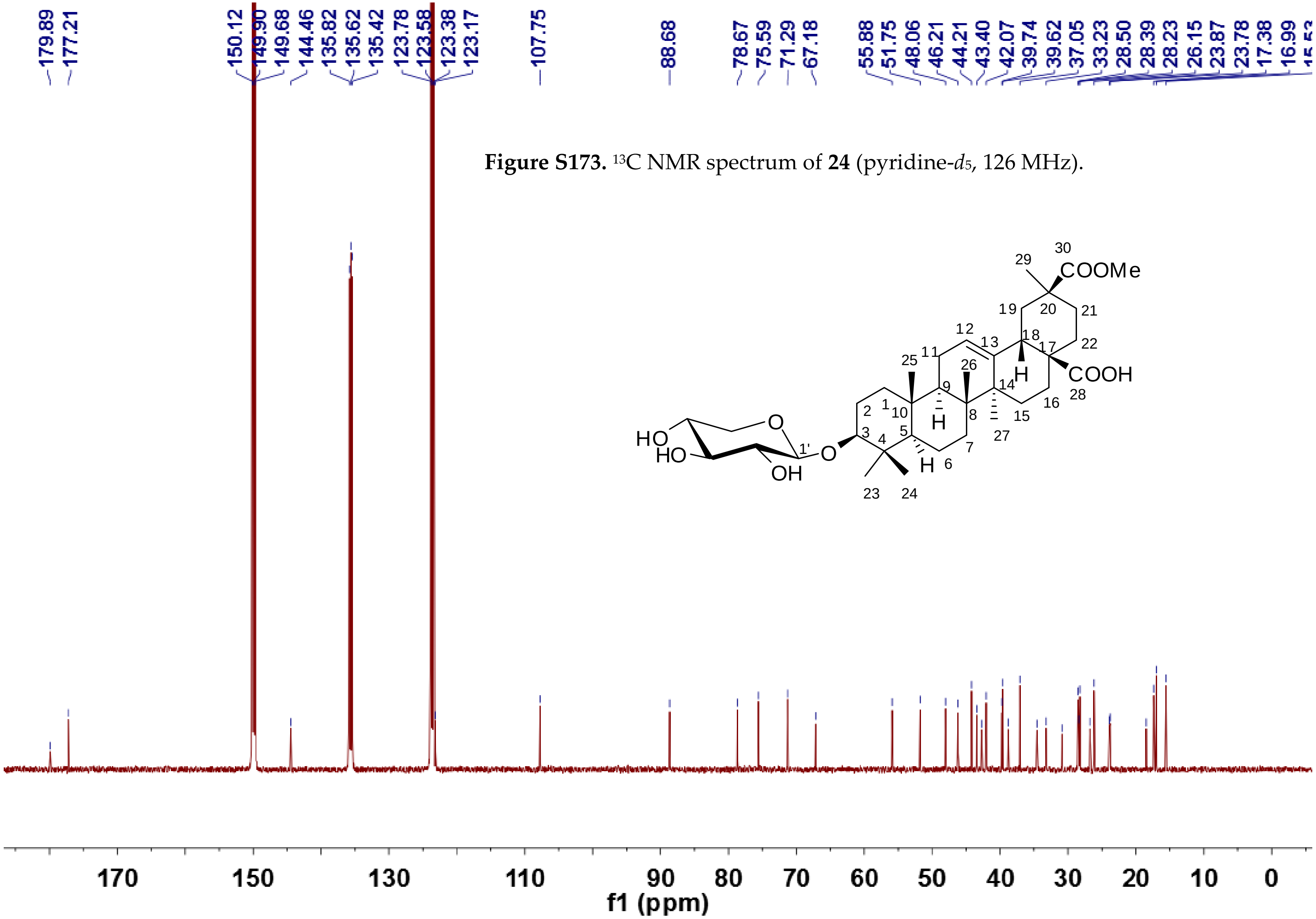

Figure S173. ^{13}C NMR spectrum of **24** (pyridine-d_5, 126 MHz).

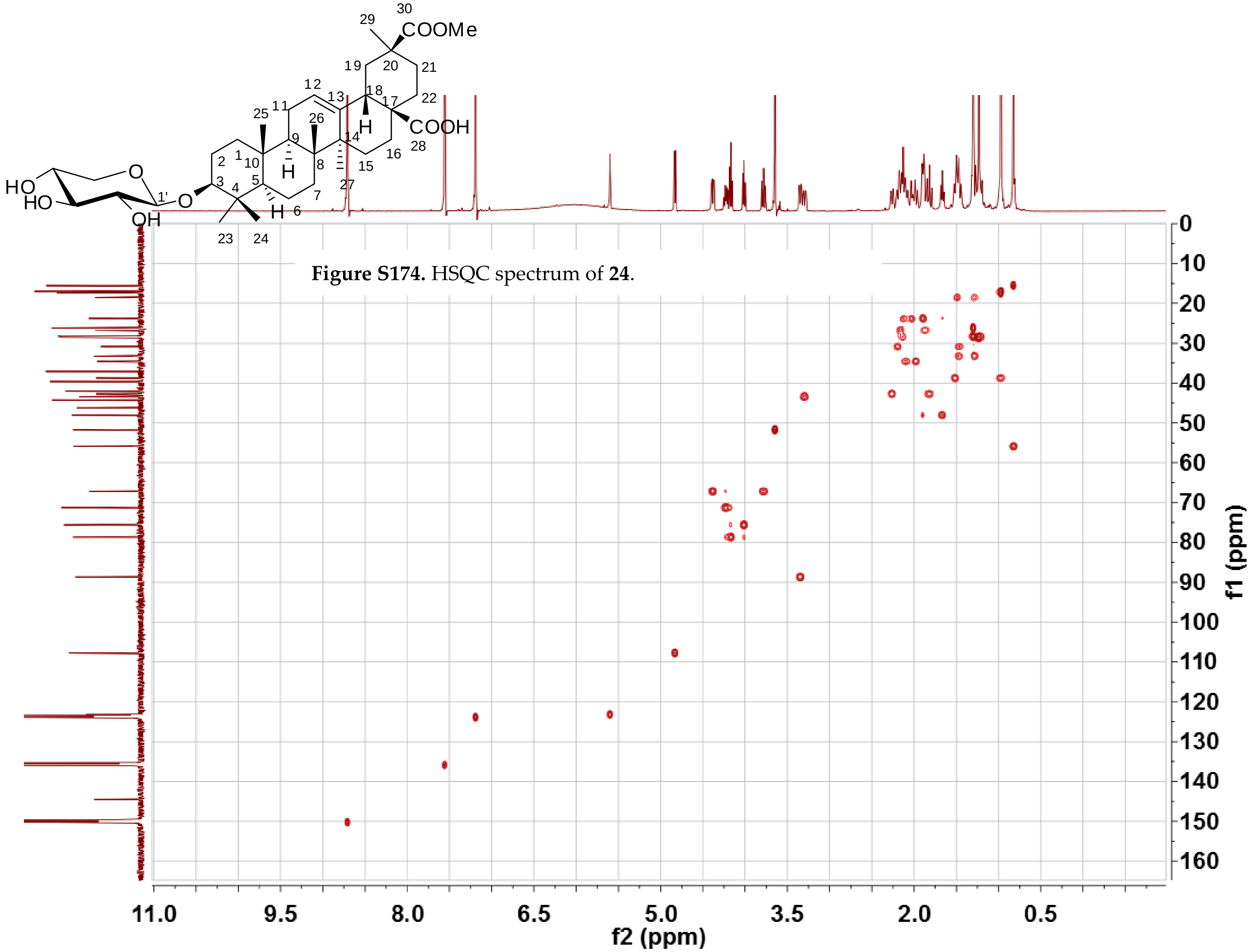

Figure S174. HSQC spectrum of **24**.

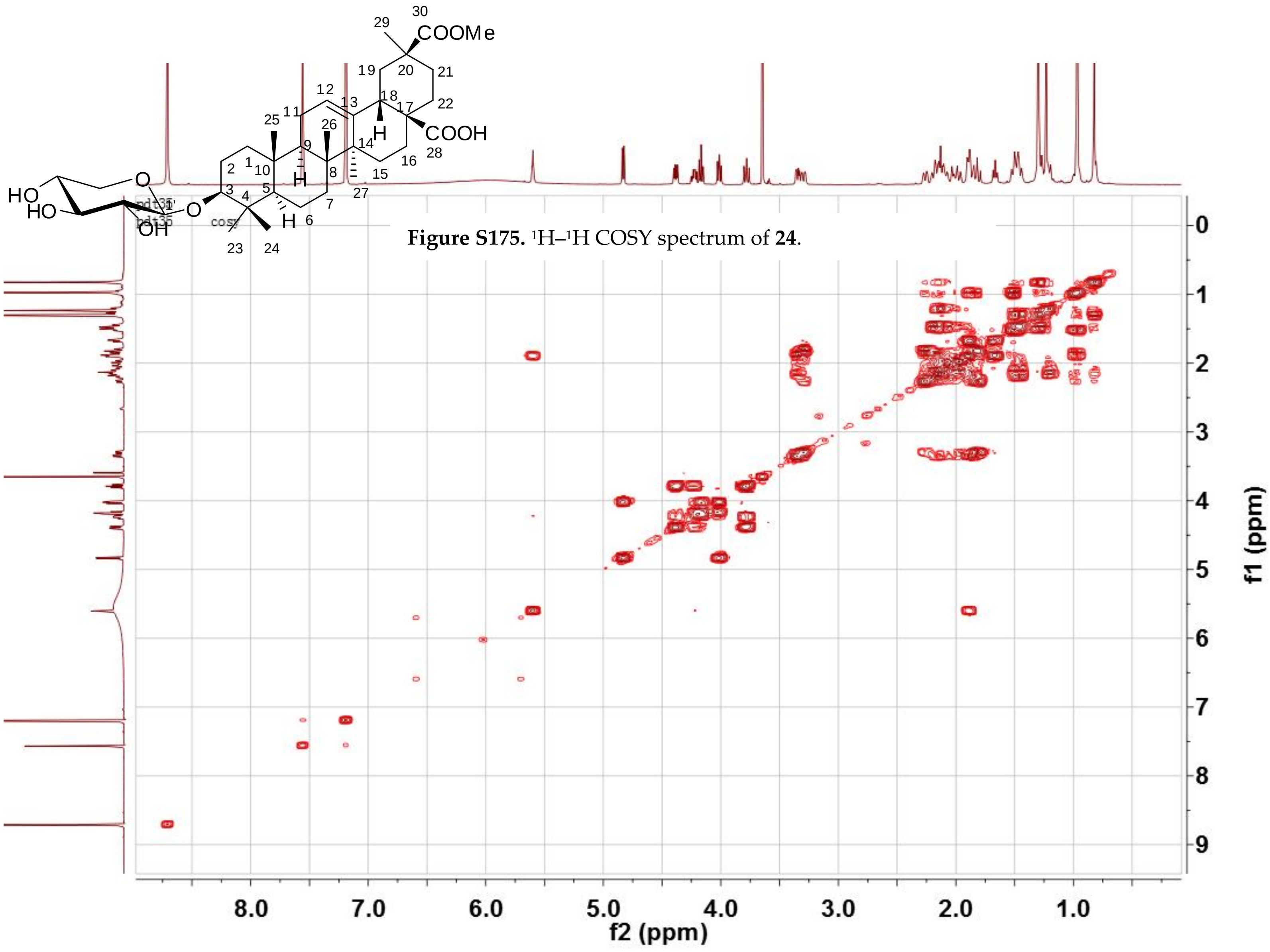

Figure S175. ^{1}H–^{1}H COSY spectrum of **24**.

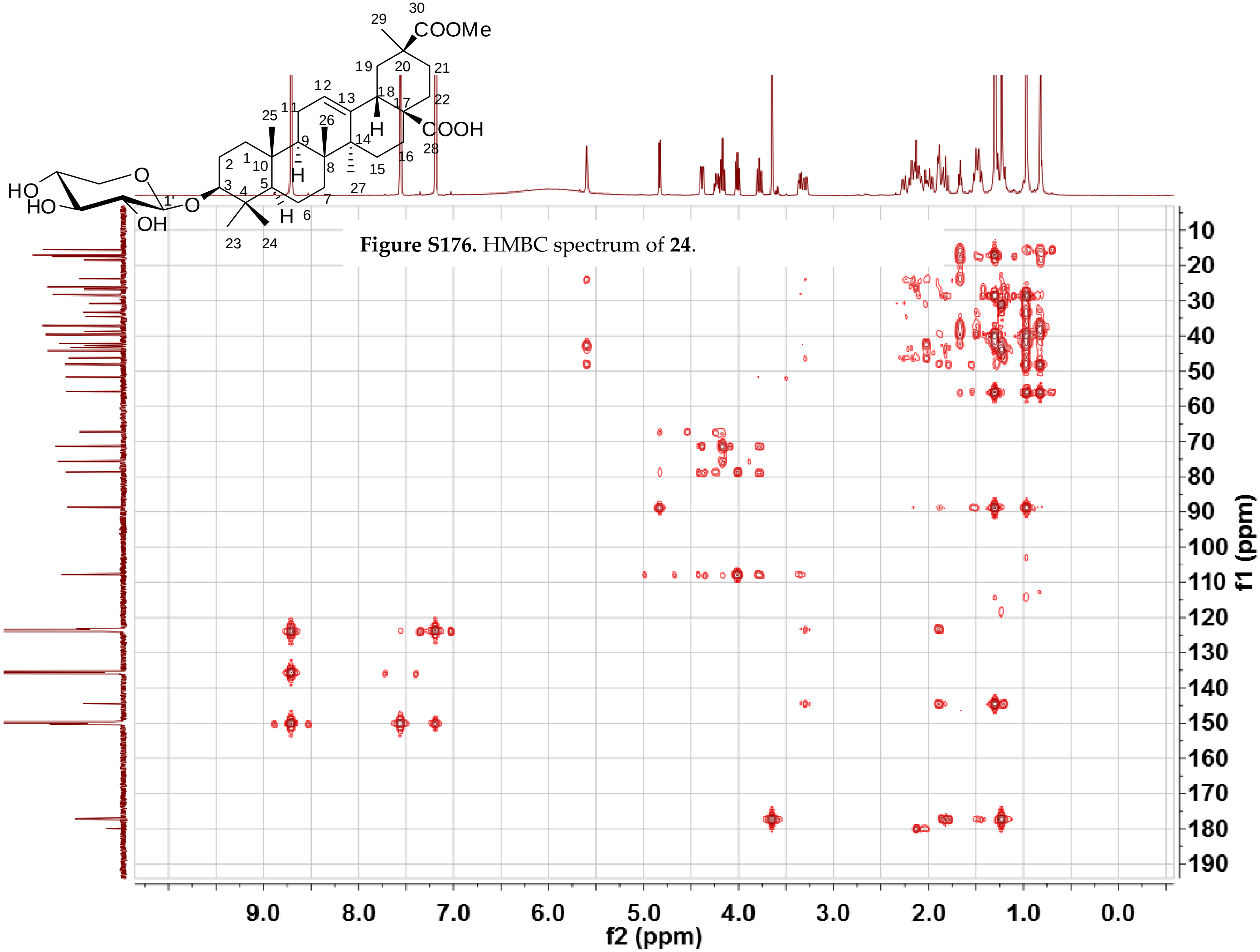

Figure S176. HMBC spectrum of **24**.

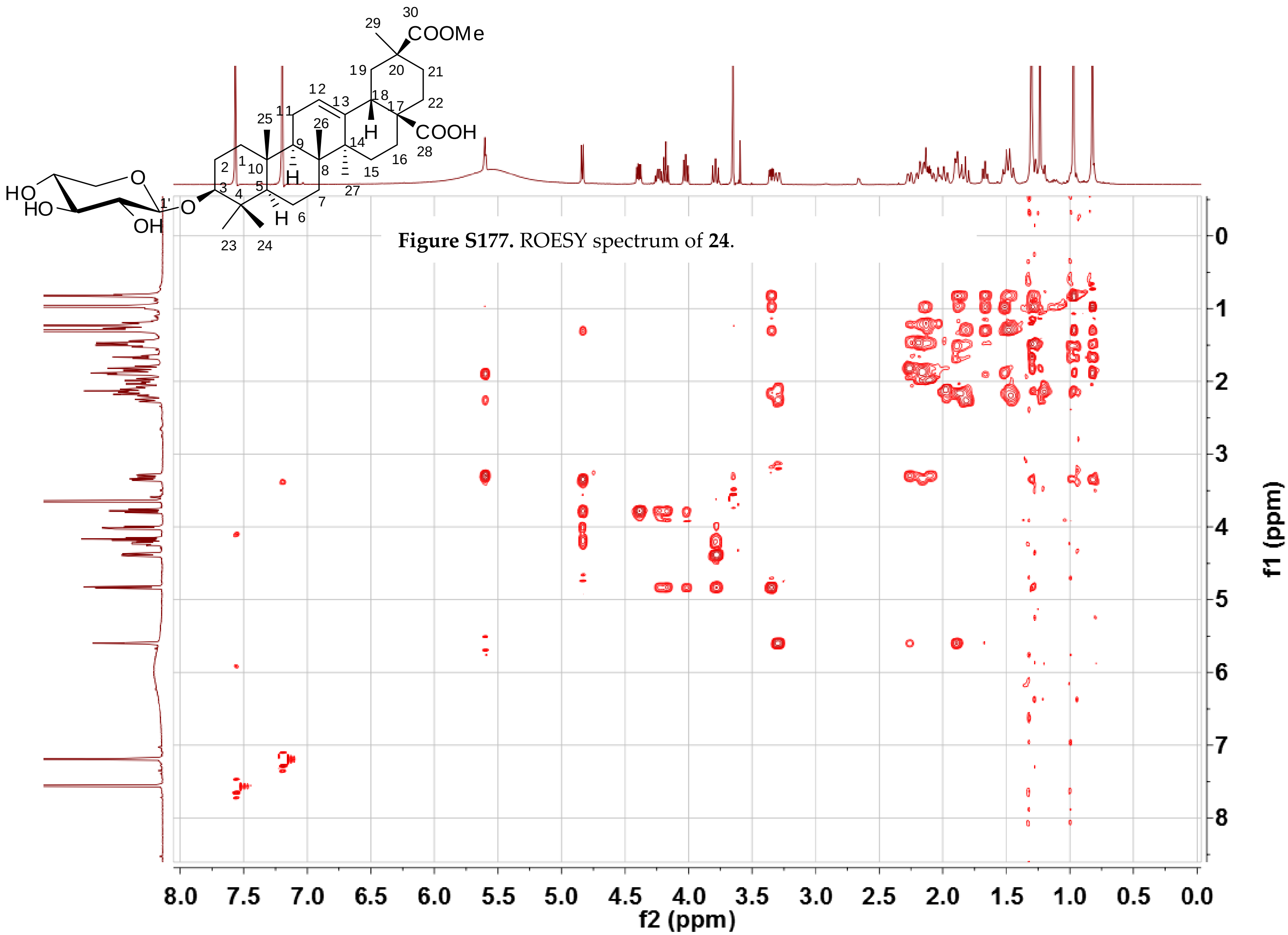

Figure S177. ROESY spectrum of **24**.

**Data Filename**	190107ESIA6.d	**Sample Name**	pdt35
**Sample Type**	Sample	**Position**	
**Instrument Name**	Agilent G6230 TOF MS	**User Name**	KIB
**Acq Method**	ESI.m	**Acquired Time**	1/7/2019 3:11:02 PM
**IRM Calibration Status**	Success	**DA Method**	ESI.m
**Comment**			

**Sample Group**		**Info.**
**Acquisition SW Version**	6200 series TOF/6500 series Q-TOF B.05.01 (B5125.2)	

User Spectra

Fragmentor Voltage	Collision Energy	Ionization Mode
320	0	ESI

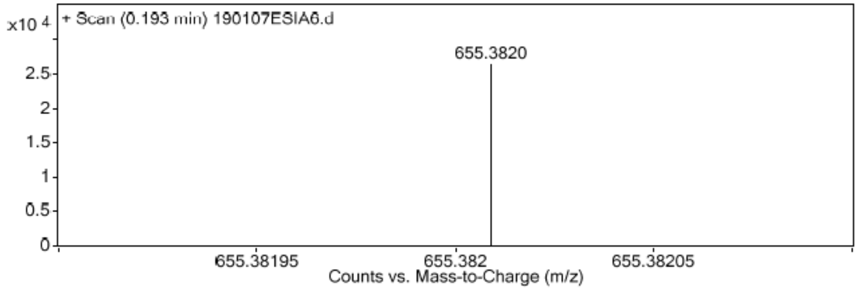

Peak List

m/z	z	Abund	Formula	Ion
106.0392	1	12415.93		
122.5483	2	27243.45		
166.0629	1	26196.08		
182.0404	1	78880.84		
194.0606	1	14238.85		
655.382	1	26472.7	C36 H56 Na O9	M+
671.3535	1	13972.36		
696.4058	1	16606.49		
826.3661	1	11042.4		
1287.7718	1	11313.11		

Formula Calculator Element Limits

Element	Min	Max
C	0	200
H	0	400
O	5	12
Na	1	1

Formula Calculator Results

Formula	CalculatedMass	Mz	Diff.(mDa)	Diff. (ppm)	DBE
C36 H56 Na O9	655.3822	655.3820	0.2	0.3	8.5

--- End Of Report ---

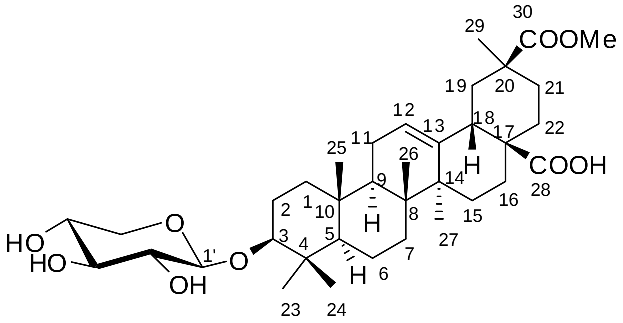

Figure S178. HRESIMS spectrum of **24**.

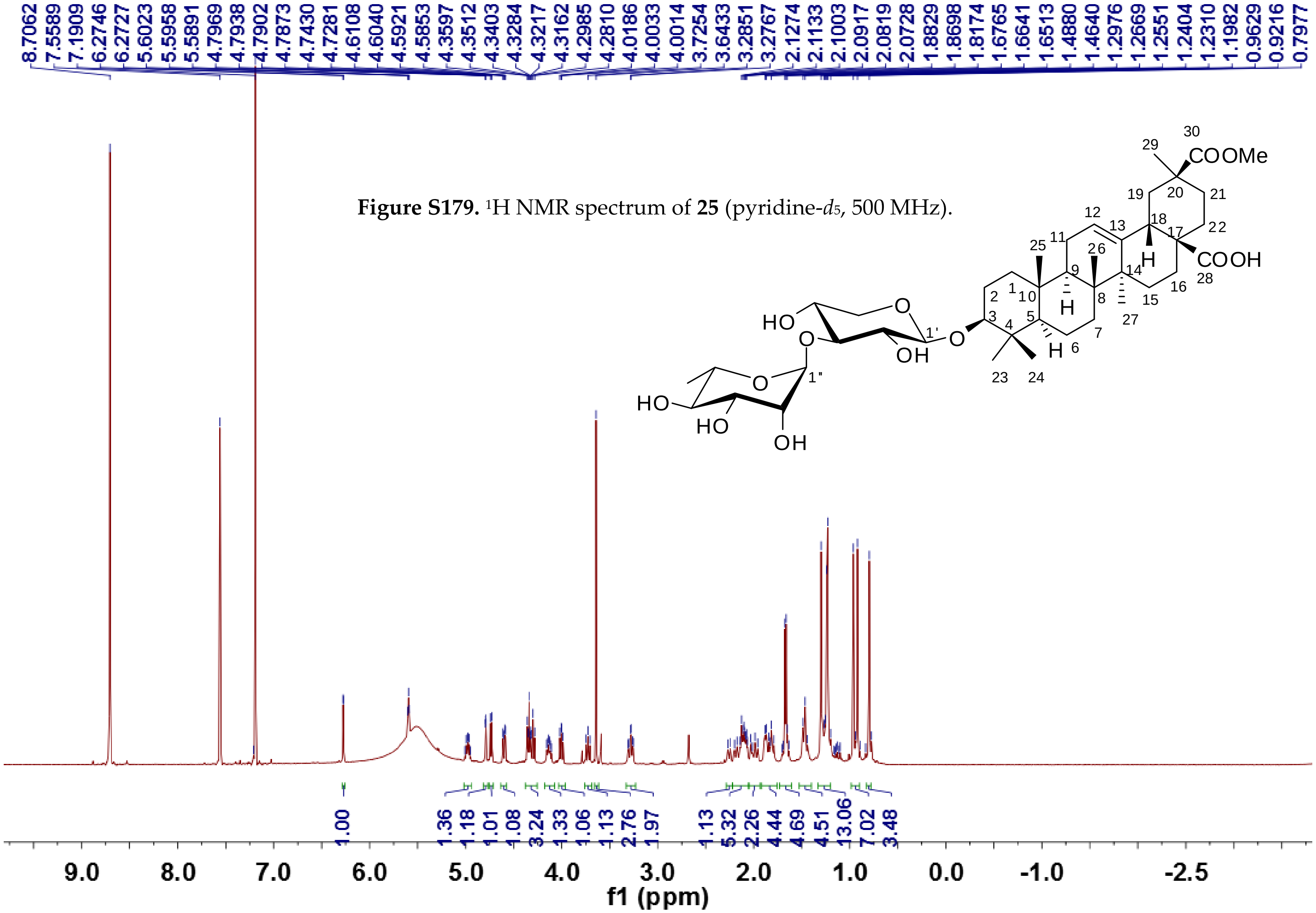

Figure S179. ¹H NMR spectrum of **25** (pyridine-d_5, 500 MHz).

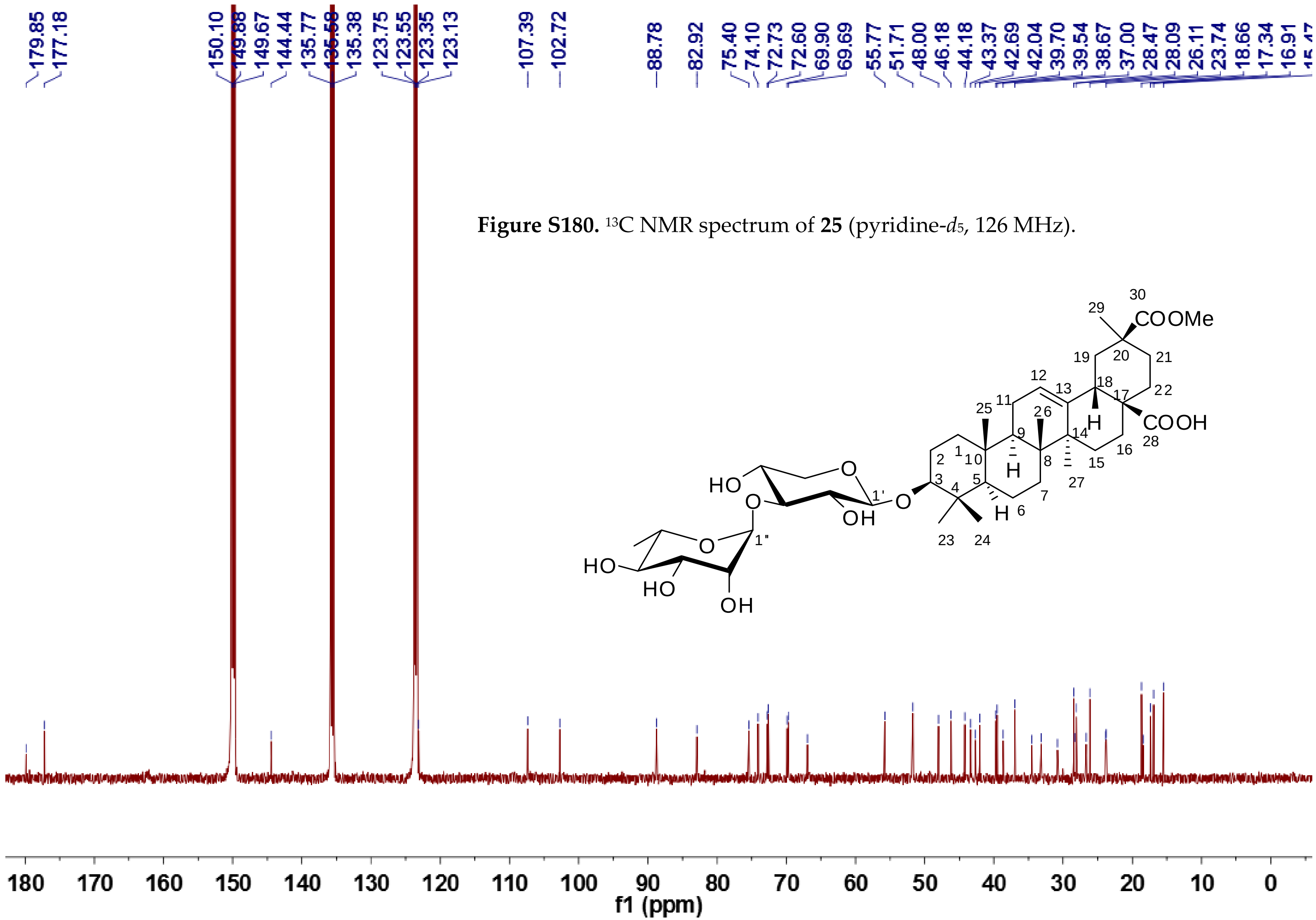

Figure S180. ^{13}C NMR spectrum of **25** (pyridine-*d*$_5$, 126 MHz).

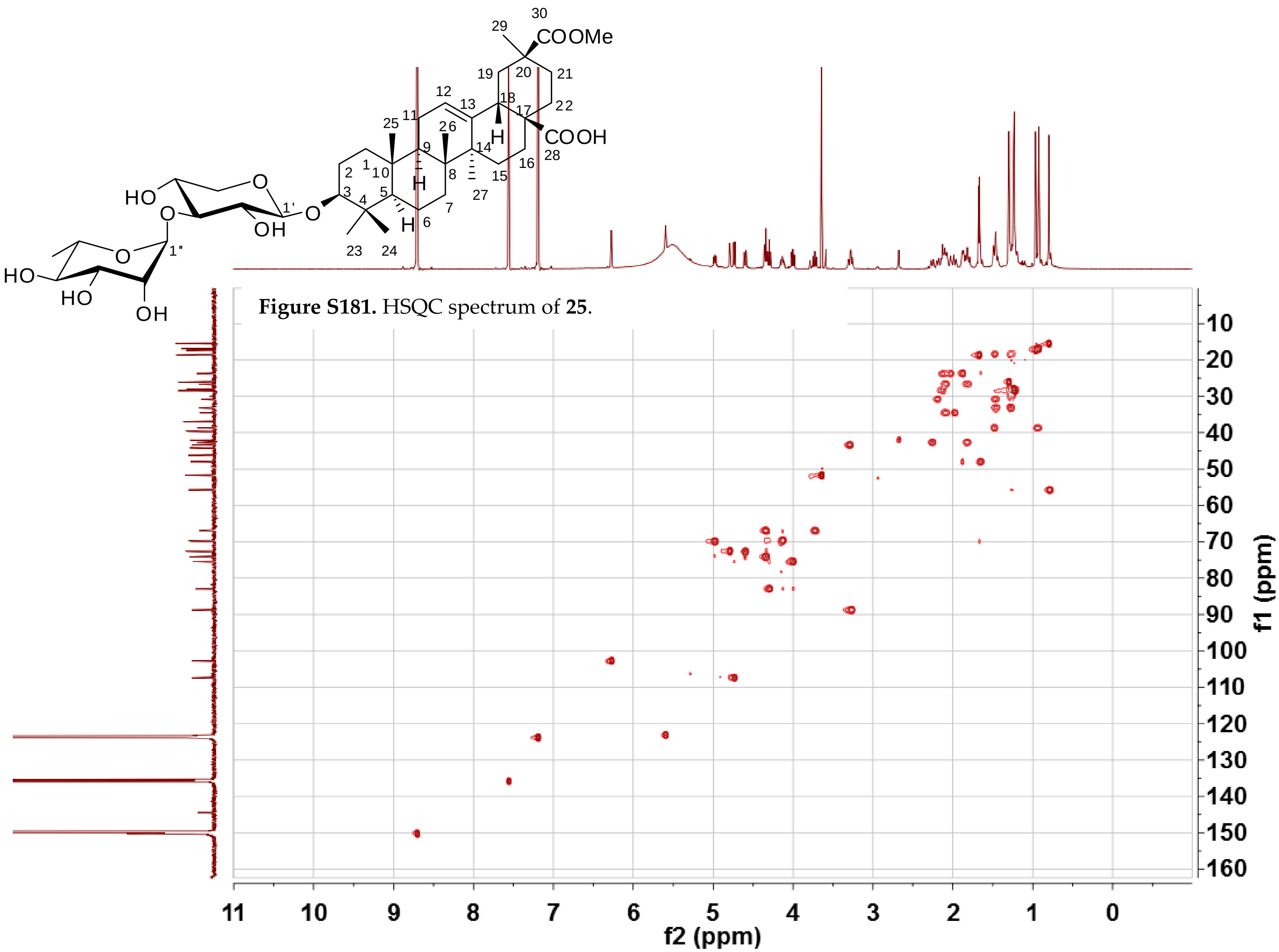

Figure S181. HSQC spectrum of **25**.

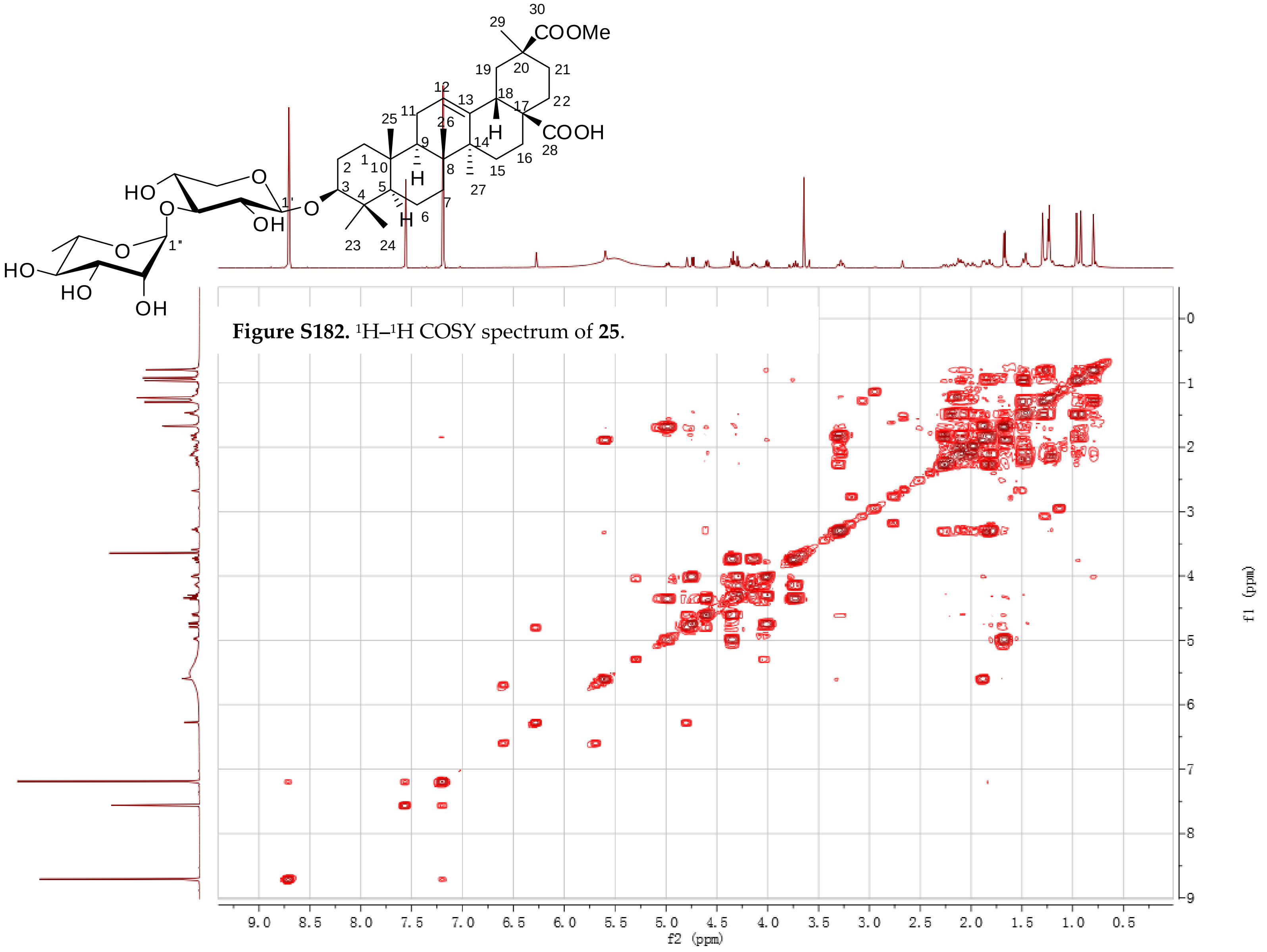

Figure S182. ^{1}H–^{1}H COSY spectrum of **25**.

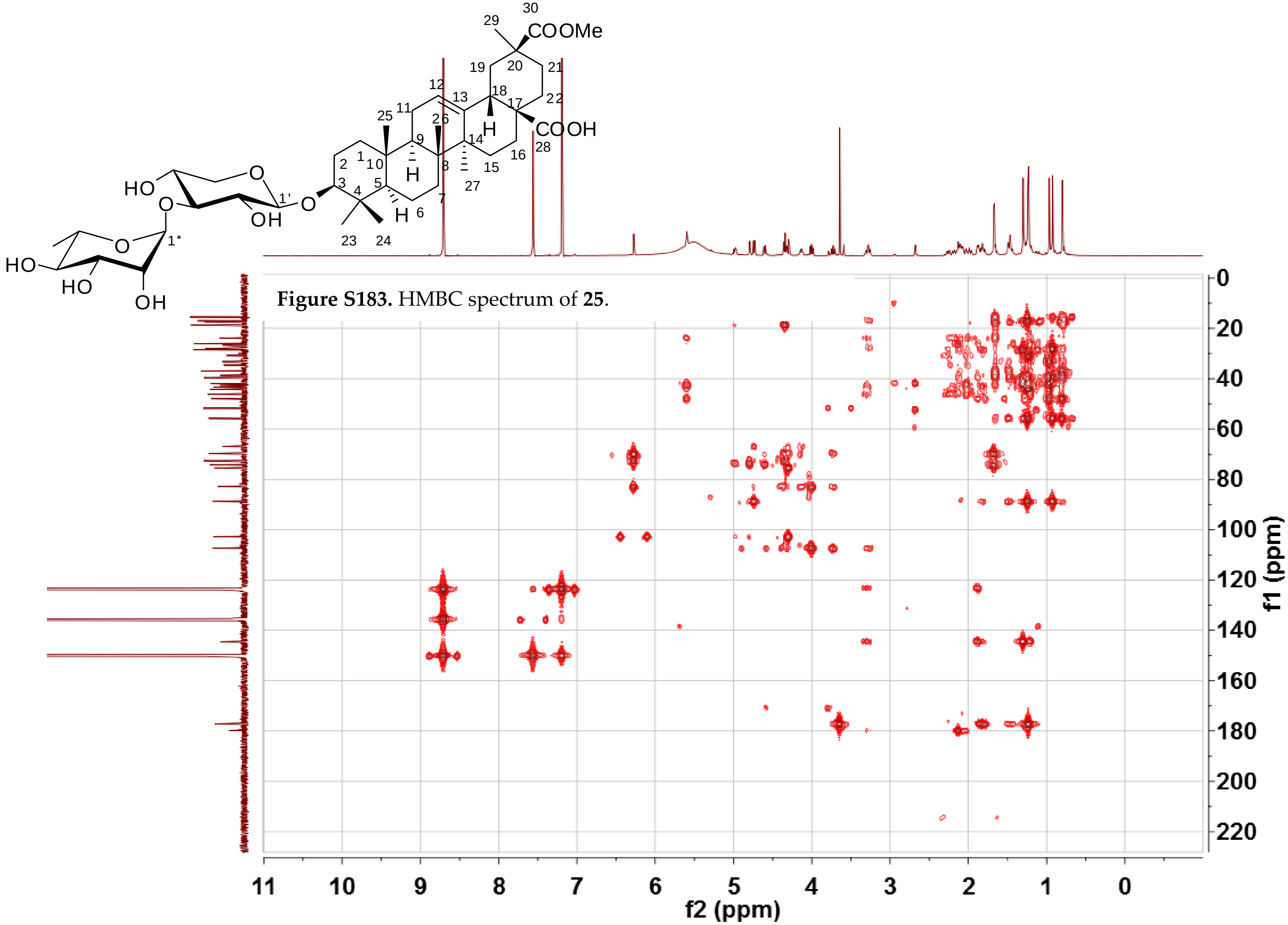

Figure S183. HMBC spectrum of **25**.

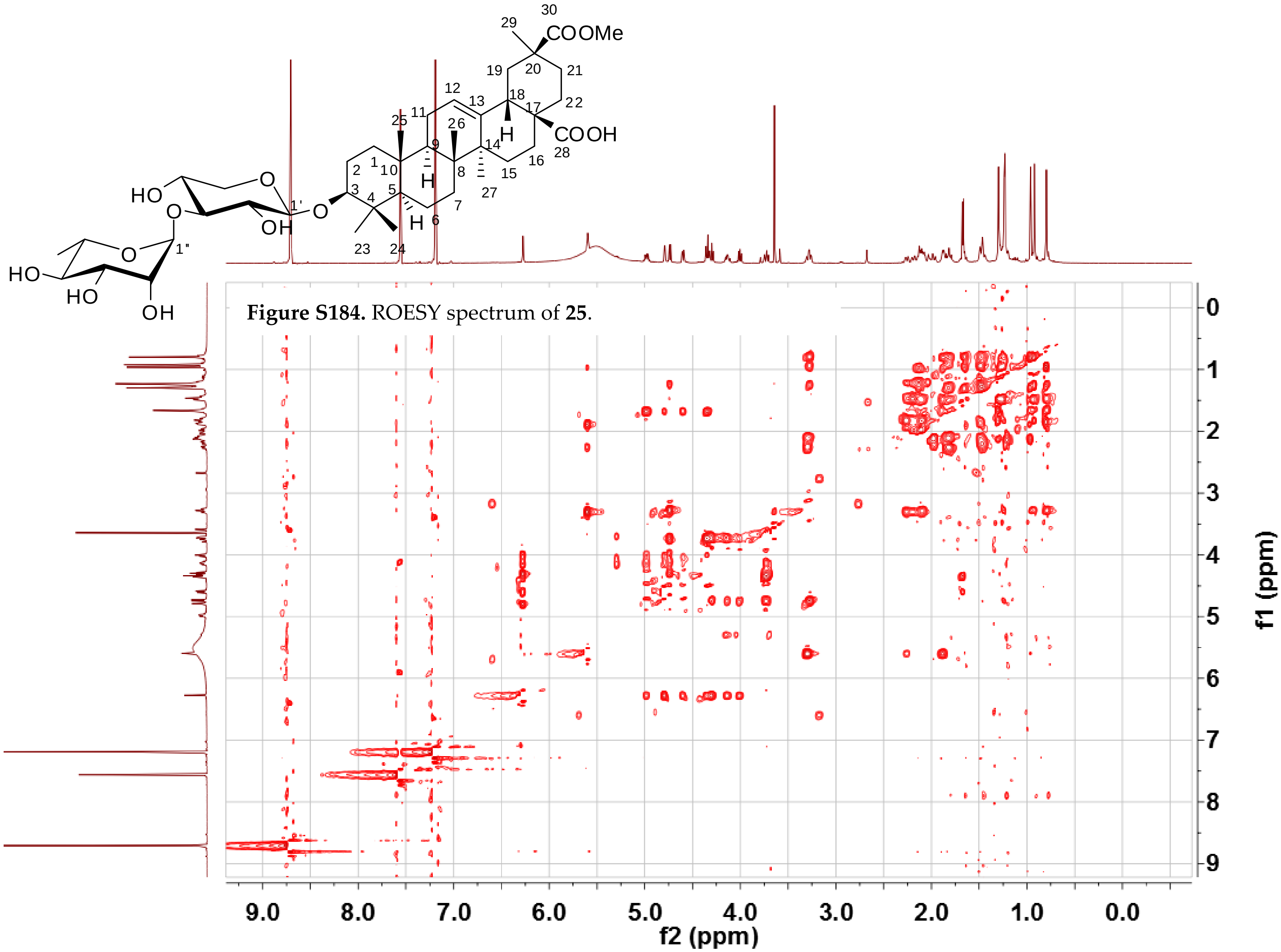

Figure S184. ROESY spectrum of **25**.

Figure S185. HRESIMS spectrum of **25**.

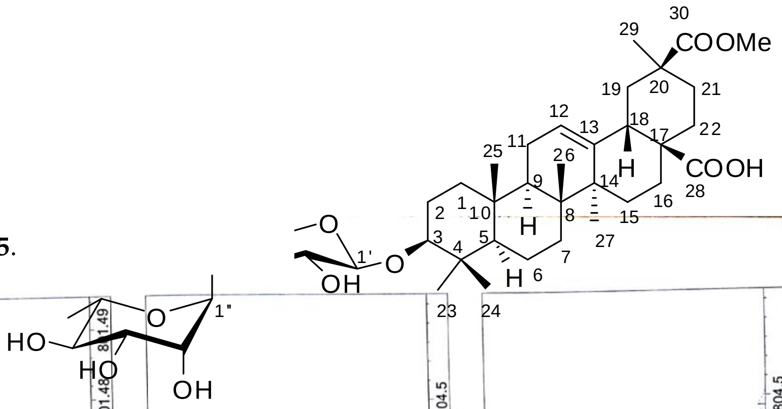

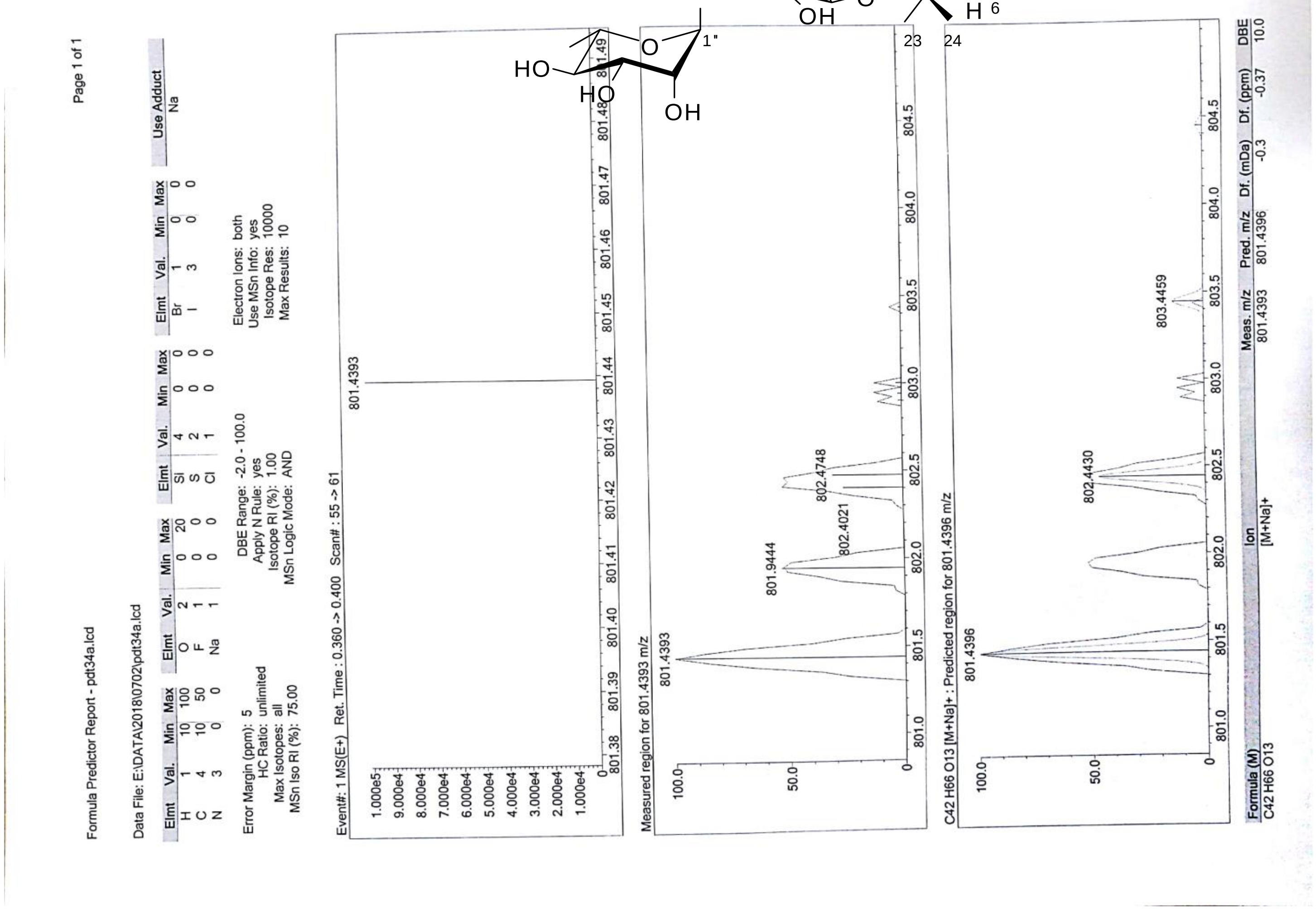

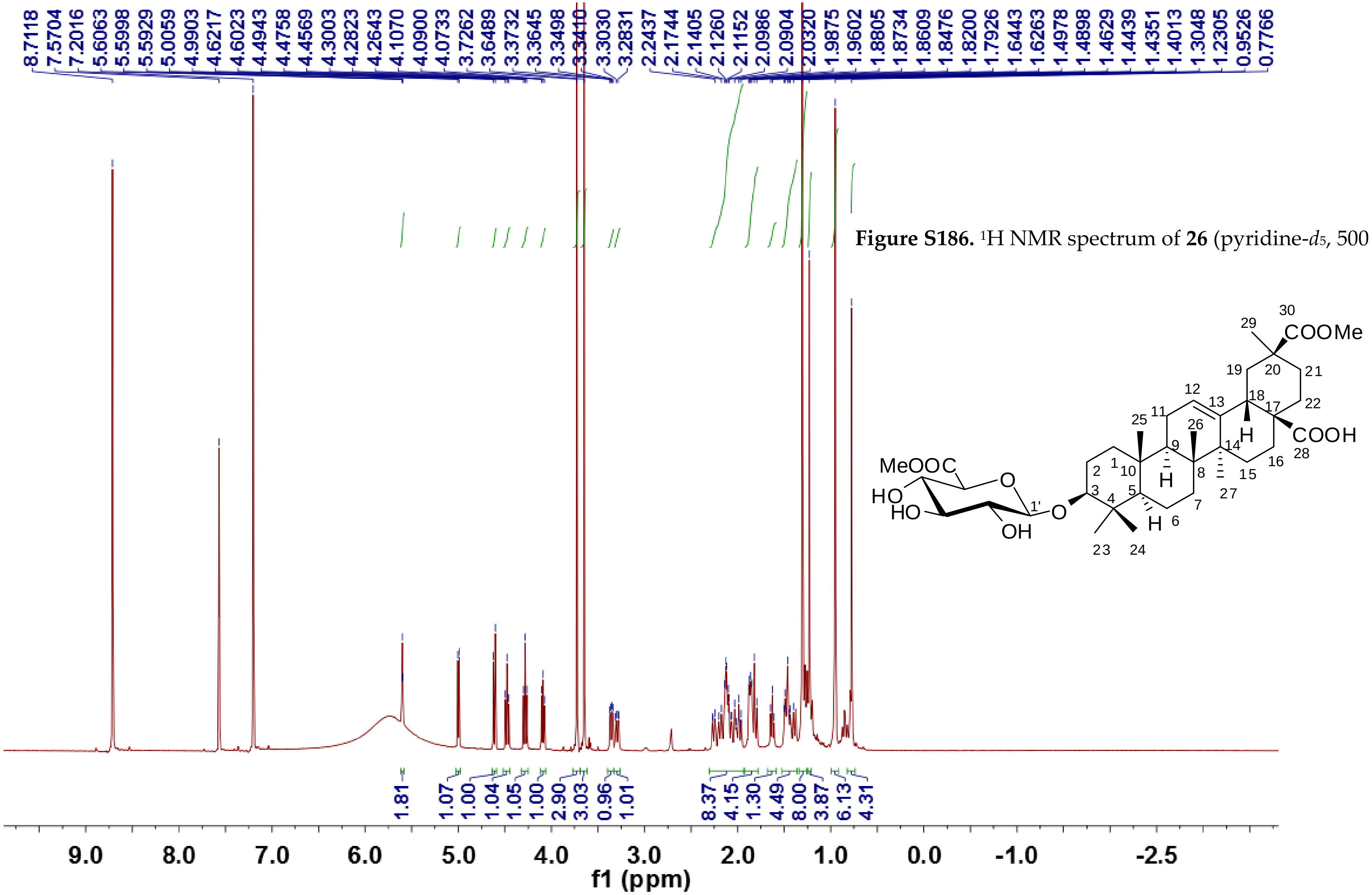

Figure S186. ^{1}H NMR spectrum of **26** (pyridine-*d*5, 500 MHz).

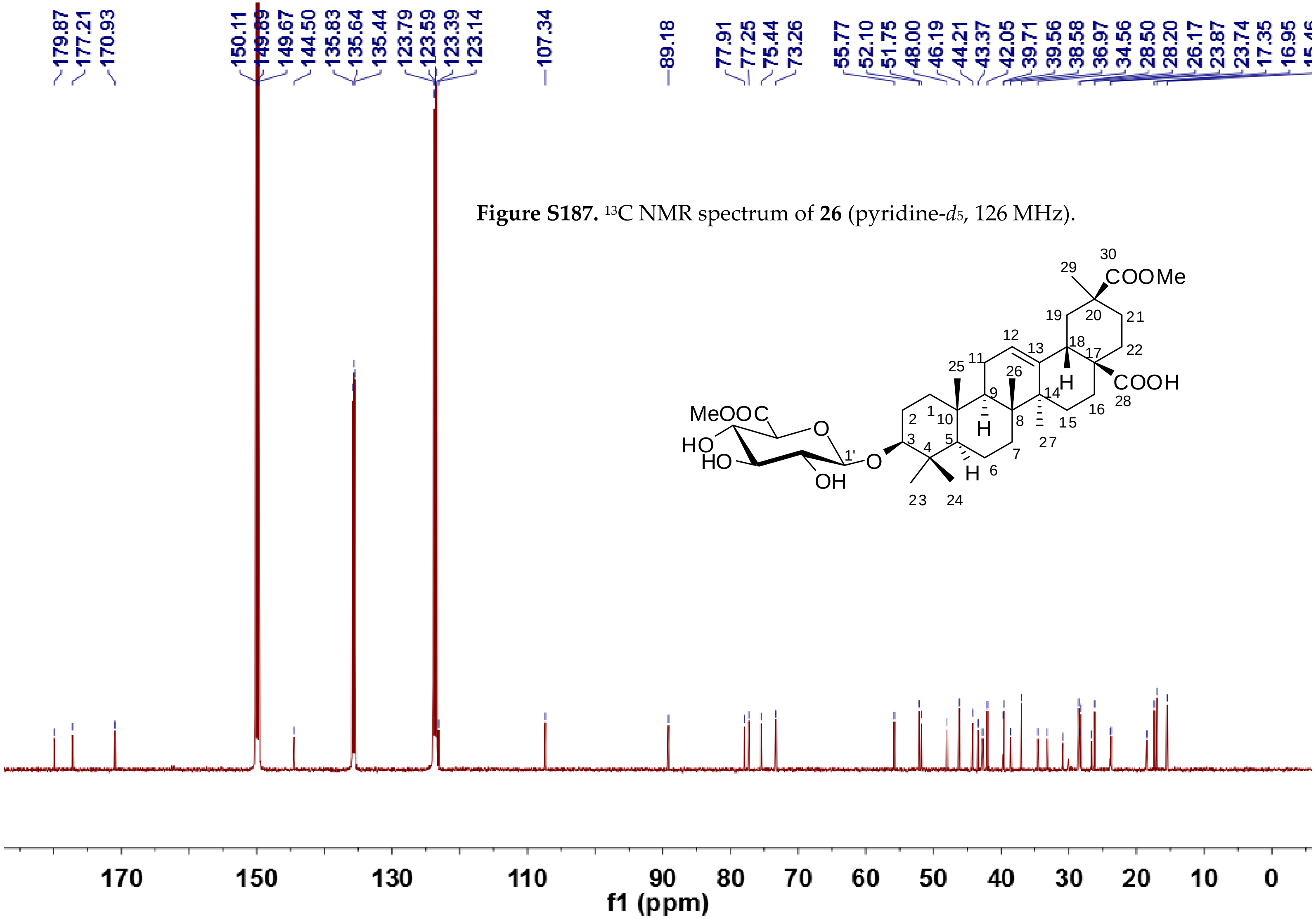

Figure S187. ^{13}C NMR spectrum of **26** (pyridine-d_5, 126 MHz).

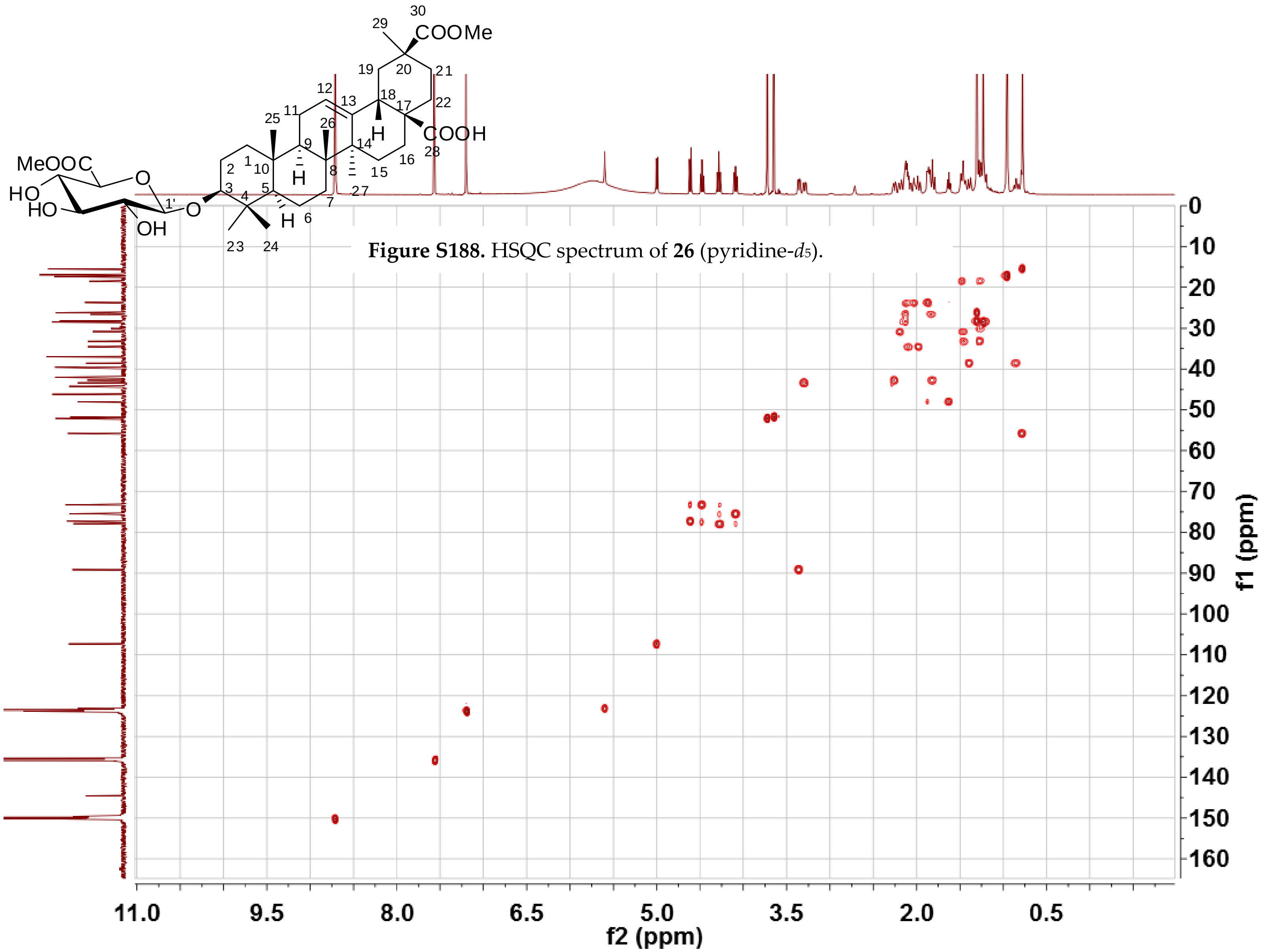

Figure S188. HSQC spectrum of **26** (pyridine-*d*5).

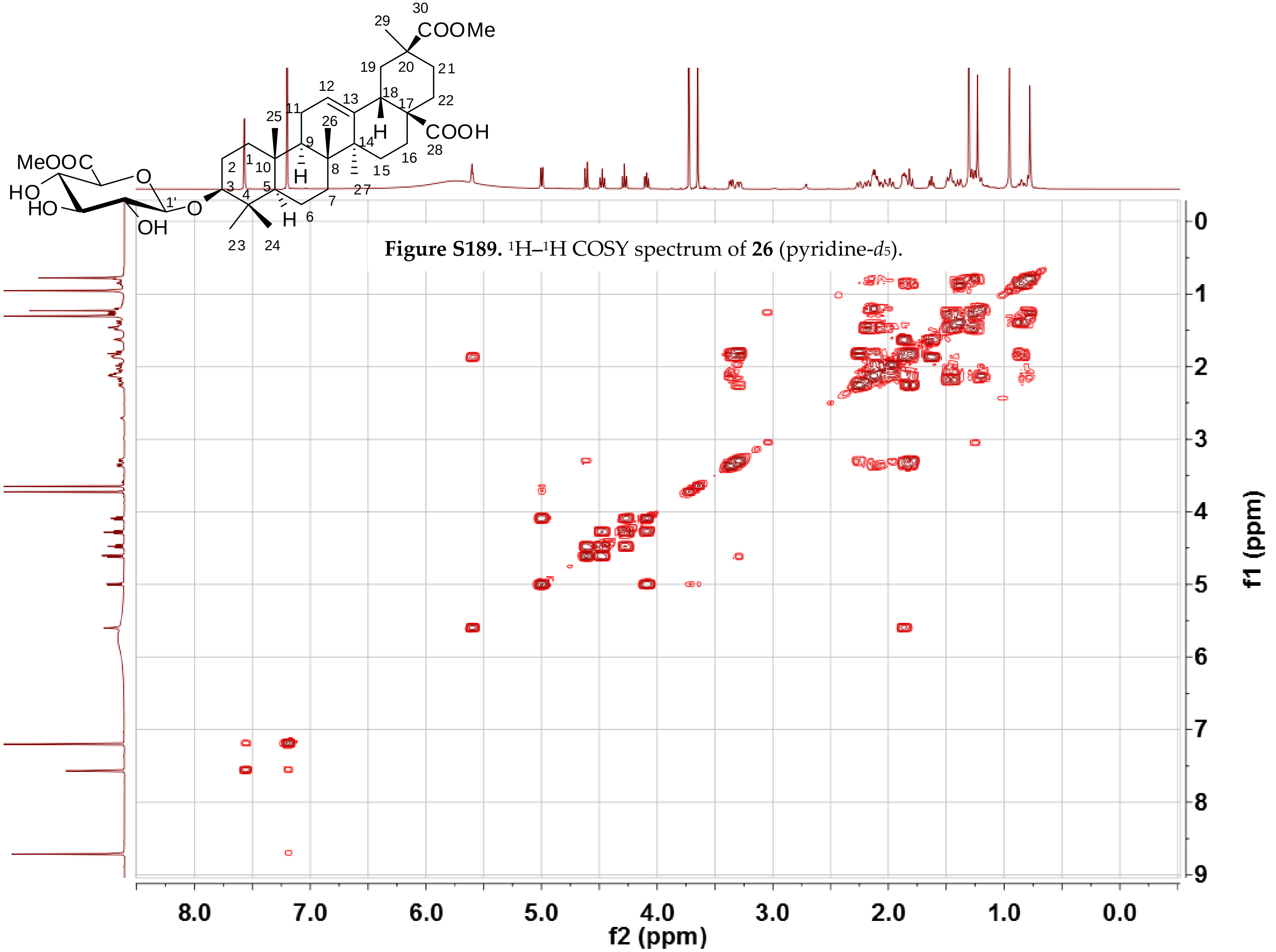

Figure S189. ^{1}H–^{1}H COSY spectrum of **26** (pyridine-*d*5).

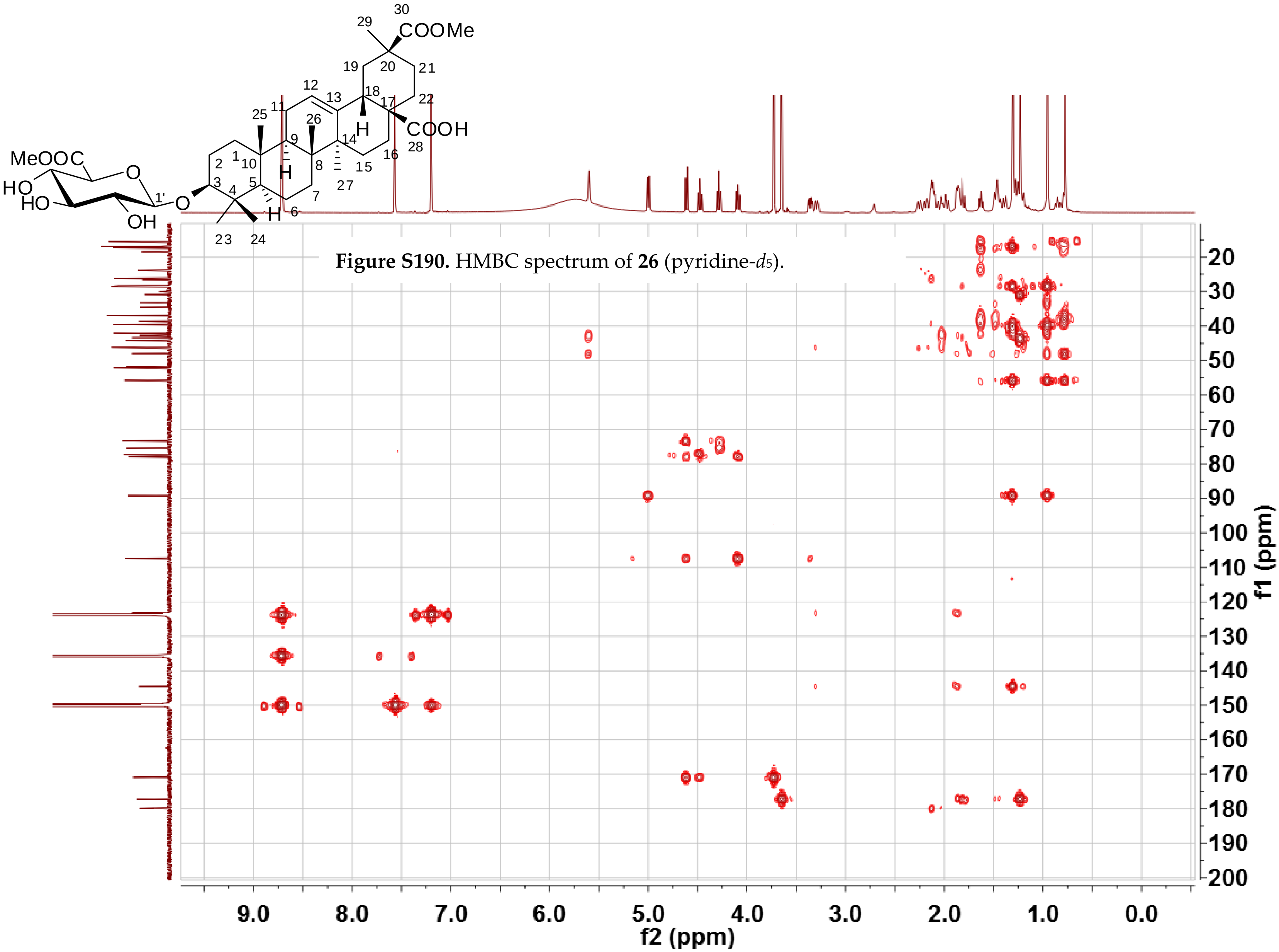

Figure S190. HMBC spectrum of **26** (pyridine-d_5).

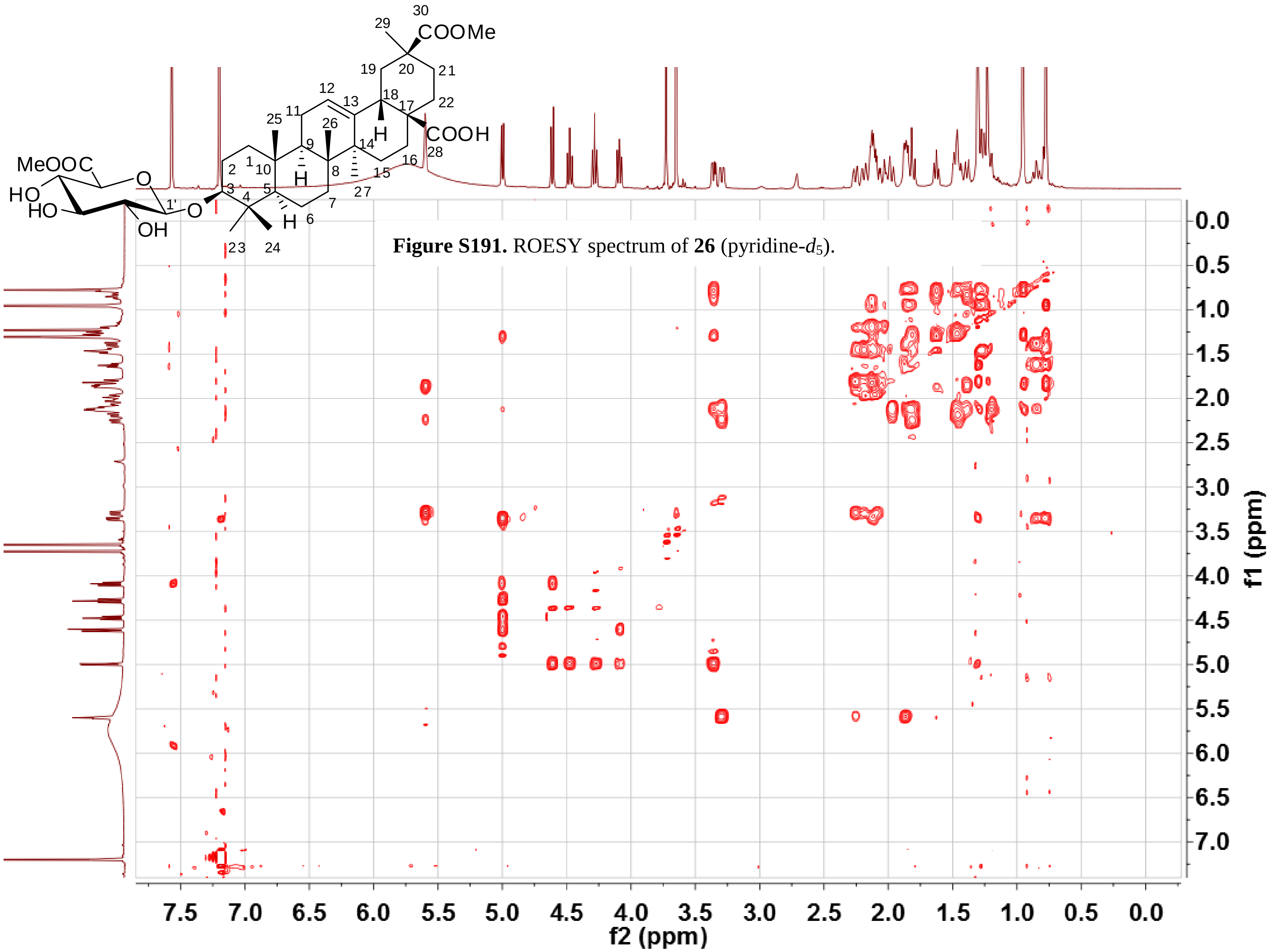

Figure S191. ROESY spectrum of **26** (pyridine-d_5).

**Data Filename**	190107ESIA7.d	**Sample Name**	pdt36
**Sample Type**	Sample	**Position**	
**Instrument Name**	Agilent G6230 TOF MS	**User Name**	KIB
**Acq Method**	ESI.m	**Acquired Time**	1/7/2019 3:12:16 PM
**IRM Calibration Status**	Success	**DA Method**	ESI.m
**Comment**			

**Sample Group**		**Info.**
**Acquisition SW Version**	6200 series TOF/6500 series Q-TOF B.05.01 (B5125.2)	

User Spectra

Fragmentor Voltage	Collision Energy	Ionization Mode
320	0	ESI

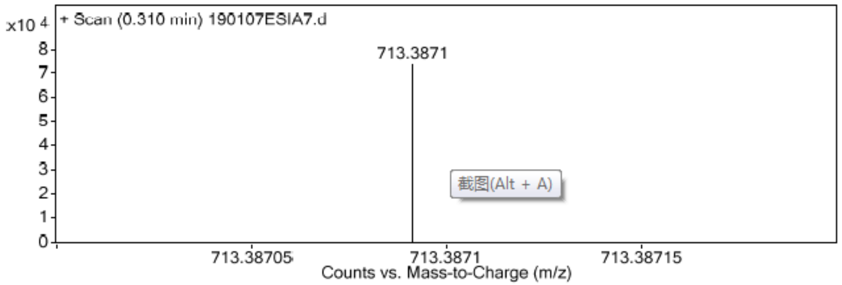

Figure S192. HRESIMS spectrum of **26**.

Peak List

m/z	z	Abund	Formula	Ion
122.5492	2	70062.16		
141.0137		49665.91		
182.0401	1	85748.97		
713.3871	1	73789.83	C38 H58 Na O11	M+
729.3569	1	105981.48		
730.36	1	43108.97		
922.0098	1	129708.32		
1403.7801	1	37441.74		
1419.7523	1	36564.46		
1420.7547	1	32141.41		

Formula Calculator Element Limits

Element	Min	Max
C	0	200
H	0	400
O	7	15
Na	1	1

Formula Calculator Results

Formula	CalculatedMass	Mz	Diff.(mDa)	Diff. (ppm)	DBE
C38 H58 Na O11	713.3877	713.3871	0.6	0.8	9.5

--- End Of Report ---

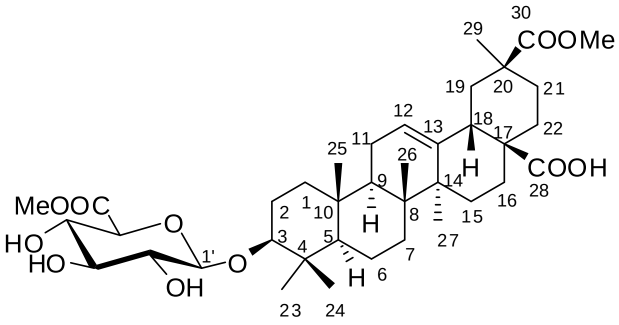

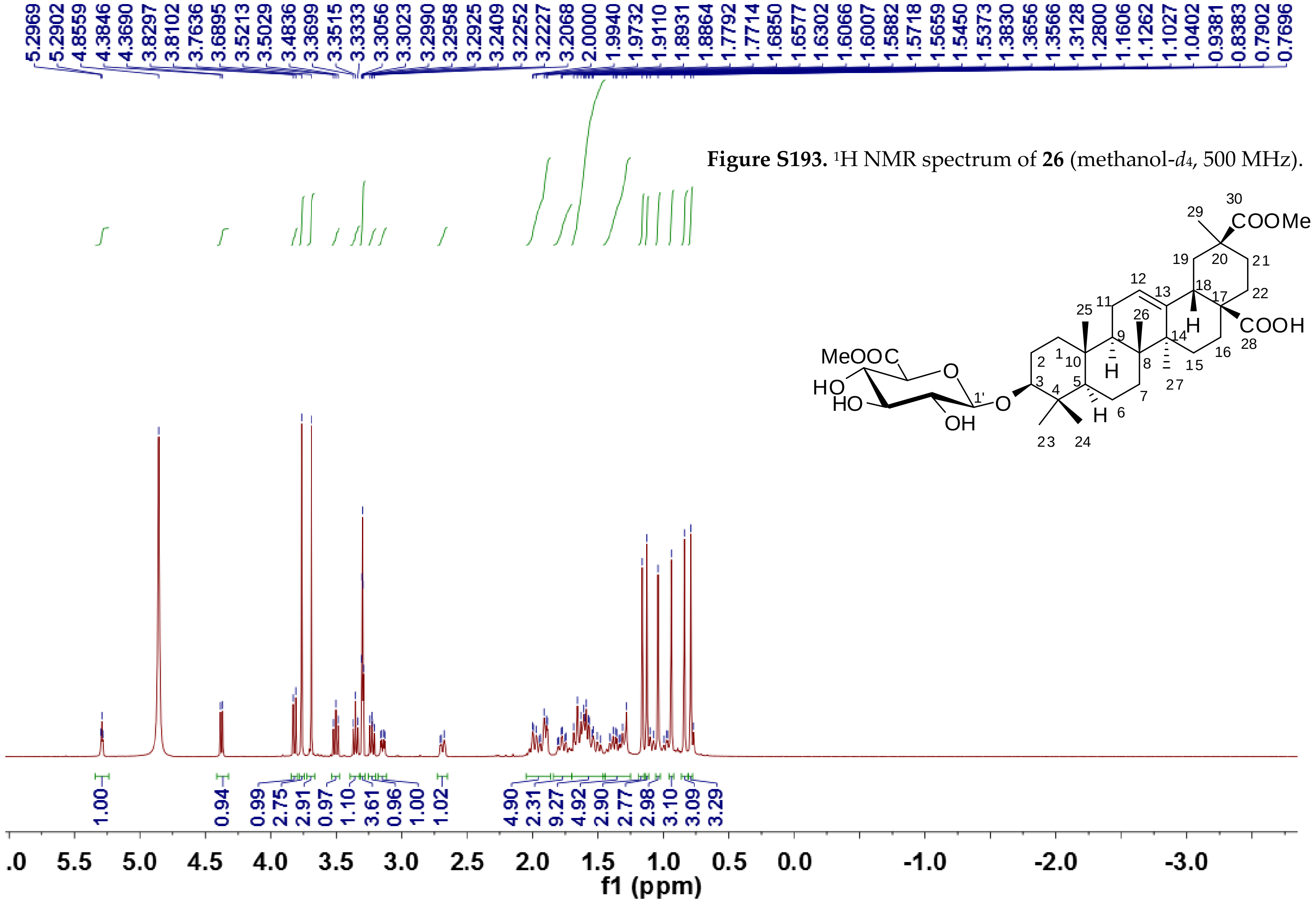

Figure S193. ^{1}H NMR spectrum of **26** (methanol-d_4, 500 MHz).

Figure S194. ^{13}C NMR spectrum of **26** (methanol-d_4, 126 MHz).

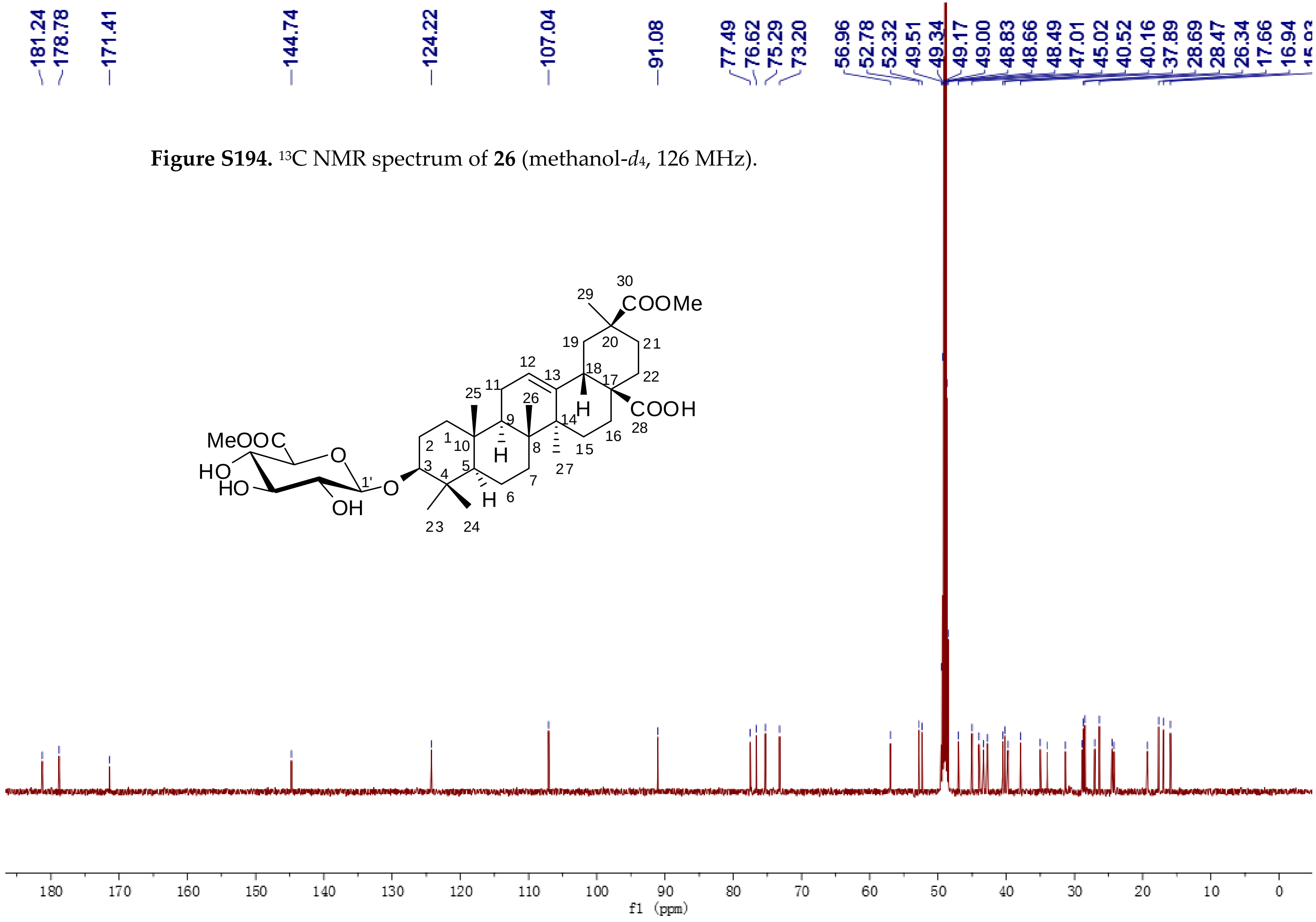

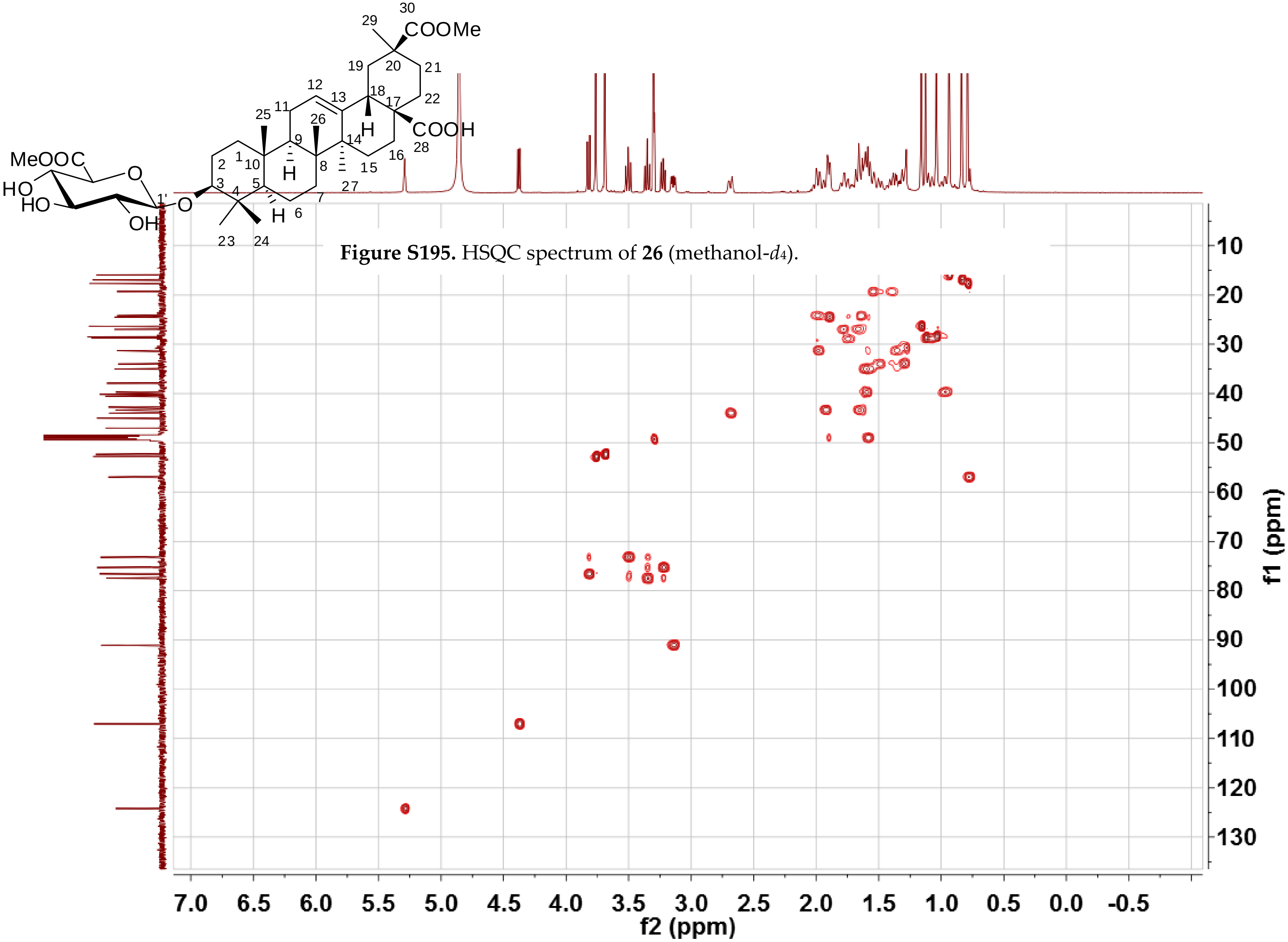

Figure S195. HSQC spectrum of **26** (methanol-*d*4).

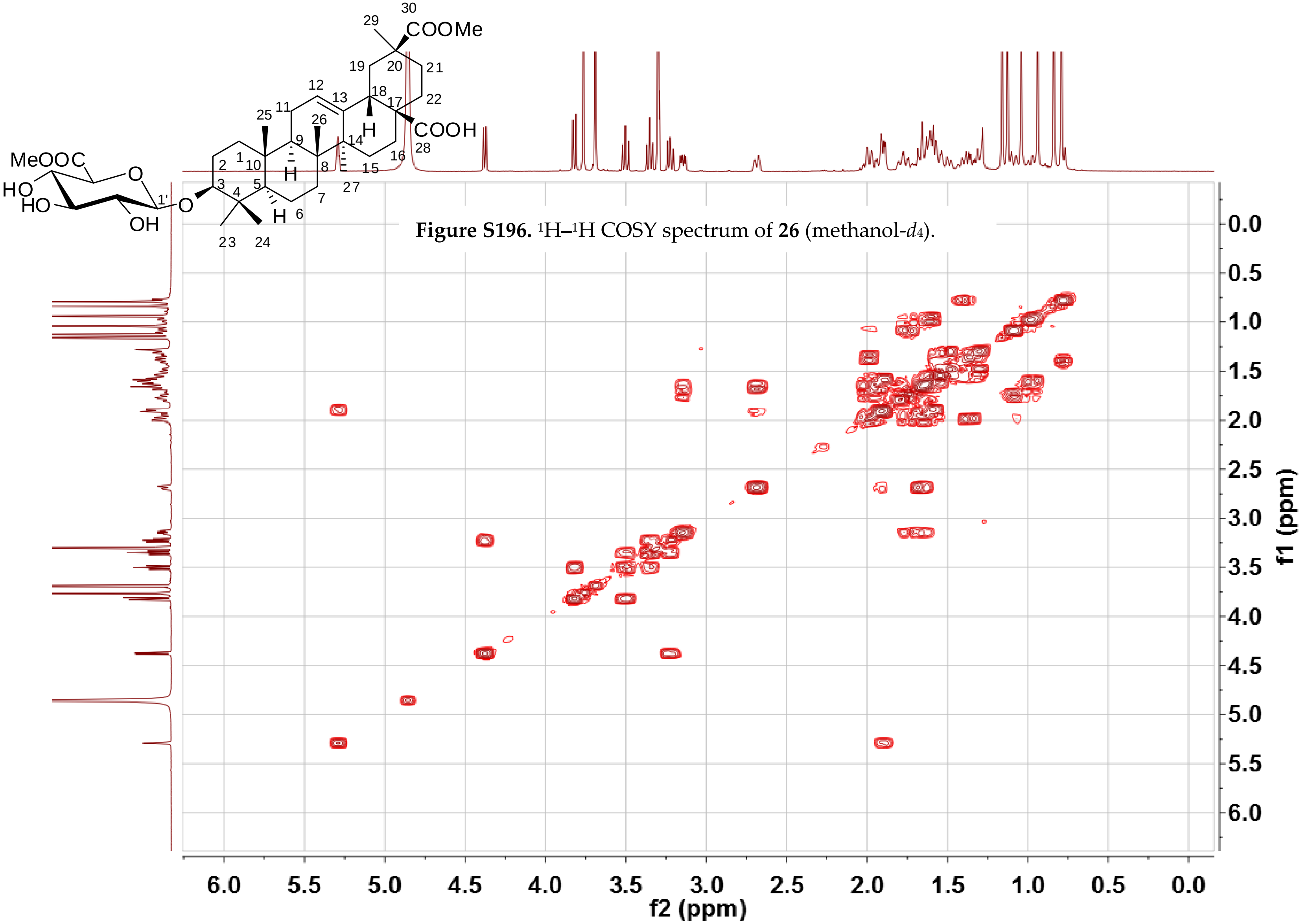

Figure S196. ^{1}H–^{1}H COSY spectrum of **26** (methanol-d_4).

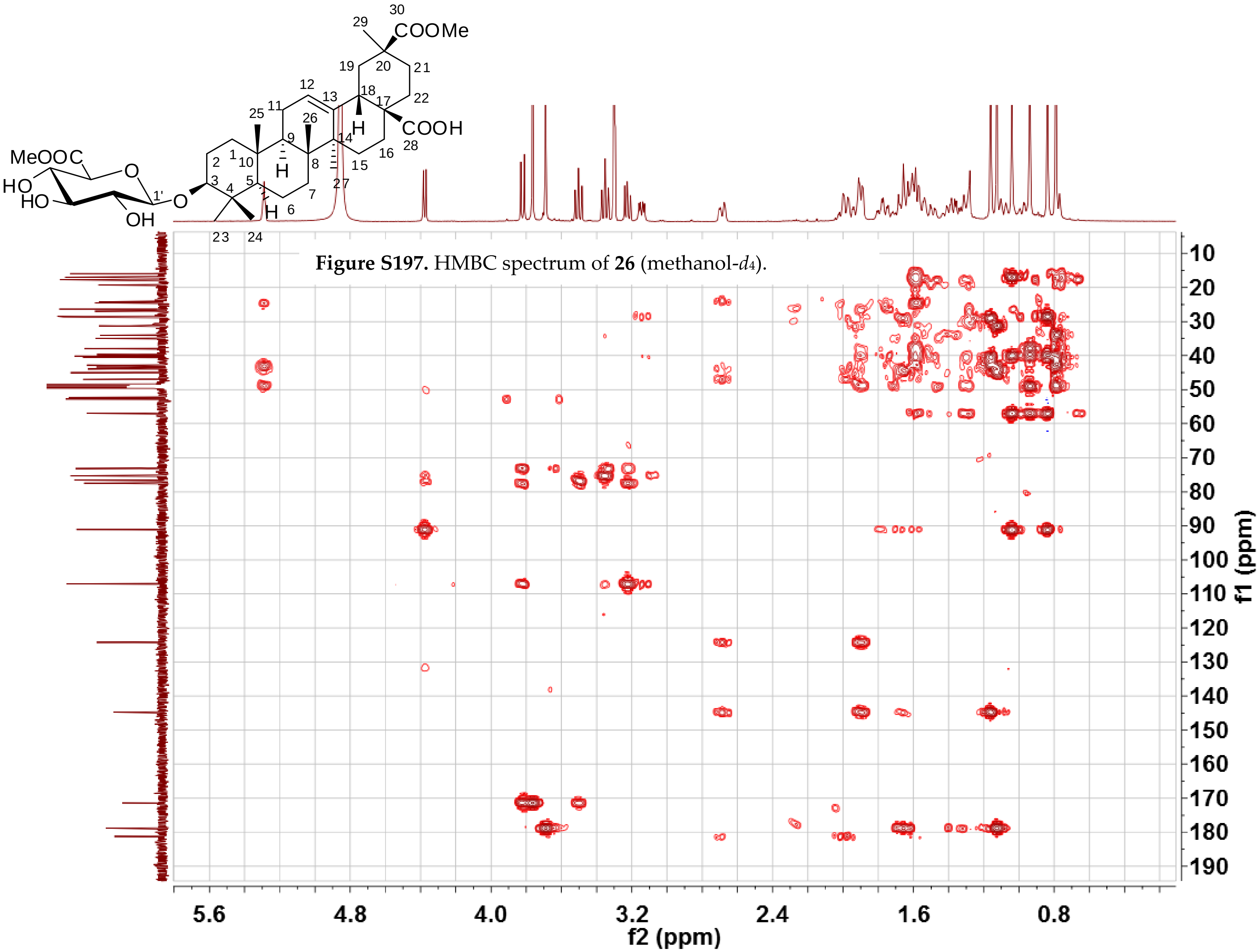

Figure S197. HMBC spectrum of **26** (methanol-*d*4).

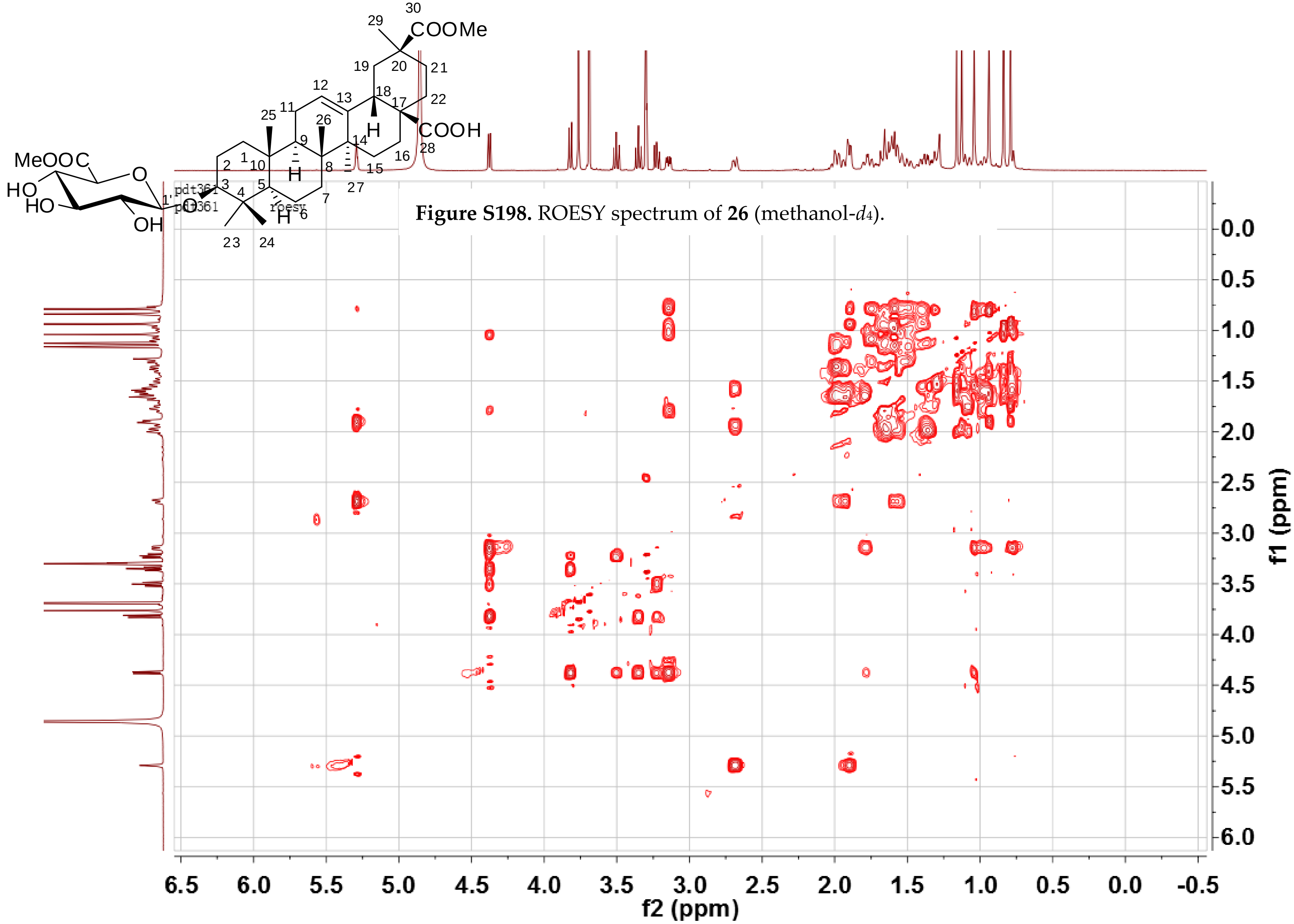

Figure S198. ROESY spectrum of **26** (methanol-d_4).
